# Alternating oligo(*o*,*p*-phenylenes) *via* ruthenium catalyzed diol–diene benzannulation: orthogonality to cross-coupling enables *de novo* nanographene and PAH construction†
†Electronic supplementary information (ESI) available: Experimental procedures and spectroscopic data for all new compounds (^1^H NMR, ^13^C NMR, IR, HRMS), including images of NMR spectra. Single crystal X-ray diffraction data for compounds **11** (1856649), **14** (1856650) and **16a** (1856651). For ESI and crystallographic data in CIF or other electronic format see DOI: 10.1039/c8sc03236j


**DOI:** 10.1039/c8sc03236j

**Published:** 2018-08-30

**Authors:** Zachary A. Kasun, Hiroki Sato, Jing Nie, Yasuyuki Mori, Jon A. Bender, Sean T. Roberts, Michael J. Krische

**Affiliations:** a University of Texas at Austin , Department of Chemistry , Austin , TX 78712 , USA . Email: mkrische@mail.utexas.edu ; Email: roberts@cm.utexas.edu

## Abstract

Ruthenium(0) catalyzed diol–diene benzannulation is applied to the conversion of oligo(*p*-phenylene vinylenes) **2a–c**, **5** and **6** to alternating oligo(*o*,*p*-phenylenes) **10a–c**, **11–13**. Orthogonality with respect to conventional palladium catalyzed biaryl cross-coupling permits construction of *p*-bromo-terminated alternating oligo(*o*,*p*-phenylenes) **10b**, **11–13**, which can be engaged in Suzuki cross-coupling and Scholl oxidation. In this way, structurally homogeneous nanographenes **16a–f** are prepared. Nanographene **16a**, which incorporates 14 fused benzene rings, was characterized by single crystal X-ray diffraction. In a similar fashion, *p*-bromo-terminated oligo(*p*-phenylene ethane diol) **9**, which contains a 1,3,5-trisubstituted benzene core, is converted to the soluble, structurally homogeneous hexa-*peri*-hexabenzocoronene **18**. A benzothiophene-terminated pentamer **10c** was prepared and subjected to Scholl oxidation to furnish the helical bis(benzothiophene)-fused picene derivative **14**. The steady-state absorption and emission properties of nanographenes **14**, **16a**,**b**,**d**,**e**,**h** and **18** were characterized. These studies illustrate how orthogonality of ruthenium(0) catalyzed diol–diene benzannulation with respect to classical biaryl cross-coupling streamlines oligophenylene and nanographene construction.

## Abstract 2

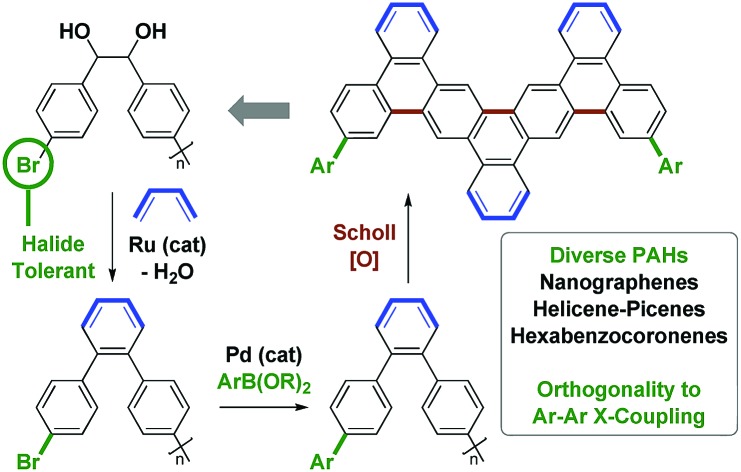
Ruthenium(0) catalyzed diol–diene benzannulation enables formation of *p*-bromo-terminated alternating oligo(*o*,*p*-phenylenes) and, therefrom, diverse PAH materials.

## Introduction

Oligophenylenes comprise a wide-ranging and varied class of PAH compounds, which due to their tunable physical properties are promising candidates for organic photovoltaic materials.^1^ Additionally, Scholl oxidation^2^–^4^ of oligophenylenes and related structures enables access to structurally homogeneous nanographene materials.^5^,^6^ Despite the longstanding importance of oligophenylenes and nanographenes to the field of molecular electronics, their construction *de novo* remains challenging and relatively few methods for their synthesis are broadly applied. Strategies involving biaryl cross-coupling^7^ followed by Scholl oxidation^2^–^4^ or palladium catalyzed cyclo-dehydrohalogenations^8^–^10^ are among the most powerful. While many other benzannulation protocols have been reported,^11^ scalable, non-cryogenic catalytic methods that are *orthogonal* to biaryl cross-coupling would be especially valuable in terms of streamlining access to PAH chemical space.

**Scheme 1:**
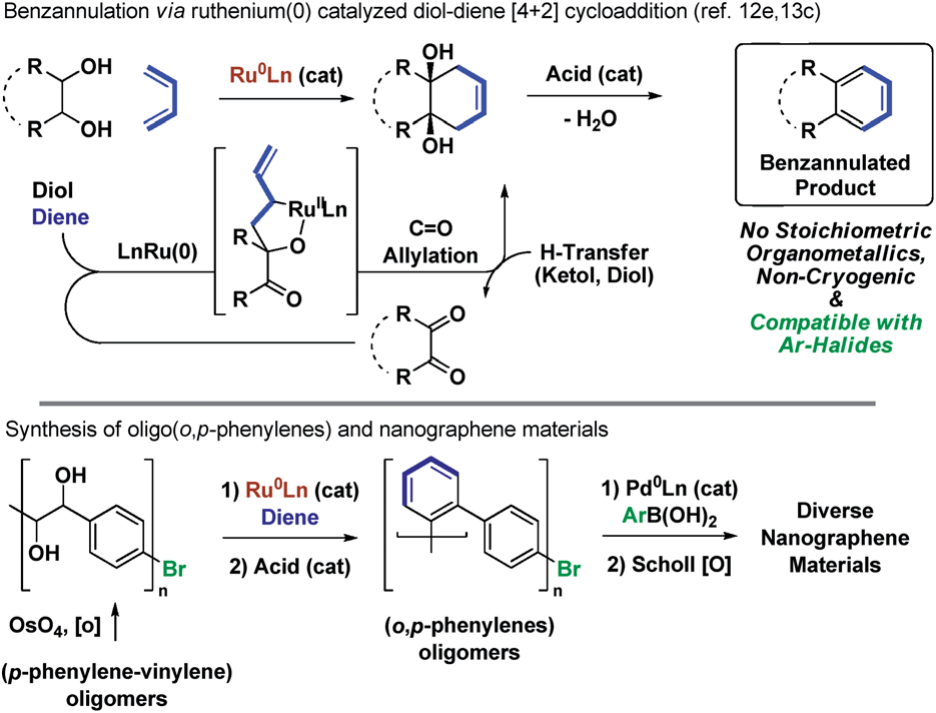
Alternating *o*,*p*-oligophenylenes and nanographenes *via* ruthenium catalyzed diol–diene benzannulation.

Utilizing the concept of alcohol-mediated carbonyl addition,^12^ a ruthenium(0) catalyzed diene–diol [4 + 2] cycloaddition was recently developed in our laboratory (Scheme 1).12e,13a,b Aromatization of the cycloadducts occurs readily, enabling access to products of benzannulation from abundant diol and diene reactants.13*c* In an initial application of this method, a homologous series of rod-like triple-stranded phenylene cages was prepared.13d,^14^ This exercise suggested the feasibility of modular nanographene syntheses wherein diol–diene benzannulation is used to generate bromide-containing oligophenylenes amenable to late-stage diversification through metal catalyzed biaryl cross-coupling followed by Scholl oxidation. In fulfillment of this objective, we report syntheses of alternating oligo(*o*,*p*-phenylenes)^15^*via* ruthenium(0) catalyzed diol–diene benzannulation and, therefrom, structurally homogeneous nanographene materials containing as many as 22 aromatic rings.

## Research design and methods

### Synthesis of oligo(*o*,*p*-phenylenes) and related PAH compounds

**Scheme 2:**
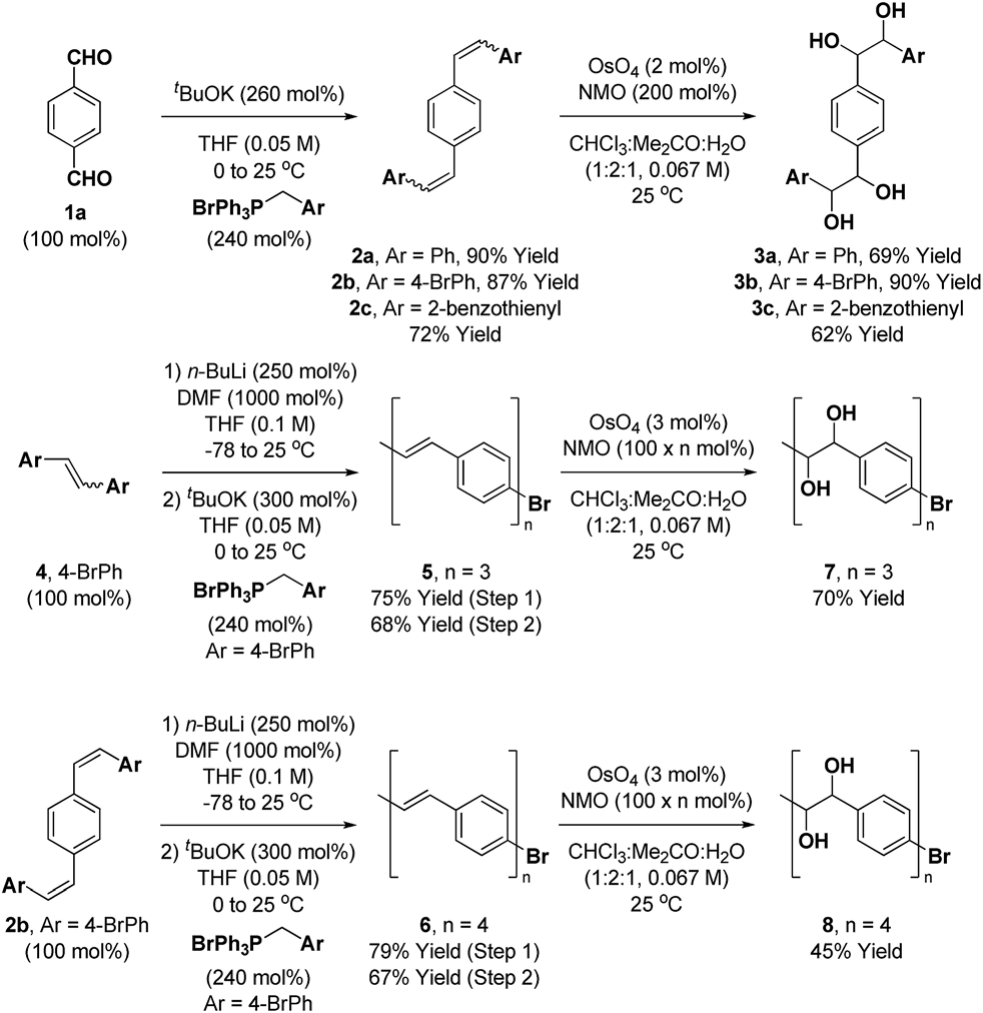
Synthesis of oligo(*p*-phenylene vinylene) diols **3a–3c**, **7** and **8**. ^a^ Yields are of material isolated by silica gel chromatography. See ESI† for further experimental details.

The synthesis of the requisite oligo(*p*-phenylene vinylene) diols **3a–3c** is readily accomplished through Wittig olefination (Scheme 2).^16^,^17^ Thus, in close analogy to the literature procedure,17*d* terephthalaldehyde **1a** was exposed to the indicated phosphonium salts in the presence of ^*t*^BuOK to furnish the respective oligo(*p*-phenylene vinylenes) **2a–c** in good yields. Alternate bases such as KOH, NaOH, NaO^*t*^Bu, ^*n*^BuLi and lithium diisopropylamide (LDA) led to incomplete conversion and the use of Horner–Wadsworth–Emmons (HWE) reagents was accompanied by substantial quantities of homo-coupling byproducts.^18^ Dihydroxylation of the oligo(*p*-phenylene vinylenes) **2a–c** proved challenging due to competing oxidative cleavage to form aldehyde byproducts.^19^ Upjohn dihydroxylation conditions using *N*-methylmorpholine *N*-oxide (NMO)^20^ as the terminal oxidant attenuated this side reaction, delivering the oligo(*p*-phenylene vinylene) diols **3a–c** in good to excellent yields. In a similar manner, a three-directional synthesis of *tris*-diol **9** was accomplished from benzene-1,3,5-tricarbaldehyde **1b** (eqn (1)).13*d*1
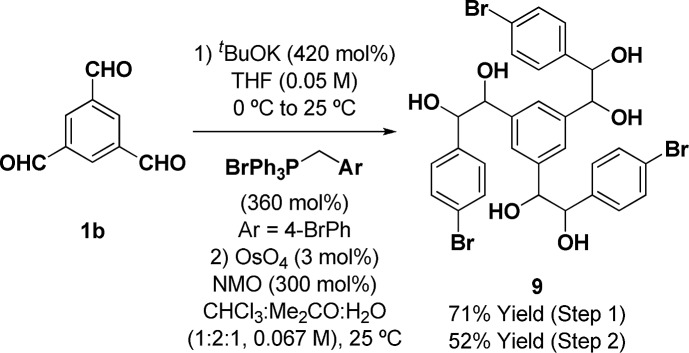



The synthesis of higher oligo(*p*-phenylene vinylene) diols **7** and **8** was accomplished in an iterative fashion through homologation of dibromo-styrene **4** and the 4-bromo-terminated oligo(*p*-phenylene vinylene) **2b** (Scheme 2). Thus, lithiation of **4** and **2b** followed by treatment with DMF provided the respective formyl derivatives,^21^ which upon Wittig olefination furnished the homologous 4-bromo-terminated oligo(*p*-phenylene vinylenes) **5** and **6**. Exposure of the oligo(*p*-phenylene vinylenes) **5** and **6** to Upjohn dihydroxylation^20^ provided the oligo(*p*-phenylene vinylene) diols **7** and **8**. To minimize competitive oxidative cleavage to form aldehydes observed in the formation of **8**,^19^ a higher loading of OsO_4_ was required to shorten the reaction time. Additionally, for the synthesis of **7** and **8**, use of the predominantly (*Z*)-selective Wittig olefination was important, as the less soluble products of (*E*)-selective HWE olefination were difficult to engage in dihydroxylation.

As the ruthenium(0) catalyzed [4 + 2] cycloaddition can be conducted from the ketol oxidation level,12e,^13^ routes involving benzoin condensation were explored. The crossed-benzoin condensation of terephthalaldehyde **1a** with benzaldehyde occurred efficiently using an *N*-heterocyclic carbene (NHC) catalyst, providing ketol *dehydro*-**3a** in good yield (eqn (2)).^22^ However, these conditions were quite substrate dependent and attempted benzoin condensation of 4-bromo benzaldehyde and 2-benzothiophene carboxaldehyde was inefficient due to competing self-condensation.2
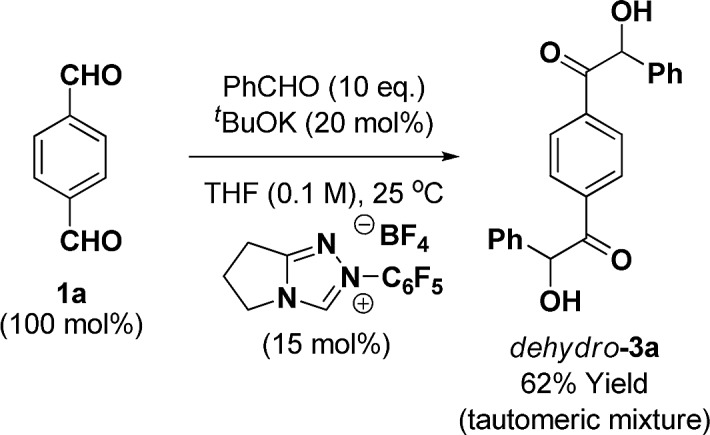



Benzannulation of oligo(*p*-phenylene vinylene) diols **3a–3c**, **7–9** to form alternating oligo(*o*,*p*-phenylenes) **10a–c**, **11–13** was next explored (Table 1). To our delight, the ruthenium(0) catalyzed cycloaddition of 1,3-butadiene with oligo(*p*-phenylene vinylene) diols **3a–3c**, **7–9** proceeded smoothly in the presence of a carboxylic acid cocatalyst to furnish the corresponding cyclohexene diols in good to excellent yield.12e,^13^ Subsequent exposure of the cycloadducts to substoichiometric quantities of *p*-toluenesulfonic acid (*p*-TsOH) resulted in dehydration to form the alternating oligo(*o*,*p*-phenylenes) **10a–c**, **11–13** in moderate to high yields.13*c* The dehydration reaction is highly temperature dependent and minor deviations from the optimal temperatures identified for each substrate caused a significant decrease in yield. Perhaps related to this observation, one-pot cycloaddition–dehydration, which was effective for the synthesis of fluoranthenes and acenes,13*c* was less efficient in the context of the present oligo(*o*,*p*-phenylene) syntheses.

**Table 1:** Ruthenium(0) catalyzed benzannulation of oligo(*p*-phenylene vinylene) diols **3a–3c**, **7–9** to form oligo(*o*,*p*-phenylenes) **10a–c**, **11–13**^*a*^

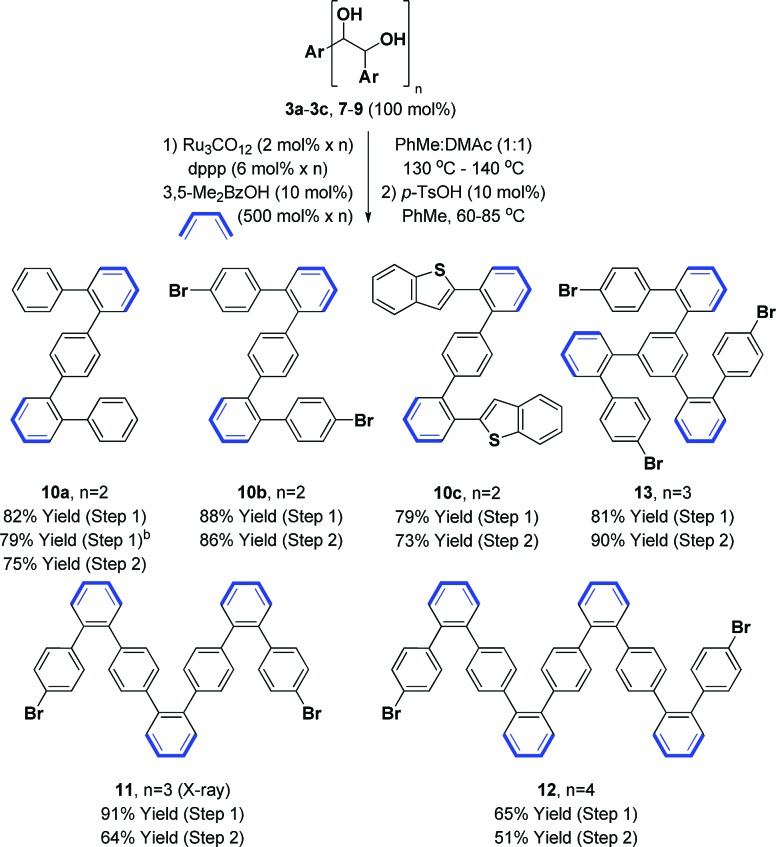

^*a*^Yields are of material isolated by silica gel chromatography.

^*b*^Yield from ketol *dehydro*-**3a**. See ESI for further experimental details.

The alternating oligo(*o*,*p*-phenylenes) **10a–c**, **11–13** prepared by our methods raise numerous possibilities for the synthesis of diverse PAH compounds, including helicenes, graphene nanodots and nanoribbons. To illustrate, the benzothiophene derived oligomer **10c** was subjected to Scholl oxidation conditions employing anhydrous FeCl_3_ (ref. 3b) to form the *S*-doped helical picene derivative **14**, which was characterized by single crystal X-ray diffraction (eqn (3)).^23^ The regioisomeric compound *iso*-**14** was not observed. This result is consistent with the findings of Hilt and co-workers, who observe similar regioselectivities in related FeCl_3_-mediated Scholl oxidations of alternating oligo(*o*,*p*-phenylenes).^24^3
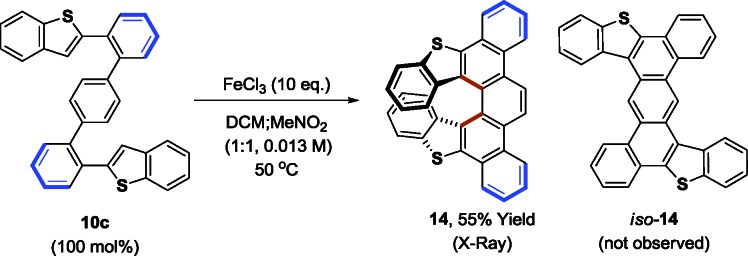



**Scheme 3:**
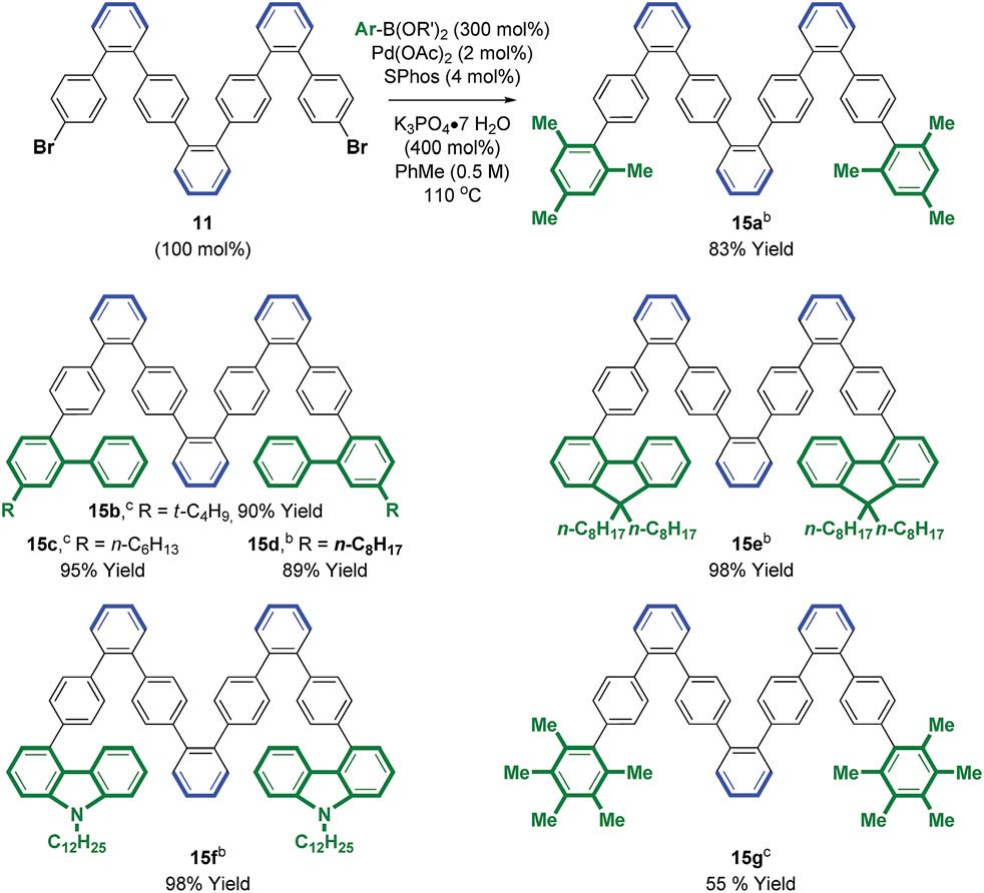
Palladium catalyzed cross-coupling of **11** to form oligophenylenes **15a–g**. ^a^ Yields are of material isolated by silica gel chromatography. See ESI† for further experimental details. ^b^ Pinacol boronate. ^c^ Boronic acid.

**Scheme 4:**
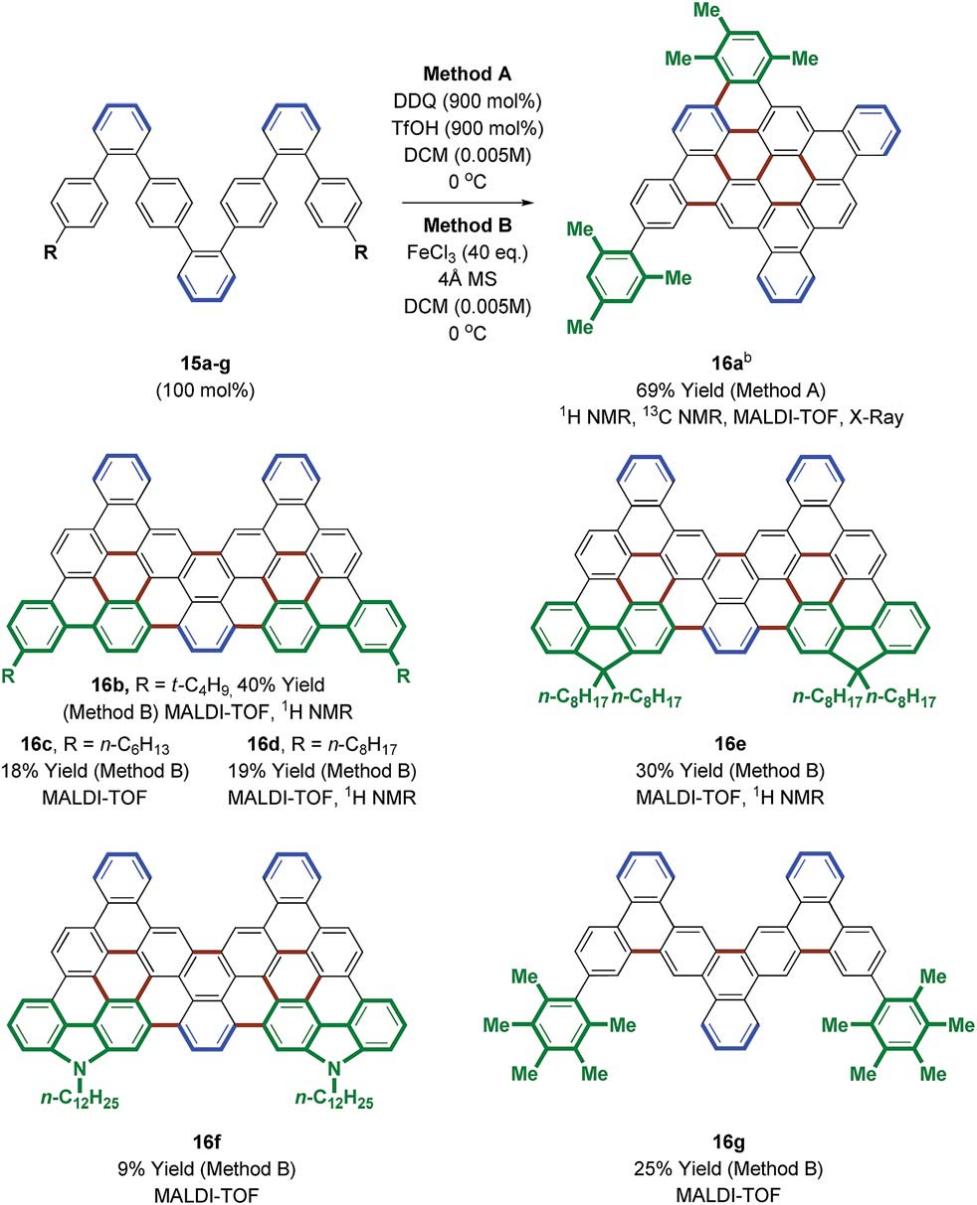
Scholl oxidation of oligophenylenes **15a–g** to form nanographenes **16a–g**. ^a^ Yields are of material isolated by silica gel chromatography or by trituration. See ESI† for further experimental details. ^b^ DDQ (600 mol%), TfOH (600 mol%).

**Fig. 1:**
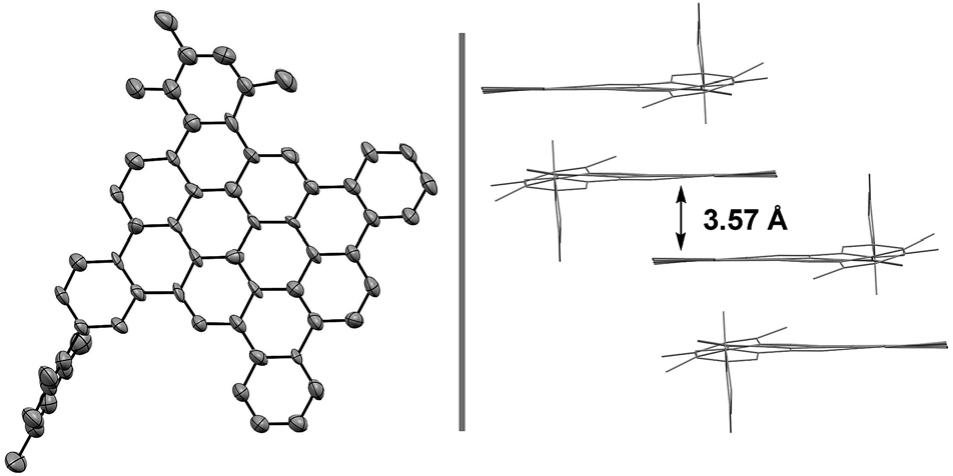
Single-crystal X-ray diffraction data of nanographene **16a**. Displacement ellipsoids are scaled to 50% probability. Hydrogens have been omitted for clarity and packing in the solid state. See ESI† for further structural details.

Access to bromo-terminated oligomers, such as **11**, led us to explore modular nanographene syntheses wherein late-stage diversification through metal catalyzed biaryl cross-coupling is followed by Scholl oxidation (Schemes 3 and 4). Toward this end, heptaphenylene **11** was subjected to Suzuki cross-coupling conditions^25^ with aryl boronate or aryl boronic acid partners that were selected to facilitate Scholl oxidation and confer solubility to the resultant nanographenes (Scheme 3).^26^ Thus, heptaphenylene **11** was converted to the bis(2,4,6-trimethylphenyl) nonaphenylene **15a**, which was exposed to DDQ and triflic acid.^27^ However, as confirmed by single crystal X-ray crystallography (Fig. 1), Scholl oxidation was accompanied by aryl and methyl migration to form **16a**, which was highly soluble in chloroform. Skeletal rearrangement is often observed during Scholl oxidation and can be difficult to predict.3b,^28^ Alternate Scholl oxidation conditions resulted in diminished yields of **16a** or produced complex mixtures of numerous products. The structure of nanographene **16a**, which contains 14 fused aromatic rings, is nevertheless quite remarkable, as crystal structures of large planar PAH compounds remain quite uncommon.^29^,^30^ The supramolecular structure of nanographene **16a** in the solid state is dominated by π–π stacking interactions, consistent with King's observation that large flanking groups on nanographenes disrupt the herringbone packing typically seen in crystal structures of PAH materials.^26^,^31^,^32^

An effort was made to design oligophenylenes that are less prone to skeletal rearrangement under Scholl oxidation conditions. It was recognized that Scholl oxidation to form triangular tribenzo[a,g,m]coronene motifs occurs in a highly efficient manner.^33^ Hence, Suzuki coupling was conducted with *ortho*-biarylboron reagents to form oligomers **15b–f** (Scheme 3). Additionally, oligomer **15g**, which incorporates pentamethylphenyl termini, was targeted, as methyl migration is not possible on the fully substituted aromatic ring. Indeed, Scholl oxidation using either DDQ and triflic acid^27^ or FeCl_3_ in the presence of molecular sieves^34^ gave **16b–g** without rearrangement. Nanographenes **16b**, **16d** and **16e** were sparingly soluble in chloroform and nanographenes **16c**, **16f** and **16g** were highly insoluble. MALDI-TOF mass spectrometry was used to characterize all compounds, as well as ^1^H NMR for **16b**, **16d** and **16e**. Finally, an alternate strategy for Scholl oxidation in the absence of skeletal rearrangement entailed conversion of heptaphenylene **11** to the bis(*n*-octyl ethyl) **15h***via* copper catalyzed C–O bond formation.^35^ Scholl oxidation of bis(*n*-octyl ethyl) **15h** under DDQ and triflic acid conditions^27^ gave the nanographene **16h** in 65% yield (eqn (4)). This derivative was soluble enough to be characterized by ^1^H and ^13^C NMR, in addition to MALDI-TOF mass spectrometry.4
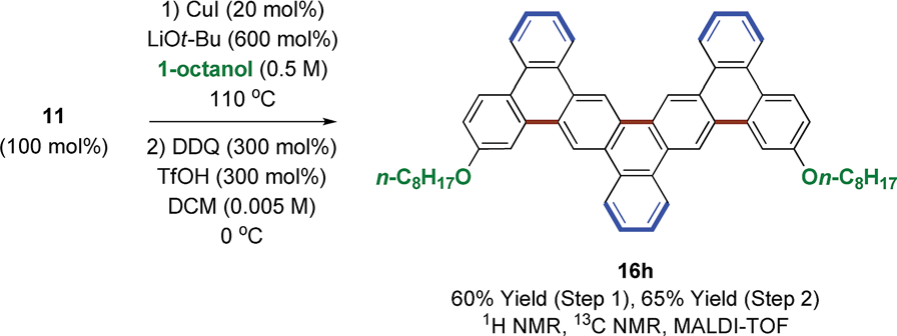



Hexa-*peri*-hexabenzocoronenes (HBCs) represent yet another class of fully benzenoid PAHs that have garnered interest as potential materials for opto-electronic devices.^6^ However, current methods available for HBC synthesis are limited. This is especially true for HBCs with low symmetry,^36^ electron deficient HBCs^37^ or those substituted at the bay region.^38^ The synthesis of the electron deficient *D*_3h_ symmetric HBC **17**, which incorporates bromo-substituents in the bay region, was achieved through Scholl oxidation of the branched heptaphenylene **13** under DDQ and triflic acid conditions (eqn (5)).^27^ Although the resulting HBC **17** is quite insoluble, Sonogashira coupling^39^ occurred in good yield to furnish the chloroform-soluble **18**, which was characterized by ^1^H and ^13^C NMR and MALDI-TOF mass spectrometry.5
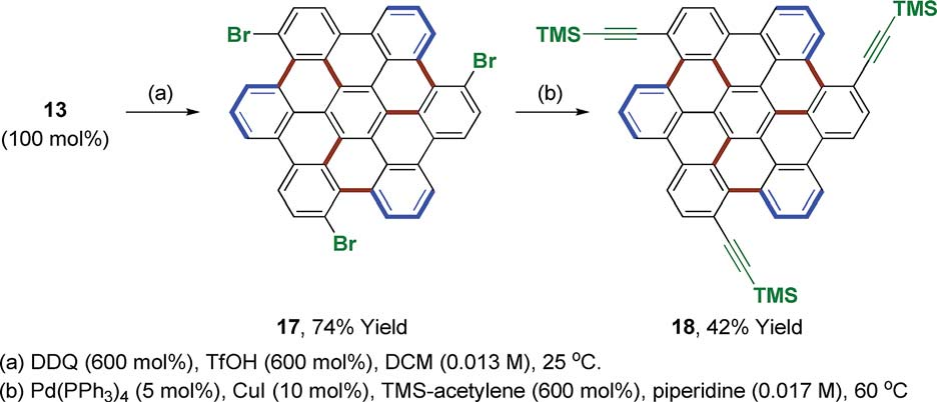



### Spectroscopic analysis

**Fig. 2:**
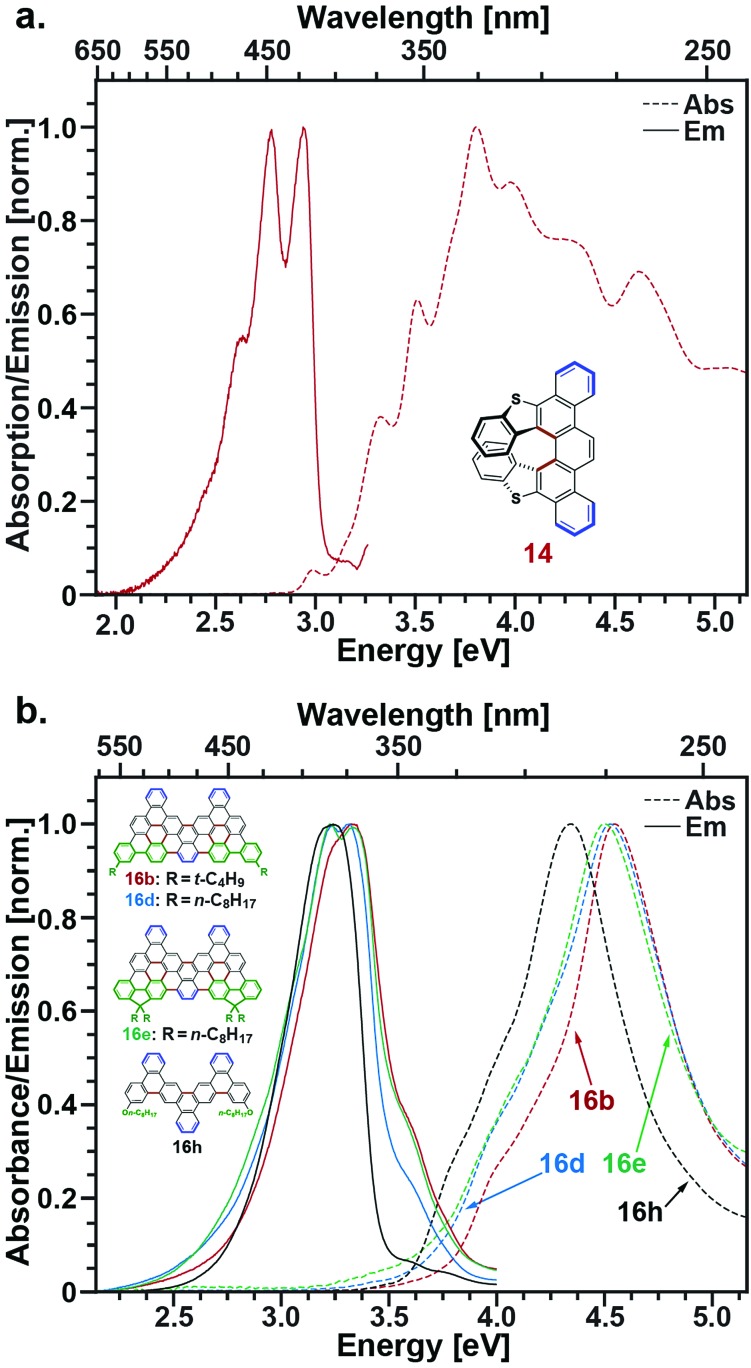
(a) Absorption (dashed) and emission (solid) of **14**. (b) Absorption (dashed) and emission (solid) of **16b** (red), **16d** (blue), **16e** (green), and **16h** (black).

The photophysical properties of a subset of the molecules presented herein that were sufficiently soluble in dichloromethane (**14**, **16a**,**b**,**d**,**e**,**h**, **18**) were characterized by steady-state absorption and fluorescence spectroscopies. Experimental details and spectra measured for **16a** and **18** are included in the ESI.† Fig. 2a displays absorption and emission spectra of the helicene **14**. This compound features a broad absorption profile that rises from ∼2.9 eV and contains a series of sharp resonances best explained by comparison with similar picene and helicene examples previously explored.^40^,^41^ In particular, the absorption spectrum can be qualitatively explained as a combination of low-lying higher helicene transitions^40^ and high-energy transitions associated with the fused thiophene rings contained in the picene backbone, as studied by Morin and coworkers.41*b* Likewise, the emission spectrum of **14** is consistent with other higher helicenes, particularly [7]-helicene, the first in the series for which ring overlap begins with an increasing number of rings.40e,g It is worth noting that helicenes, particularly higher helicenes containing thiophenes, have attracted interest as chiral nonlinear optical materials.^42^ To our knowledge, compound **14** is the first reported example of a picene-helicene hybrid and we believe its chiroptical properties will be of future interest.

Fig. 2b displays absorption and emission spectra of the nanographenes **16b**,**d**,**e**, and **h**. Although these compounds feature the largest extended π-conjugated systems among the compounds we have characterized spectroscopically, their absorption and emission spectra peak at higher energies than compounds **14**, **16a**, and **18**. This is best explained by the sole presence of arm-chair edges along the periphery of these materials, which are thought to bestow nanographenes with larger bandgaps.^43^ Interestingly, reducing the number of rings along the nanographene short axis, as is done for **16h**, acts to reduce its optical bandgap relative to **16b**, **16d**, and **16e** even though this also reduces the size of its π-system.

## Conclusions

In summary, we report the synthesis of oligophenylenes and various PAH materials constructed though the use of Ru(0)-catalyzed diol–diene cycloaddition coupling.^4^ Oligo-1,2-diols were constructed *via* iterative Wittig coupling and dihydroxylation. Furthermore, orthogonality to Pd-catalyzed cross coupling allows for bromo-terminated polyphenylenes that could be functionalized to provide various nanographenes **16a–h** after Scholl oxidation. Additionally, Scholl oxidation of **10c** and **13** provided benzothiophene helical picene **14** and hexa-*peri*-hexabenzocoronene **18**, respectively. Thus, we have demonstrated the use of Ru(0) catalyzed diol–diene benzannulation in the fabrication of three distinct types of PAH materials. Photophysical analysis of **14**, **16a**,**b**,**d**,**e**,**h** and **18** demonstrated that nanographenes prepared by these synthetic routes can display highly variable optical properties, which make these methods useful for the preparation of organic electronic materials. Future studies will focus on the development of related methods for alcohol-mediated benzannulation and their application to PAH construction, including the use of symmetric 2,3-diaryl-substituted butadiene building blocks.

## Conflicts of interest

There are no conflicts to declare.

## Supplementary Material

Supplementary information

Crystal structure data
