# Supplementary material for: Alternating oligo(o,p-phenylenes) via ruthenium catalyzed diol–diene benzannulation: orthogonality to cross-coupling enables de novo nanographene and PAH construction
Source: Chem Sci. 2018 Aug 30;9(40):7866–73. doi: 10.1039/c8sc03236j (PMC6194800; doi:10.1039/c8sc03236j)
Supplement: Supplementary file 1 [file SC-009-C8SC03236J-s001.pdf]

Electronic Supplementary Material (ESI) for Chemical Science.
This journal is © The Royal Society of Chemistry 2018

Supporting Information

Alternating oligo(*o,p*-Phenylenes) *via* Ruthenium Catalyzed Diol-Diene Benzannulation: Orthogonality to Cross-Coupling Enables *De Novo* Nanographene and PAH Construction

Zachary A. Kasun, Hiroki Sato, Jing Nie, Yasuyuki Mori, Jon A. Bender, Sean T. Roberts and Michael J. Krische*

University of Texas at Austin, Department of Chemistry,

Austin, TX 78712, USA

Table of Contents

General Comments………………………………………………………………………… S1

Synthesis and Characterization of Oligo-Vinylphenylenes and Vicinal 1,2-Diols……………... S2

Synthesis and Characterization of Cycloadducts and Oligophenylenes **10a-c**, **11**, **12**, and **13**…S49

Synthesis and Characterization of Helicene **14**……….………………..……….……………….S81

Synthesis and Characterization of HBC **17** and **18**…………………..…………..…………S84

Synthesis and Characterization of Biphenyl Boronic Acids and Boronates……....………..…S88

Synthesis and Characterization of Oligophenylenes **15a-h** via Cross Coupling……….…….S104

Synthesis and Characterization of Nanographenes **16a-h** ……………..……………………..… S128

Photophysical Properties……………………………………………………………………S148

Single Crystal Diffraction Data …………………………………………………………… S151

References………………………….………………………………………………… S206

General Comments

All glassware was oven dried at 120 °C overnight and cooled in a desiccator. All ruthenium catalyzed reactions were carried in sealed pressure tubes (13 x 100 mm). THF was purified by distillation from sodium and benzophenone immediately before use. Ruthenium carbonyl [$Ru_3(CO)_{12}$], dppp, 3,5-dimethylbenzoic acid were purchased from commercial suppliers and used as received. Analytical thin-layer chromatography (TLC) was carried out using 0.25 mm commercial silica gel plates. Visualization was accomplished with UV light followed by dipping in a cerium ammonium molybdate solution and heating. Purification of reaction products was carried out by flash column chromatography using 40-63 μm silica gel. ^{1}H NMR (600, MHz, 500 MHz, 400 MHz) and ^{13}C NMR (150 MHz, 125 MHz, 100 MHz) ^{31}P NMR (160 MHz) were recorded with a Varian Gemini 400, Oxford Instruments 600, or Bruker Avance III 500 equipped with Prodigy Cryoprobe in $CDCl_3$ solutions unless otherwise noted. ^{13}C NMR spectra were run with broadband decoupling. Chemical shifts for ^{1}H and ^{13}C are reported in parts per million (ppm) downfield from TMS, using residual $CHCl_3$ (7.26 ppm and triplet at 77.0 ppm, respectively). The following abbreviations are used: m (multiplet), s (singlet), d (doublet), t (triplet), q (quartet), dd (doublet of doublets), etc. Infrared spectra were recorded on a Thermo Nicolet 380 spectrometer. Mass spectra (MS) were obtained on a Water Micromass AutoSpec Ultima (HR-CI), Agilent Technologies 6530 Accurate Mas Q-TOF (HR-ESI, HR-APCI, HR-APPI), and Applied Biosystems Voyager DE-Pro (MALDI-TOF, 337nm N_2 laser, 3 ns pulse, 20,000 kV source) are reported as m/z. Masses are reported for the molecular ion (M-H, M, M+H, M+Na, M+K, or other suitable adduct, as noted). Tetracyanoquinodimethane (TCNQ) was used as a matrix in MALDI-TOF experiments.

Synthesis and Characterization Data of Oligo-Vinylphenylenes and Vicinal 1,2-Diols

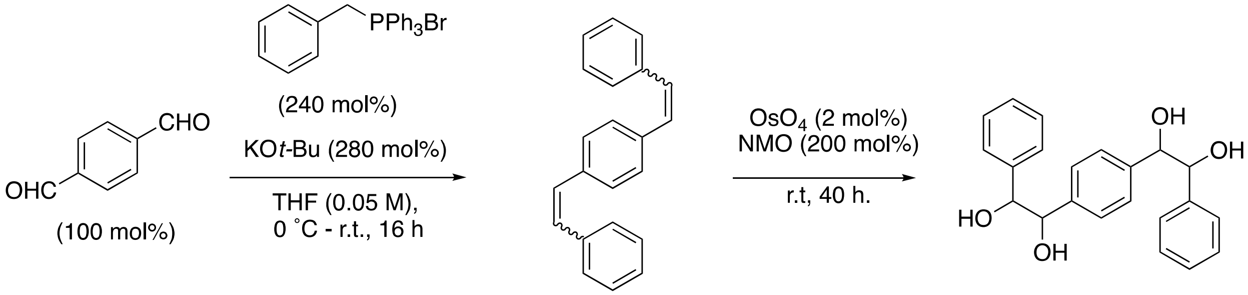

1a **2a** **3a**

<u>1,4-distyrylbenzene (**2a**):</u>

To a solution of KO*t*-Bu (1.12 g, 28.0 mmol, 280 mol%) in anhydrous THF (100 mL) cooled to 0 ˚C was added phosphonium salt (10.4 g, 24.0 mmol, 240 mol%). The mixture was allowed to stir at the same temperature for 30 min followed by the addition of terephthalaldehyde (1.34 g, 10.0 mmol, 100 mol%) in THF (100 mL) dropwise over 30 min. The reaction was then warmed to room temperature and allowed to stir for 16 hours. The solution was concentrated under vacuum followed by addition of water (50 mL). The aqueous layer was then extracted with Et_2O (3 x 25 mL) and the combined organic layers were washed with brine (50 mL), dried (Na_2SO_4) and filtered. Evaporation under reduced pressure provided an oily residue which was subjected to flash column chromatography (SiO_2; hexanes:ethyl acetate = 99:1) to furnish the title compound **2a** (2.54 g, 9.0 mmol) in 90% yield as a white solid.

<u>TLC (SiO_2)</u>: R_f = 0.64 (hexanes : ethyl acetate= 90:10).

<u>^{1}H NMR</u>: (400 MHz, $CDCl_3$): δ = 7.55–7.52 (m, 8H), 7.40–7.36 (m, 4H), 7.29–7.24 (m, 2H), 7.13 (t, *J* = 1.4 Hz, 4H) ppm.

<u>^{13}C NMR:</u> (100 MHz, $CDCl_3$): δ = 137.3, 136.7, 128.7, 128.6, 128.3, 127.6, 126.8, 126.5 ppm.

<u>HRMS</u>: (CI^+) Calculated for $C_{22}H_{18}$ [M^+] = 282.1409, Found 282.1407.

<u>FTIR</u>: (neat): 2357, 969, 816, 691 cm^{-1}.

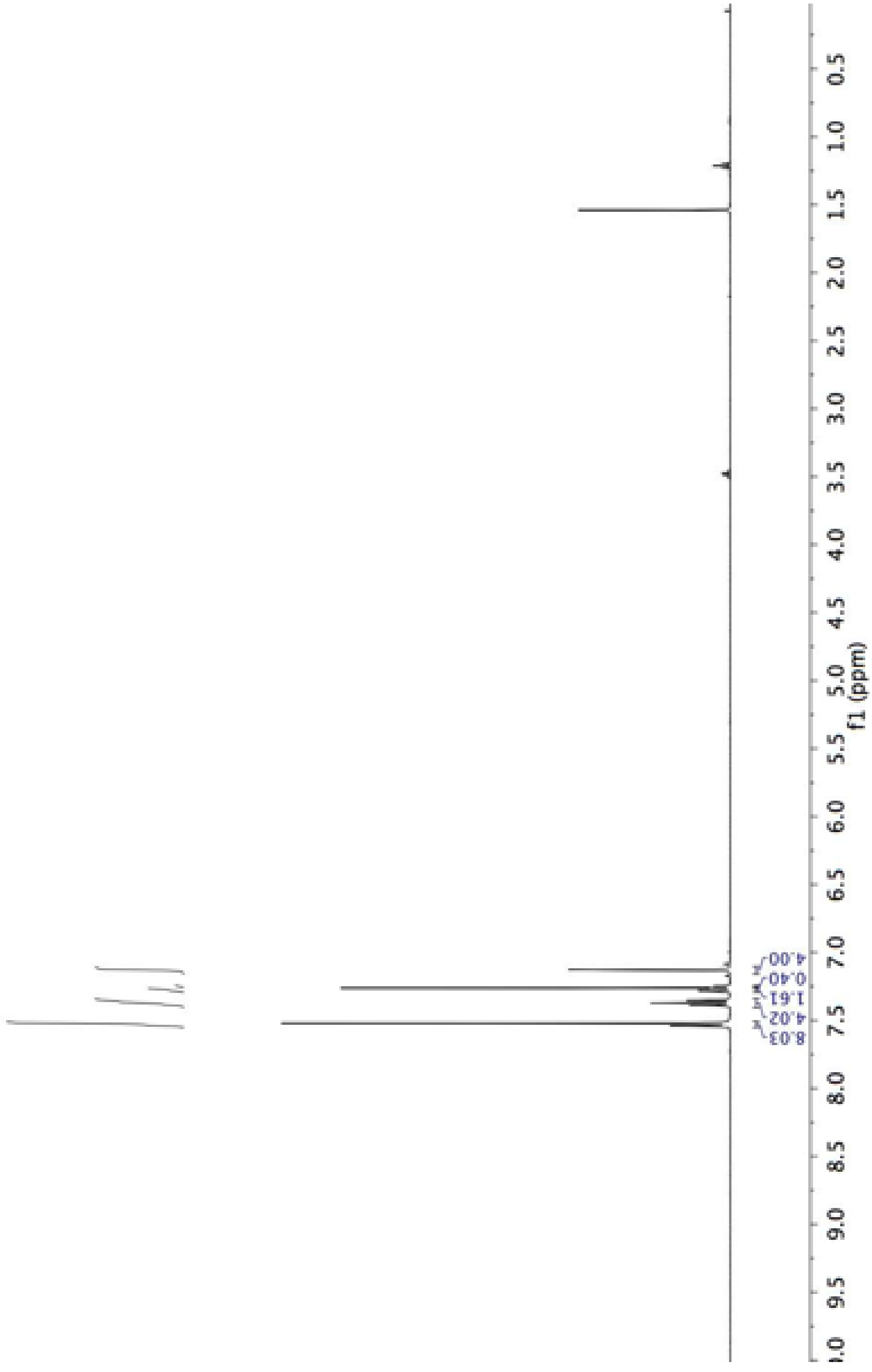
f1 (ppm)
0.5
1.0
1.5
2.0
2.5
3.0
3.5
4.0
4.5
5.0
5.5
6.0
6.5
7.0
7.5
8.0
8.5
9.0
9.5
4.00
0.40
1.61
4.02
8.03

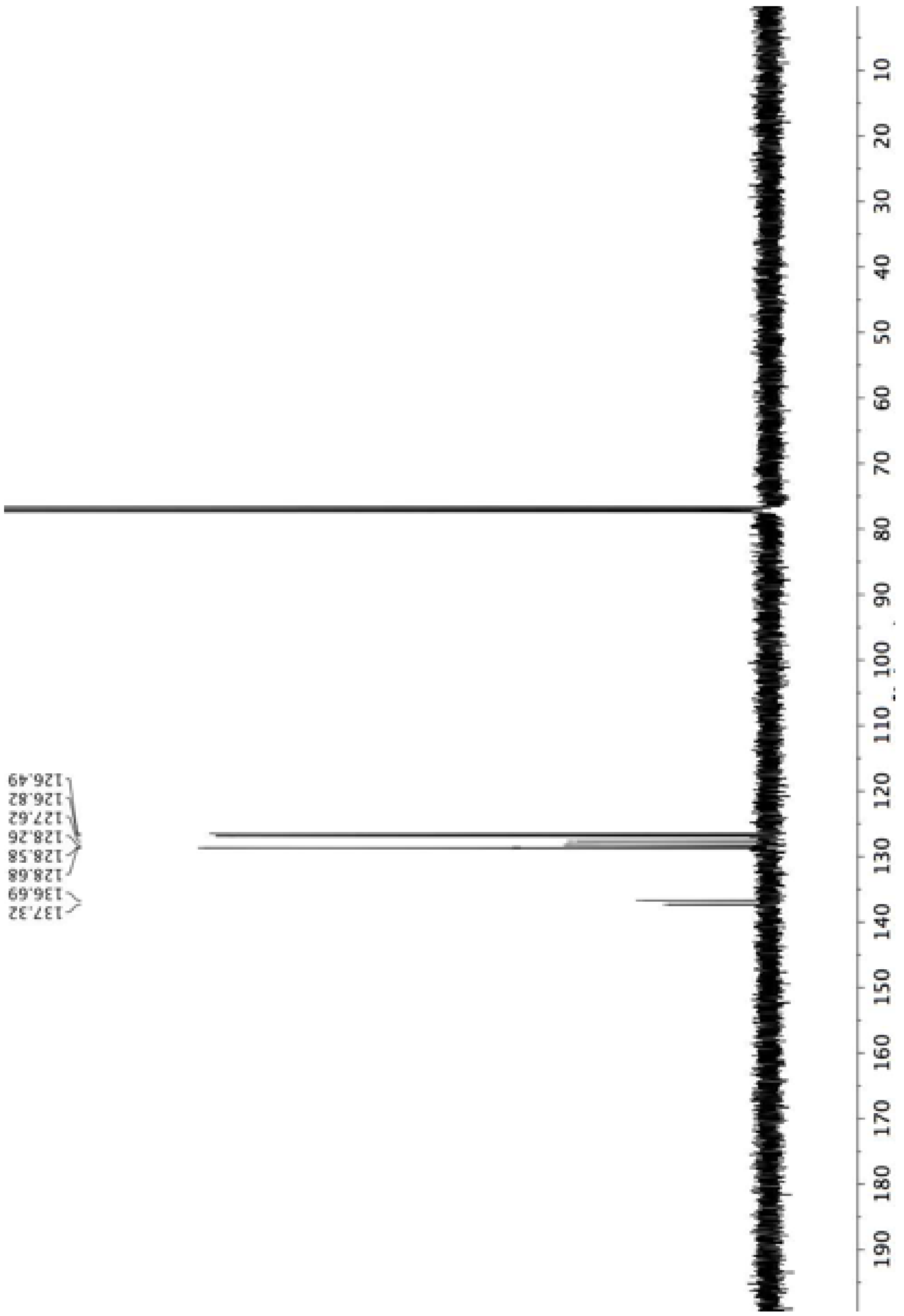
126.49
126.82
127.62
128.26
128.58
128.68
136.69
137.32
10 20 30 40 50 60 70 80 90 100 110 120 130 140 150 160 170 180 190

2,2'-(1,4-phenylene)bis(1-phenylethane-1,2-diol) (**3a**):

To a solution of diene **2a** (1.41 g, 5.0 mmol, 100 mol%) in acetone (38 mL), chloroform (19 mL), and water (18 mL) was added NMO in water (w/w 50%) (2.34 g, 10.0 mmol, 200 mol%) and OsO_4 (1M in *t*BuOH, 0.1 mL, 2 mol%).. The mixture was allowed to stir 40 hours. Toluene (30 mL) was added, and concentrated under vacuum. Provided solid was subjected to flash column chromatography (SiO_2; hexanes:ethyl acetate = 70:30 to 50:50) to furnish the title compound **3a** (1.57 g, 3.5 mmol) in 69% yield as a white solid.

TLC (SiO_2): R_f = 0.58 (ethyl acetate : MeOH = 95:5).
^{1}H NMR: (400 MHz, d_6-DMSO): δ = 7.67–7.38 (m, 14H), 5.24–4.98 (m, 6H), 4.71–4.67 (m, 2H) ppm.
^{13}C NMR: (100 MHz, d_6-DMSO, 3 diastereomers): δ = 152.5, 152.3, 152.3, 152.2, 152.2, 151.3, 151.1, 150.7, 138.0, 137.9, 137.9, 137.8, 137.8, 137.8, 137.7, 137.6, 137.3, 137.1, 137.1, 137.0, 136.9, 89.4, 89.4, 89.4, 89.4, 89.4, 88.3, 88.2, 88.2, 88.1 ppm.
HRMS: (ESI) Calculated for $C_{22}H_{22}O_4$ [M+Na $^+$] = 373.1410, Found 373.1414.
FTIR: (neat): 2365, 1739, 1366, 1217 cm^{-1}.

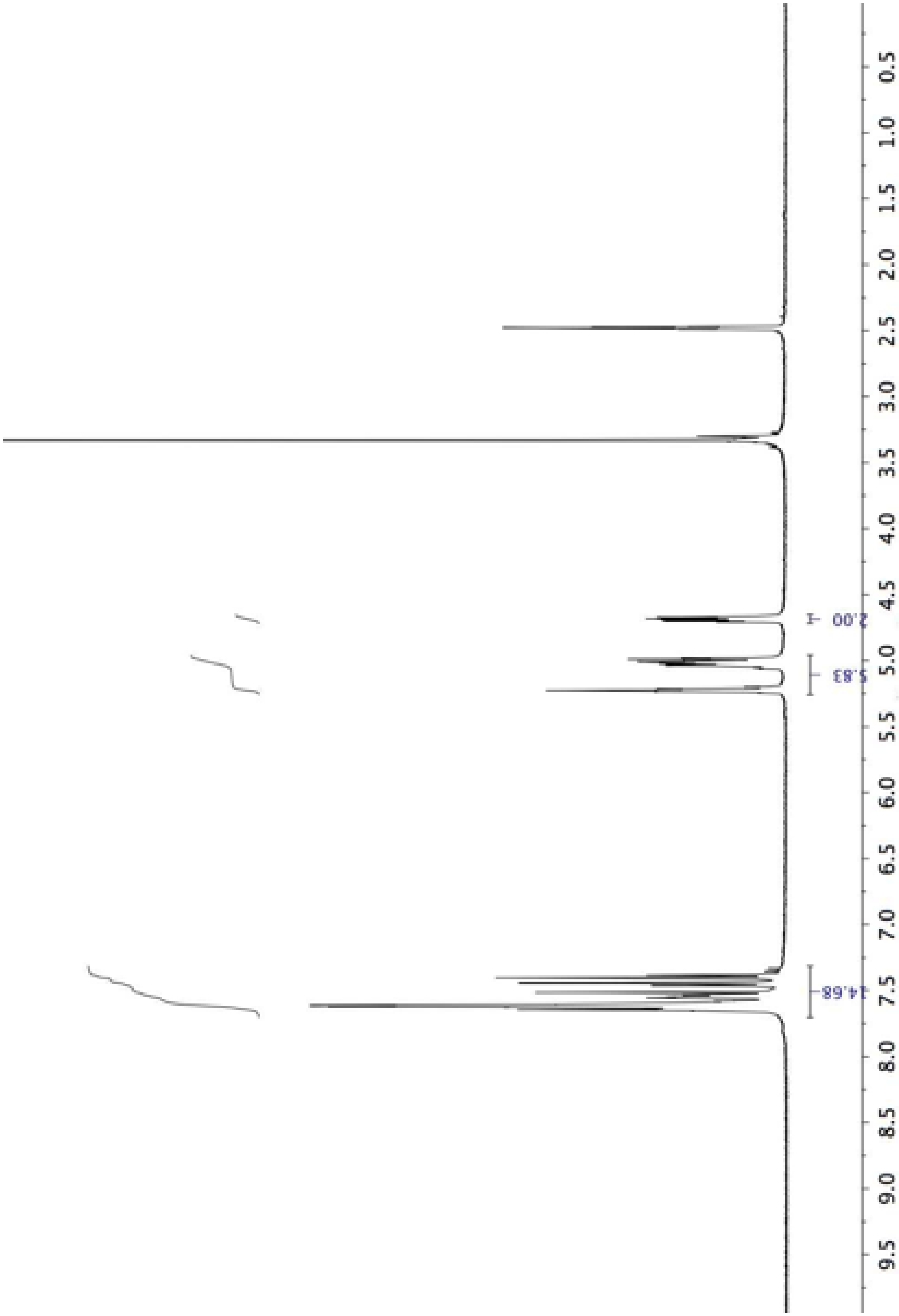

0.5
1.0
1.5
2.0
2.5
3.0
3.5
4.0
4.5
5.0
5.5
6.0
6.5
7.0
7.5
8.0
8.5
9.0
9.5
2.00
5.83
14.68

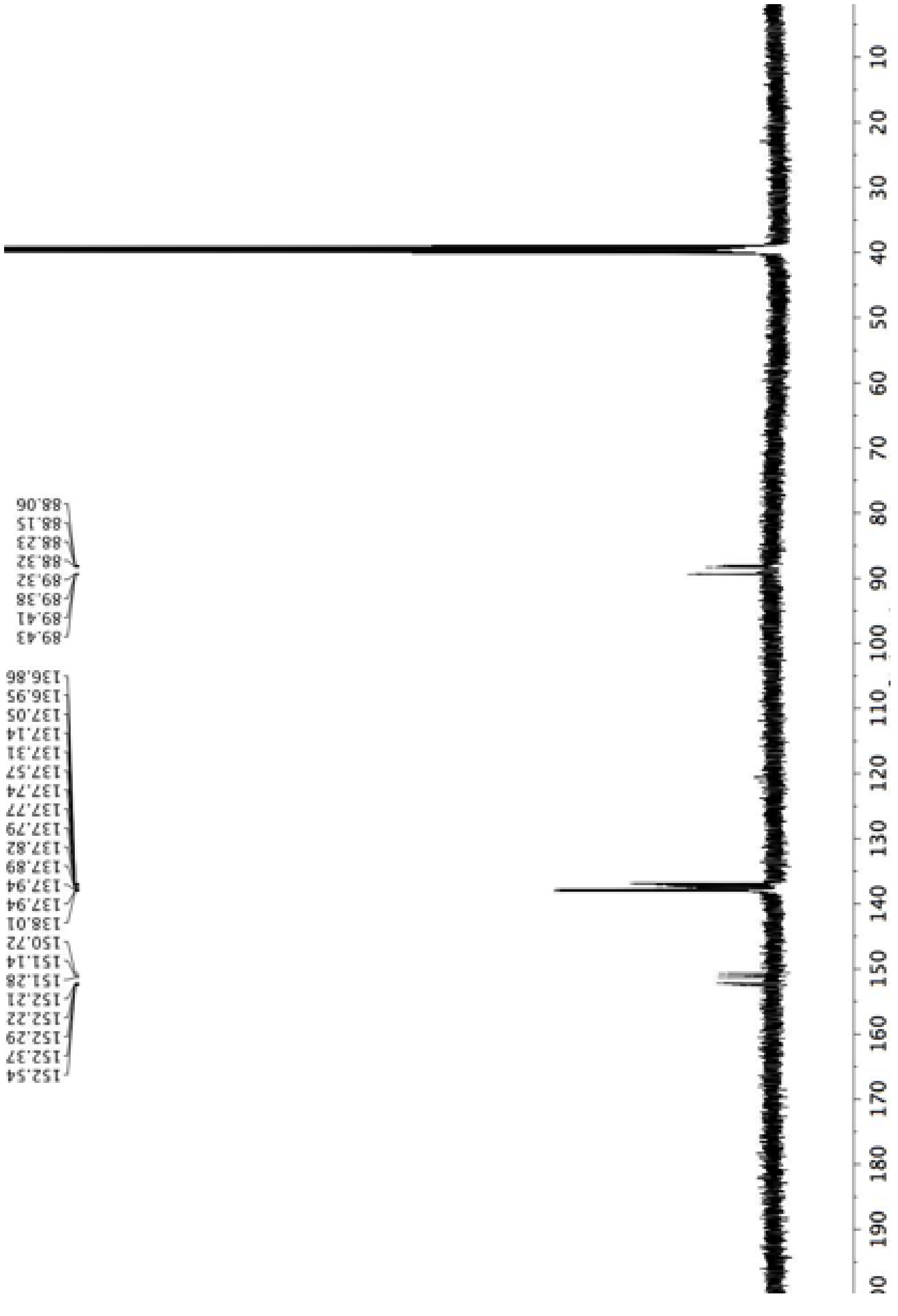
88.06
88.15
88.23
88.32
89.32
89.38
89.41
89.43
136.86
136.95
137.05
137.14
137.31
137.57
137.74
137.77
137.79
137.82
137.89
137.94
137.94
138.01
150.72
151.14
151.28
152.21
152.22
152.29
152.37
152.54
10
20
30
40
50
60
70
80
90
100
110
120
130
140
150
160
170
180
190

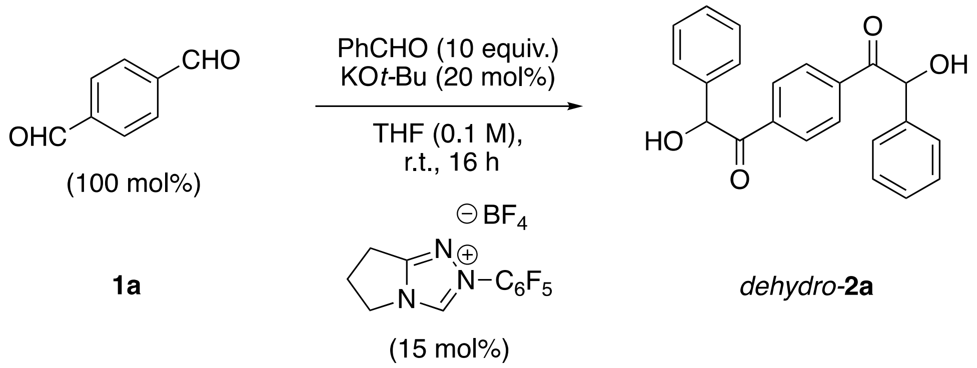

1,1'-(1,4-phenylene)bis(2-hydroxy-2-phenylethan-1-one) (and other tautomers) (*dehydro*-**2a**):

To a solution of terephthalaldehyde (67 mg, 0.5 mmol, 100 mol%), benzaldehyde (0.49 mL, 5.0 mmol, 1000 mol%), and 6,7-dihydro-2-pentafluorophenyl-5H-pyrrolo[2,1,c]-1,2,4-triazolium tetrafluoroborate (27 mg, 0.075 mmol, 15 mol%) in anhydrous THF (5 mL) at ambient temperature was added KOt-Bu (11 mg, 0.1 mmol, 20 mol%). The mixture was allowed to stir for 16 hours. The solution was quenched with water (5 mL) and extracted with EtOAc (3 x 5 mL). The combined organic layers were dried (Na_2SO_4) and volatiles were removed under vacuum. The residue which was subjected to flash column chromatography (SiO_2; hexanes:ethyl acetate = 7:3-1:1) to furnish the title compound *dehydro*-**2a** (108 mg, 0.31 mmol) in 62% yield as an off-white solid.

TLC (SiO_2): R_f = 0.14 (hexanes : ethyl acetate = 70:30).

^{1}H NMR: (500 MHz, $CDCl_3$, mixture of isomers): δ = 7.81 (d, *J* = 8Hz, 4H), 7.52-7.42 (m, 2H), 7.40-7.32 (m, 4H), 7.25-7.21 (m, 4H), 5.92 (d, *J* = 4.6 Hz, 2/3 H), 5.87 (d, *J* = 4.8 Hz, 2/3 H), 5.83 (d, *J* = 6.7 Hz, 2/3 H) ppm.

^{13}C NMR: (125 MHz, $CDCl_3$, mixture of isomers): δ = 198.5, 198.3, 198.2, 198.1, 144.6, 139.23, 139.19, 138.6, 134.3, 134.1, 133.4, 133.2, 133.0, 130.1, 129.9, 129.2, 129.1, 128.8, 128.72, 128.68, 128.52, 128.51, 128.4, 128.0, 127.9, 127.7, 76.3, 75.6, 75.44, 75.38 ppm.

HRMS: (ESI$^+$) Calculated for $C_{22}H_{18}O_4$ [M+Na$^+$] = 369.1097 , Found 369.1093.

FTIR: (neat): 3390, 2340, 1673, 1256, 1088, 976, 811, 680 cm^{-1}.

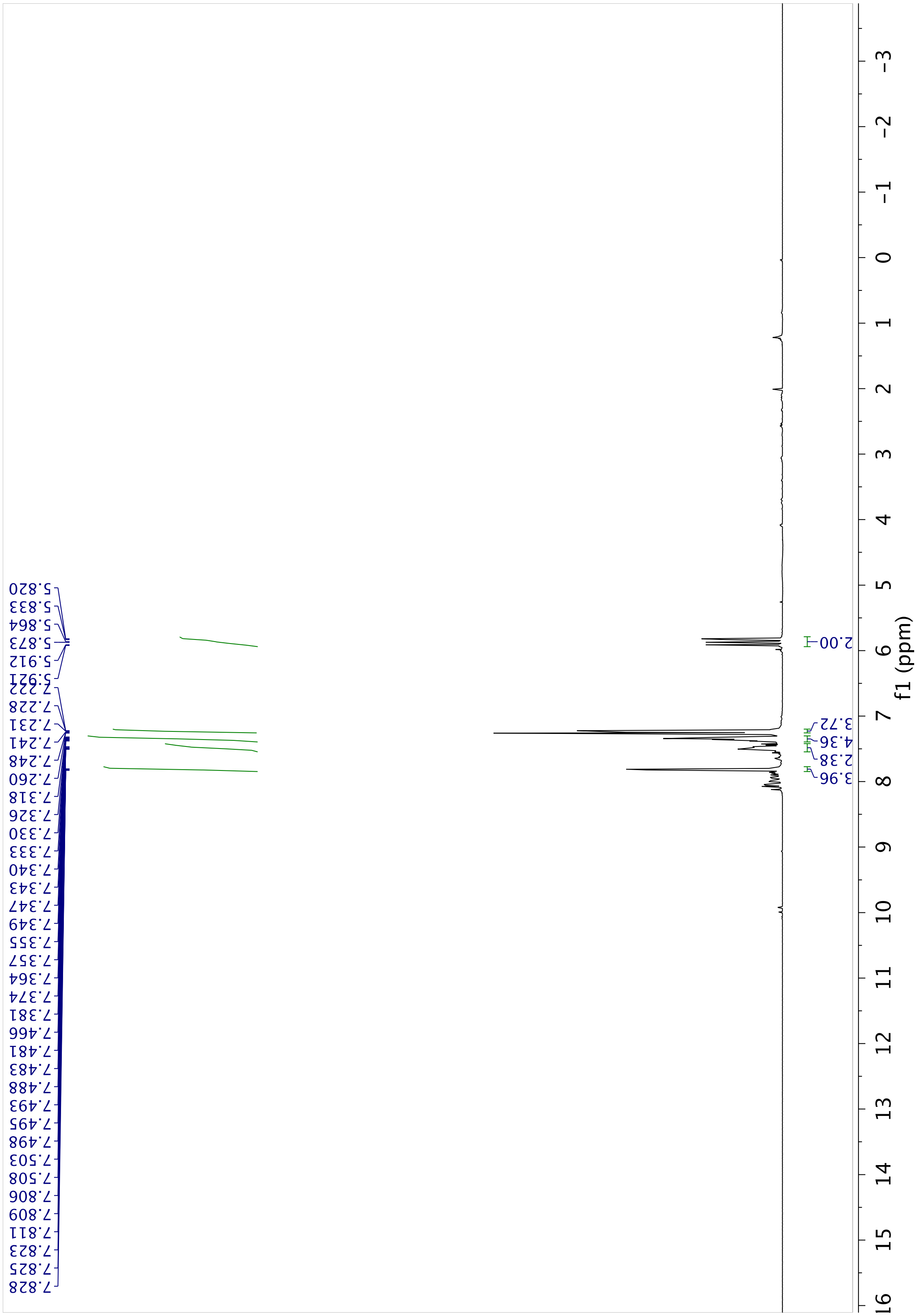

7.828
7.825
7.823
7.811
7.809
7.806
7.508
7.503
7.498
7.495
7.493
7.488
7.483
7.481
7.466
7.381
7.374
7.364
7.357
7.355
7.349
7.347
7.343
7.340
7.333
7.330
7.326
7.318
7.260
7.248
7.241
7.231
7.228
7.222
5.921
5.912
5.873
5.864
5.833
5.820
3.96
2.38
4.36
3.72
2.00
16 15 14 13 12 11 10 9 8 7 6 5 4 3 2 1 0 −1 −2 −3
f1 (ppm)

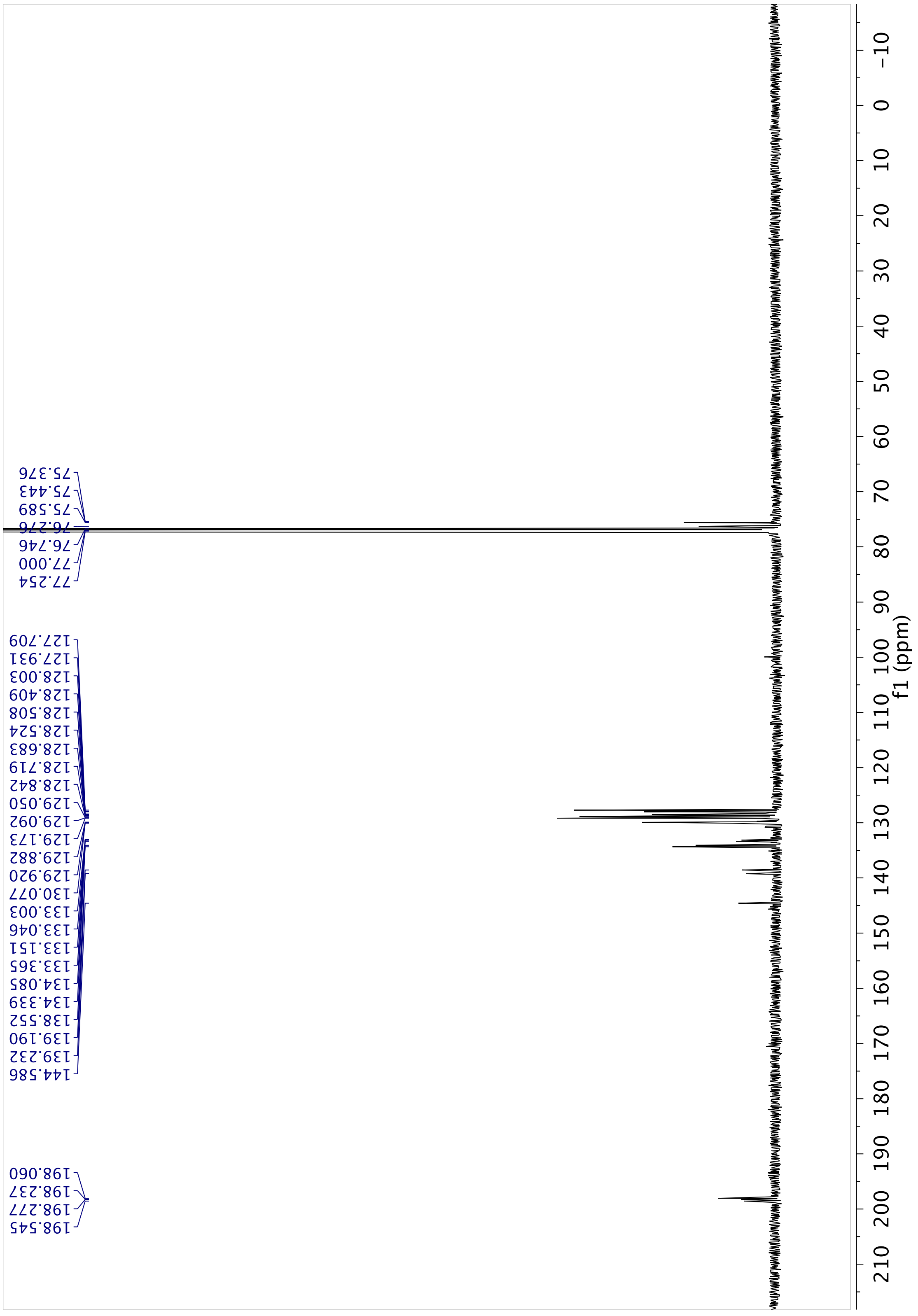
75.376
75.443
75.589
76.276
76.746
77.000
77.254
127.709
127.931
128.003
128.409
128.508
128.524
128.683
128.719
128.842
129.050
129.092
129.173
129.882
129.920
130.077
133.003
133.046
133.151
133.365
134.085
134.339
138.552
139.190
139.232
144.586
198.060
198.237
198.277
198.545
-10
0
10
20
30
40
50
60
70
80
90
100
110
120
130
140
150
160
170
180
190
200
210
f1 (ppm)

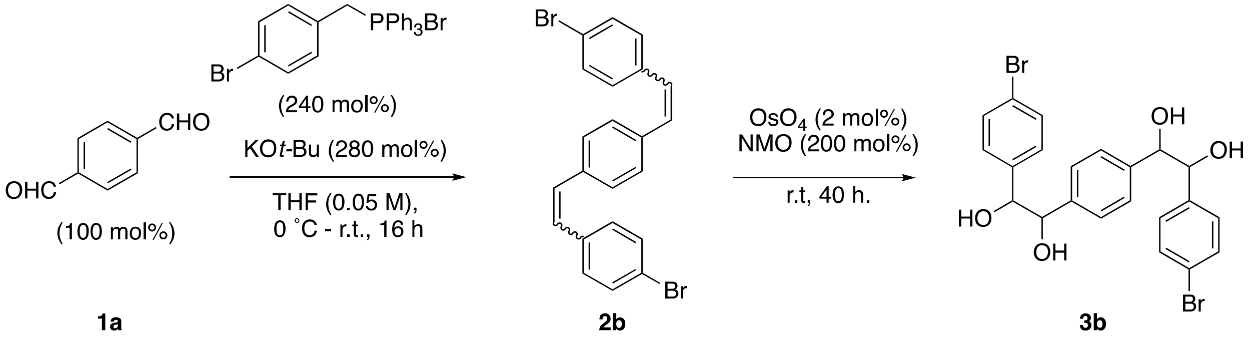

1,4-bis(4-bromostyryl)benzene (**2b**):

To a solution of KO*t*-Bu (1.12 g, 28.0 mmol, 280 mol%) in anhydrous THF (100 mL) cooled to 0 ˚C was added Wittig reagent (12.3 g, 24.0 mmol, 240 mol%). The mixture was allowed to stir at the same temperature for 30 min followed by the addition of terephthalaldehyde (1.34 g, 10.0 mmol, 100 mol%) in THF (100 mL) dropwise over 30 min. The reaction was then warmed to room temperature and allowed to stir for 16 hours. The solution was concentrated under vacuum followed by addition of water (50 mL). The aqueous layer was then extracted with Et_2O (3 x 25 mL) and the combined organic layers were washed with brine (50 mL), dried (Na_2SO_4) and filtered. Evaporation under reduced pressure provided an oily residue which was subjected to flash column chromatography (SiO_2; hexanes:ethyl acetate = 99:1) to furnish the title compound **2b** (3.83 g, 8.7 mmol) in 87% yield as a white solid.

TLC (SiO_2): R_f = 0.78 (hexanes : ethyl acetate = 90:10).
^{1}H NMR: (400 MHz, $CDCl_3$, 2 diastereomers): δ = 7.47 (d, *J* = 8.2 Hz, 2H), 7.38–7.35 (m, 5H), 7.26–7.21 (m, 3H), 7.14(d, *J* = 8.1 Hz, 2H), 7.03 (d, *J* = 3.0 Hz, 2H), 6.62–6.50 (M, 2H) ppm.
^{13}C NMR: (100 MHz, $CDCl_3$, 2 diastereomers): δ = 136.4, 136.2, 136.1, 136.0, 131.8, 131.4, 130.5, 130.5, 129.3, 129.1, 128.9, 127.9, 127.5, 126.5, 121.4, 121.0 ppm.
HRMS: (CI^+) Calculated for $C_{22}H_{16}Br_2$ [M^+] = 439.9598, Found 439.9600.
FTIR: (neat): 2918, 1738, 1366, 1228, 836 cm^{-1}.

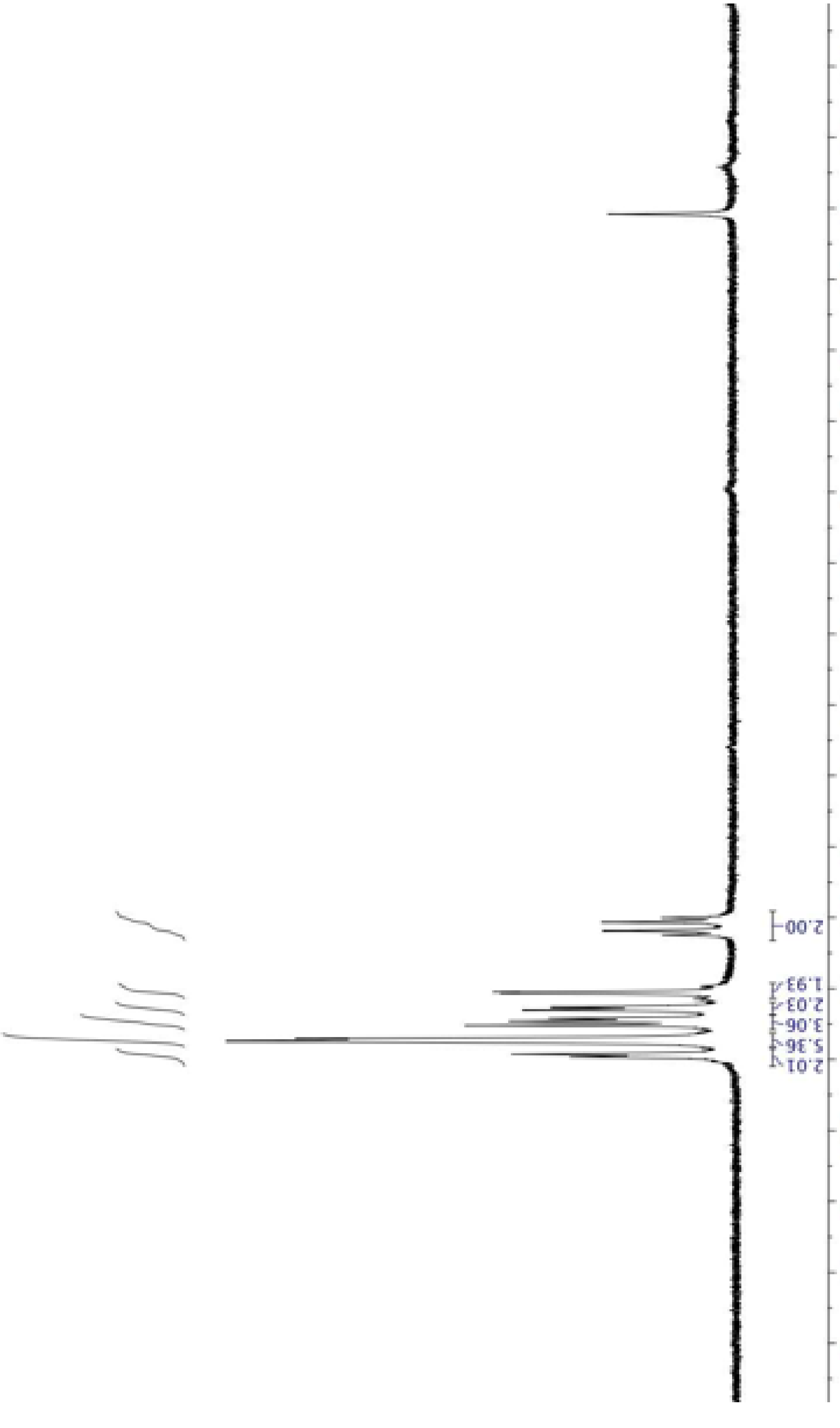

2.00
1.93
2.03
3.06
5.36
2.01
0.5
1.0
1.5
2.0
2.5
3.0
3.5
4.0
4.5
5.0
5.5
6.0
6.5
7.0
7.5
8.0
8.5
9.0
9.5

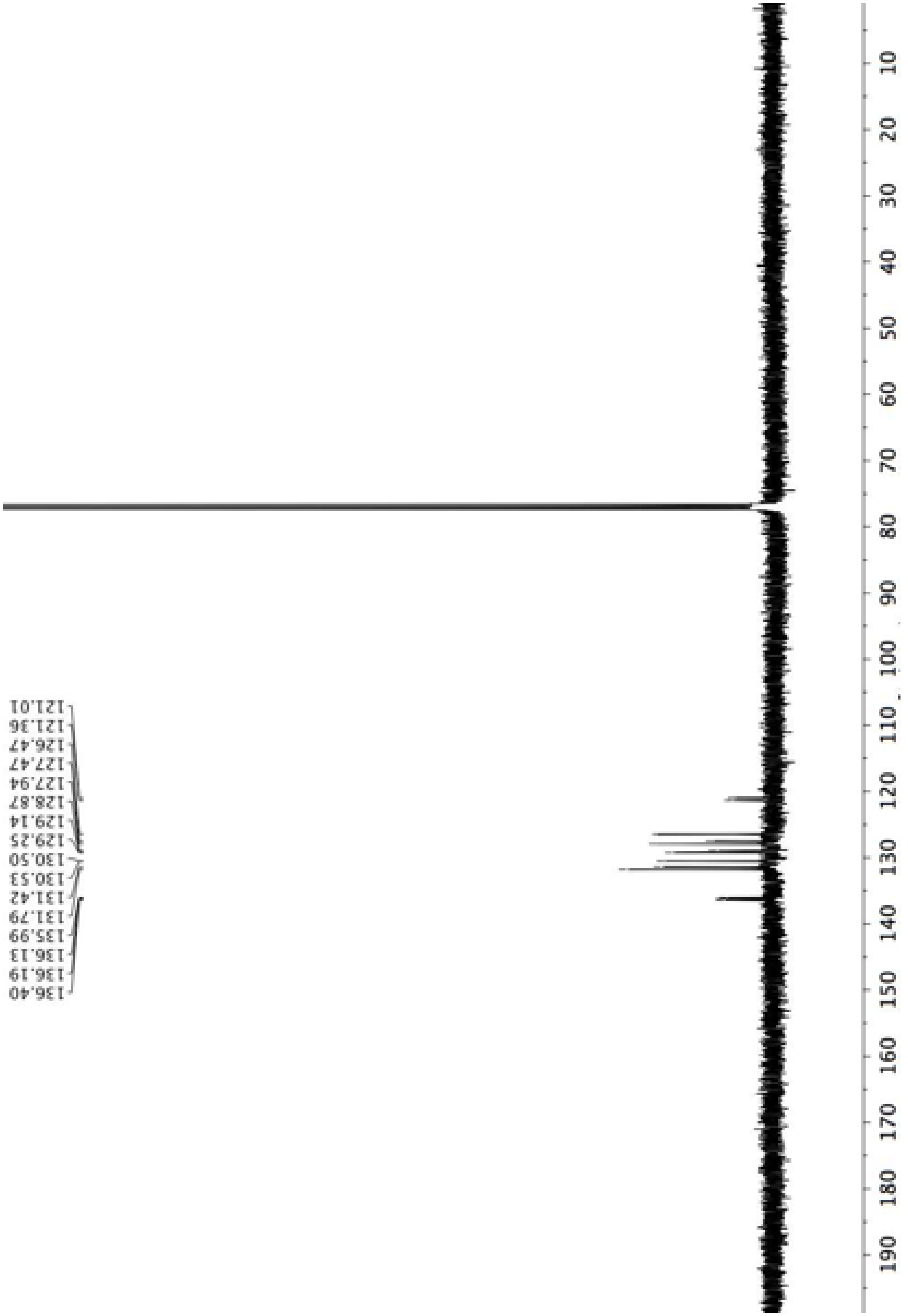
121.01
121.36
126.47
127.47
127.94
128.87
129.14
129.25
130.50
130.53
131.42
131.79
135.99
136.13
136.19
136.40
10
20
30
40
50
60
70
80
90
100
110
120
130
140
150
160
170
180
190

2,2'-(1,4-phenylene)bis(1-(4-bromophenyl)ethane-1,2-diol) (**3b**):

To a solution of diene **2b** (2.2 g, 5.0 mmol, 100 mol%) in acetone (38 mL), chloroform (19 mL), and water (17 mL) was added NMO in water (w/w 50%) (2.34 g, 10.0 mmol, 200 mol%) and OsO_4 (1M in *t*BuOH, 0.1 mL, 2 mol%).. The mixture was allowed to stir 40 hours. Toluene (30 mL) was added, and concentrated under vacuum. Provided solid was subjected to flash column chromatography (SiO_2; hexanes:ethyl acetate = 30:70 to 50:50) to furnish the title compound **3b** (2.28 g, 4.5 mmol) in 90% yield as a white solid.

TLC (SiO_2): R_f = 0.58 (ethyl acetate : MeOH = 95:5).
^{1}H NMR: (400 MHz, d_6-DMSO, 3 diastereomers): δ = 7.39–7.33 (m, 4H), 7.07–6.93 (m, 8H), 5.43–5.19 (m, 4H), 4.56–4.50 (m, 4H) ppm.
^{13}C NMR: (100 MHz, d_6-DMSO, 3 diastereomers): δ = 142.7, 142.5, 142.3, 141.5, 141.4, 140.7, 130.5, 130.5, 130.4, 130.4, 130.1, 130.0, 129.9, 126.7, 126.6, 126.6, 120.1, 120.0, 77.7, 77.6, 77.3, 77.3, 76.9, 76.9 ppm.
HRMS: (ESI) Calculated for $C_{22}H_{20}Br_2O_4$ [M+Na$^+$] = 530.9602, Found 530.9609.
FTIR: (neat): 2918, 2849, 1738, 1366, 1217 cm^{-1}.

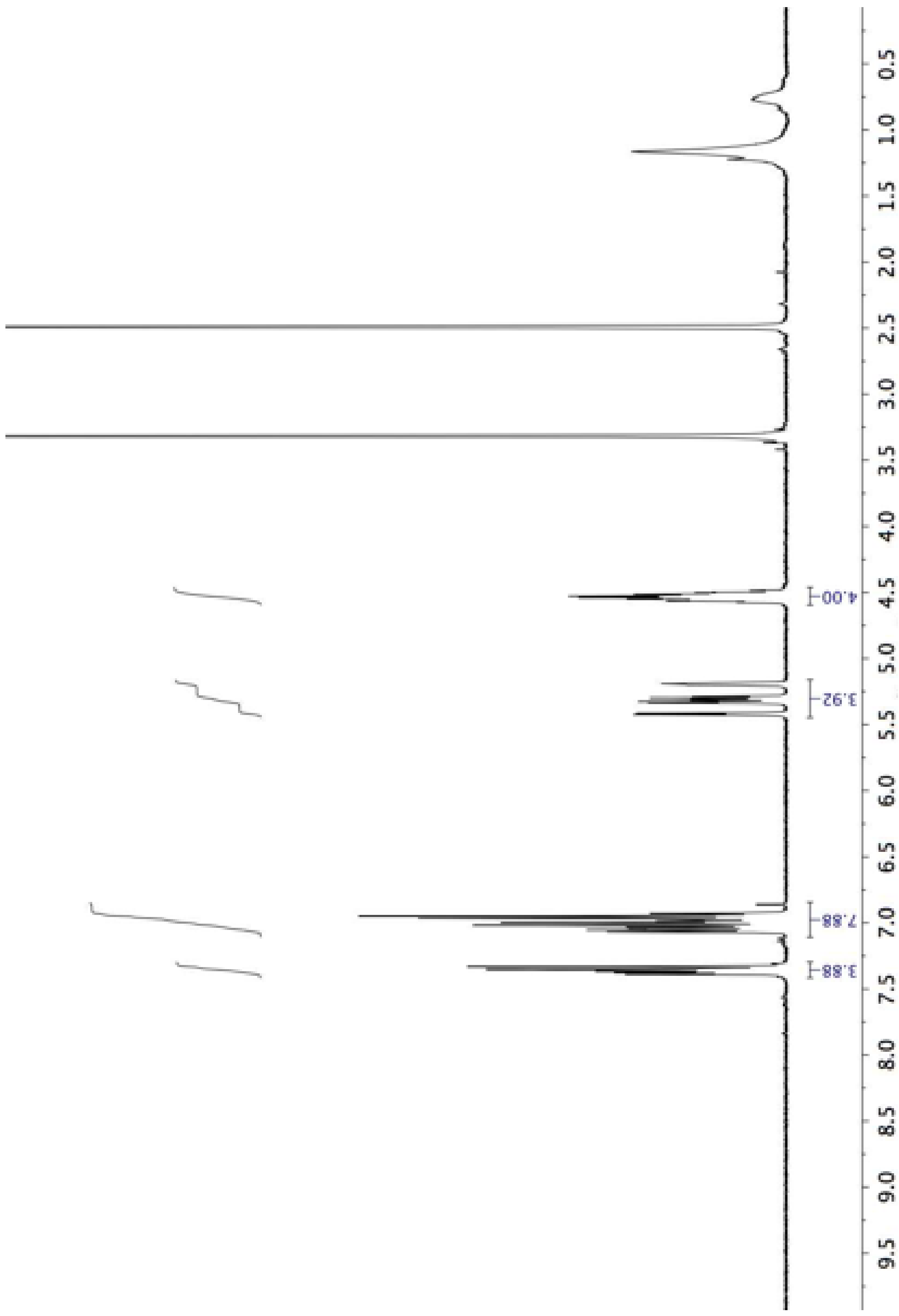
0.5
1.0
1.5
2.0
2.5
3.0
3.5
4.0
4.5
5.0
5.5
6.0
6.5
7.0
7.5
8.0
8.5
9.0
9.5
4.00
3.92
7.88
3.88

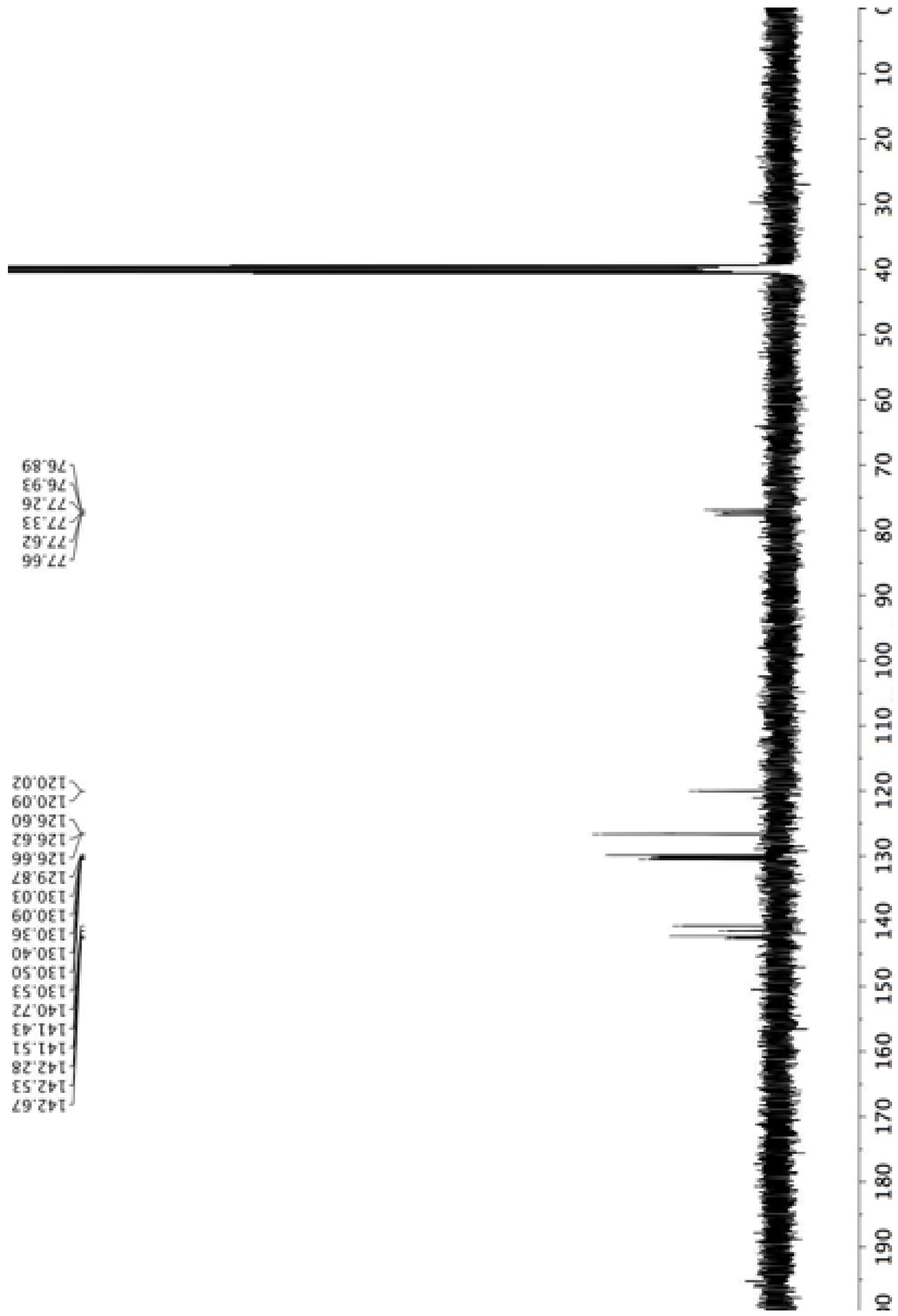
76.89
76.93
77.26
77.33
77.62
77.66
120.02
120.09
126.60
126.62
126.66
129.87
130.03
130.09
130.36
130.40
130.50
130.53
140.72
141.43
141.51
142.28
142.53
142.67
0
10
20
30
40
50
60
70
80
90
100
110
120
130
140
150
160
170
180
190

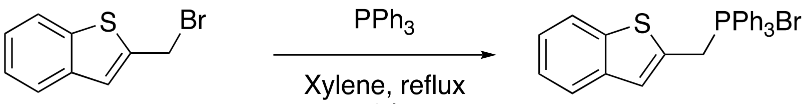

S1 S2

(benzo[*b*]thiophen-2-ylmethyl)bromotriphenylphosphane (S2):

Bromide **S1** was synthesized according to known procedures[i] and their characterization data[ii] were identical in all respects.

To 2-bromomethylbenzothiophene (**S1**) (3.31 g, 15.0 mmol, 100 mol%) in xylene (30 mL) was added triphenylphosphine (3.93 g, 15.0 mmol, 100 mol%). The mixture was stirred under reflux for 16 h. Then, the reaction mixture was allowed to cool down to room temperature. The precipitation was filtrated, and the residue was washed with toluene and hexanes to furnish the title compound **S2** (6.90 g, 14.1 mmol) in 94% yield as a white solid.

TLC (SiO_2): R_f = 0.20 (MeOH = 100).

^{1}H NMR: (400 MHz, $CDCl_3$): δ = 7.85–7.76 (m, 9H), 7.65–7.58 (m, 9H), 7.29–7.23 (m, 2H), 5.96 (d, *J* = 14.1 Hz, 2H) ppm.

^{13}C NMR: (100 MHz, $CDCl_3$): δ = 139.9, 135.1, 135.0, 134.3, 134.2, 130.2, 130.0, 124.7, 124.5, 123.9, 121.8, 118.0, 117.2 ppm.

^{31}P NMR: (160 MHz, $CDCl_3$): *δ* 21.3 ppm (85% H_3PO_4 was used as internal standard 0 ppm).

HRMS: (ESI) Calculated for $C_{27}H_{22}PS$ [M$^+$] =409.11740, Found 409.11860.

FTIR: (neat): 3032, 3010, 2835, 2774, 1738, 1438, 1225, 1109, 710, 686 cm^{-1}.

MP : 262-263 °C

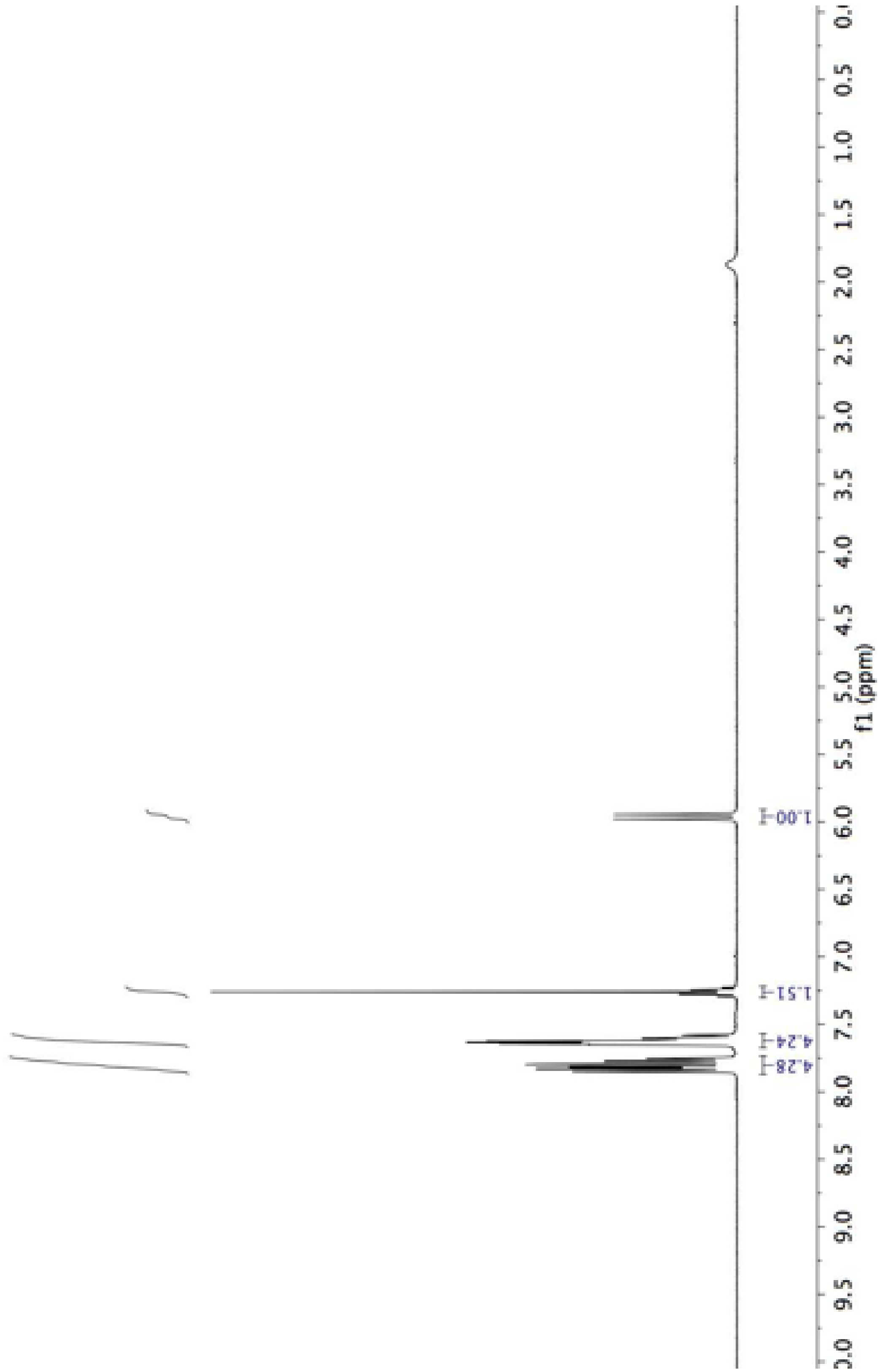
f1 (ppm)
1.00
1.51
4.24
4.28

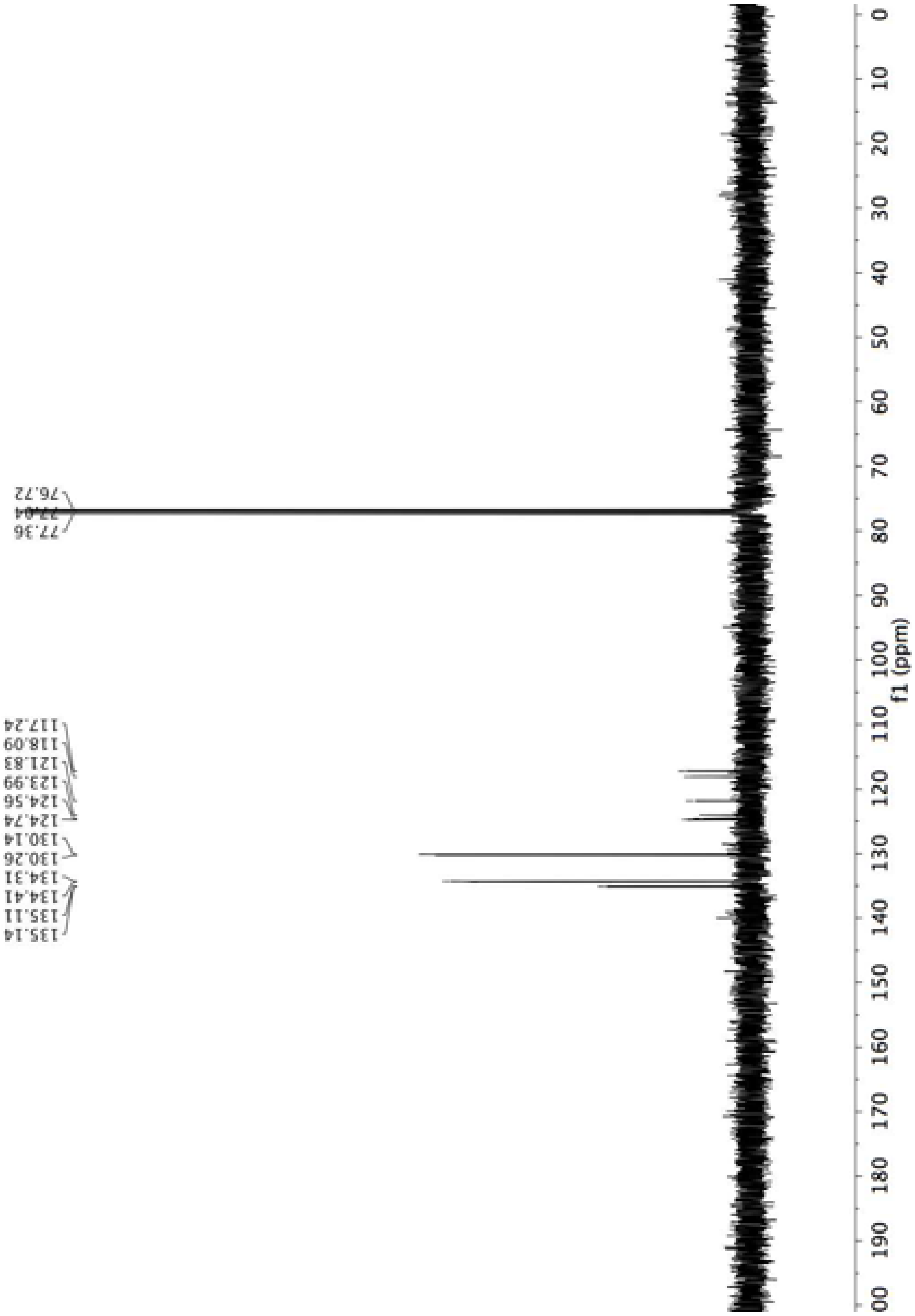
76.72
77.04
77.36
117.24
118.09
121.83
123.99
124.56
124.74
130.14
130.26
134.31
134.41
135.11
135.14
f1 (ppm)

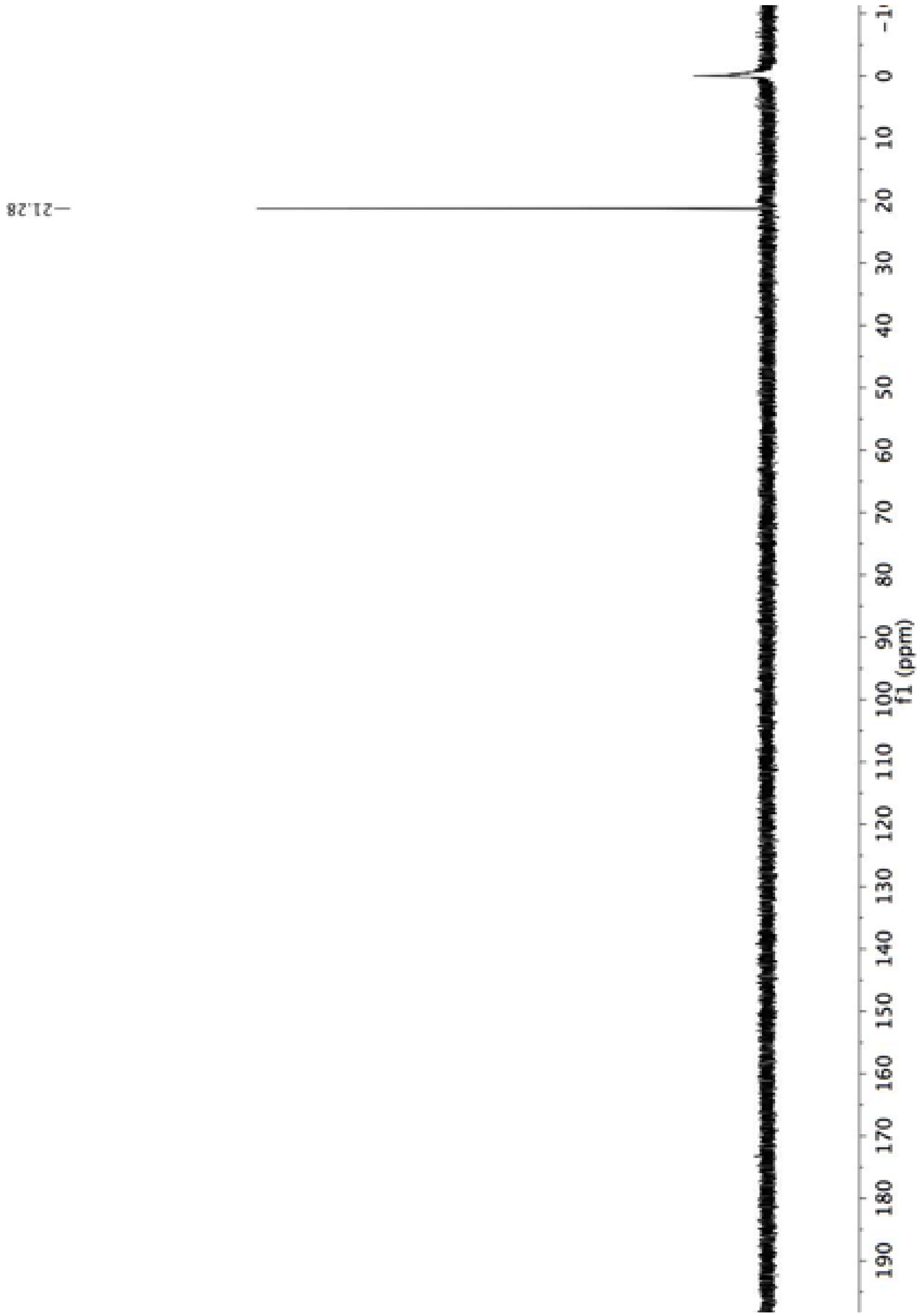
21.28
f1 (ppm)
0 10 20 30 40 50 60 70 80 90 100 110 120 130 140 150 160 170 180 190

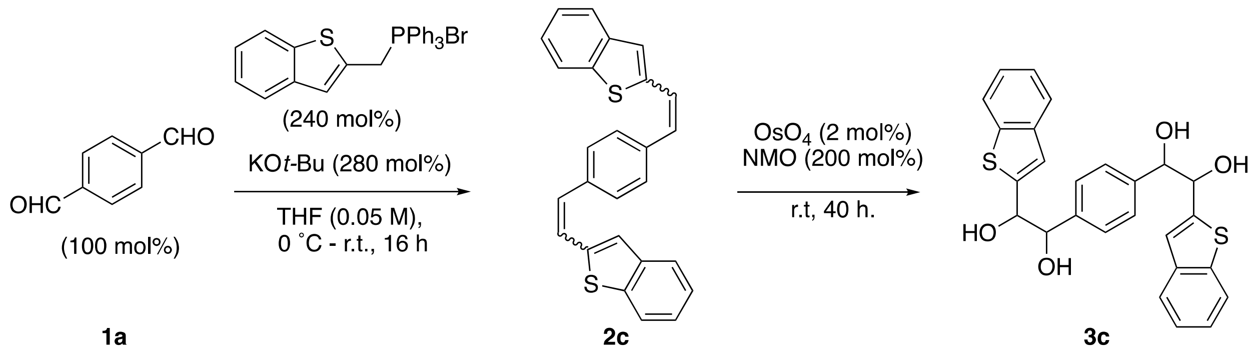

1a **2c** **3c**

1,4-bis(2-(benzo[*b*]thiophen-2-yl)vinyl)benzene (**2c**):

To a solution of KO*t*-Bu (1.12 g, 28.0 mmol, 280 mol%) in anhydrous THF (100 mL) cooled to 0 ˚C was added Wittig reagent (11.7 g, 24.0 mmol, 240 mol%). The mixture was allowed to stir at the same temperature for 30 min followed by the addition of terephthalaldehyde (1.34 g, 10.0 mmol, 100 mol%) in THF (100 mL) dropwise over 30 min. The reaction was then warmed to room temperature and allowed to stir for 16 hours. The solution was concentrated under vacuum followed by addition of water (50 mL). The aqueous layer was then extracted with Et_2O (3 x 25 mL) and the combined organic layers were washed with brine (50 mL), dried (Na_2SO_4) and filtered. Evaporation under reduced pressure provided an oily residue which was subjected to flash column chromatography (SiO_2; hexanes:DCM = 90:10) to furnish the title compound **2c** (2.84 g, 7.2 mmol, *Z,Z*:*E,Z* = 1:1) in 72% yield as a yellow solid.

TLC (SiO_2): R_f = 0.78 (DCM:hexanes= 1:8).

^{1}H NMR: (400 MHz, $CDCl_3$, *Z*,*Z* isomer): δ 7.59–7.56 (m, 4H), 7.35 (s, 4H), 7.28–7.22 (m, 4H), 7.25 (s, 2H), 6.74 (d, *J* = 12.0 Hz, 2H), 6.66 (d, *J* = 12.0 Hz, 2H) ppm.

^{1}H NMR: (400 MHz, $CDCl_3$, *E*,*Z* isomer): δ 7.79–7.77 (m, 1H), 7.72–7.70 (m, 1H), 7.67–7.64 (m, 2H), 7.51 (d, *J* = 8.6 Hz, 2H), 7.42 (d, *J* = 16.0 Hz, 2H), 7.37 (d, *J* = 16.0 Hz, 1H), 7.33–7.30 (m, 2H), 7.29–7.26 (m, 2H), 7.26 (s, 1H), 7.25 (s, 1H), 7.02 (d, *J* = 16.0 Hz, 1H), 6.79 (d, *J* = 11.7 Hz, 1H), 6.70 (d, *J* = 11.7 Hz, 1H) ppm.

^{13}C NMR: (100 MHz, $CDCl_3$, *Z*,*Z* isomer and *E*,*Z* isomer): δ 142.9, 140.2, 140.0, 140.0, 139.9, 139.1, 139.0, 138.9, 136.5, 136.4, 136.1, 133.8, 133.6, 130.9, 130.8, 130.5, 129.5, 129.1, 129.1, 128.7, 128.5, 128.4, 126.6, 125.2, 125.2, 124.8, 124.6, 124.6, 124.5, 124.2, 124.2, 124.1, 123.9, 123.4, 123.3, 122.5, 122.2, 122.0, 122.0 ppm.

HRMS: (CI^+) Calculated for $C_{26}H_{18}S_2$ [M^+] =394.0850, Found 394.0852.

FTIR: (neat) 3053, 3017, 2945, 1625, 1587, 1455, 1147, 1014, 962, 743 cm^{-1}.

MP: >360 °C (color change : yellow to black)

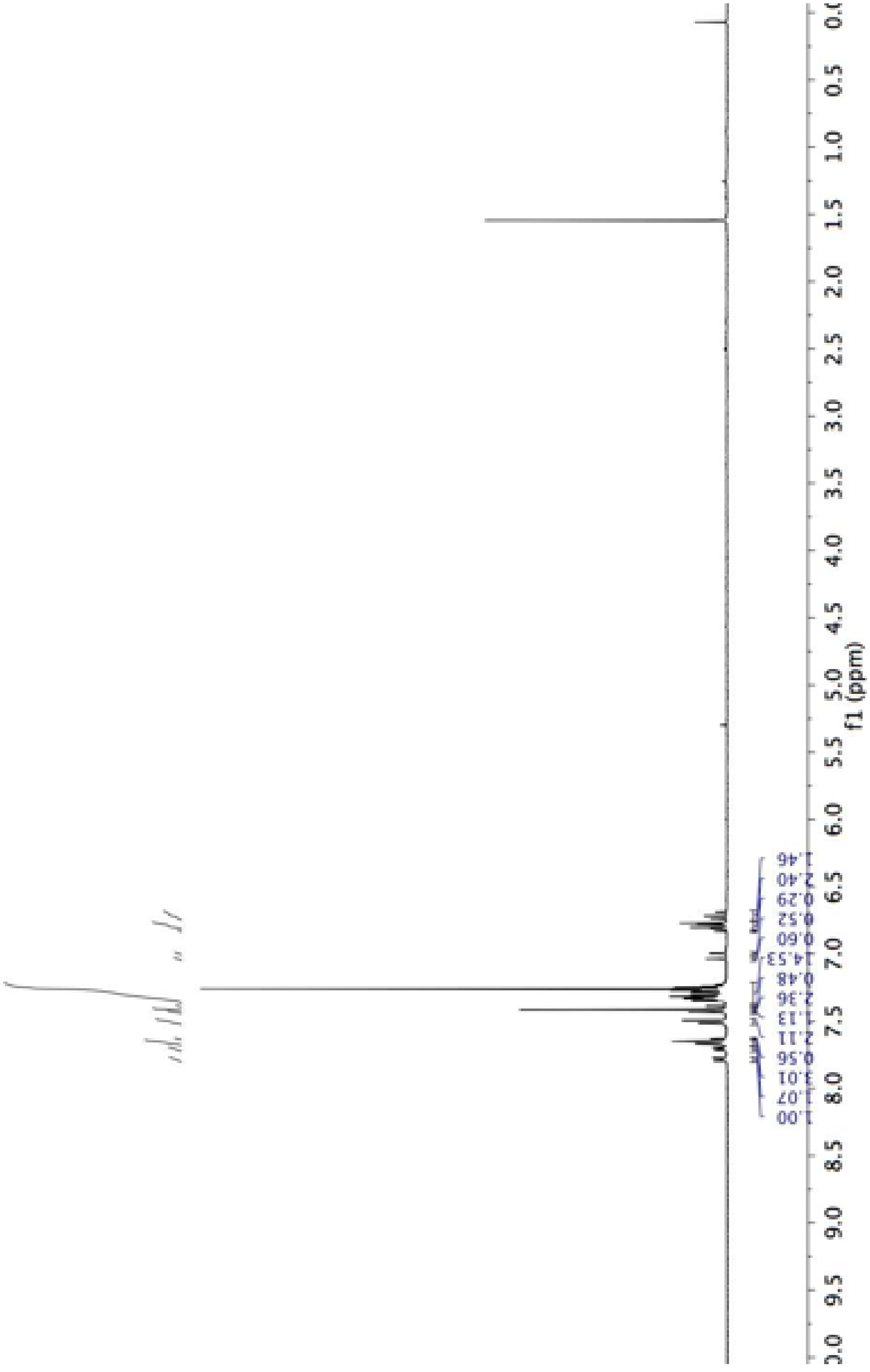
1.46
2.40
0.29
0.52
0.60
14.53
0.48
2.36
1.13
2.11
0.56
3.01
1.07
1.00
f1 (ppm)
0.5
1.0
1.5
2.0
2.5
3.0
3.5
4.0
4.5
5.0
5.5
6.0
6.5
7.0
7.5
8.0
8.5
9.0
9.5

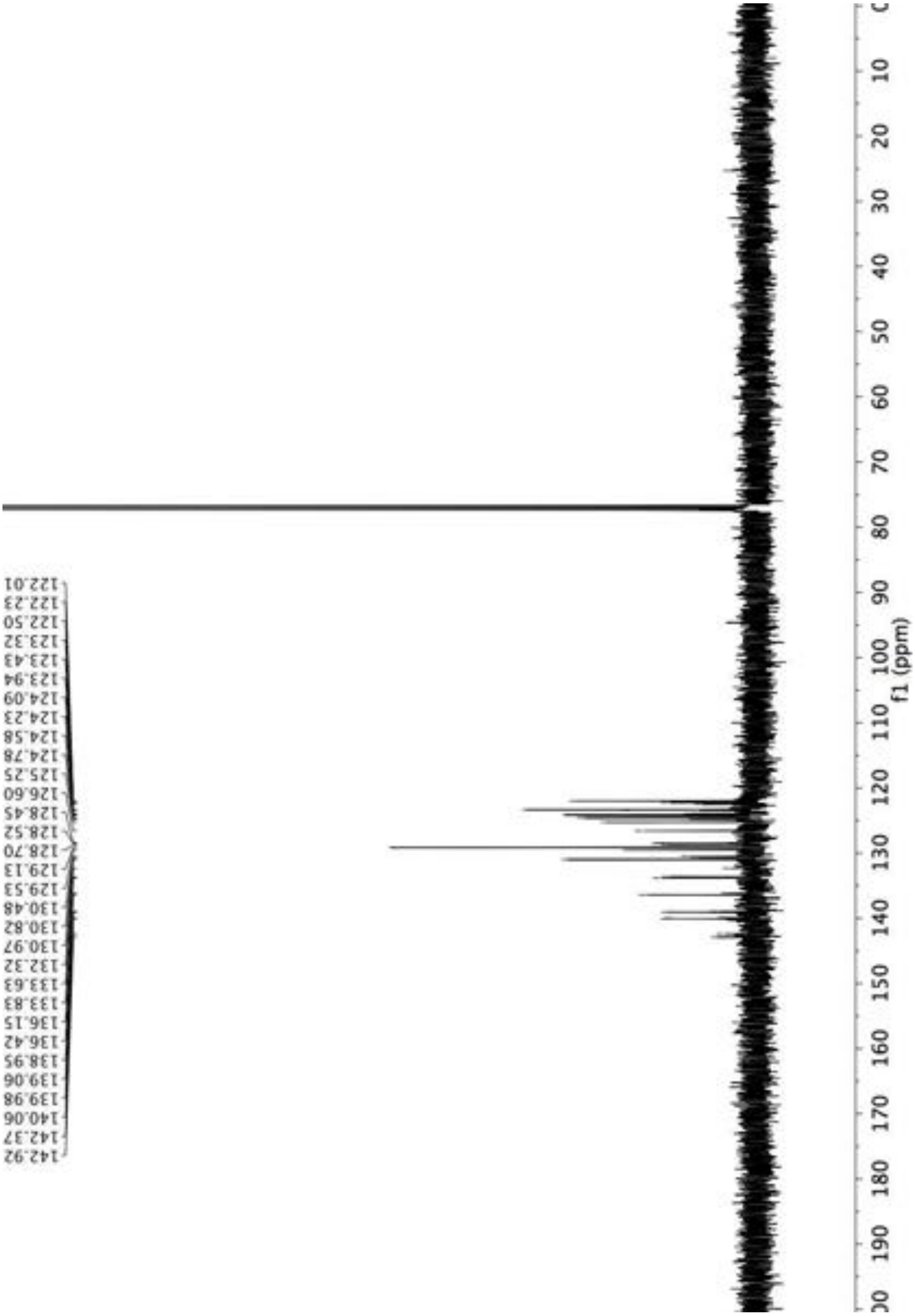
122.01
122.23
122.50
123.32
123.43
123.94
124.09
124.23
124.58
124.78
125.25
126.60
128.45
128.52
128.70
129.13
129.53
130.48
130.82
130.97
132.32
133.63
133.83
136.15
136.42
138.95
139.06
139.98
140.06
142.37
142.92
f1 (ppm)

2,2'-(1,4-phenylene)bis(1-(benzo[*b*]thiophen-2-yl)ethane-1,2-diol) (**3c**):

To a solution of diene **2c** (1.47 g, 5.0 mmol, 100 mol%) in acetone (38 mL), chloroform (19 mL), and water (18 mL) was added NMO in water (w/w 50%) (2.34 g, 10.0 mmol, 200 mol%) and OsO_4 (1M in *t*BuOH, 0.1 mL, 2 mol%). The mixture was allowed to stir 40 hours. Toluene (30 mL) was added, and concentrated under vacuum. Provided solid was subjected to flash column chromatography (SiO_2; hexanes:ethyl acetate = 1:1) to furnish the title compound **3c** (1.55 g, 3.4 mmol) in 67% yield as a white solid.

TLC (SiO_2): R_f = 0.25 (hexanes : ethyl acetate = 1:2).
^{1}H NMR: (400 MHz, DMSO-d6, isomer mixture): δ 7.87–7.82 (m, 2H), 7.72–7.59 (m, 2H), 7.30–6.93 (m, 10H), 5.91–4.61 (m, 2H) ppm.
^{13}C NMR: (100 MHz, DMSO-d6, isomer mixture): δ 148.5, 148.2, 148.2, 147.5, 147.5, 141.4, 140.4, 139.1, 138.8, 126.4, 126.3, 123.9, 123.8, 123.5, 123.1, 122.2, 121.0, 120.9, 120.9, 120.6, 120.6, 76.9, 76.4, 76.3, 76.2, 74.0, 73.8 ppm
HRMS: (ESI) Calculated for $C_{26}H_{22}O_4S_2$ [M+Na$^+$] = 485.08520, Found 485.08570.
FTIR: (neat) 3349, 3057, 2890, 1688, 1456, 1036, 1013, 837, 741, 694 cm^{-1}.

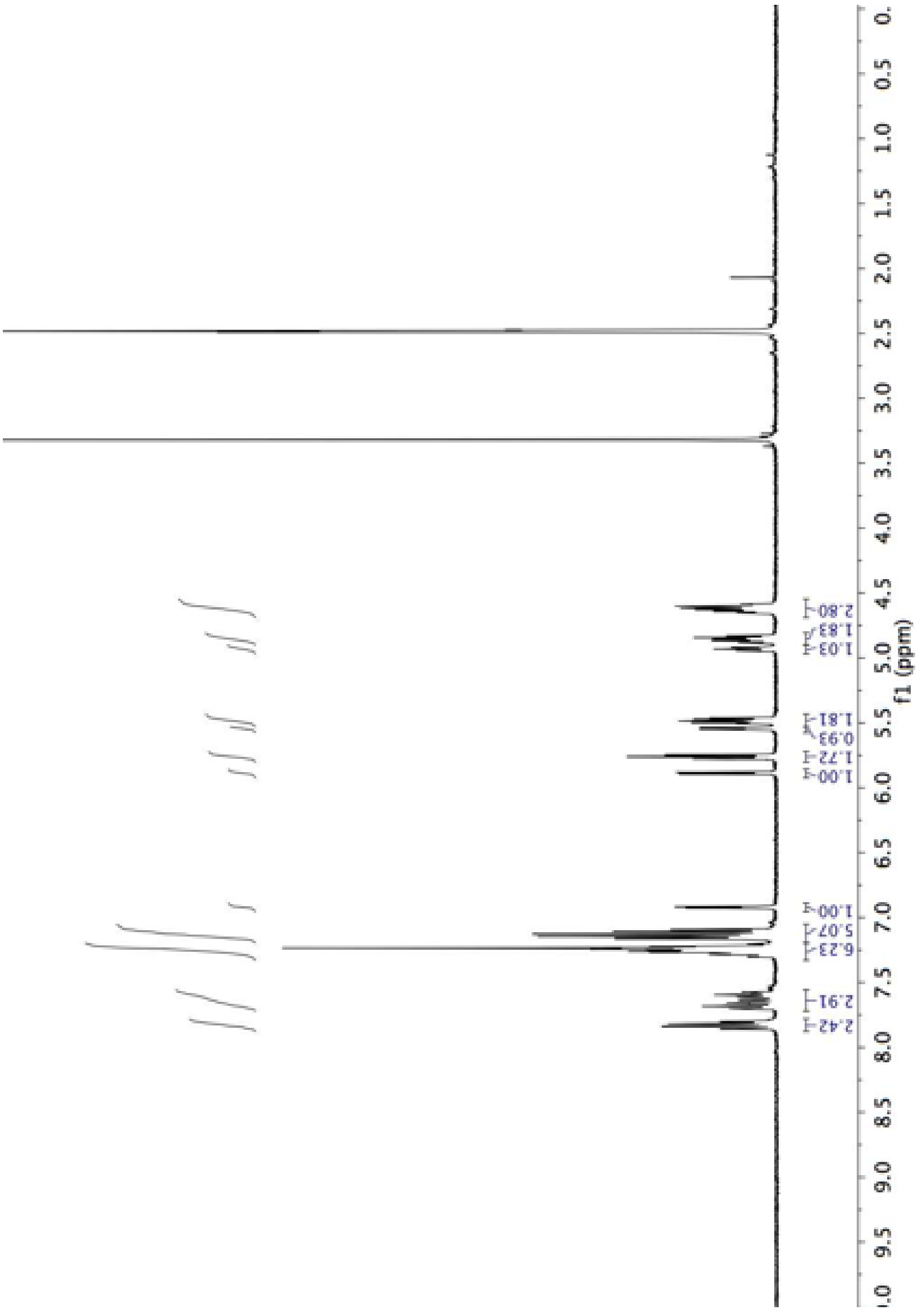

f1 (ppm)
2.80
1.83
1.03
1.81
0.93
1.72
1.00
1.00
5.07
6.23
2.91
2.42

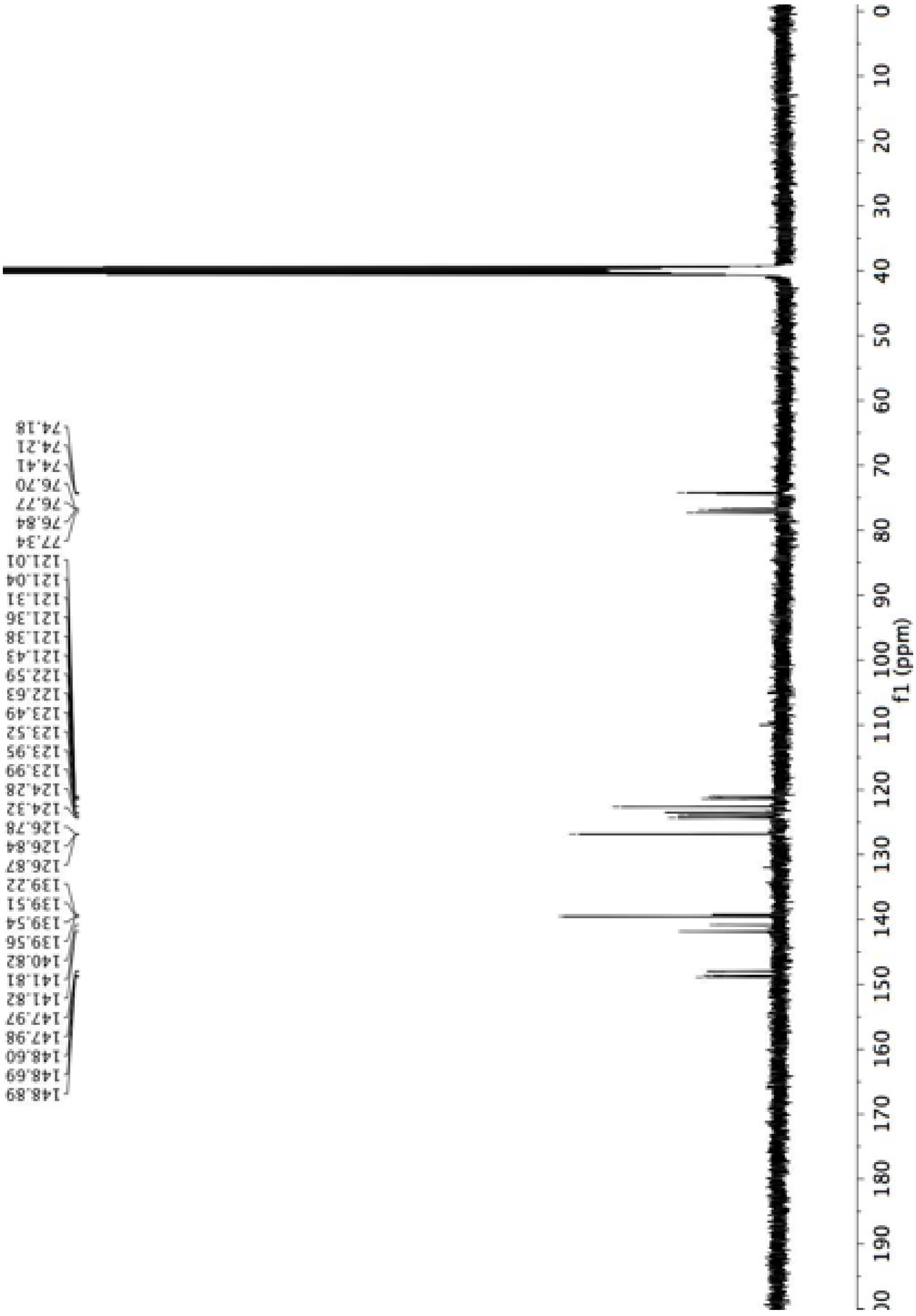
74.18
74.21
74.41
76.70
76.77
76.84
77.34
121.01
121.04
121.31
121.36
121.38
121.43
122.59
122.63
123.49
123.52
123.95
123.99
124.28
124.32
126.78
126.84
126.87
139.22
139.51
139.54
139.56
140.82
141.81
141.82
147.97
147.98
148.60
148.69
148.89
0
10
20
30
40
50
60
70
80
90
100
110
120
130
140
150
160
170
180
190
f1 (ppm)

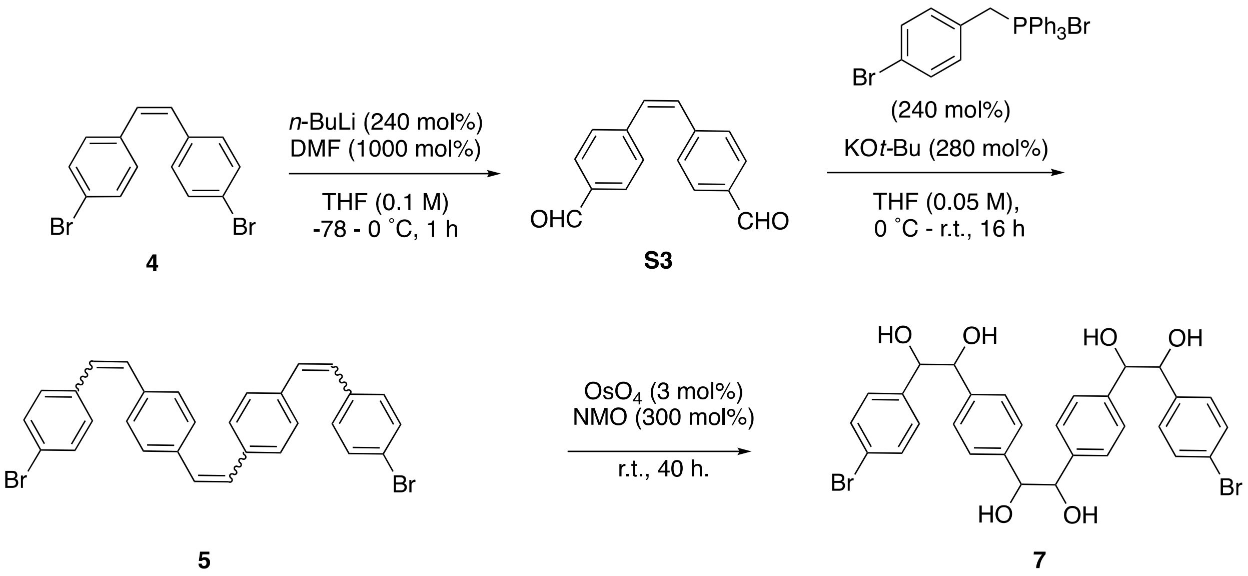

Bromide **4** was synthesized according to known procedures[iii] and their characterization data is identical in all respects.

(*Z*)-4,4'-(ethene-1,2-diyl)dibenzaldehyde (**S3**):

To a solution of alkene **4** (5.07 g, 15 mmol, 100 mol%) in anhydrous THF (150 mL) cooled to -78 ˚C was added *n*-BuLi (2.5 M in hexanes, 14.4 mL, 36.0 mmol, 240 mol%). The mixture was allowed to stir at the same temperature for 30 min followed by the addition of freshly distilled DMF (11.6 mL, 150 mmol, 1000 mol%). The reaction was then warmed to 0 ˚C and allowed to stir for 1 hour. The reaction was subsequently quenched by the addition of saturated NH_4Cl solution (50 mL). The aqueous layer was then extracted with Et_2O (3 x 50 mL) and the combined organic layers were washed with brine (50 mL), dried (Na_2SO_4) and filtered. Evaporation under reduced pressure provided a solid, which was subjected to flash column chromatography (SiO_2; hexanes: ethyl acetate = 4:1) to furnish the title compound **S3** (2.66 g, 11.3 mmol) in 75% yield as a white solid.

TLC (SiO_2): R_f = 0.28 (hexanes : ethyl acetate = 70:30).
^{1}H NMR: (400 MHz, $CDCl_3$): δ = 10.0 (s, 2H), 7.90 (d, *J* = 7.9 Hz, 4H), 7.69 (d, *J* = 7.9 Hz, 4H), 7.30 (s, 2H) ppm.
^{13}C NMR: (100 MHz, $CDCl_3$): δ = 191.5, 142.4, 135.9, 130.7, 130.3, 127.3 ppm.
HRMS: (Cl+) Calculated for $C_{16}H_{12}O_2$ [M^+] = 236.0837, Found 236.0840.
FTIR: (neat): 2918, 2849, 1737, 1366, 1217 cm^{-1}.

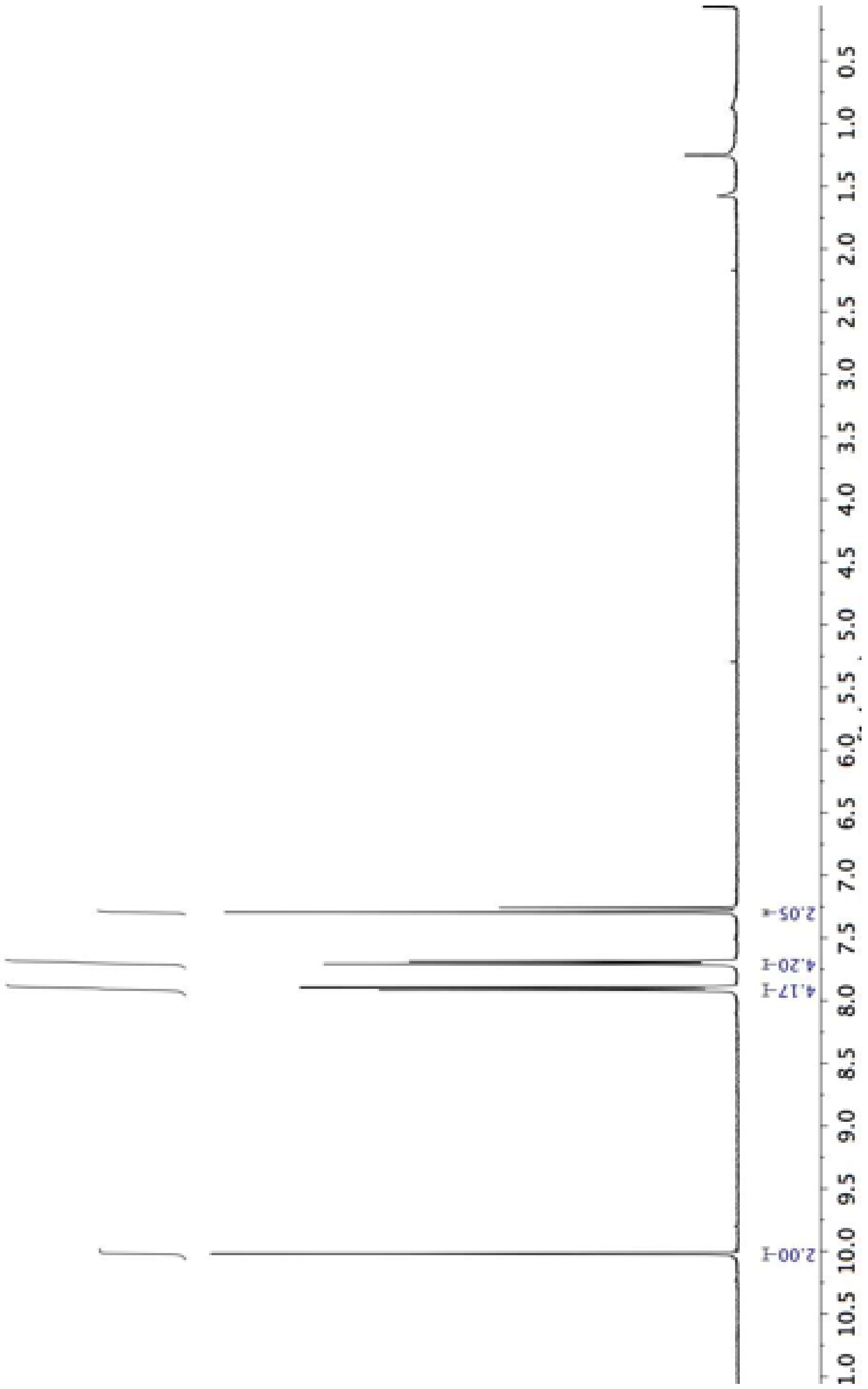

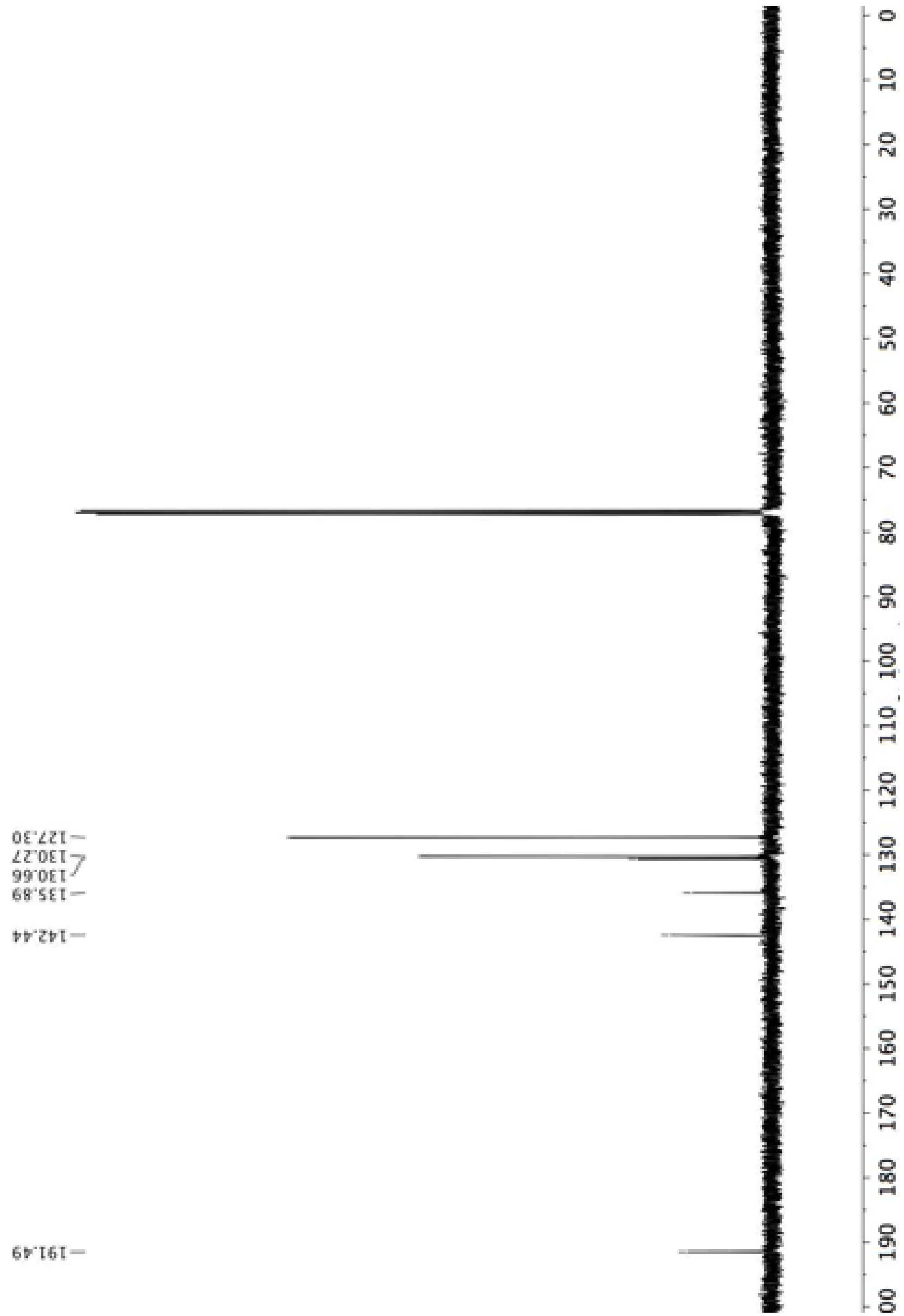

191.49
142.44
135.89
130.66
130.27
127.30
200
190
180
170
160
150
140
130
120
110
100
90
80
70
60
50
40
30
20
10
0

1,2-bis(4-(4-bromostyryl)phenyl)ethene (**5**):

To a solution of KO*t*-Bu (1.12 g, 28.0 mmol, 280 mol%) in anhydrous THF (100 mL) cooled to 0 ˚C was added phosphonium salt (12.3 g, 24.0 mmol, 240 mol%). The mixture was allowed to stir at the same temperature for 30 min followed by the addition of dialdehyde **S3** (2.36 g, 10.0 mmol, 100 mol%) in THF (100 mL) dropwise over 30 min. The reaction was then warmed to room temperature and allowed to stir for 16 hours. The solution was concentrated under vacuum followed by addition of water (50 mL). The aqueous layer was then extracted with Et_2O (3 x 25 mL) and the combined organic layers were washed with brine (50 mL), dried (Na_2SO_4) and filtered. Evaporation under reduced pressure provided an oily residue which was subjected to flash column chromatography (SiO_2; hexanes:ethyl acetate = 97.5:2.5) to furnish the title compound **5** (3.69 g, 6.8 mmol) in 68% yield as a yellow solid.

TLC (SiO_2): R_f = 0.73 (hexanes : ethyl acetate = 90:10).

^{1}H NMR: (400 MHz, $CDCl_3$, 3 diastereomers): δ = 7.41–7.39 (m, 1H), 7.30–7.24 (m, 5H), 7.09–6.96 (m, 10H), 6.52–6.40 (m, 5H) ppm.

^{13}C NMR: (100 MHz, $CDCl_3$, 3 diastereomers): δ = 136.3, 136.2, 135.7, 131.8, 131.4, 130.8, 130.7, 130.6, 130.5, 130.0, 129.3, 129.1, 129.0, 129.0, 128.9, 128.8, 128.7, 128.7, 128.0, 127.4, 126.4, 121.0 ppm.

HRMS: (CI) Calculated for $C_{30}H_{22}Br_2$ [M $^+$] = 542.0068, Found 542.0058.

FTIR: (neat): 1738, 1366, 1216 cm^{-1}.

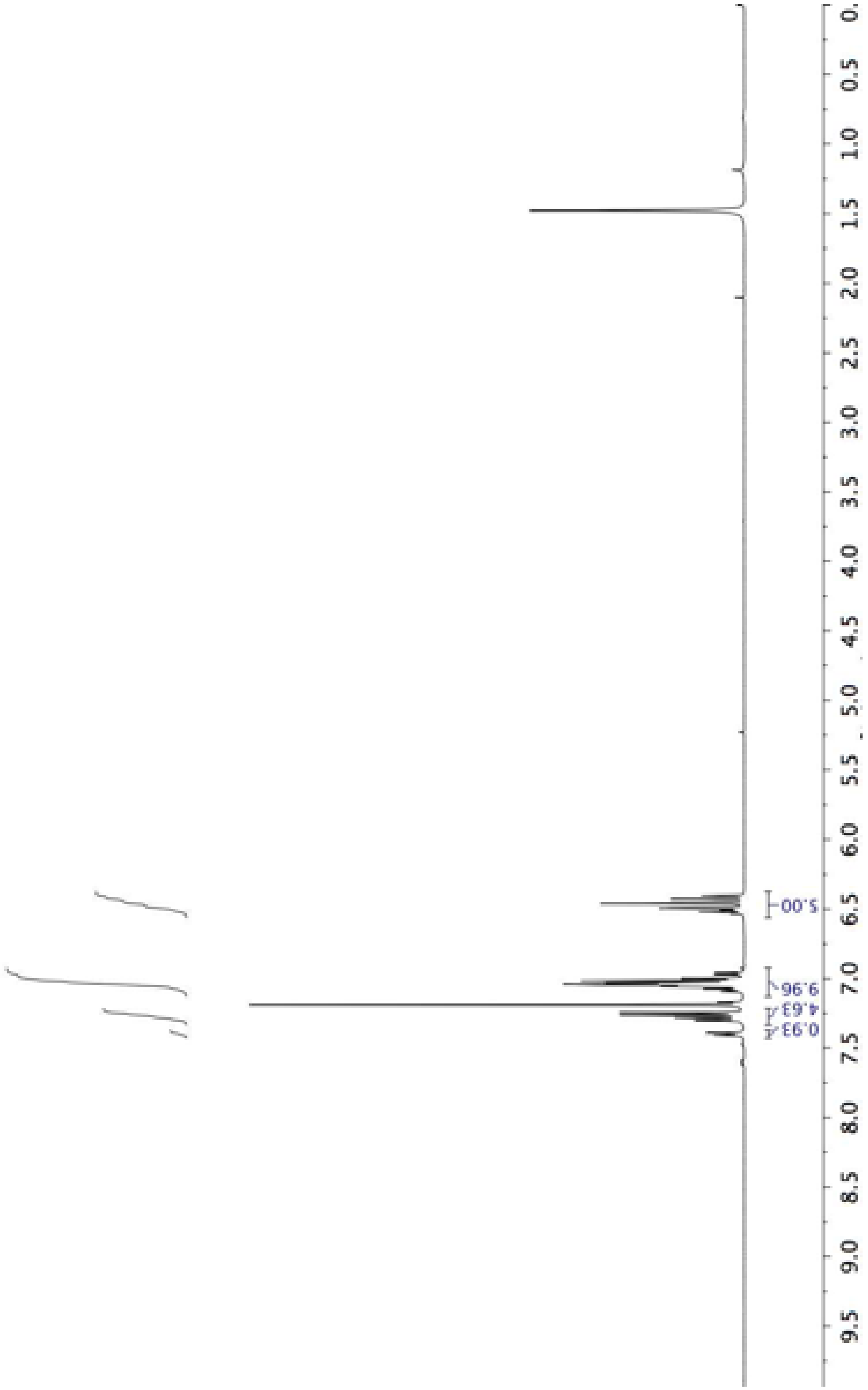
0.0
0.5
1.0
1.5
2.0
2.5
3.0
3.5
4.0
4.5
5.0
5.5
6.0
6.5
7.0
7.5
8.0
8.5
9.0
9.5
5.00
9.96
4.63
0.93

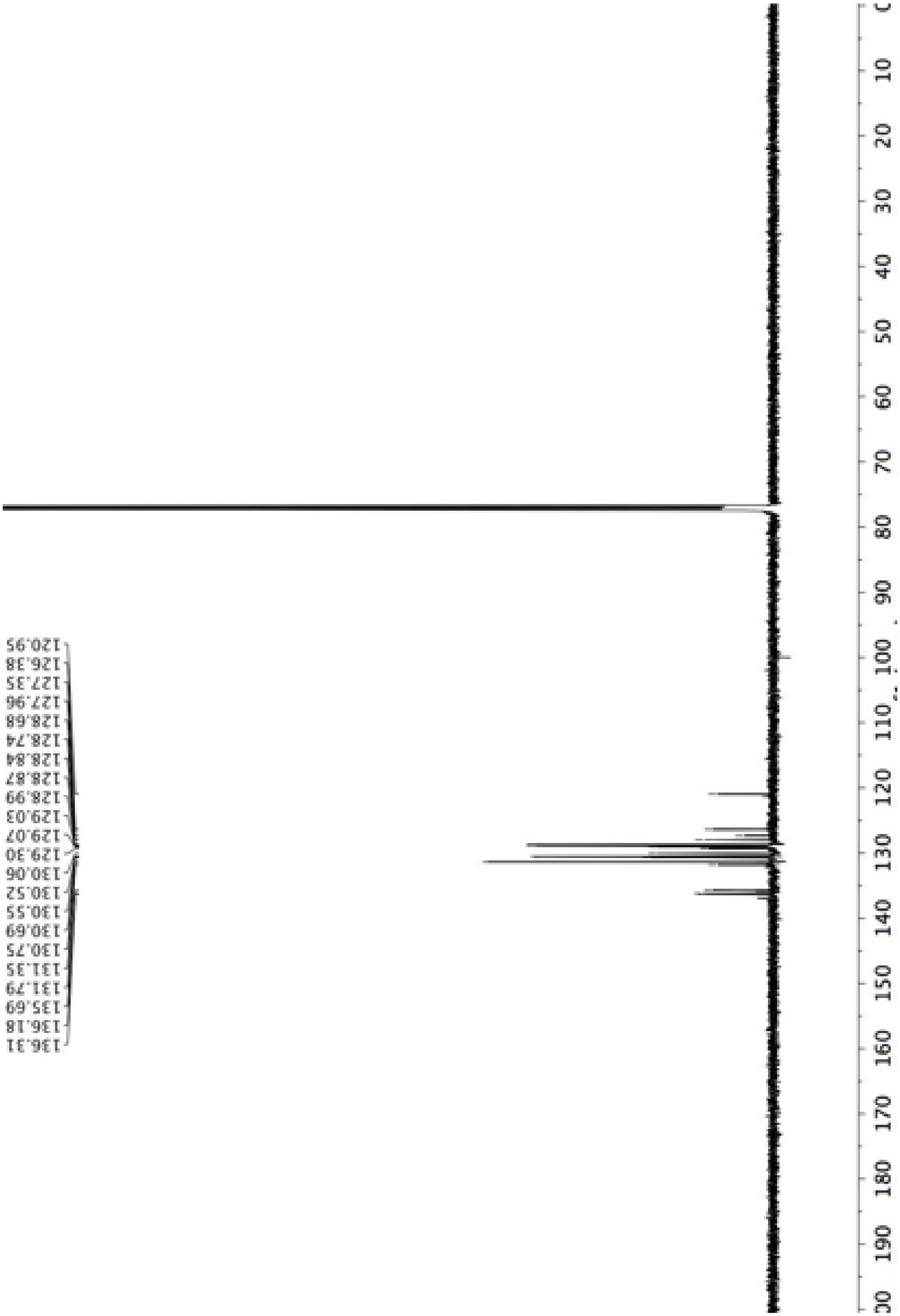
120.95
126.38
127.35
127.96
128.68
128.74
128.84
128.87
128.99
129.03
129.07
129.30
130.06
130.52
130.55
130.69
130.75
131.35
131.79
135.69
136.18
136.31
10
20
30
40
50
60
70
80
90
100
110
120
130
140
150
160
170
180
190

2,2'-((1,2-dihydroxyethane-1,2-diyl)bis(4,1-phenylene))bis(1-(4-bromophenyl)ethane-1,2-diol) (7):

To a solution of triene **5** (2.71 g, 5.0 mmol, 100 mol%) in acetone (38 mL), chloroform (19 mL), and water (18 mL) was added NMO in water (w/w 50%) (3.51 g, 15.0 mmol, 300 mol%) followed by OsO_4 (1M in *t*BuOH, 0.15 mL, 3 mol%). The mixture was allowed to stir 40 hours. Toluene (30 mL) was added, and concentrated under vacuum. Provided solid was subjected to flash column chromatography (SiO_2; hexanes:ethyl acetate = 1:1 to ethyl acetate = 1) to furnish the title compound **7** (2.26 g, 3.5 mmol) in 70% yield as a white solid.

TLC (SiO_2): R_f = 0.42 (ethyl acetate : MeOH = 95:5).

^{1}H NMR: (400 MHz, d_6-DMSO): major diastereomer δ = 7.45–7.40 (m, 4H), 7.18–7.13 (m, 4H), 7.07–6.95 (m, 8H), 5.31 (d, *J* = 3.9 Hz, 2H), 5.21 (d, *J* = 3.9 Hz, 2H), 5.11–5.07 (m, 2H), 4.62– 4.51 (m, 6H) ppm.

^{1}H NMR: (400 MHz, d_6-DMSO): minor diastereomer δ = 7.37–7.35 (m, 4H), 7.07–6.95 (m, 12H), 5.42 (d, *J* = 4.3 Hz, 2H), 5.34 (d, *J* = 4.3 Hz, 2H), 5.11–5.07 (m, 2H), 4.62– 4.51 (m, 6H) ppm.

^{13}C NMR: (100 MHz, d_6-DMSO, 2 diastereomers): δ = 143.1, 142.4, 141.9, 141.6, 141.5, 140.6, 138.1, 130.5, 130.5, 130.1, 129.9, 126.9, 126.8, 126.6, 126.5, 120.1, 120.1, 77.7, 77.4, 77.1, 77.0, 77.0 ppm.

HRMS: (ESI) Calculated for $C_{30}H_{28}Br_2O_6$ [M+H$^+$] = 667.0127, Found 667.0124.

FTIR: (neat): 3365, 2359, 1030 cm^{-1}.

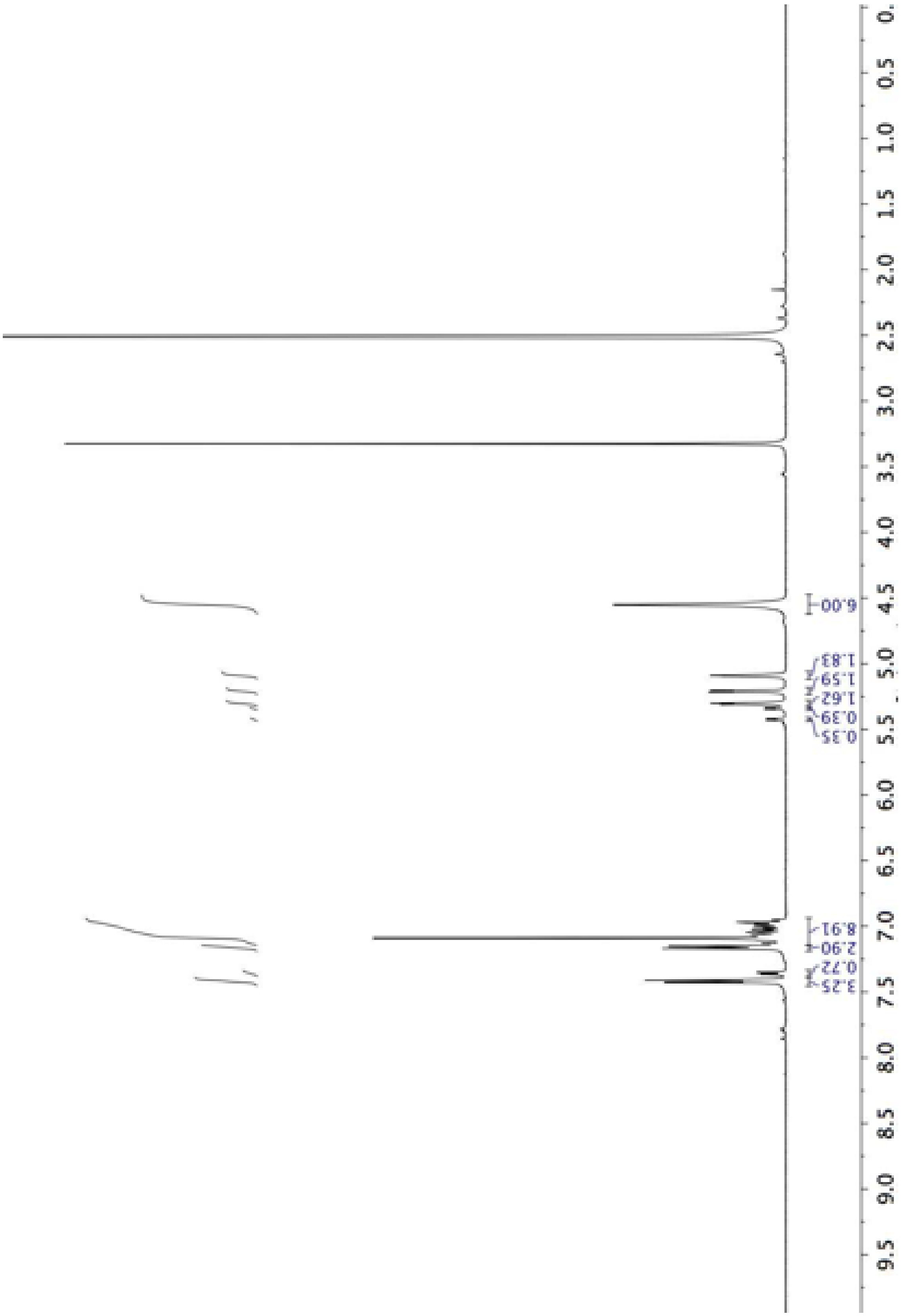
6.00
1.83
1.59
1.62
0.39
0.35
8.91
2.90
0.72
3.25
0.
0.5
1.0
1.5
2.0
2.5
3.0
3.5
4.0
4.5
5.0
5.5
6.0
6.5
7.0
7.5
8.0
8.5
9.0
9.5

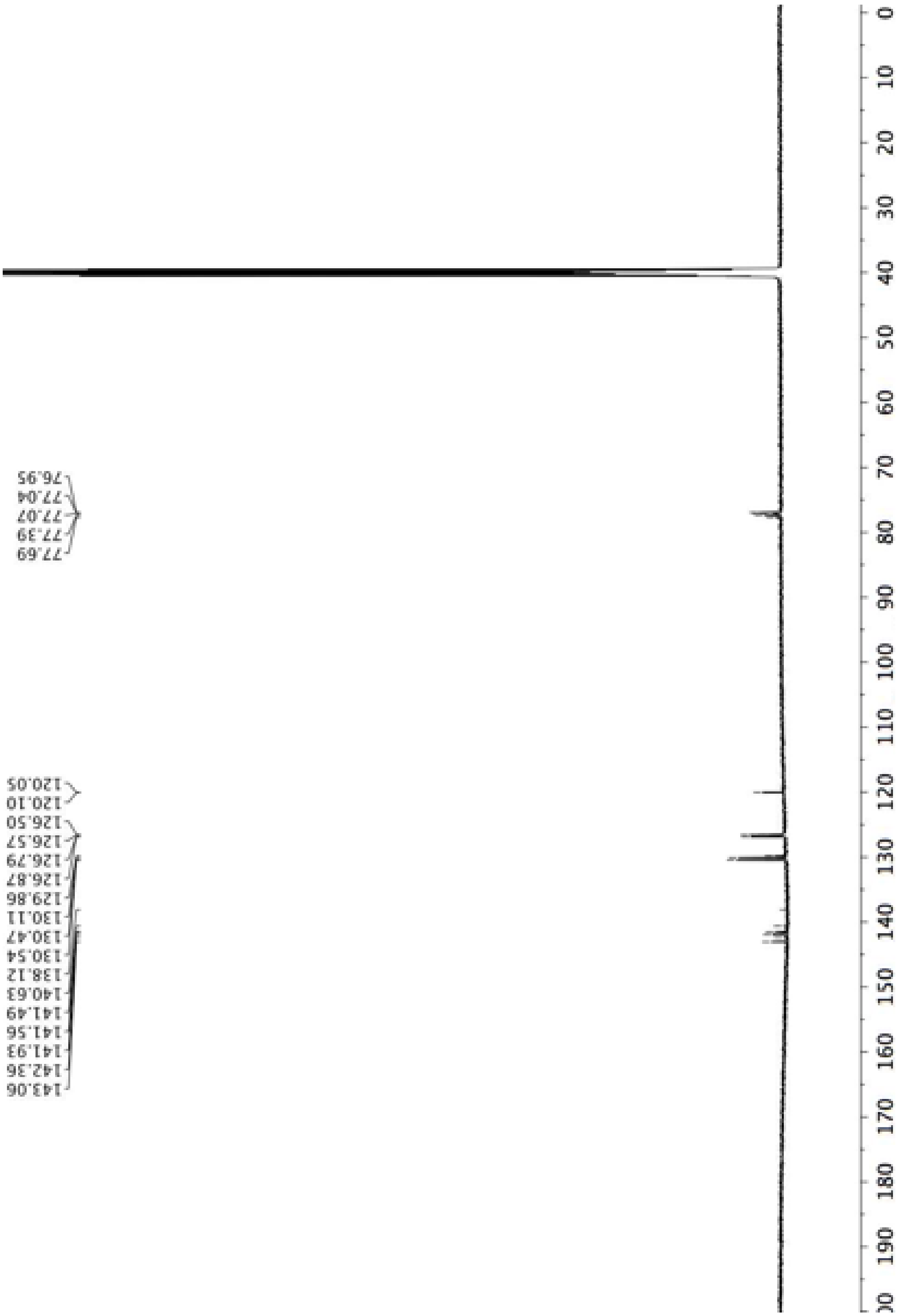
76.95
77.04
77.07
77.39
77.69
120.05
120.10
126.50
126.57
126.79
126.87
129.86
130.11
130.47
130.54
138.12
140.63
141.49
141.56
141.93
142.36
143.06
0
10
20
30
40
50
60
70
80
90
100
110
120
130
140
150
160
170
180
190

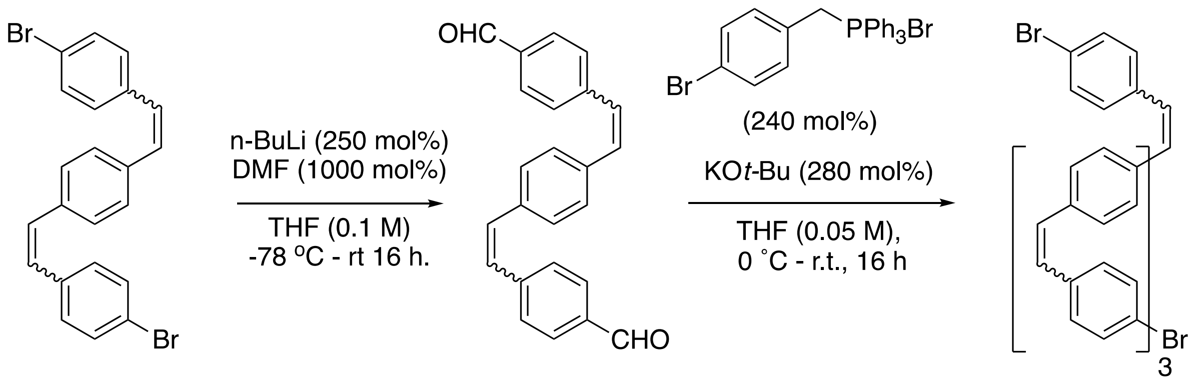

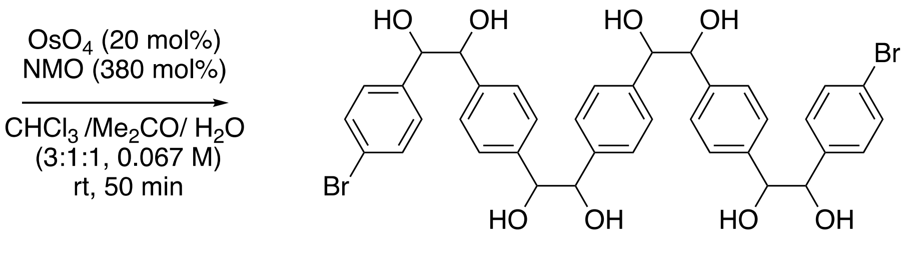

Dialdehyde **S4** was synthesized analogously to known procedures[iv] and their characterization data is identical in all respects.

1,4-bis(4-(4-bromostyryl)styryl)benzene **(6)**

To a solution of KO*t*-Bu (0.84g, 7.5 mmol, 280 mol%) in anhydrous THF (75 mL) cooled to 0 ˚C was added Wittig reagent (3.07 g, 6.0 mmol, 240 mol%). The mixture was allowed to stir at the same temperature for 30 min followed by the addition of dialdehyde **S4** (1.34 g, 10.0 mmol, 100 mol%) in THF (75 mL) dropwise over 30 min. The reaction was then warmed to room temperature and allowed to stir for 16 hours. The solution was concentrated under vacuum followed by addition of water (25 mL). The aqueous layer was then extracted with Et_2O (3 x 25 mL) and the combined organic layers were washed with brine (50 mL), dried (Na_2SO_4) and filtered. Evaporation under reduced pressure provided an oily residue which was subjected to flash column chromatography (short plug, SiO_2; hexanes) to furnish the title compound **2c** (1.08 g, 1.67 mmol, mixture of isomers) in 67% yield as a waxy yellow solid.

TLC (SiO_2): R_f = 0.67 (hexanes).

^{1}H NMR: (500 MHz, $CDCl_3$, diastereomeric mixture): δ = 7.48 (t, *J* = 7.9 Hz, 3H), 7.36 (t, *J* = 8.5 Hz, 4H), 7.31 (dd, *J* = 8.4, 3.7 Hz, 3H), 7.14-7.06 (m, 10 H), 7.03-6.99 (m, 2H), 6.55 (d, *J* = 15.3 Hz, 5H), 6.46 (t, *J* = 11.8 Hz, 1H) ppm.

^{13}C NMR: (125 MHz, $CDCl_3$, diastereomeric mixture) δ 137.0, 136.4, 136.3, 136.1, 136.1, 135.9, 135.8, 135.6, 135.6, 131.9, 131.8, 131.3, 131.3, 130.7, 130.7, 130.5, 130.2, 130.1, 129.9, 129.9, 129.3, 129.0, 128.98, 128.95, 128.85, 128.76, 128.73, 128.70, 128.65, 128.12, 128.00, 127.92, 127.25, 126.36, 121.6, 121.3, 121.0 ppm.

HRMS: (CI^+) Calculated for $C_{38}H_{28}Br_2[M^+]$ = 642.0558, Found 642.0560.

FTIR: (neat): 3015, 1484, 1070, 1009, 961, 880, 812, 754, 695 cm^{-1}.

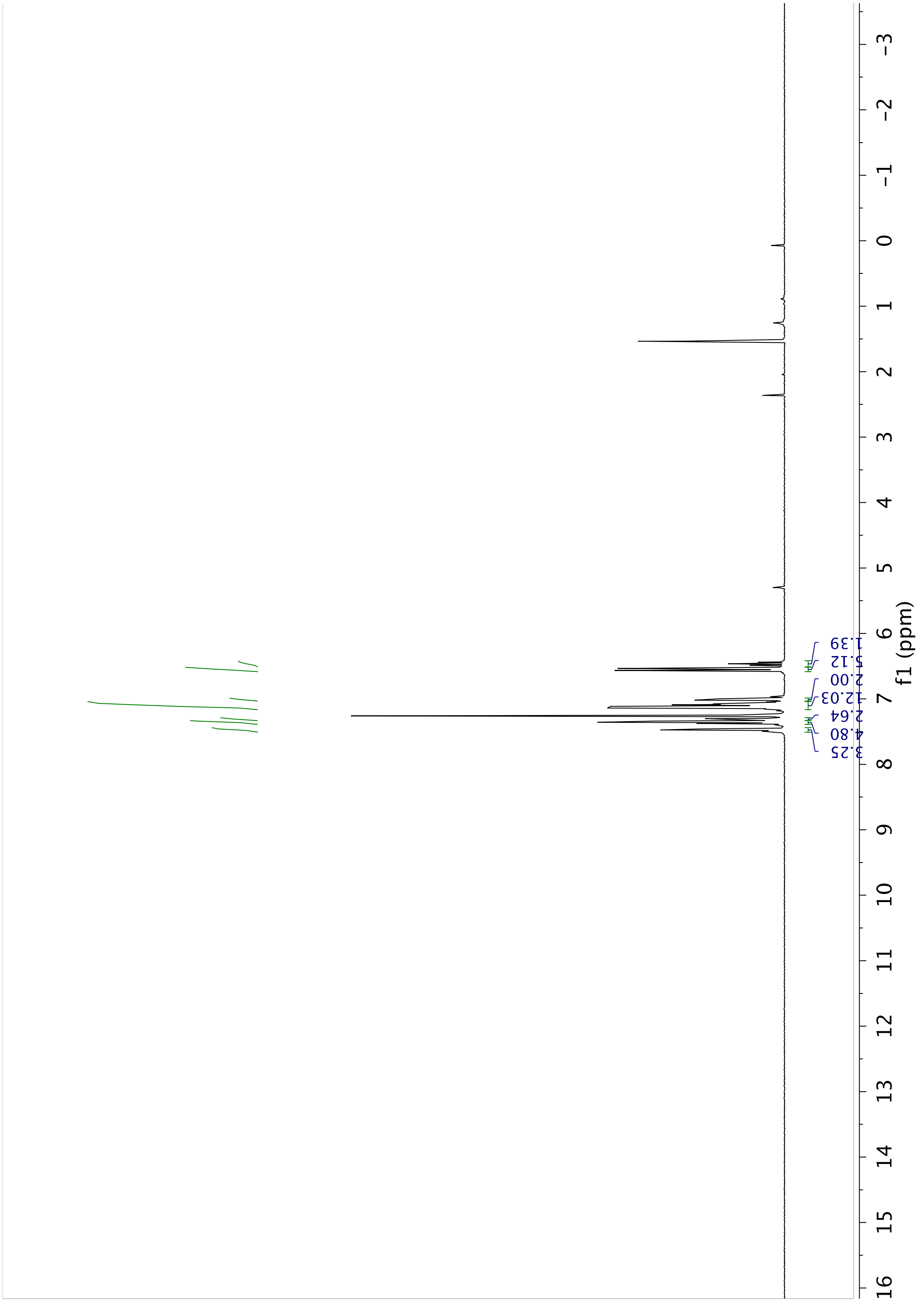
1.39
5.12
2.00
12.03
2.64
4.80
3.25
f1 (ppm)

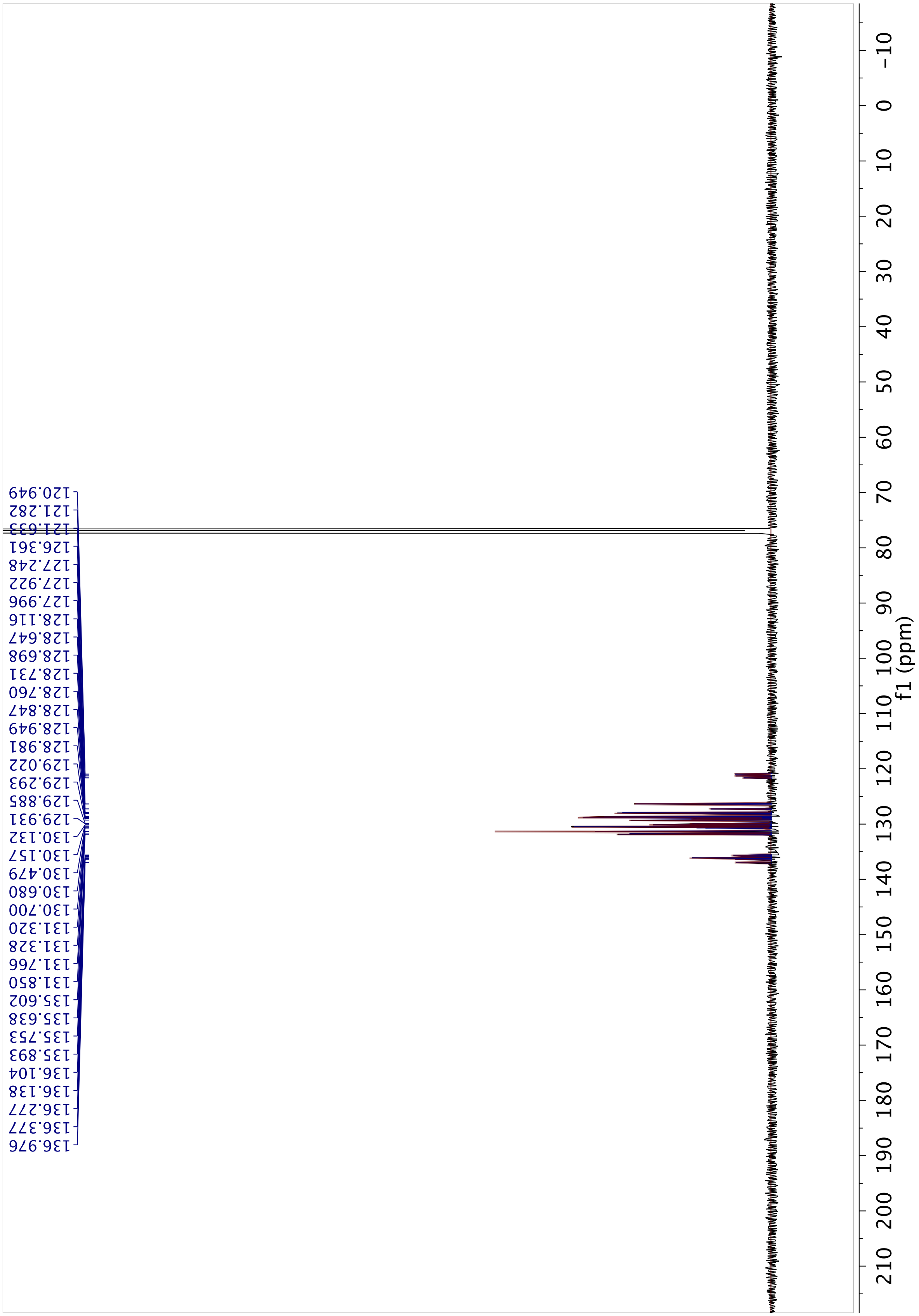
120.949
121.282
121.633
126.361
127.248
127.922
127.996
128.116
128.647
128.698
128.731
128.760
128.847
128.949
128.981
129.022
129.293
129.885
129.931
130.132
130.157
130.479
130.680
130.700
131.320
131.328
131.766
131.850
135.602
135.638
135.753
135.893
136.104
136.138
136.277
136.377
136.976
f1 (ppm)

2,2'-(1,4-phenylene)bis(1-(4-(2-(4-bromophenyl)-1,2-dihydroxyethyl)phenyl)ethane-1,2-diol) (**8**)

To a solution of tetraene **6** (65 mg, 0.1 mmol, 100 mol%) in acetone (0.9 mL), chloroform (0.3 mL), and water (0.3 mL) was added NMO in water (w/w 50%) (94 mg, 0.4 mmol, 400 mol%). The mixture was allowed to stir for 50 min. Silica and ~100 mg of activated carbon was added to the reaction mixture, and volatiles were removed under vacuum. The resulting solids were loaded onto a column of silica and subjected to flash column chromatography (SiO_2; CH_2Cl_2: ethyl acetate = 1:1 to CH_2Cl_2: ethyl acetate: MeOH = 4:3:1) and then triterated with Et_2O to furnish the title compound **8** (35 mg, 0.045 mmol) in 45% yield as a gray solid.

TLC (SiO_2): R_f = 0.30, 0.25 (inseparable isomers, CH_2Cl_2: ethyl acetate: MeOH = 4:3:1).

^{1}H NMR: (500 MHz, CD_3OD, water suppression, mixtures of isomers): δ = 7.85-7.67 (m, 2H), 7.41-7.42 (m, 5H), 7.13-6.84 (m, 13H), 4.74-4.52 (m, 1.5H), 3.74 (s, 2H), 3.27 (s, 0.5 H), 2.76 (s, 2H), 2.50 (s, 2H) ppm.

^{13}C NMR: (125 MHz, CD_3OD, mixture of isomers): δ = 141.9, 141.5, 141.5, 141.4, 141.3, 131.9, 131.6, 130.7, 130.5, 130.4, 127.9, 127.7, 122.0, 121.9, 80.1, 79.6, 78.9, 78.6, 78.5, 66.5, 55.6, 49.9, 45.4, 30.8 ppm.

HRMS: (ESI^+) Calculated for $C_{38}H_{36}Br_2O_8$ [$M+Na^+$] = 801.0669, Found 801.0661.

FTIR: (neat): 3370, 2389, 1045, 895, 678 cm^{-1}.

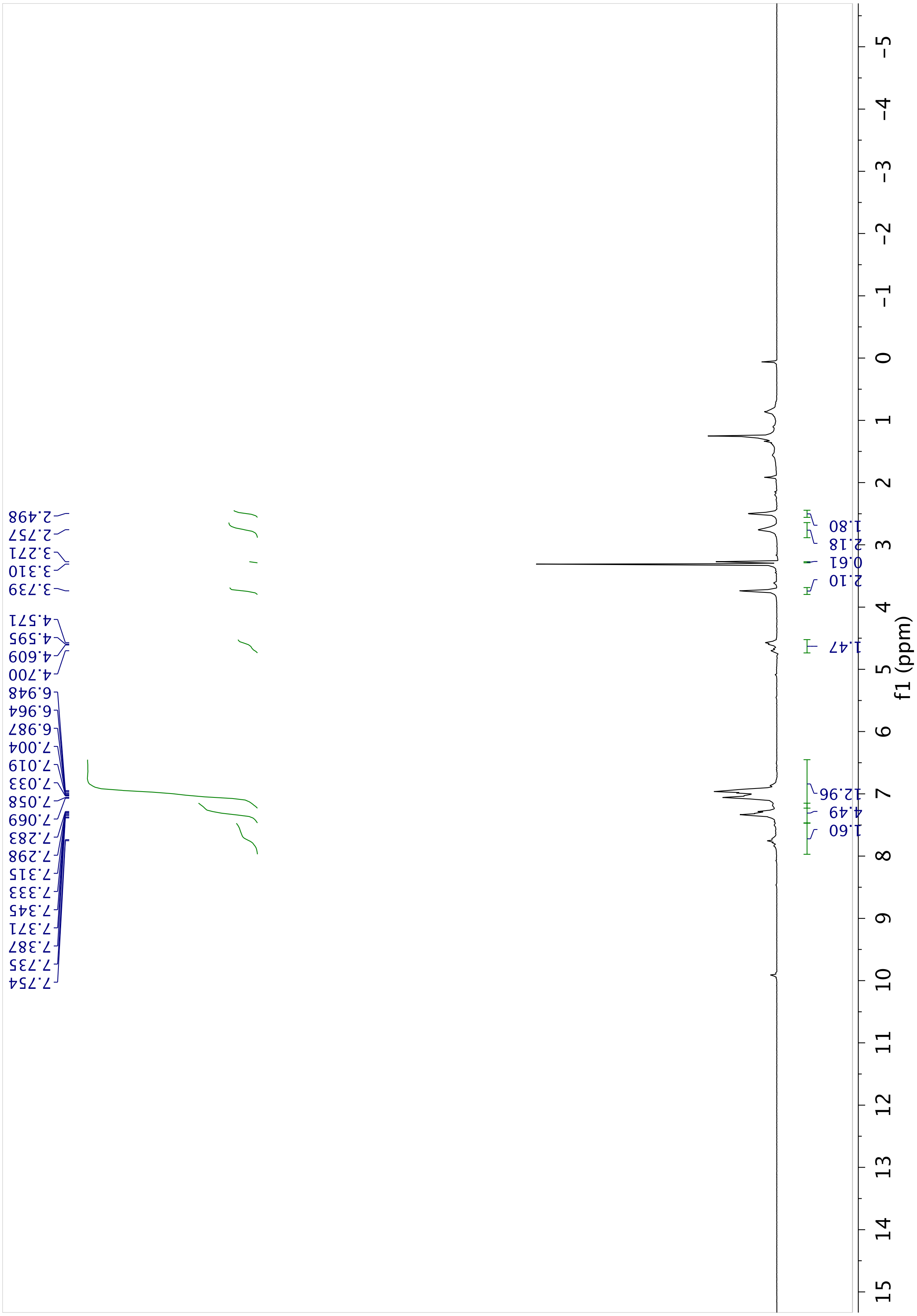
2.498
2.757
3.271
3.310
3.739
4.571
4.595
4.609
4.700
6.948
6.964
6.987
7.004
7.019
7.033
7.058
7.069
7.283
7.298
7.315
7.333
7.345
7.371
7.387
7.735
7.754
1.80
2.18
0.61
2.10
1.47
12.96
4.49
1.60
f1 (ppm)

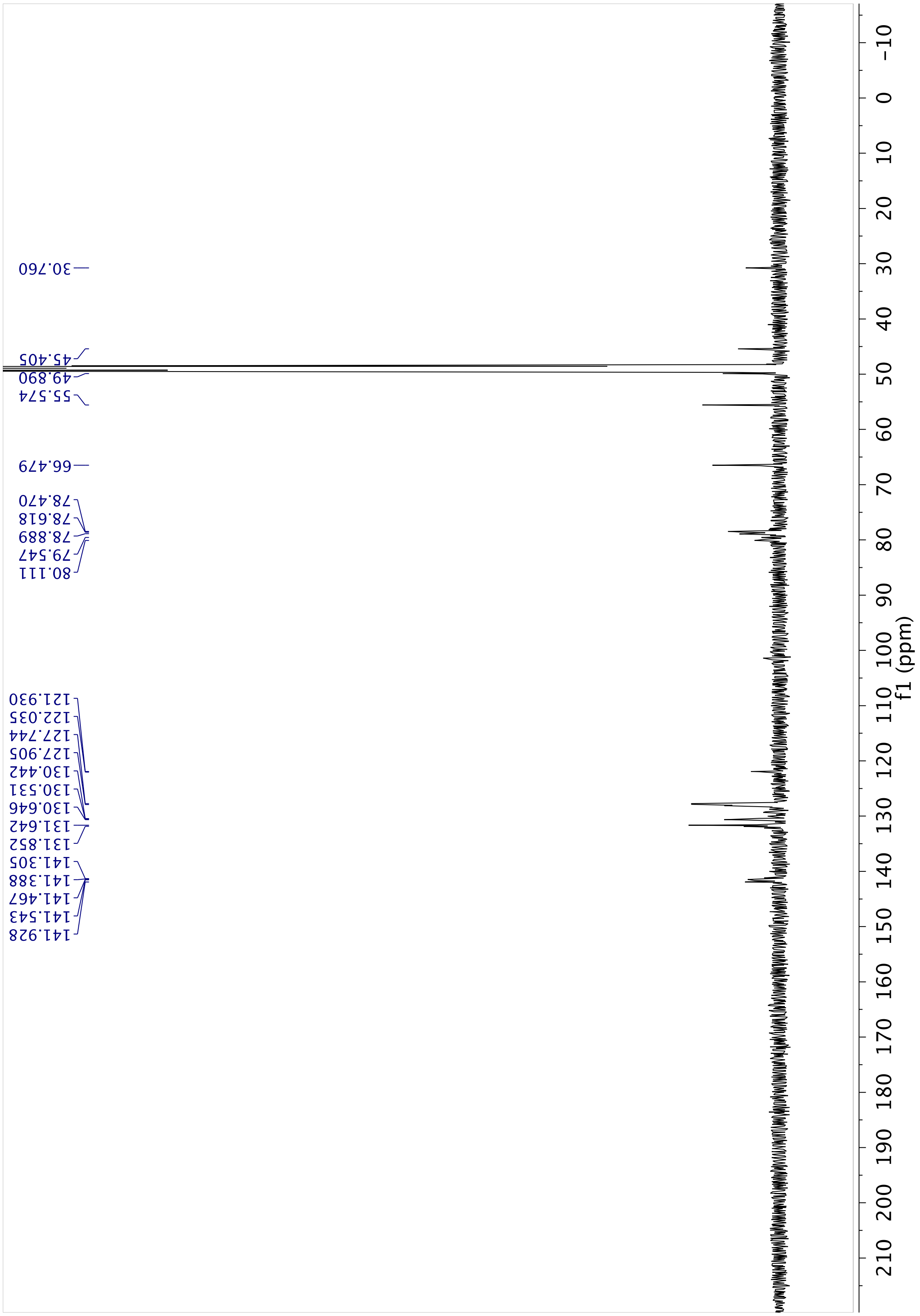
30.760
45.405
49.890
55.574
66.479
78.470
78.618
78.889
79.547
80.111
121.930
122.035
127.744
127.905
130.442
130.531
130.646
131.642
131.852
141.305
141.388
141.467
141.543
141.928
f1 (ppm)

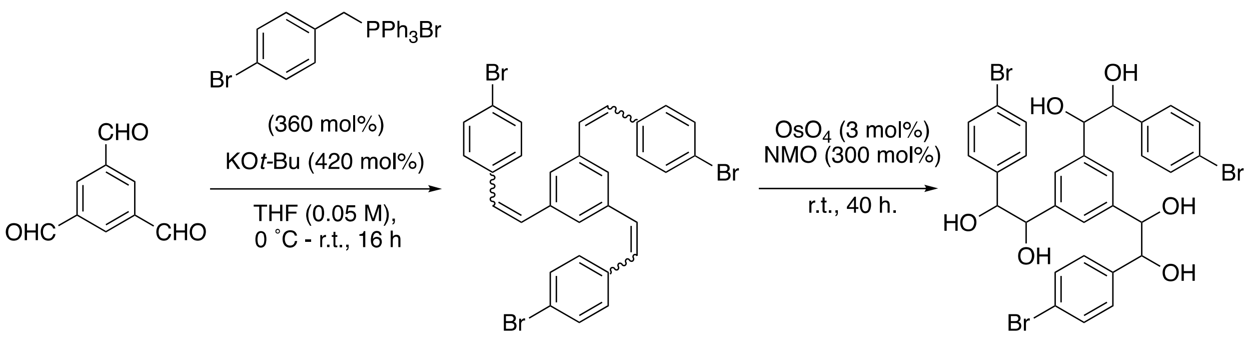

1b **S5** **9**

Benzene-1,3,5-tricarboxaldehyde **1b** was synthesized according to known procedures[v] and their characterization data match our own in all respects.

1,3,5-tris(4-bromostyryl)benzene (**S5**)

To a solution of KO*t*-Bu (4.72 g, 42.0 mmol, 420 mol%) in anhydrous THF (100 mL) cooled to 0 ˚C was added phosphonium salt (18.4 g, 36.0 mmol, 360 mol%). The mixture was allowed to stir at the same temperature for 30 min followed by the addition of tricarbonyl **1b** (1.62 g, 10.0 mmol, 100 mol%) in THF (100 mL) dropwise over 30 min. The reaction was then warmed to room temperature and allowed to stir for 16 hours. The solution was concentrated under vacuum followed by addition of water (50 mL). The aqueous layer was then extracted with Et_2O (3 x 25 mL) and the combined organic layers were washed with brine (50 mL), dried (Na_2SO_4) and filtered. Evaporation under reduced pressure provided an oily residue which was subjected to flash column chromatography (SiO_2; hexanes:ethyl acetate = 99:1 to 98:2) to furnish the title compound **S5** (4.41 g, 7.1 mmol) in 71% yield as a yellow solid.

TLC (SiO_2): R_f = 0.73 (hexanes : ethyl acetate = 90:10).
^{1}H NMR: (400 MHz, $CDCl_3$, 3 diastereomers): δ = 7.53–6.86 (m, 17H), 6.66–6.42 (m, 4H) ppm.
^{13}C NMR: (100 MHz, $CDCl_3$, 3 diastereomers): δ = 136.8, 136.5, 136.5, 136.4, 136.2, 136.1, 136.1, 135.0, 135.0, 134.9, 134.9, 134.9, 134.7, 132.8, 132.6, 120.8, 130.8, 130.8, 130.7, 130.5, 130.5, 130.4, 130.3, 129.5, 129.4, 129.4, 129.4, 129.3, 129.2, 129.2, 128.7, 128.5, 128.3, 127.8, 127.7, 127.5, 127.5, 127.4, 127.4, 127.2, 127.2, 127.1, 127.0, 126.9, 125.2, 124.9, 123.2, 122.9, 120.6, 120.5, 120.5, 120.1, 120.0, 120.0 ppm.
HRMS:(CI) Calculated for $C_{30}H_{21}Br_3$ [M^+] = 621.9173, Found 621.9167.
FTIR: (neat): 3012, 2692, 1485, 1071, 1009 cm^{-1}.

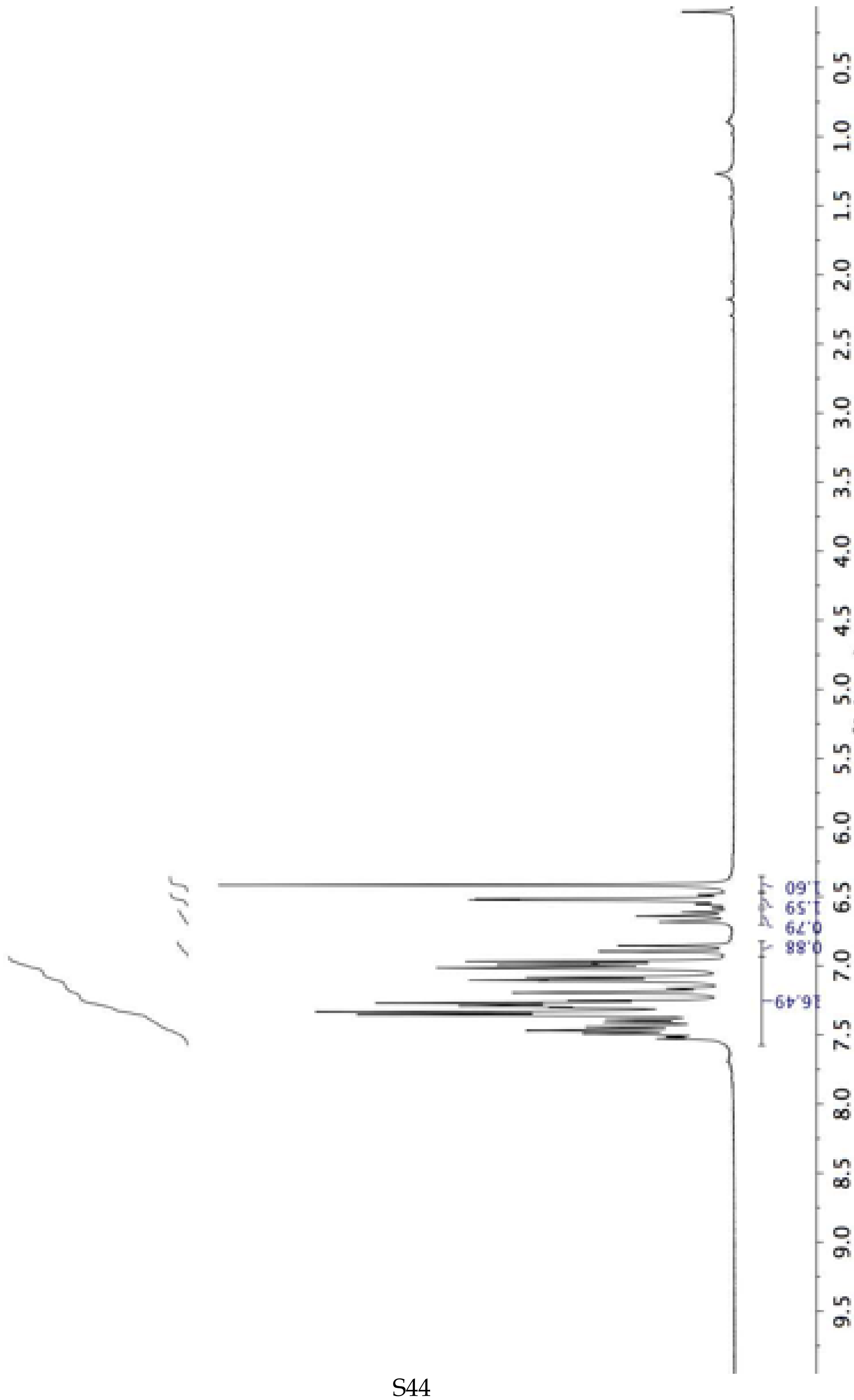

1.60
1.59
0.79
0.88
16.49
0.5
1.0
1.5
2.0
2.5
3.0
3.5
4.0
4.5
5.0
5.5
6.0
6.5
7.0
7.5
8.0
8.5
9.0
9.5

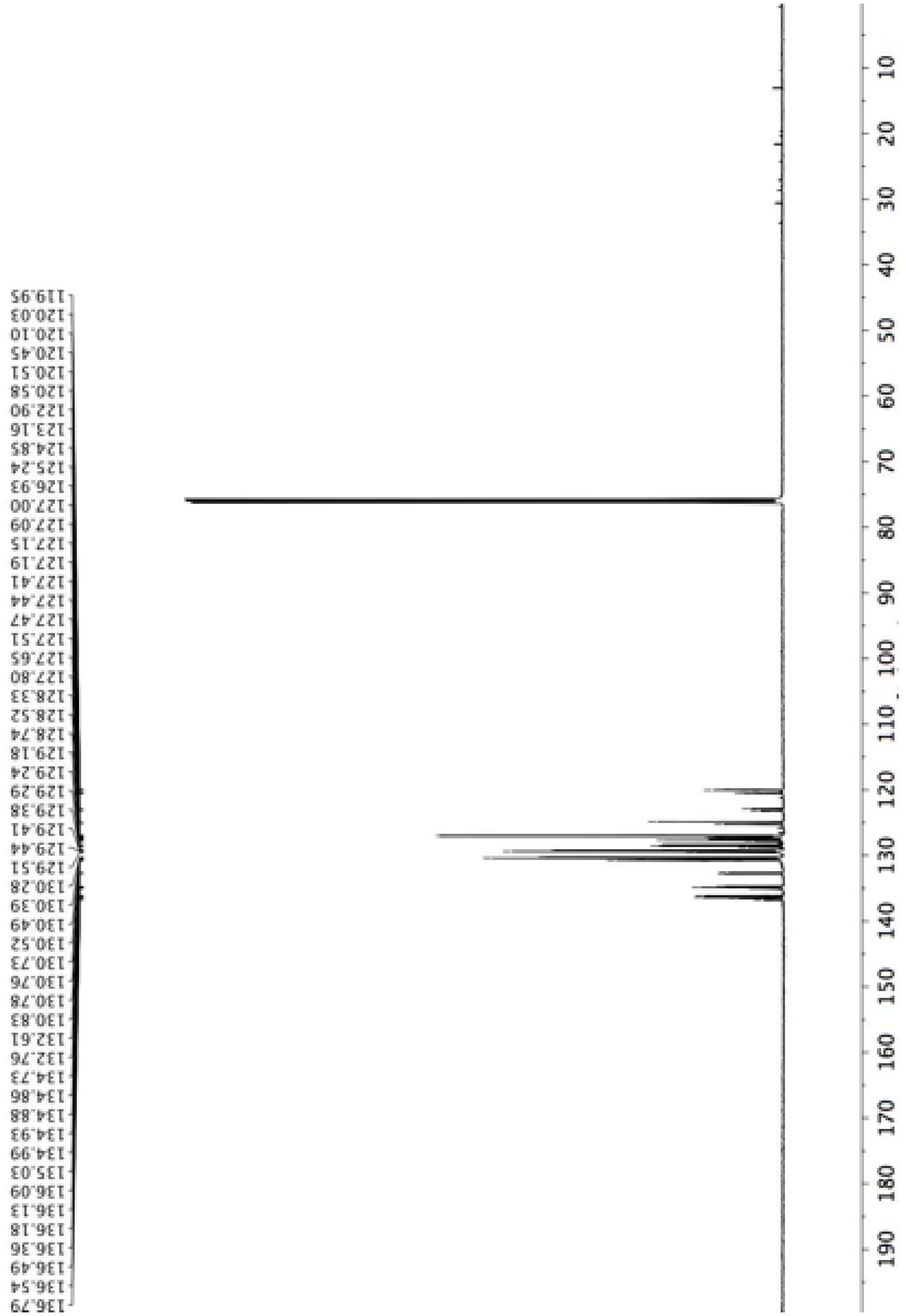
119.95
120.03
120.10
120.45
120.51
120.58
122.90
123.16
124.85
125.24
126.93
127.00
127.09
127.15
127.19
127.41
127.44
127.47
127.51
127.65
127.80
128.33
128.52
128.74
129.18
129.24
129.29
129.38
129.41
129.44
129.51
130.28
130.39
130.49
130.52
130.73
130.76
130.78
130.83
132.61
132.76
134.73
134.86
134.88
134.93
134.99
135.03
136.09
136.13
136.18
136.36
136.49
136.54
136.79
10
20
30
40
50
60
70
80
90
100
110
120
130
140
150
160
170
180
190

2,2',2''-(benzene-1,3,5-triyl)tris(1-(4-bromophenyl)ethane-1,2-diol) (**9**)

To a solution of triene **S5** (3.11 g, 5.0 mmol, 100 mol%) in acetone (38 mL), chloroform (19 mL), and water (18 mL) was added NMO in water (w/w 50%) (3.51 g, 15.0 mmol, 300 mol%). The mixture was allowed to stir 40 hours. Toluene (30 mL) was added, and concentrated under vacuum. Provided solid was subjected to flash column chromatography (SiO_2; hexanes:ethyl acetate = 50:50 to 20:80) to furnish the title compound **9** (1.84 g, 3.5 mmol) in 52% yield as a white solid.

TLC (SiO_2): R_f = 0.42 (ethyl acetate : MeOH = 95:5).
^{1}H NMR: (400 MHz, d_6-DMSO, 2 diastereomers): δ = 7.51–7.43 (m, 7H), 7.23–6.80 (m, 8H), 5.41–5.11 (m, 6H), 4.59–4.40 (m, 6H) ppm.
^{13}C NMR: (100 MHz, d_6-DMSO, 2 diastereomers): δ = 143.2, 143.0, 142.9, 142.4, 142.0, 141.8, 141.5, 140.9, 140.7, 132.5, 132.0, 131.9, 130.6, 130.5, 130.3, 130.2, 130.2, 129.9, 129.3, 129.2, 129.2, 125.0, 120.2, 120.1, 78.2, 77.8, 77.4, 77.4, 77.2, 77.1 ppm.
HRMS: (ESI) Calculated for $C_{30}H_{28}Br_2O_6$ [M+H$^+$] = 667.0127, Found 667.0124.
FTIR: (neat): 2360, 2343, 1070, 1010cm^{-1}.

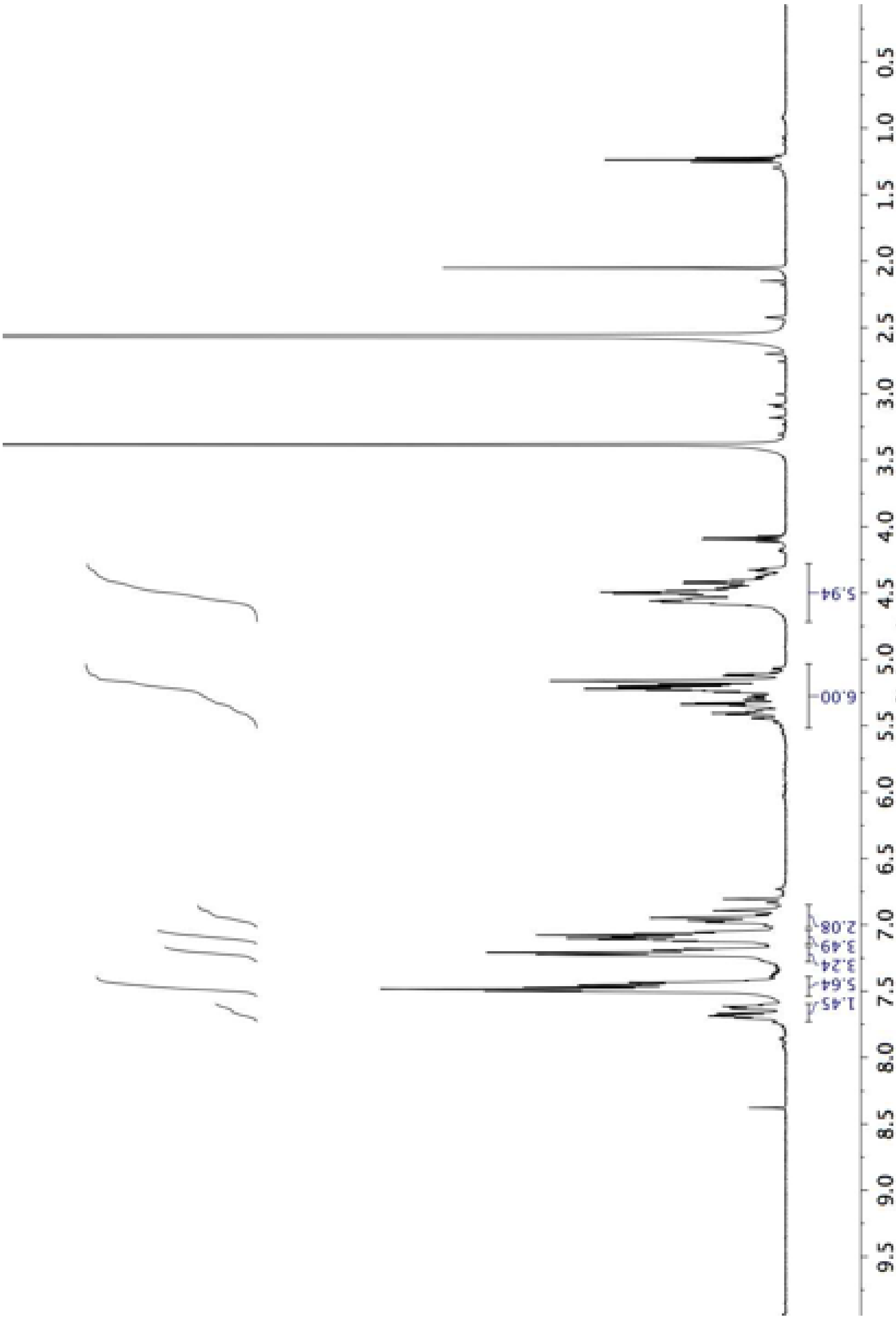

0.5
1.0
1.5
2.0
2.5
3.0
3.5
4.0
4.5
5.0
5.5
6.0
6.5
7.0
7.5
8.0
8.5
9.0
9.5
5.94
6.00
2.08
3.49
3.24
5.64
1.45

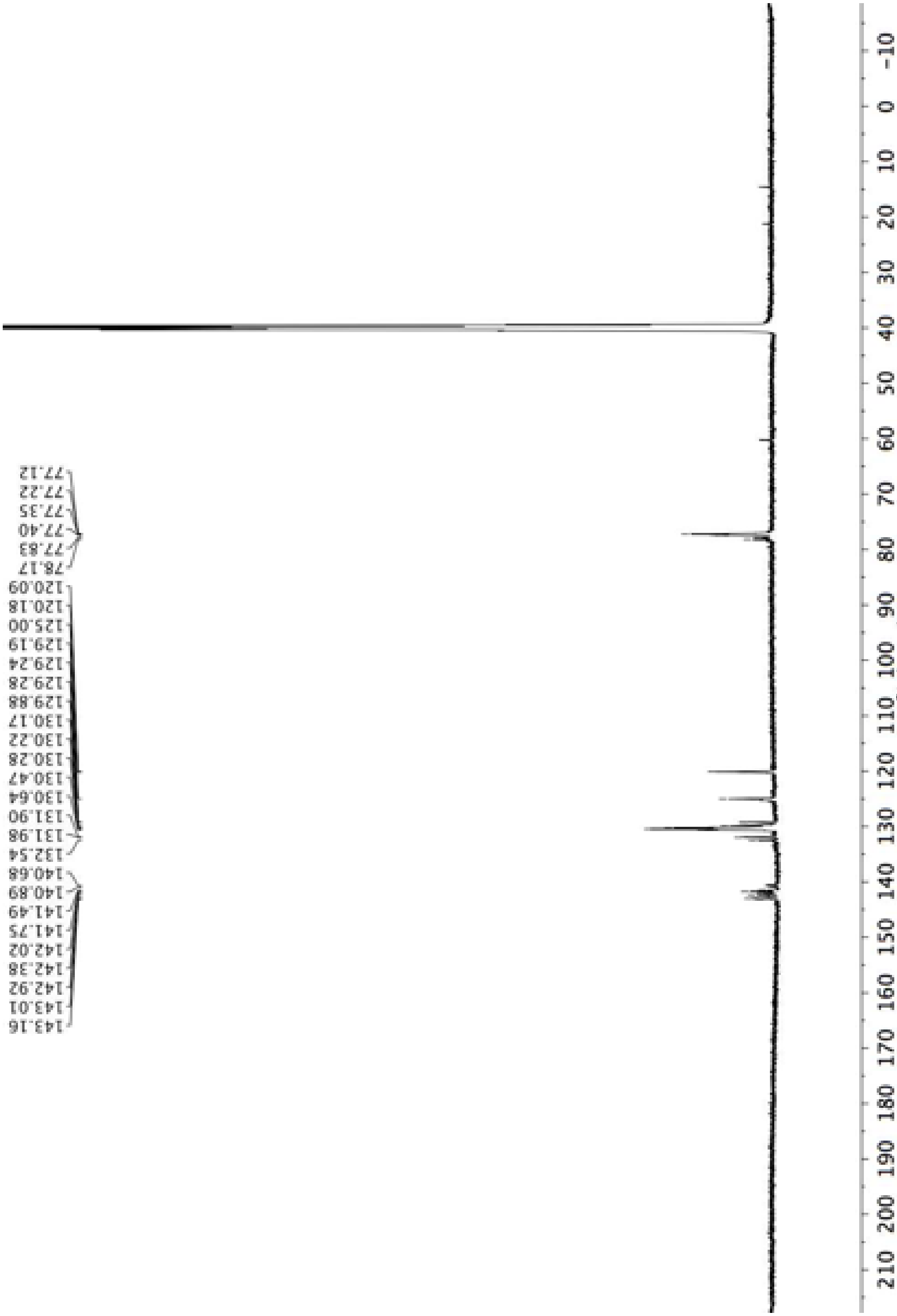
77.12
77.22
77.35
77.40
77.83
78.17
120.09
120.18
125.00
129.19
129.24
129.28
129.88
130.17
130.22
130.28
130.47
130.64
131.90
131.98
132.54
140.68
140.89
141.49
141.75
142.02
142.38
142.92
143.01
143.16
-10 0 10 20 30 40 50 60 70 80 90 100 110 120 130 140 150 160 170 180 190 200 210

Synthesis and Characterization of Cycloadducts and Oligophenylenes 10a-c, 11, 12, and 13

Ruthenium catalyzed cycloaddition reactions

A resealable pressure tube (ca. 13 x 100 mm) was charged with [$Ru_3(CO)_{12}$] (2.7 mg, 0.004 mmol, 2 mol%), dppp (4.8 mg, 0.012 mmol, 6 mol%), 3,5-Me_2BzOH (3.0 mg, 0.02 mmol, 10 mol%), polyol (0.2 mmol, 100 mol%). The pressure tube was purged with argon and toluene/dimethylacetamide (1:1, 0.40 mL) was added via syringe, followed by freshly condensed butadiene (0.17 mL, 2.0 mmol, 1000 mol%; or 5 equivalents per diol). The septum was replaced with a screw cap, and the reaction was placed in a 130 ˚C oil bath. After 40 hours, the reaction vessel was removed from the oil bath and allowed to cool to room temperature The volatiles were removed *in vacuo* and the residue was subjected to flash column chromatography (SiO_2) under the conditions noted to afford the desired products.

Dehydration reactions with *p*-toluenesulfonic acid

A resealable pressure tube (ca. 13 x 100 mm) was charged with cycloadduct (0.2 mmol, 100 mol%) followed by *p*-toluenesulfonic acid (3.8 mg, 0.02 mmol, 10 mol%). The pressure tube was purged with argon and toluene (2.9 mL) was added via syringe. The septum was replaced with a screw cap, and the reaction was placed in a 75 ˚C oil bath. After 40 hours, the reaction vessels was removed from the oil bath and allowed to cool to room temperature. The volatiles were removed *in vacuo*, and the residue was subjected to flash column chromatography (SiO_2) under the conditions noted to afford the desired product **10a-c**, **11-13**.

3',3''',6',6'''-tetrahydro-[1,1':2',1'':4'',1''':2''',1'''-quinquephenyl]-1',1''',2',2'''-tetraol (**S6**)

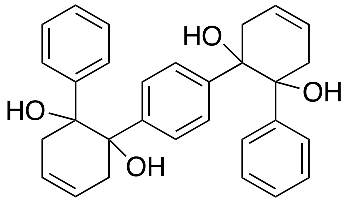

The reaction was conducted with bis diol **3a** in accordance with the general procedure. Flash column chromatography (SiO_2, hexanes:ethyl acetate =80:20 to 50:50) provided the title compound **S6** (64.5 mg, 0.18 mmol) in 82% yield as a white solid.

TLC (SiO_2): R_f = 0.25 (hexanes : ethyl acetate = 50:50).
^{1}H NMR: (400 MHz, $CDCl_3$): δ = 7.24–7.13 (m, 6H), 7.07–7.01 (m, 4H), 6.01–5.93 (m, 4H), 2.93–2.74 (m, 8H), 2.62–2.52 (m, 4H) ppm.
HRMS: (ESI) Calculated for $C_{30}H_{30}O_4$ [M+Na$^+$] = 477.2036, Found 477.2044.
FTIR: (neat): 2365, 1739, 1366, 1217 cm^{-1}.

*Due to the mixture of stereoisomers, ^{13}C data is not reported.

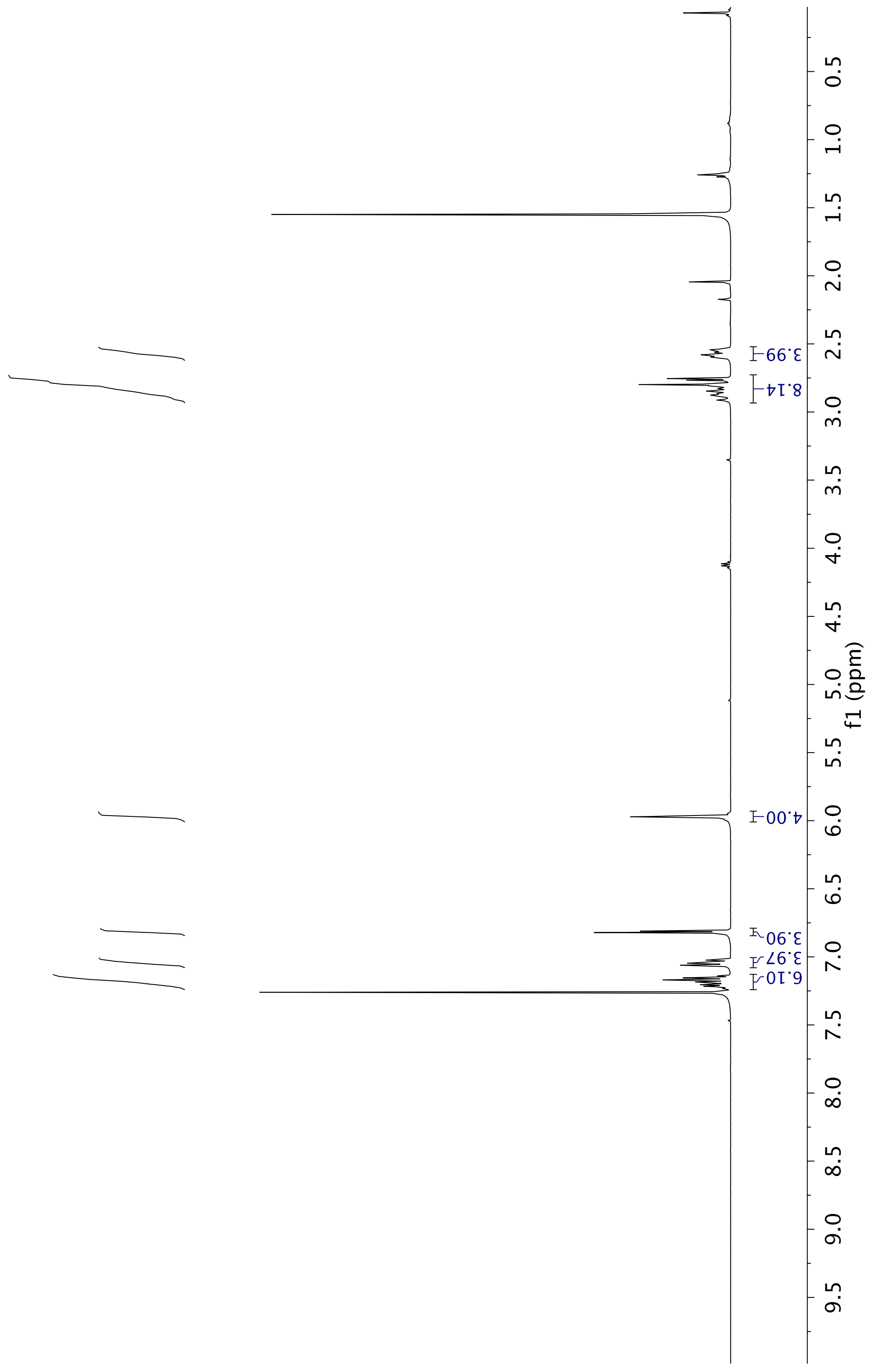
f1 (ppm)
3.99
8.14
4.00
3.90
3.97
6.10

1,1':2',1'':4'',1''':2''',1''''-quinquephenyl (**10a**)

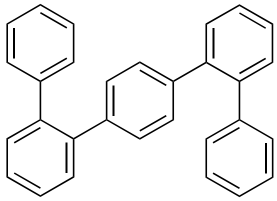

The reaction was conducted with cycloadduct **S6** in accordance with the general procedure. Flash column chromatography (SiO_2, hexanes:DCM = 90:10) provided the title compound **10a** (57.4 mg, 0.14 mmol) in 75% yield as a white solid. The NMR spectra were consistent with a literature report.[vi]

TLC (SiO_2): R_f = 0.30 (hexanes : DCM = 90:10).
^{1}H NMR: (400 MHz, $CDCl_3$): δ = 7.42–7.39 (m, 8H), 7.23–7.20 (m, 6H), 7.14–7.11 (m, 4H), 6.98 (s, 4H) ppm.
^{13}C NMR: (100 MHz, $CDCl_3$): δ = 141.4, 140.5, 140.3, 139.6, 130.5, 130.5, 129.9, 129.4, 127.8, 127.4, 126.4 ppm.
HRMS: (CI) Calculated for $C_{30}H_{22}$ [M^+] = 382.1722, Found 382.1722.
FTIR: (neat): 2922, 2854, 1114, 965 cm^{-1}.

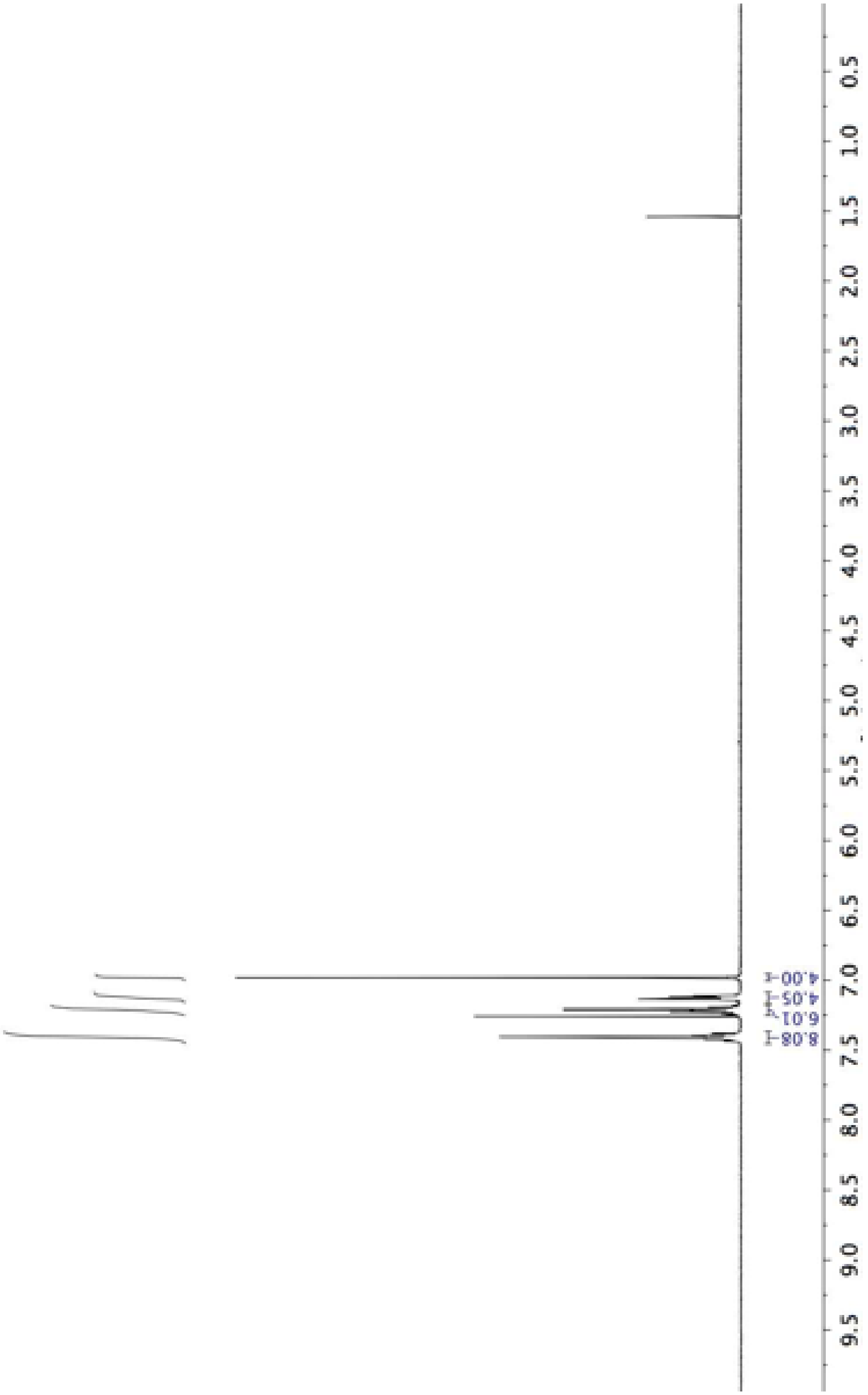

4.00
4.05
6.01
8.08
0.5
1.0
1.5
2.0
2.5
3.0
3.5
4.0
4.5
5.0
5.5
6.0
6.5
7.0
7.5
8.0
8.5
9.0
9.5

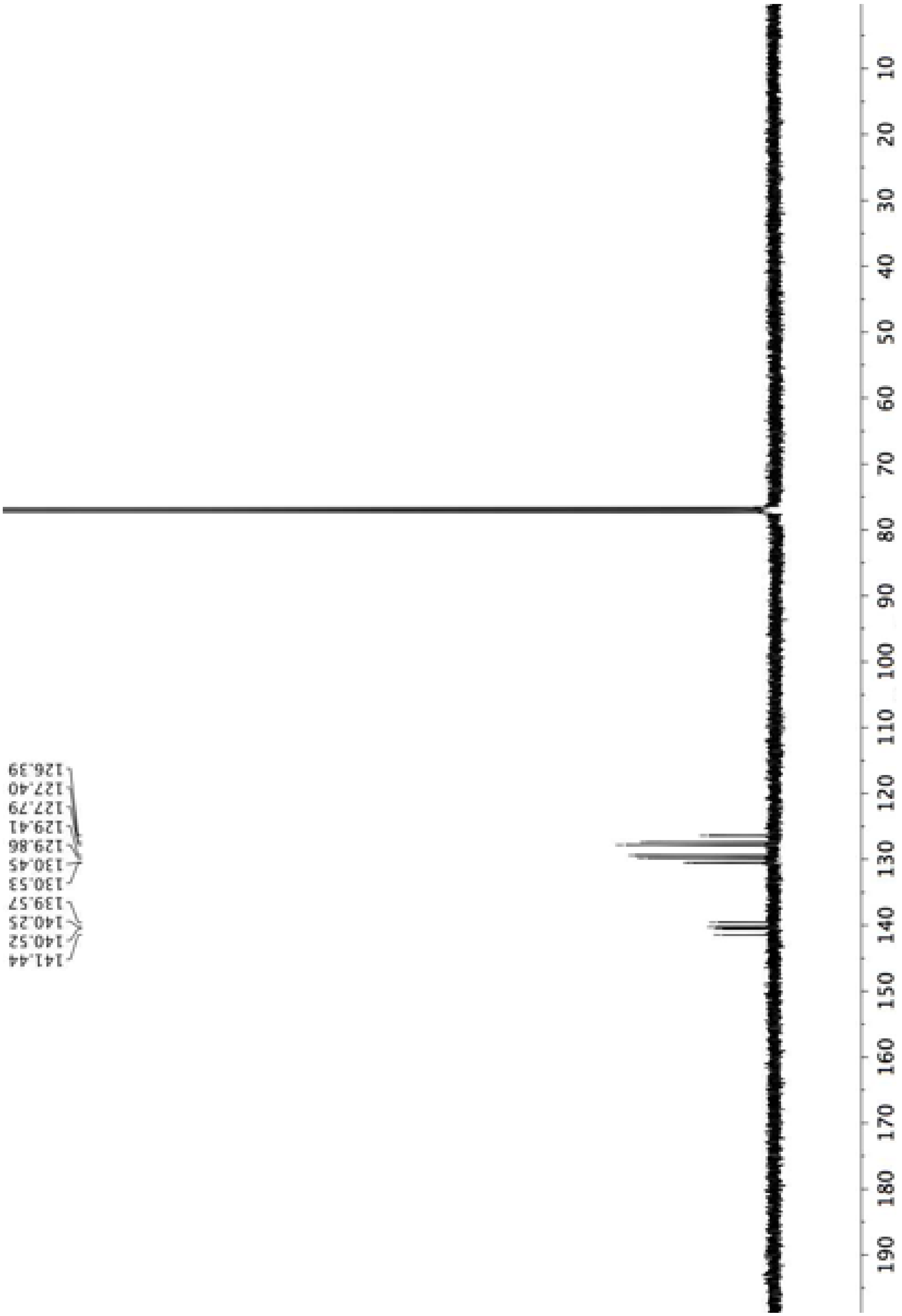
126.39
127.40
127.79
129.41
129.86
130.45
130.53
139.57
140.25
140.52
141.44
10 20 30 40 50 60 70 80 90 100 110 120 130 140 150 160 170 180 190

4,4''''-dibromo-3',3''',6',6'''-tetrahydro-[1,1':2',1'':4'',1''':2''',1''''-quinquephenyl]-1',1''',2',2'''-tetraol (S7)

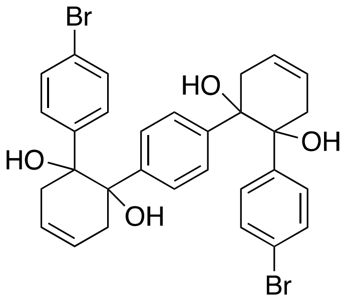

The reaction was conducted with bis diol **3b** in accordance with the general procedure. Flash column chromatography (SiO_2, hexanes:ethyl acetate =80:20 to 50:50) provided the title compound **S7** (107.7 mg, 0.18 mmol) in 88% yield as a white solid.

TLC (SiO_2): R_f = 0.25 (hexanes : ethyl acetate = 50:50).
^{1}H NMR: (400 MHz, $CDCl_3$): δ = 7.30–7.26 (m, 4H), 46.93–6.84 (m, 8H), 6.01–5.94 (m, 4H), 2.87–2.50 (m, 12H) ppm.
HRMS: (ESI) Calculated for $C_{30}H_{28}Br_2O_6$ [M+Na$^+$] =635.0229, Found 635.0237.
FTIR: (neat): 3445, 2918, 763, 719 cm^{-1}.

*Due to the mixture of stereoisomers, ^{13}C data is not reported.

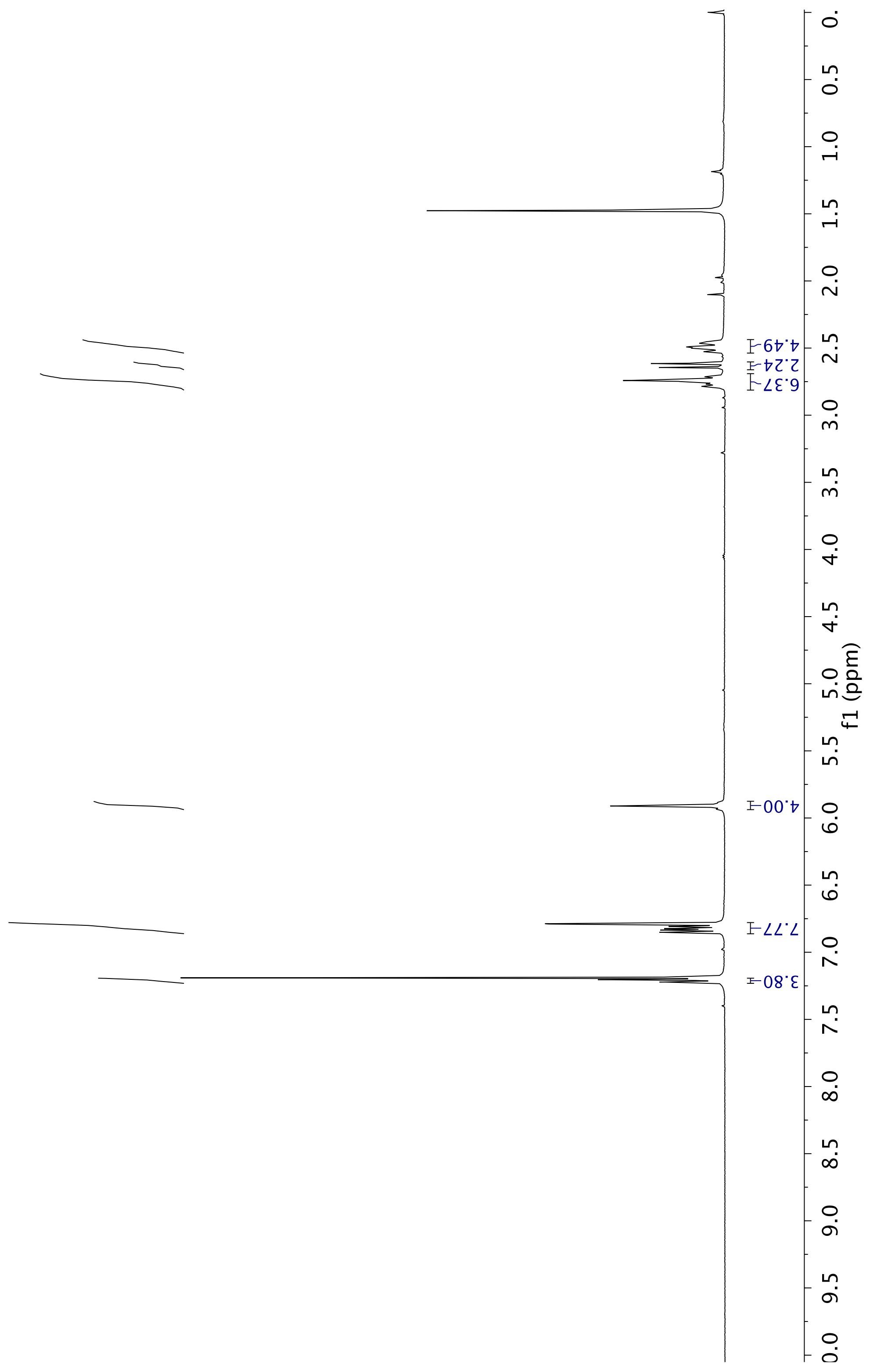
f1 (ppm)
4.49
2.24
6.37
4.00
7.77
3.80

4,4''''-dibromo-1,1':2',1'':4'',1''':2''',1''''-quinquephenyl (**10b**)

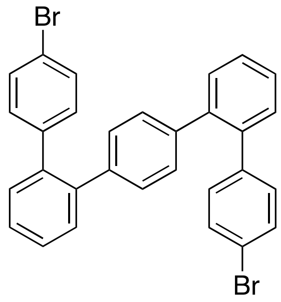

The reaction was conducted with cycloadduct **S7** in accordance with the general procedure. Flash column chromatography (SiO_2, hexanes:DCM = 90:10) provided the title compound **10b** (92.9 mg, 0.17 mmol) in 86% yield as a white solid.

TLC (SiO_2): R_f = 0.36 (hexanes : DCM = 90:10).
^{1}H NMR: (400 MHz, $CDCl_3$): δ = 7.42–7.34 (m, 12H), 7.01–6.99 (m, 8H) ppm.
^{13}C NMR: (100 MHz, $CDCl_3$): δ = 140.4, 140.0, 139.5, 139.2, 131.5, 131.0, 130.6, 130.4, 129.5, 127.8, 127.6, 120.9 ppm.
MP: 232 – 235 °C.
HRMS: (CI^+) Calculated for $C_{30}H_{20}Br_2$ [M^+] = 539.9911, Found 539.9915.
FTIR: (neat): 1468, 1001, 827, 752 cm^{-1}.

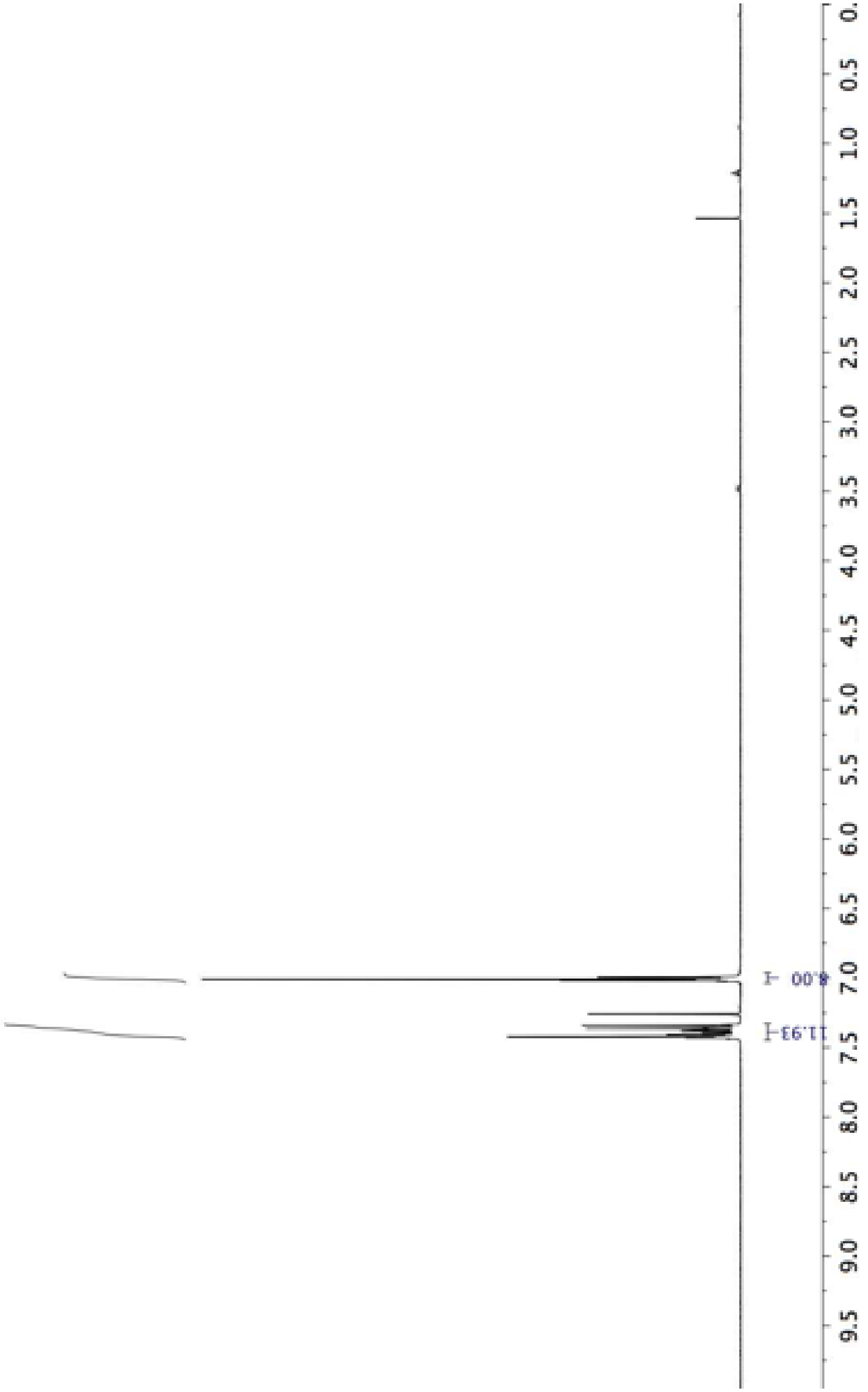

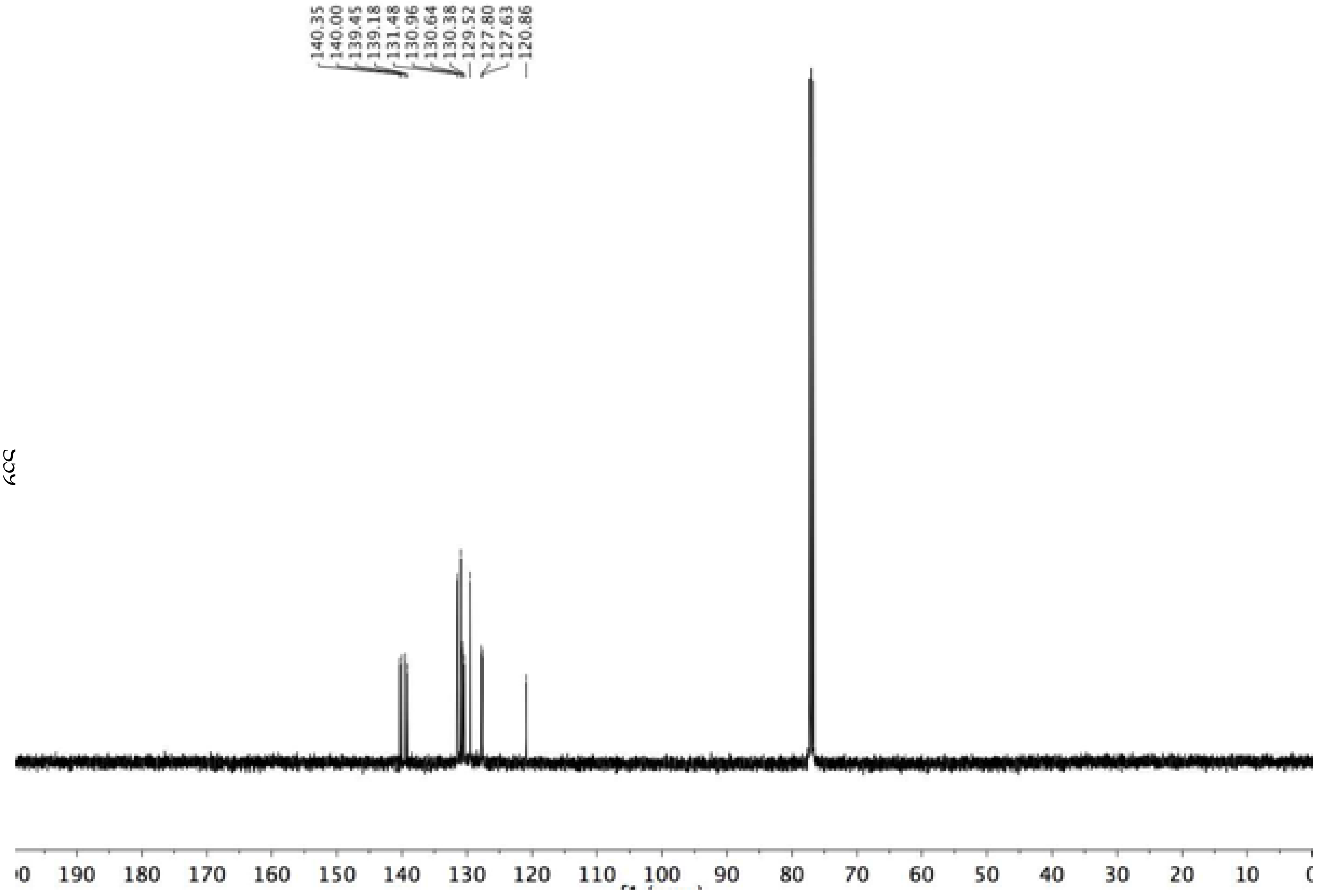

140.35
140.00
139.45
139.18
131.48
130.96
130.64
130.38
129.52
127.80
127.63
120.86
0 190 180 170 160 150 140 130 120 110 100 90 80 70 60 50 40 30 20 10 0

2,2"-bis(benzo[*b*]thiophen-2-yl)-3,3",6,6"-tetrahydro-[1,1':4',1"-terphenyl]-1,1",2,2"(2*H*,2"*H*)-tetraol (**S8**)

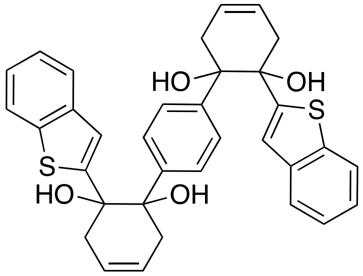

The reaction was conducted with bis diol **3c** in accordance with the general procedure in 2.0 mL toluene/DMA = 1:1 (0.1 M). Flash column chromatography (SiO_2, hexanes:ethyl acetate = 50:50) provided the title compound **S8** (89.5 mg, 0.16 mmol) in 79% yield as a white solid.

TLC (SiO_2): R_f = 0.29 (hexanes : ethyl acetate = 1:2).

^{1}H NMR: (400 MHz, $CDCl_3$, isomer A) δ 7.71 - 7.68 (m, 2H), 7.57 - 7.53 (m, 2H), 7.30 - 7.24 (m, 4H), 7.02 (s, 4H), 6.72 (s, 2H), 5.99 (d, J = 10.2 Hz, 2H), 5.87 (d, J = 10.2 Hz, 2H), 3.03 - 2.63 (m, 12H) ppm.

^{13}C NMR: (100 MHz, $CDCl_3$, isomer A) δ 147.7, 141.1, 139.3, 139.2, 126.7, 125.6, 125.6, 124.7, 124.0, 123.9, 123.3, 121.9, 121.4, 77.2, 76.9, 39.5, 39.4 ppm

^{1}H NMR: (400 MHz, CDCl3, isomer B) δ 7.71 - 7.68 (m, 2H), 7.57 - 7.53 (m, 2H), 7.30 - 7.24 (m, 4H), 7.04 (s, 4H), 6.79 (s, 2H), 5.99 (d, J = 10.2 Hz, 2H), 5.87 (d, J = 10.2 Hz, 2H), 3.03 - 2.63 (m, 12H) ppm.

^{13}C NMR: (100 MHz, $CDCl_3$, isomer B) δ 147.8, 141.2, 139.4, 139.2, 126.8, 125.6, 125.6, 124.6, 124.0, 123.9, 123.3, 122.0, 121.4, 77.2, 76.8, 39.5, 39.4 ppm

MP: 122-148 °C

HRMS: (ESI) Calculated for $C_{34}H_{30}O_4S_2$ [M+Na$^+$] =589.1478, Found 589.1477.

FTIR: (neat) 3497, 3367, 3056, 3025, 2915, 1509, 1434, 1042, 974, 831, 746, 725 cm^{-1}

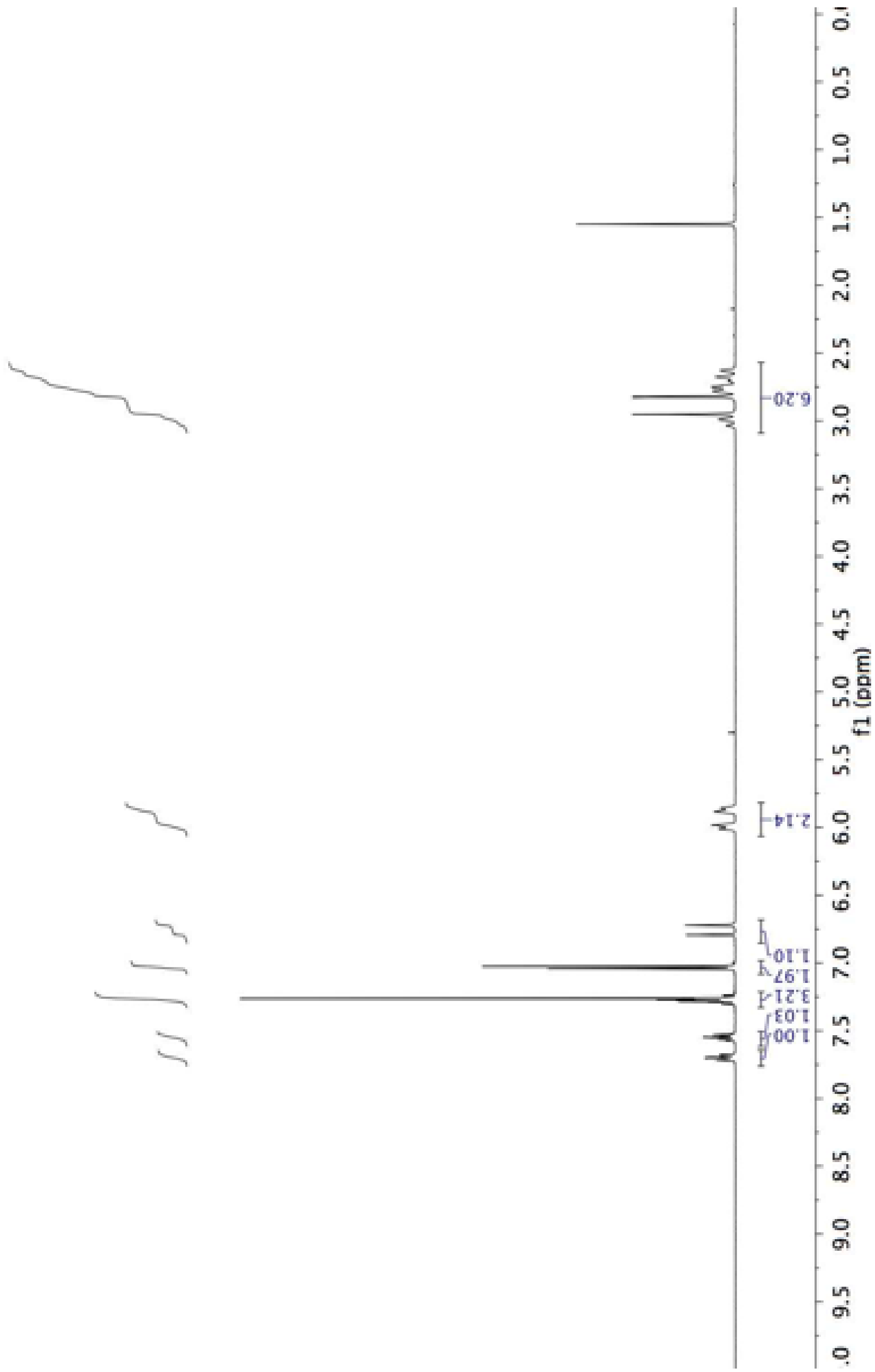
f1 (ppm)

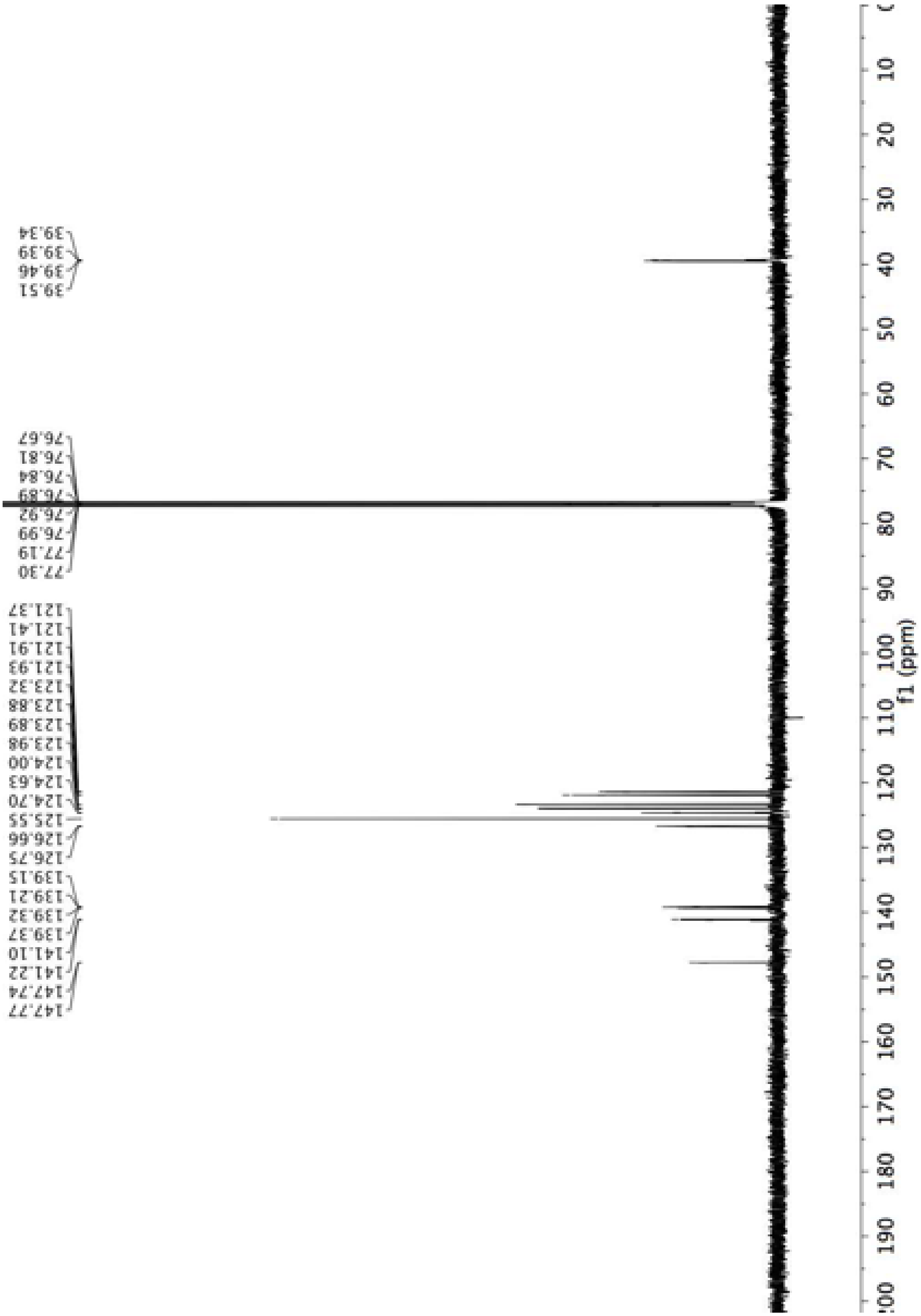

39.34
39.39
39.46
39.51
76.67
76.81
76.84
76.89
76.92
76.99
77.19
77.30
121.37
121.41
121.91
121.93
123.32
123.88
123.89
123.98
124.00
124.63
124.70
125.55
126.66
126.75
139.15
139.21
139.32
139.37
141.10
141.22
147.74
147.77
f1 (ppm)

2,2"-bis(benzo[*b*]thiophen-2-yl)-1,1':4',1"-terphenyl (**10c**)

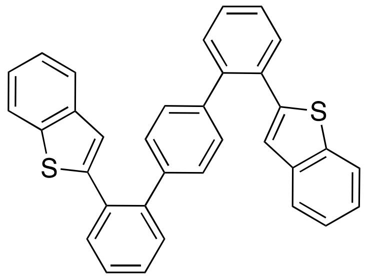

The reaction was conducted with cycloadduct **S8** in accordance with the general procedure. Flash column chromatography (SiO_2, hexanes:DCM = 80:20) provided the title compound **10c** (72.2 mg, 0.15 mmol) in 73% yield as a white solid.

TLC (SiO_2): R_f = 0.79 (hexanes : DCM = 4:1).
^{1}H NMR: (400 MHz, $CDCl_3$): δ 7.71 (d, J = 7.0 Hz, 2H), 7.66 - 7.63 (m, 2H), 7.61 (d, J = 7.0 Hz, 2H), 7.45 - 7.41 (m, 2H), 7.44 (s, 4H), 7.32 - 7.25 (m, 8H), 7.02 (s, 2H) ppm.
^{13}C NMR: δ 143.6, 140.8, 140.4, 140.0, 139.9, 133.1, 131.0, 130.9, 129.4, 129.4, 128.3, 127.6, 124.1, 123.9, 123.6, 123.4, 122.0 ppm.
MP: 197-198 °C
HRMS: (CI^+) Calculated for $C_{34}H_{22}S_2$ [M^+] =494.1163, Found 494.1163.
FTIR: 3053, 2980, 1472, 1432, 1004, 867, 743, 680 cm^{-1}.

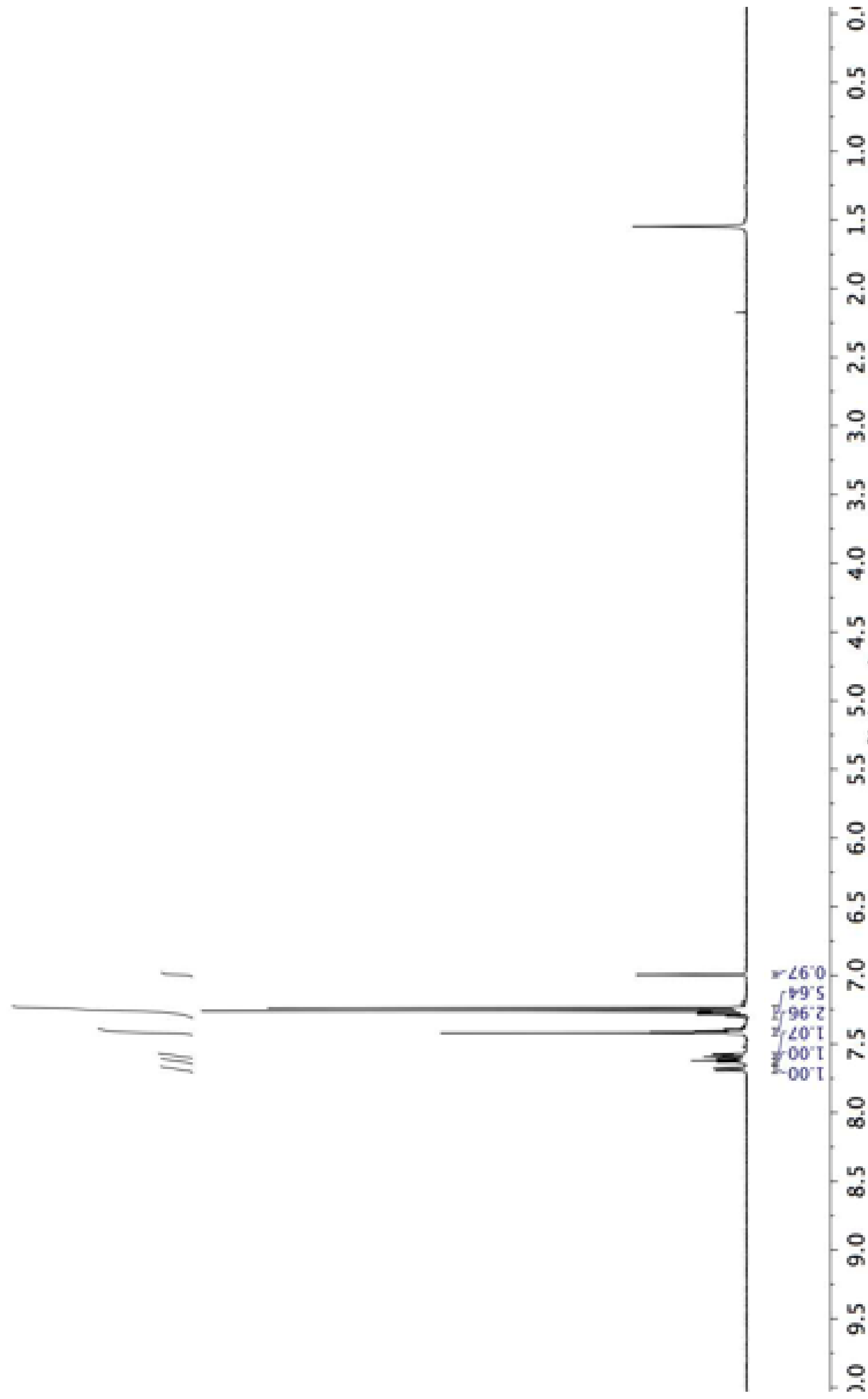
f1 (ppm)

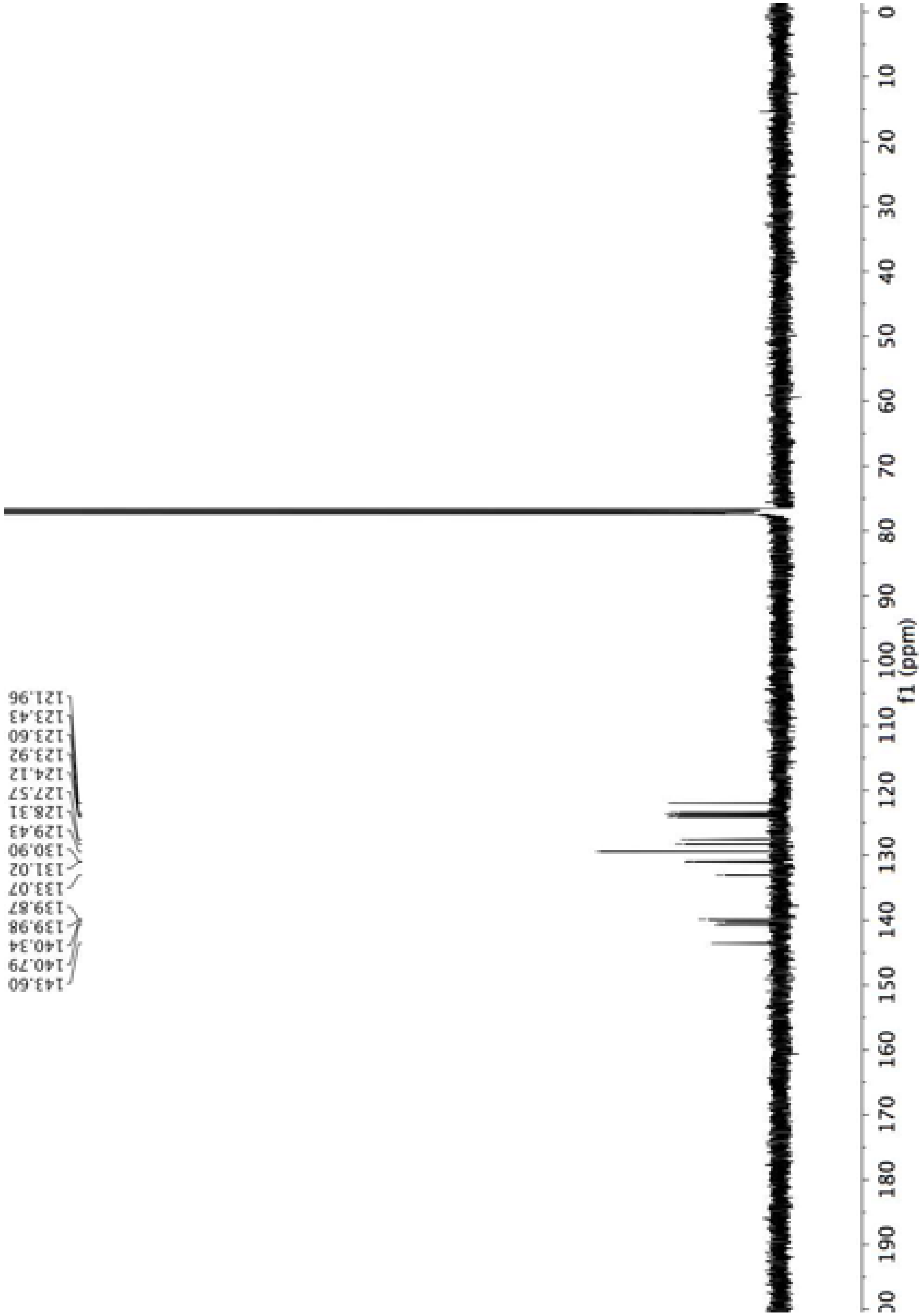
121.96
123.43
123.60
123.92
124.12
127.57
128.31
129.43
130.90
131.02
133.07
139.87
139.98
140.34
140.79
143.60
0
10
20
30
40
50
60
70
80
90
100
110
120
130
140
150
160
170
180
190
f1 (ppm)

4,4'''''-dibromo-3',3''',3''''',6',6''',6'''''-hexahydro-[1,1':2',1'':4'',1''':2''',1'''':4'''',1''''':2''''',1''''''-sepiphenyl]- 1',1''',1''''', 2',2''',2'''''-hexaol (**S9**)

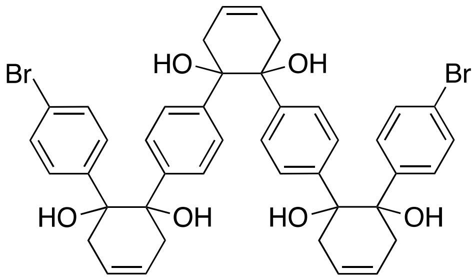

The reaction was conducted with tris diol **7** at 140 ˚C in accordance with the general procedure. Flash column chromatography (SiO_2, hexanes:ethyl acetate =80:20 to 50:50) provided the title compound **S9** (145.7 mg, 0.18 mmol) in 91% yield as a white solid.

TLC (SiO_2): R_f = 0.35 (hexanes : ethyl acetate = 60:40).

^{1}H NMR: (400 MHz, $CDCl_3$): δ = 7.29–7.24 (m, 5H), 6.94–6.79 (m, 11H), 6.02–5.88 (m, 6H), 2.89–2.49 (m, 14H) ppm.

HRMS: (ESI) Calculated for $C_{42}H_{40}Br_2O_6$ [$M+NH_4^+$] =818.1501, Found 818.1514.

FTIR: (neat): 3430, 1365, 1216, 824 cm^{-1}.

*Due to the mixture of stereoisomers, ^{13}C data is not reported.

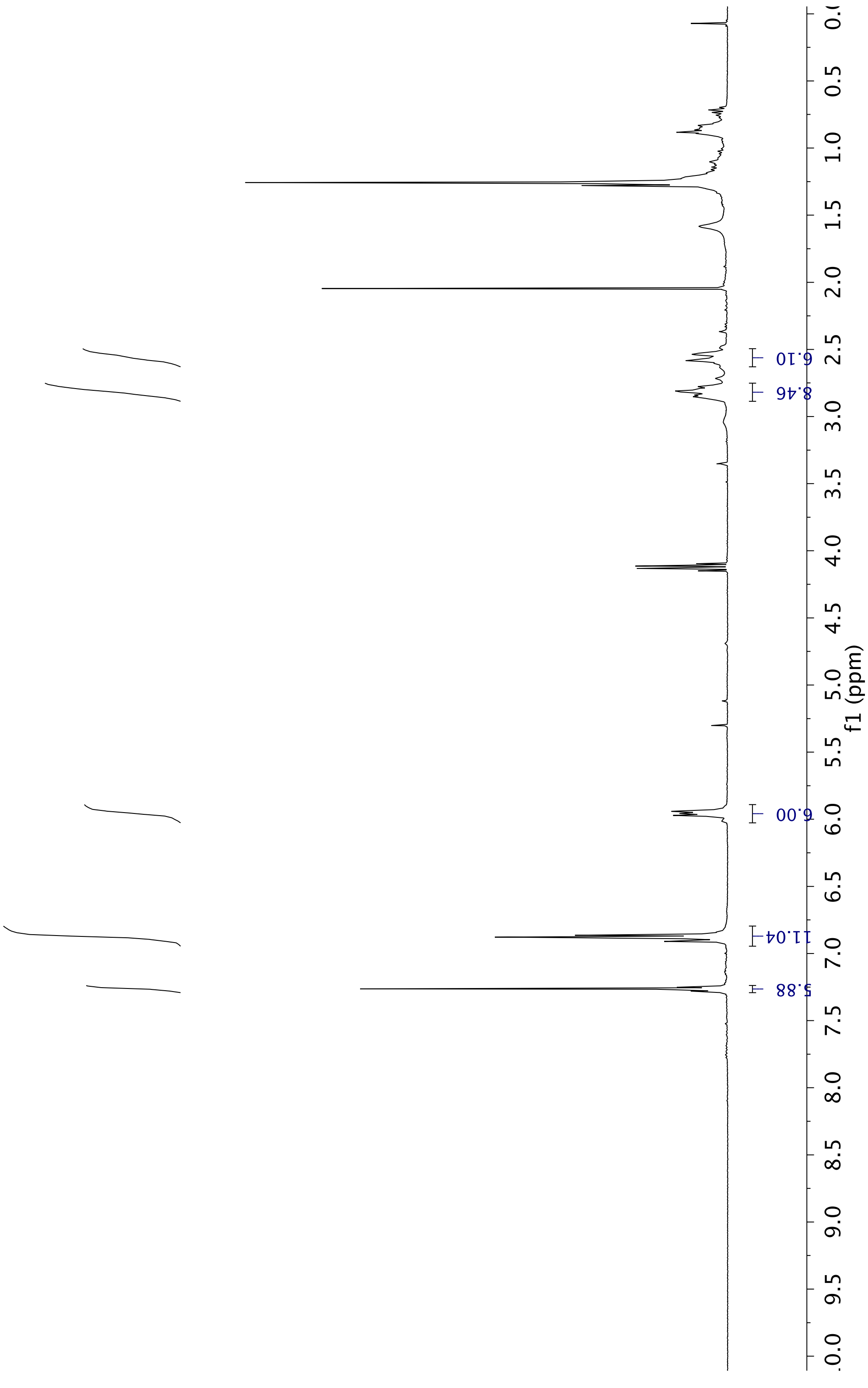
f1 (ppm)
6.10
8.46
6.00
11.04
5.88

4,4''''''-dibromo-1,1':2',1'':4'',1''':2''',1'''':4'''',1''''':2''''',1''''''-sepiphenyl (**11**)

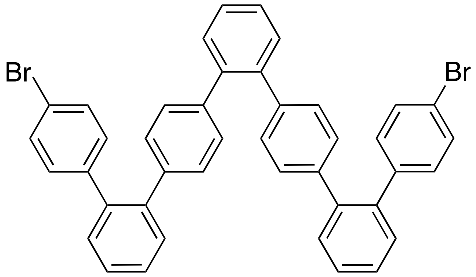

The reaction was conducted with cycloadduct **S9** in accordance with the general procedure. The reacetion was heated to 80 ^oC Flash column chromatography (SiO_2, hexanes:DCM = 90:10) provided the title compound **11** (52.8 mg, 0.14 mmol) in 69% yield as a white solid.

TLC (SiO_2): R_f = 0.32 (hexanes : DCM = 90:10).
^{1}H NMR: (400 MHz, $CDCl_3$): δ = 7.44–7.36 (m, 12H), 7.26–7.24 (q, 4H), 7.03–6.95 (m, 12H) ppm.
^{13}C NMR: (100 MHz, $CDCl_3$): δ = 140.5, 140.2, 139.9, 139.7, 139.5, 139.3, 131.6, 130.9, 130.7, 130.7, 130.4, 129.6, 129.4, 127.8, 127.6, 127.6, 120.8 ppm.
MP: >250 °C.
HRMS: (CI^+) Calculated for $C_{42}H_{28}Br_2$ [$M+H^+$] = 692.0537, Found 692.0538.
FTIR: (neat): 1470, 1071, 824, 750 cm^{-1}.

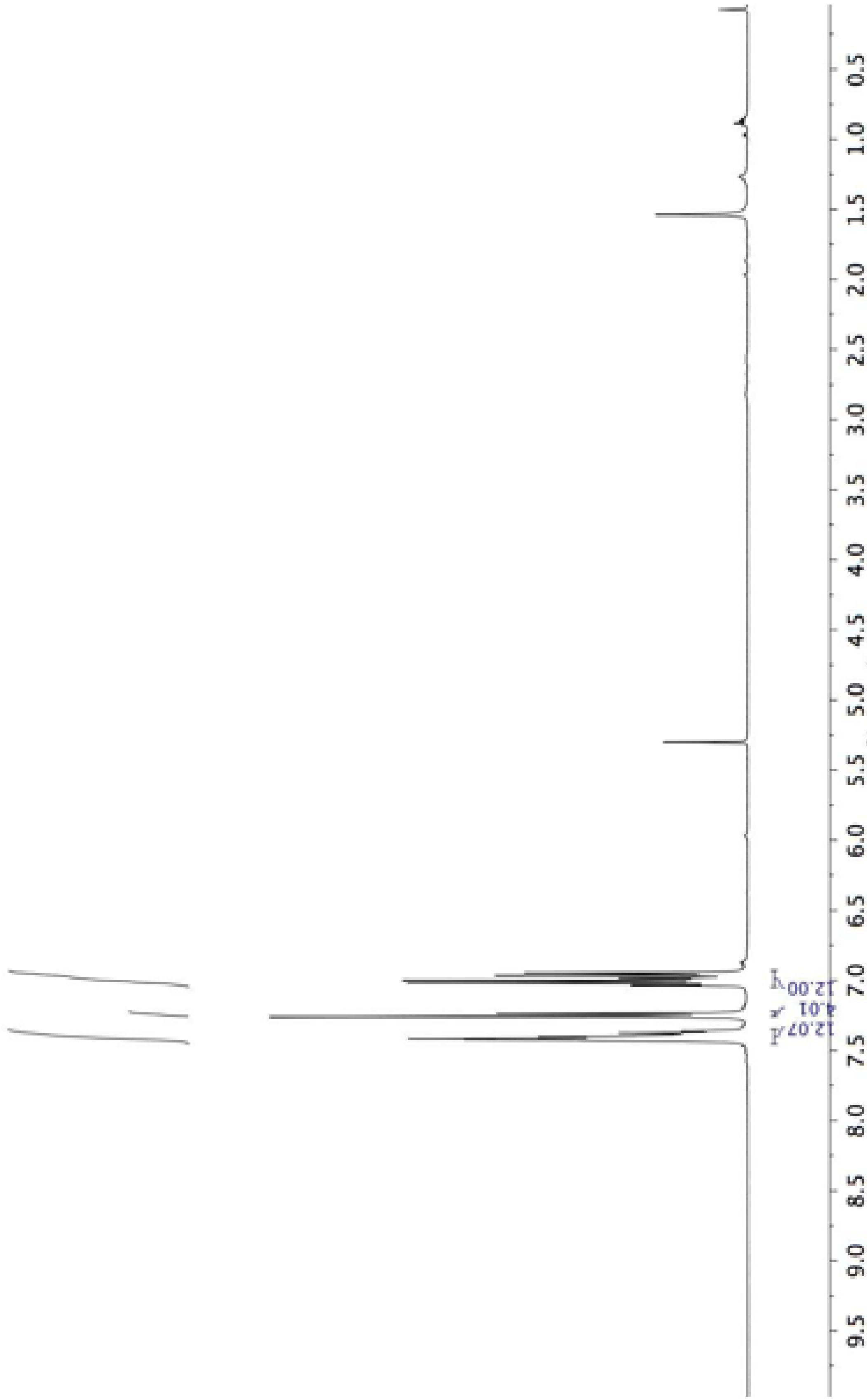
0.5
1.0
1.5
2.0
2.5
3.0
3.5
4.0
4.5
5.0
5.5
6.0
6.5
7.0
7.5
8.0
8.5
9.0
9.5
12.00
4.01
12.07

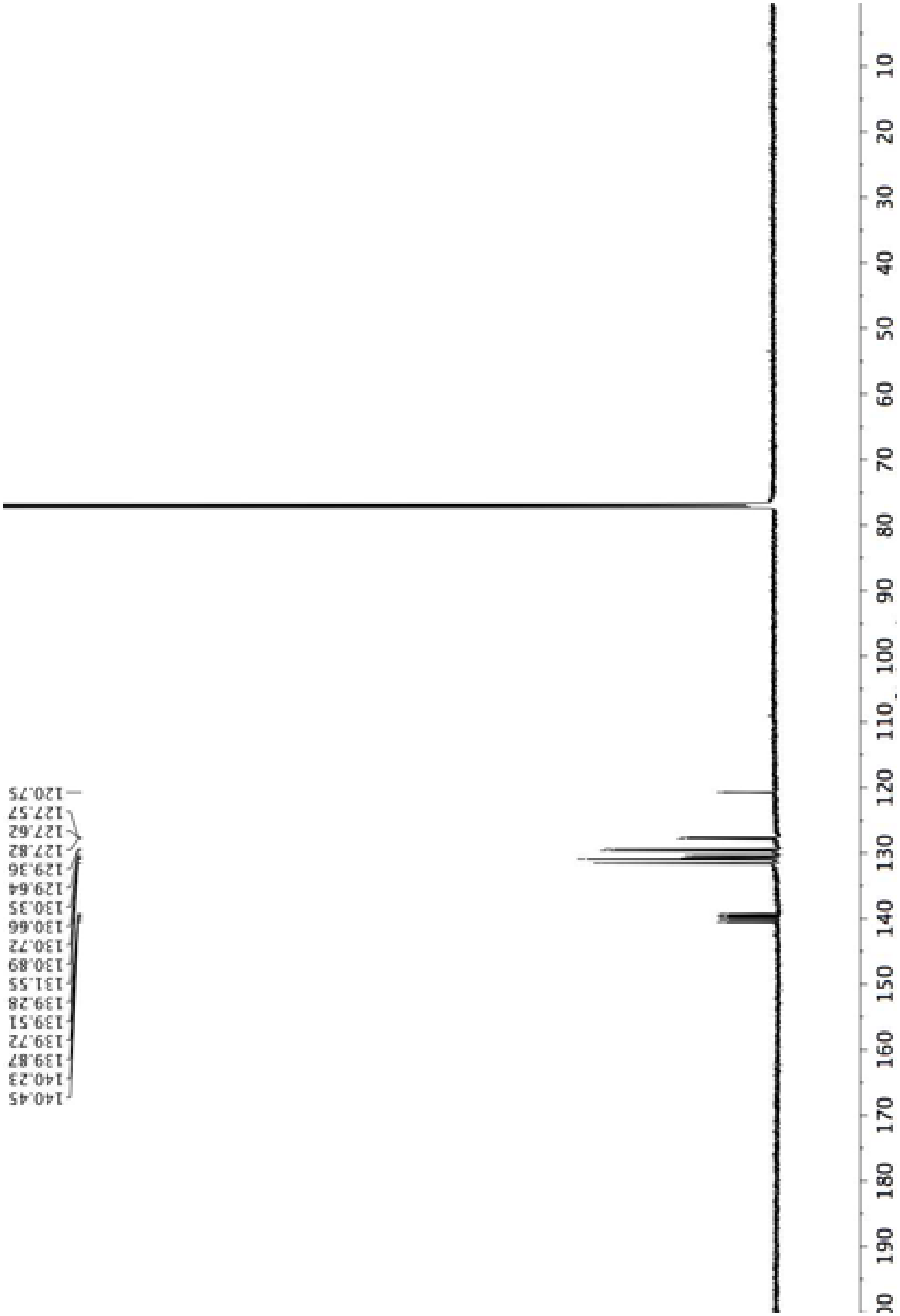
120.75
127.57
127.62
127.82
129.36
129.64
130.35
130.66
130.72
130.89
131.55
139.28
139.51
139.72
139.87
140.23
140.45
10
20
30
40
50
60
70
80
90
100
110
120
130
140
150
160
170
180
190

__4,4''''''''-dibromo-3',3''',3''''',3''''''',6',6''',6''''',6'''''''-octahydro-[1,1':2',1'':4'',1''':2''',1'''':4'''',1''''':2''''',1'''''':4'''''',1''''''':2''''''',1''''''''-novemphenyl]-1',1''',1''''',1''''''', 2',2''',2''''',2'''''''-octaol (S10)__

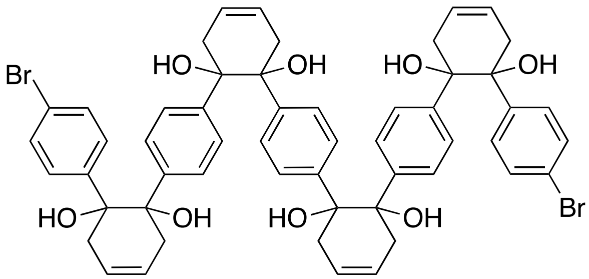

The reaction was conducted with tetra(bis-diol) **8** in accordance with the general procedure, utilizing 39 mg, 0.05 mmol of **8**. Flash column chromatography (SiO_2, CH_2Cl_2: ethyl acetate = 50:50) provided the title compound **S10** (32 mg, 0.33 mmol) in 65% yield as an ecru solid. Some minor impurities (<5%) were inseparable.

__TLC (SiO_2)__: R_f = 0.30 , 0.37 (CH_2Cl_2 : ethyl acetate = 1:1).

__^{1}H NMR__: (400 MHz, $CDCl_3$, mixture of isomers): δ = 7.68–7.21 (m, 6H), 7.11-6.97 (m, 2H), 6.95-6.80 (m, 12H), 5.95 (br s, 6H), 5.60 (t, *J* = 1.4 Hz, 1H), 5.44-5.33 (m, 1H), 3.35 (s, 1H), 2.91-2.71 (m, 9H), 2.67-2.43 (m, 5H), 2.32 (d, *J* = 16.5 Hz, 1H) 2.19 (d, *J* = 16.4 Hz, 1H), 2.12 – 1.73 (m, 5H), 1.58 (s, 1H), 1.25 (s, 1H) ppm.

__HRMS__: (ESI^+) Calculated for $C_{54}H_{52}Br_2O_8$ [M+Na^+] =1009.1921, Found 1009.1928.

__FTIR__: (neat): 3459, 2900, 1056, 763, 750 cm^{-1}.

*Due to the mixture of stereoisomers, ^{13}C data is not reported.

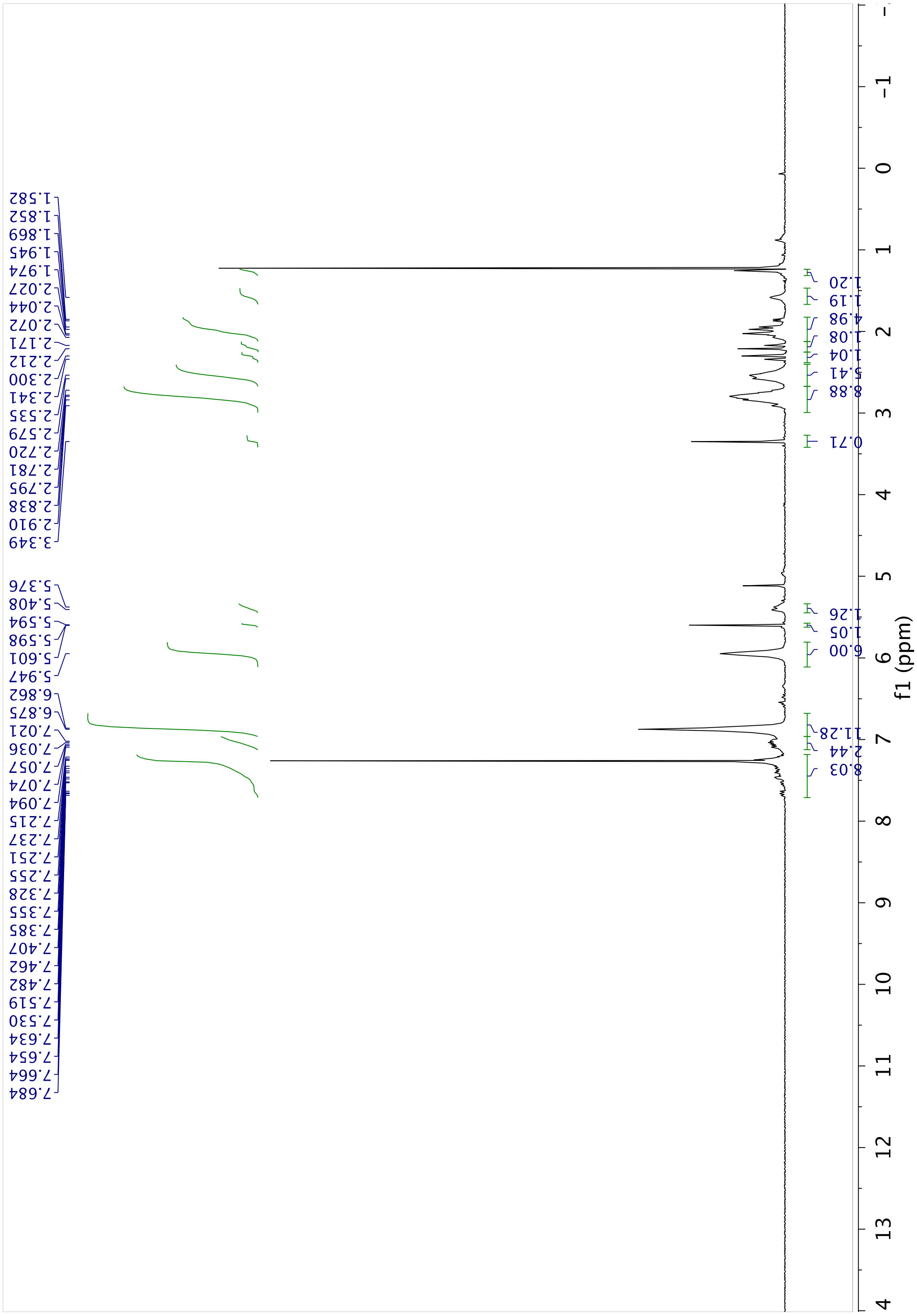
1.582
1.852
1.869
1.945
1.974
2.027
2.044
2.072
2.171
2.212
2.300
2.341
2.535
2.579
2.720
2.781
2.795
2.838
2.910
3.349
5.376
5.408
5.594
5.598
5.601
5.947
6.862
6.875
7.021
7.036
7.057
7.074
7.094
7.215
7.237
7.251
7.255
7.328
7.355
7.385
7.407
7.462
7.482
7.519
7.530
7.634
7.654
7.664
7.684
1.20
1.19
4.98
1.08
1.04
5.41
8.88
0.71
1.26
1.05
6.00
11.28
2.44
8.03
f1 (ppm)

4,4''''''''-dibromo-1,1':2',1'':4'',1''':2''',1'''':4'''',1''''':2''''',1'''''':4'''''',1''''''':2''''''',1''''''''-novemphenyl (**12**)

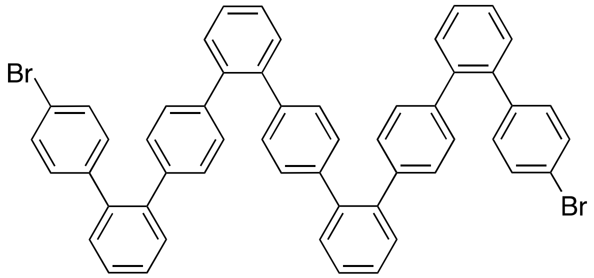

The reaction was conducted in accordance with the general procedure, using **S10** (35 mg, 0.035 mmol). The reaction was heated to 85 ºC. Flash column chromatography (SiO_2, hexanes:ethyl acetate = 95:5) provided the title compound **12** (15 mg, 0.018 mmol) in 51% yield as a colorless solid.

TLC (SiO_2): R_f = 0.60 (hexanes : ethyl acetate = 95:5).

^{1}H NMR: (500 MHz, $CDCl_3$): δ = 7.69 (s, 2H), 7.66 (dd, *J* = 8.0, 1.0 Hz, 2H), 7.53-7.30 (m, 20H), 7.25-7,15 (m, 4H), 7.09-6.88 (m, 8H) ppm.

^{13}C NMR: (125 MHz, $CDCl_3$): δ = 141.7, 140.7, 140.1, 139.7, 139.1, 131.5, 130.9, 130.6, 130.3, 129.7, 129.5, 129, 129.4, 129.0, 128.8, 128.7, 127.5, 127.3, 127.0, 126.9, 126.41, 126.37, 120.7 ppm.

HRMS: (CI^+) Calculated for $C_{54}H_{36}$ [M^+] =.842.1184, Found 842.1189.

FTIR: (neat): 2985, 1498, 999, 878, 733 cm^{-1}.

MP: >250 °C.

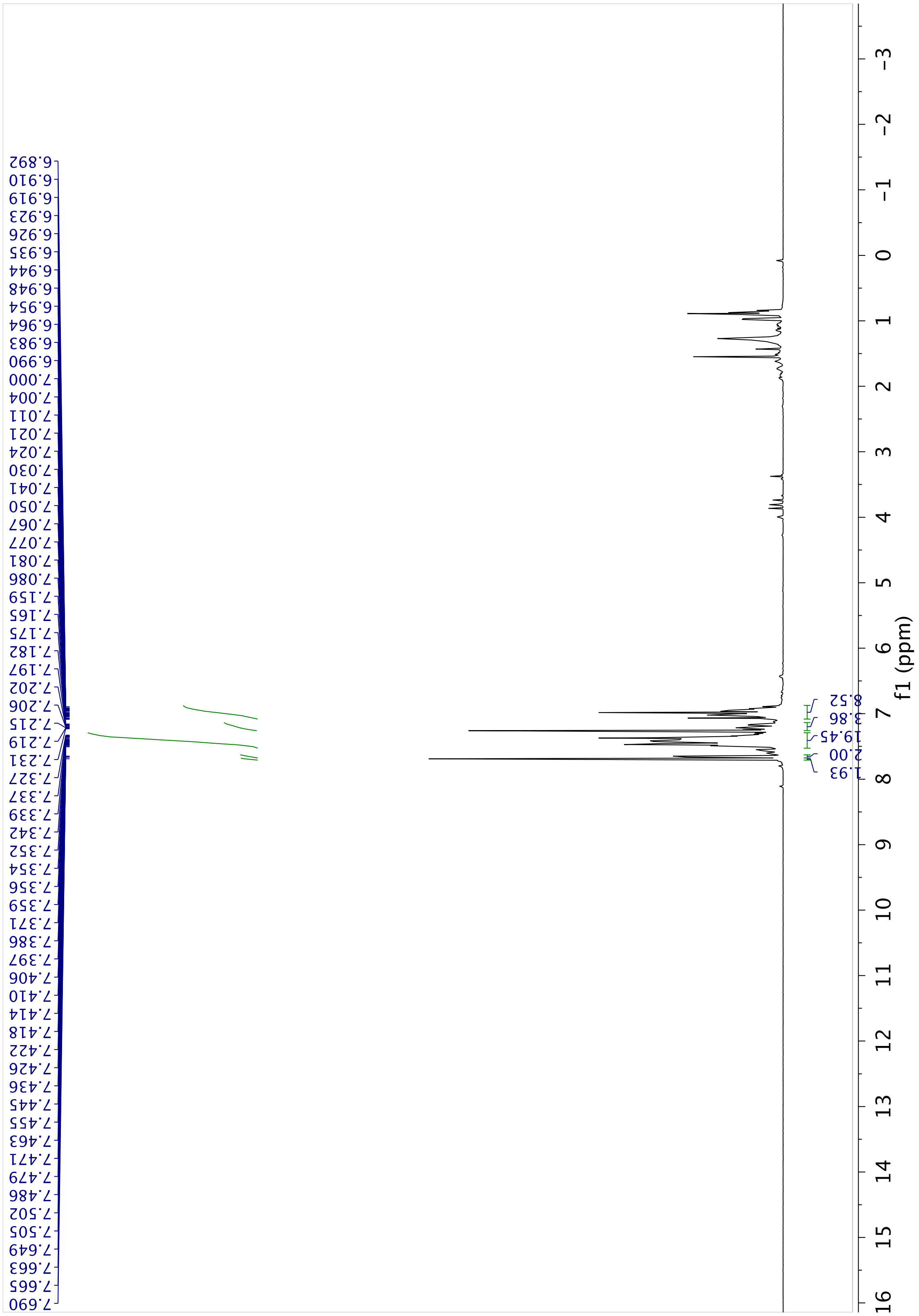
6.892
6.910
6.919
6.923
6.926
6.935
6.944
6.948
6.954
6.964
6.983
6.990
7.000
7.004
7.011
7.021
7.024
7.030
7.041
7.050
7.067
7.077
7.081
7.086
7.159
7.165
7.175
7.182
7.197
7.202
7.206
7.215
7.219
7.231
7.327
7.337
7.339
7.342
7.352
7.354
7.356
7.359
7.371
7.386
7.397
7.406
7.410
7.414
7.418
7.422
7.426
7.436
7.445
7.455
7.463
7.471
7.479
7.486
7.502
7.505
7.649
7.663
7.665
7.690
8.52
3.86
19.45
2.00
1.93
f1 (ppm)

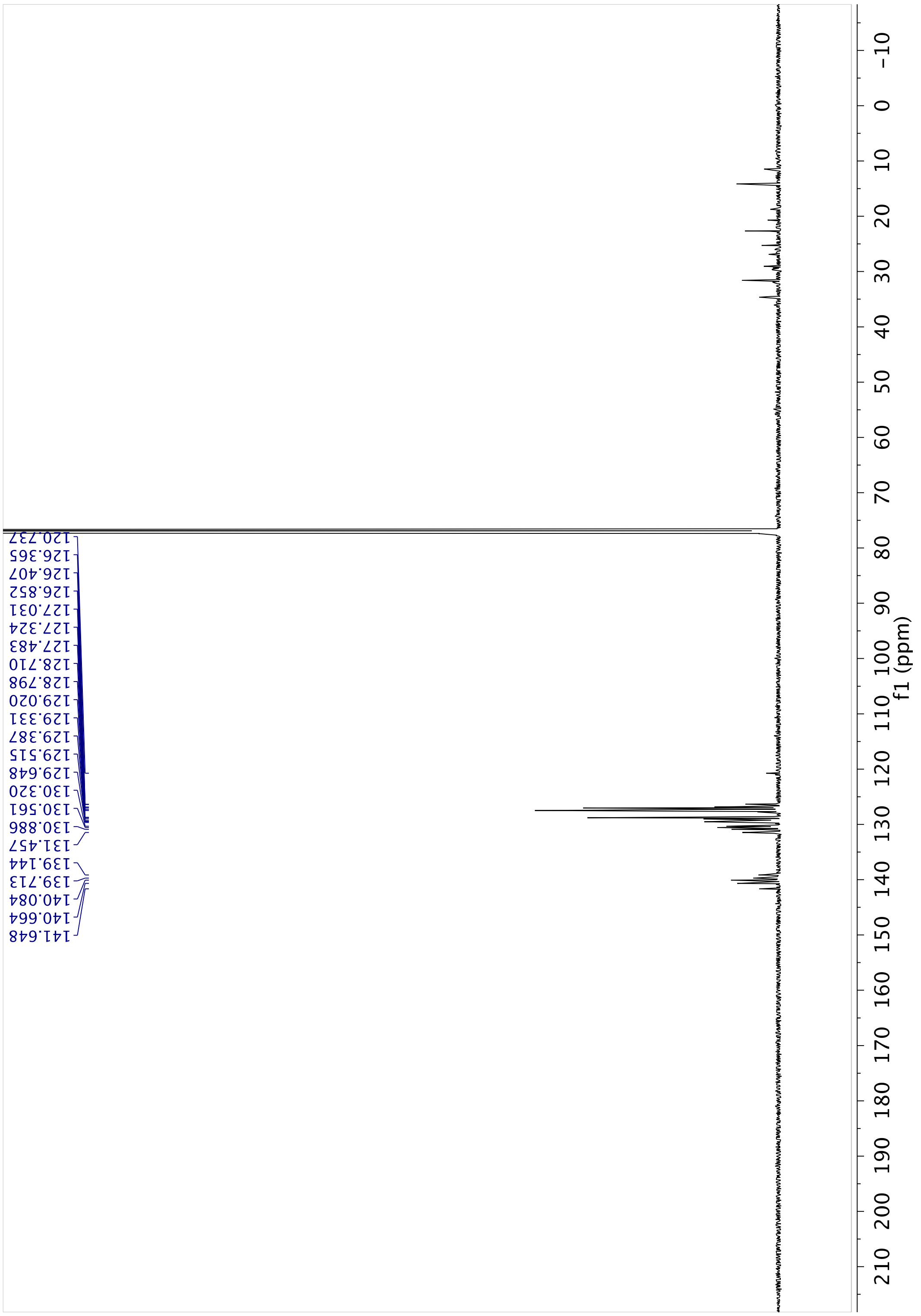

Cycloadduct **S11**

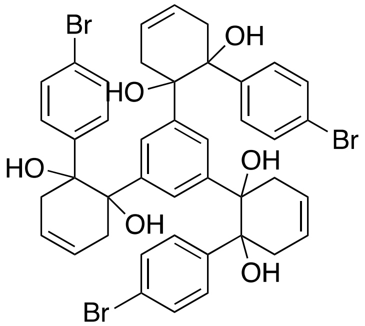

The reaction was conducted with tris diol **9** at 140 ˚C in accordance with the general procedure. Flash column chromatography (SiO_2, hexanes:ethyl acetate =80:30 to 45:55) provided the title compound **S11** (142.5 mg, 0.16 mmol) in 81% yield as a white solid.

TLC (SiO_2): R_f = 0.20 (hexanes : ethyl acetate = 50:50).
^{1}H NMR: (400 MHz, $CDCl_3$): δ = 7.28 (d, *J* = 8.2 Hz, 7H), 6.89–6.82 (m, 6H), 6.67 (s, 2H), 2.71–2.28 (m, 18H) ppm.
HRMS: (ESI) Calculated for $C_{42}H_{39}Br_3O_6$ [M+Na$^+$] = 903.0157, Found 903.0168.
FTIR: (neat): 1738, 1365, 1217 cm^{-1}.

*Due to the mixture of stereoisomers, ^{13}C data is not reported.

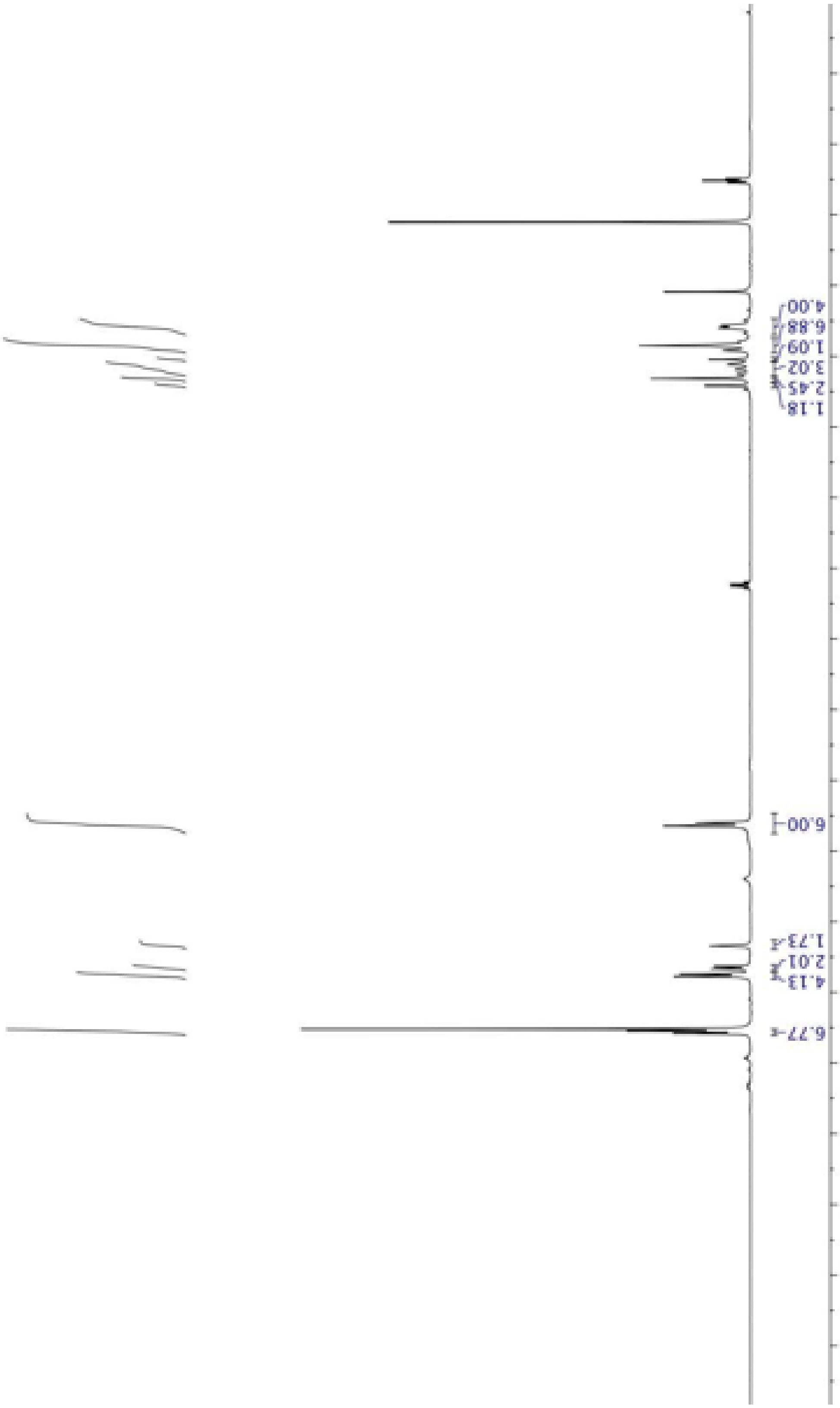

0.5
1.0
1.5
2.0
2.5
3.0
3.5
4.0
4.5
5.0
5.5
6.0
6.5
7.0
7.5
8.0
8.5
9.0
9.5
4.00
6.88
1.09
3.02
2.45
1.18
6.00
1.73
2.01
4.13
6.77

4,4''''-dibromo-5''-(4'-bromo-[1,1'-biphenyl]-2-yl)-1,1':2',1'':3'',1''':2''',1''''-quinquephenyl (**13**)

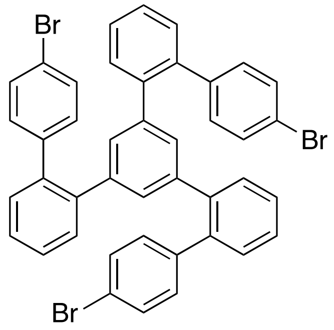

The reaction was conducted with cycloadduct **S11** in accordance with the general procedure. Flash column chromatography (SiO_2, hexanes:DCM = 90:10) provided the title compound **13** (138.9 mg, 0.18 mmol) in 90% yield as a white solid.

TLC (SiO_2): R_f = 0.28 (hexanes : DCM = 90:10).

^{1}H NMR: (400 MHz, $CDCl_3$): δ = 7.43 (d, *J* = 7.9 Hz, 6H), 7.36–7.32 (m, 9H), 6.89–6.85 (m, 9H), 6.68 (s, 3H) ppm.

^{13}C NMR: (100 MHz, $CDCl_3$): δ = 140.7. 140.6, 140.1, 139.3, 131.7, 131.0, 130.5, 130.0, 129.8, 127.8, 127.6, 120.8 ppm.

HRMS: (CI^+) Calculated for $C_{42}H_{27}Br_3$ [M^+] = 769.9642, Found 769.9670.

FTIR: (neat): 2362, 2342, 1739, 1366, 1217 cm^{-1}.

MP: >250 °C.

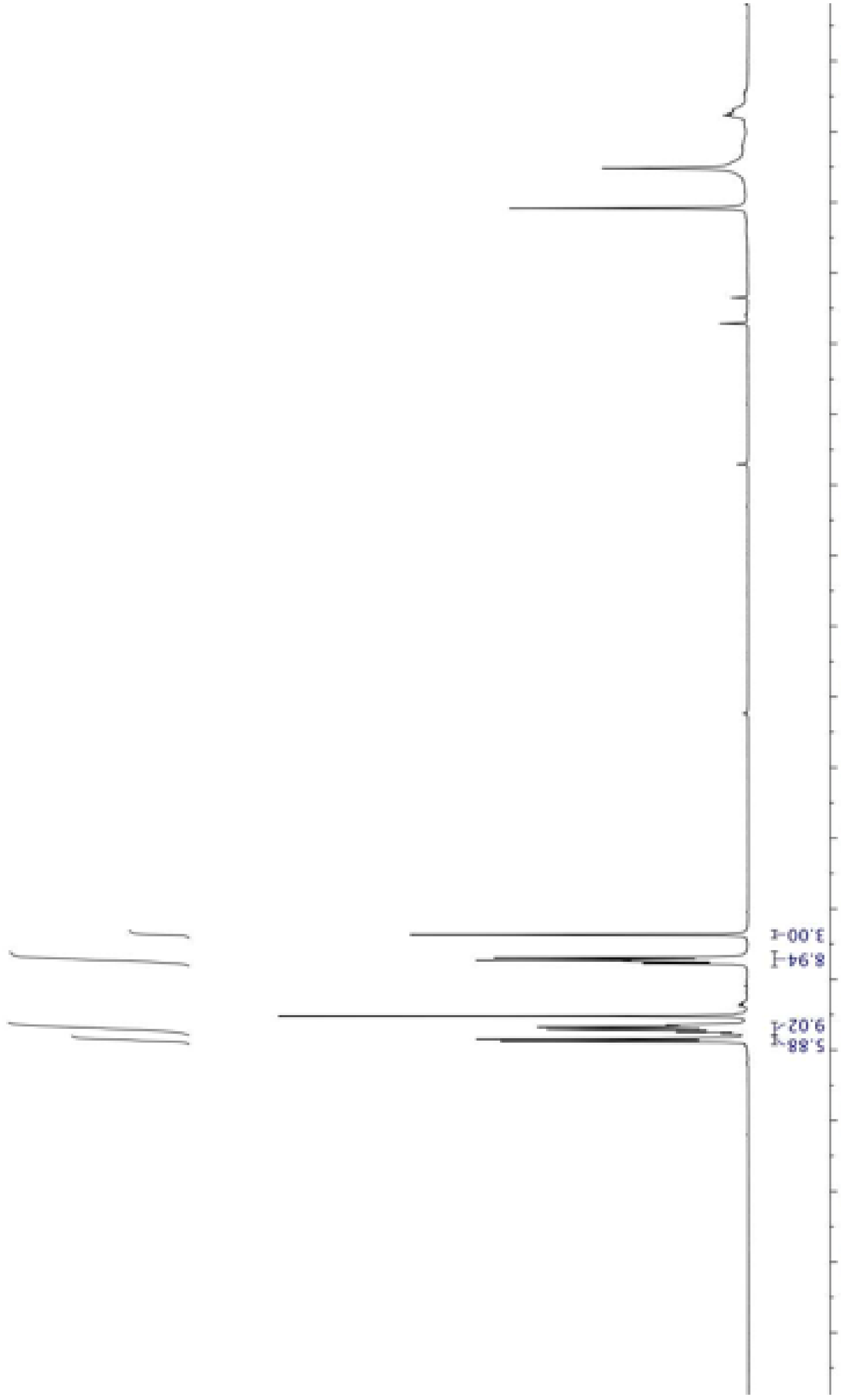
0.5
1.0
1.5
2.0
2.5
3.0
3.5
4.0
4.5
5.0
5.5
6.0
6.5
7.0
7.5
8.0
8.5
9.0
9.5
3.00
8.94
9.02
5.88

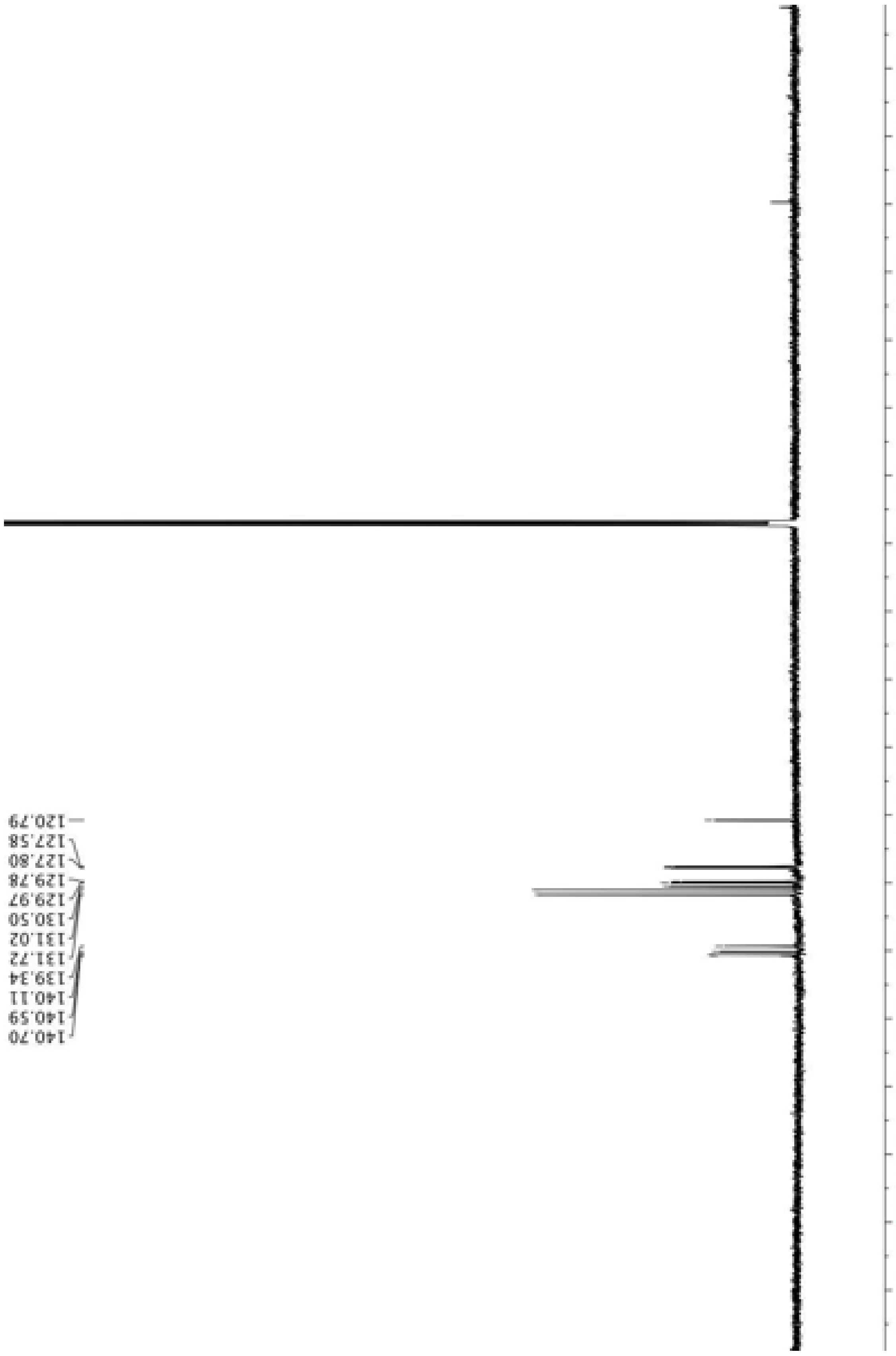
120.79
127.58
127.80
129.78
129.97
130.50
131.02
131.72
139.34
140.11
140.59
140.70
10
20
30
40
50
60
70
80
90
100
110
120
130
140
150
160
170
180
190

Synthesis and Characterization of Helicene 14

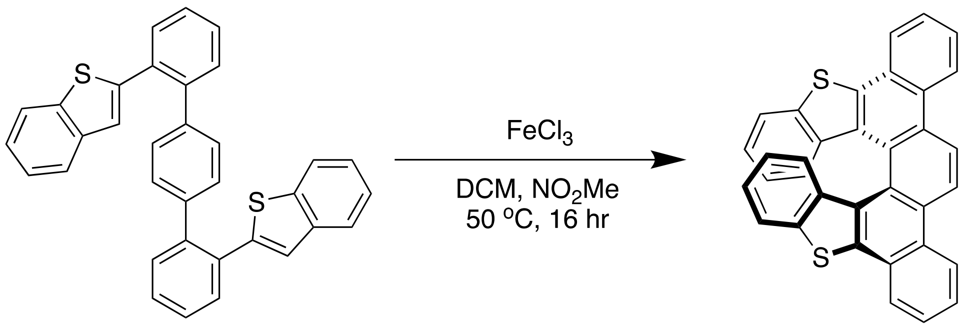

piceno[5,6-*b*:8,7-*b*′]dibenzothiophene (**14**)

To a resealable pressure tube (c.a. 13×1000) was added **10c** (37.1 mg, 0.075 mmol, 100 mol%). Dichloromethane (3 mL) was added followed by $FeCl_3$ (121.7 mg, 0.75 mmol, 1000 mol%) in nitromethane (3 mL). The tube was placed in 50 °C oil bath. When judged complete by TLC (16 h), the tube was removed from the oil bath and allowed to cool to room temperature. Then methanol (3 mL) was added to the reaction mixture. The volatiles were removed in vacuo, and the residue was subjected to flash column chromatography (SiO_2: hexanes/DCM = 4:1) to provide the title compound **14** (20.2 mg, 0.041 mmol) as a white solid in 55% yield.

TLC (SiO_2): R_f = 0.79 (hexanes : DCM = 4:1).

^{1}H NMR: (400 MHz, $CDCl_3$): δ 8.96 (s, 2H), 8.91 - 8.82 (m, 2H), 8.38 - 8.36 (m, 2H), 7.85 - 7.78 (m, 4H), 7.70 (d, *J* = 8.4 Hz, 2H), 7.18 (d, *J* = 8.6 Hz, 2H), 7.02 (dd, *J* = 8.6, 7.2 Hz, 2H), 6.68 (dd, *J* = 8.6, 7.2 Hz, 2H) ppm.

^{13}C NMR: δ 137.9, 137.7, 136.8, 130.9, 129.4, 129.3, 128.7, 127.5, 127.4, 125.1, 124.9, 124.2, 123.6, 123.4, 123.0, 121.9, 121.0 ppm.

HRMS: (ESI) Calculated for $C_{34}H_{18}S_2$ [M^+] = 490.0850, Found 490.0849.

FTIR: (neat) 3056, 2925, 2852, 1472, 1446, 1261, 1071, 1025, 936, 762, 748, 730 cm^{-1}

MP: 354 °C (sublimed)

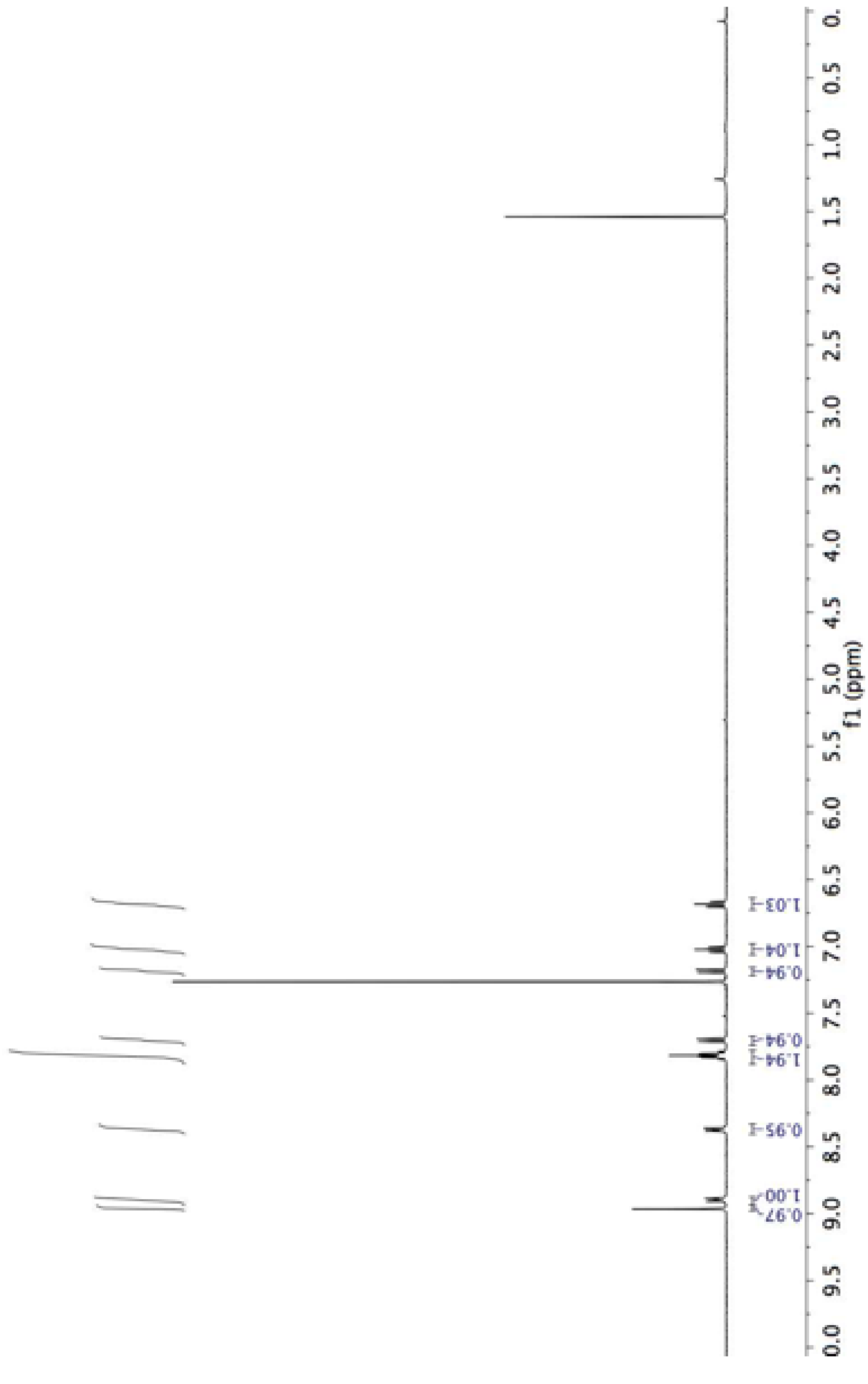

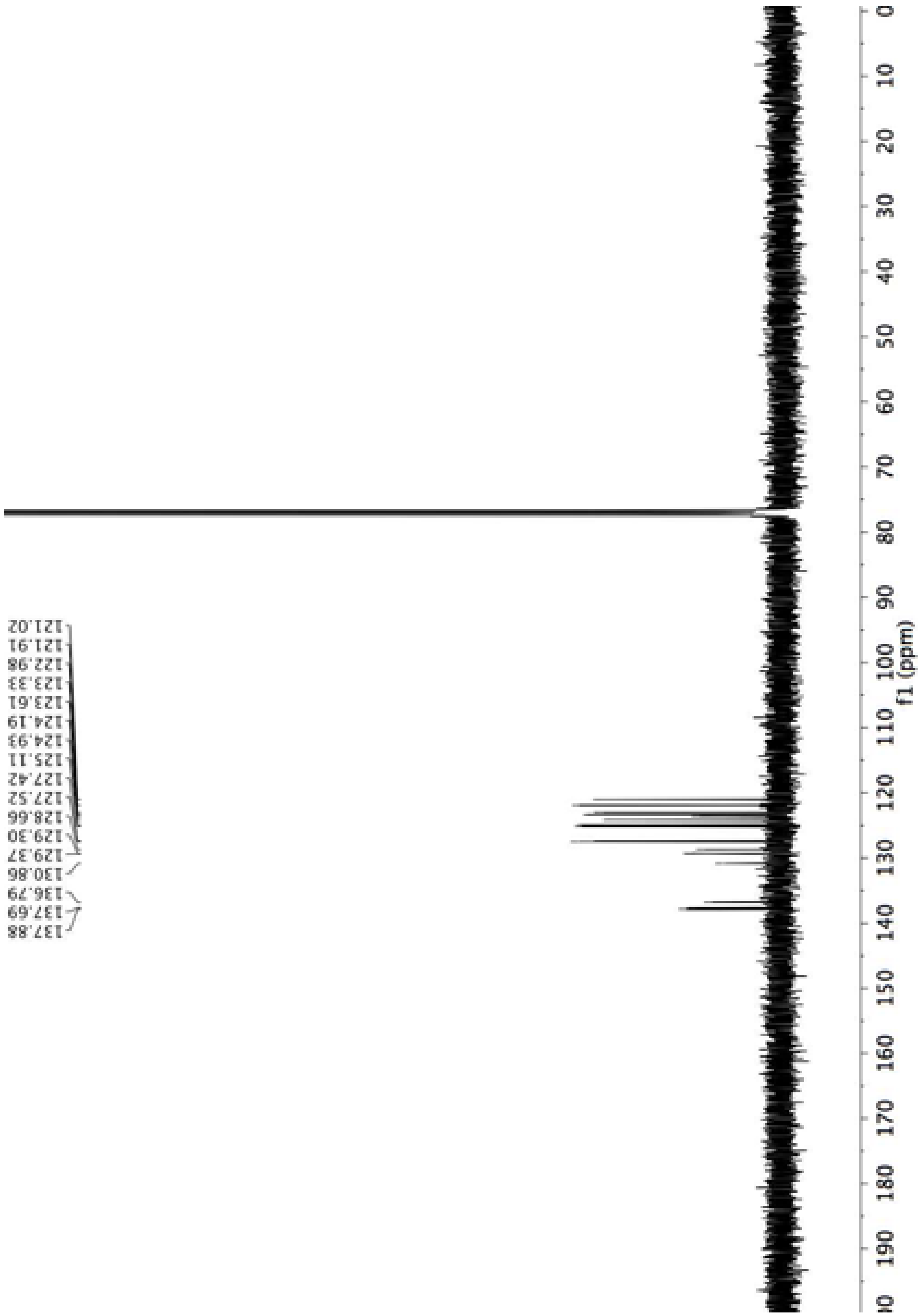
121.02
121.91
122.98
123.33
123.61
124.19
124.93
125.11
127.42
127.52
128.66
129.30
129.37
130.86
136.79
137.69
137.88
f1 (ppm)

Synthesis and Characterization of HBC 17 and 18

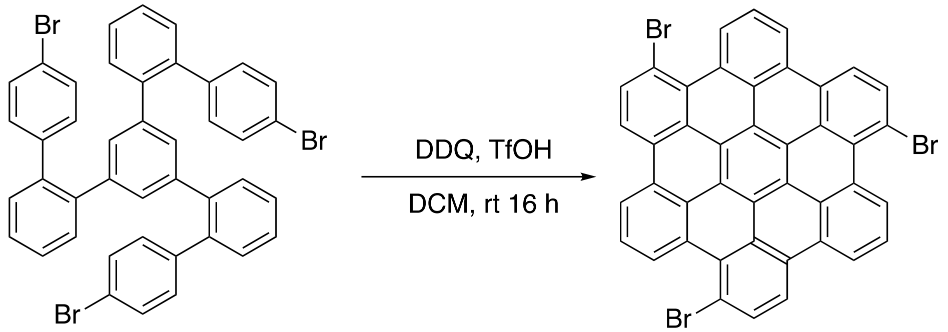

13 **17**

1,7,13-tribromohexabenzo[*bc*,*ef*,*hi*,*kl*,*no*,*qr*]coronene (**17**)

To a resealable pressure tube (c.a. 13×1000) was added **13** (77.1 mg, 0.10 mmol, 100 mol%) and DDQ (136.2 mg, 0.60 mmol, 600 mol%). Dichloromethane (7.7 mL) was added followed by TfOH (53 μL, 0.60 mmol, 600 mol%). The tube was stirred at ambient temperature. After 16 h, MeOH (7.7 mL) was added followed by water (7.7 mL). The yellow precipitation was separated by filtration and washed by DCM, THF, water, and MeOH. The residue was dried under reduced pressure to provide the title compound **17** (56.1 mg, 0.074 mmol) as a dark yellow solid in 74% yield.

MP: 354 °C (sublimed)

MS: (MALDI-TOF) Calculated for $C_{42}H_{16}Br_3$ [M+H$^+$] = 756.9, Found 756.4.

FTIR: (neat) 1737, 1626, 1370, 831 cm^{-1}

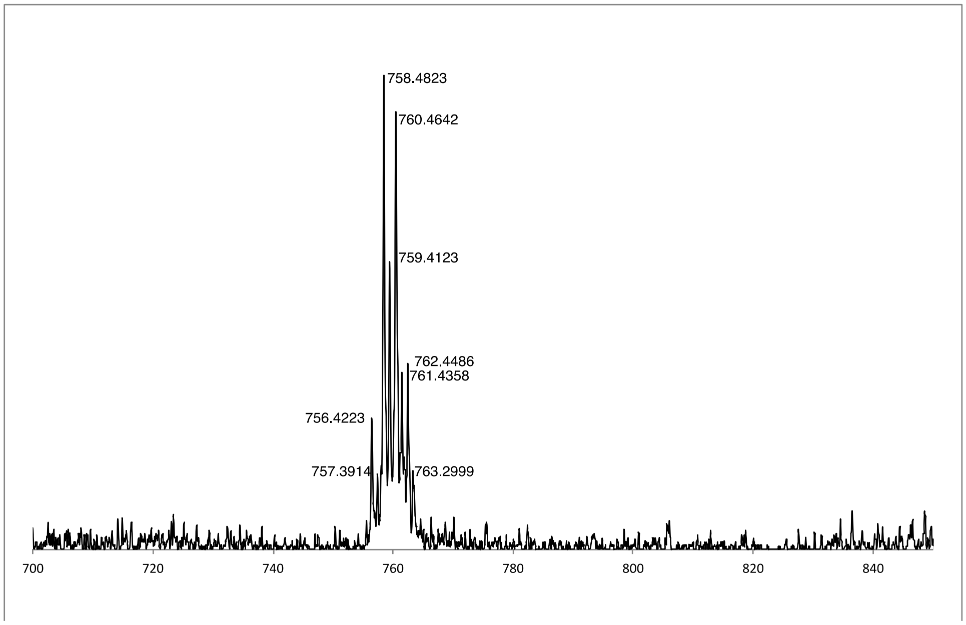

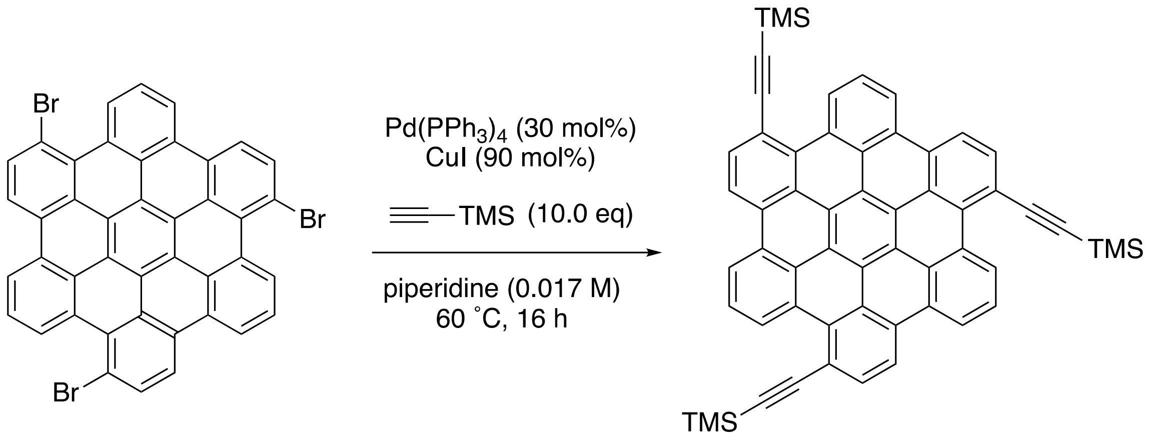

17 **18**

1,7,13-tris((trimethylsilyl)ethynyl)hexabenzo[*bc*,*ef*,*hi*,*kl*,*no*,*qr*]coronene (**18**)

To a resealable pressure tube (c.a. 13×1000) was added Pd(PPh$_3$)$_4$ (34.7 mg, 0.03 mmol, 30 mol%), CuI (17.1 mg, 0.09 mmol, 90 mol%), and **17** (75.9 mg, 0.10 mmol, 100 mol. Freshly distilled piperidine (5.9 mL) was added to the tube followed by trimethylsilylacetylene (0.14 mL, 1.0 mmol, 10 eq). The tube was placed in 60 °C oil bath. After 16 h, MeOH (20 mL) was added followed by water (10 mL). The yellow precipitation was separated by filtration and washed by MeOH and water repeatedly. The residue was subjected to flash column chromatography (SiO$_2$; hexanes:DCM = 5:95 to 20:80) to furnish the title compound **18** (38.9 mg, 0.048 mmol) in 48% yield as a bright yellow solid.

TLC (SiO$_2$): R_f = 0.25 (hexanes : DCM = 80:20).

^{1}H NMR: (400 MHz, CDCl$_3$/CS$_2$ = 1:1): δ = 8.83 (d, *J* = 6.8 Hz, 3H), 7.34–7.30 (m, 6H), 6.99 (d, *J* = 7.1 Hz, 3H), 6.60 (t, *J* = 6.8 Hz, 3H), 0.33 (s, 27H) ppm.

^{13}C NMR: (100 MHz, CDCl$_3$/CS$_2$ = 1:1): δ = 133.0, 128.6, 128.0, 126.7, 126.0, 124.6, 123.4, 122.3, 121.2, 119.0, 117.5, 117.1, 117.0, 114.8, 109.8, 98.6, 0.17 ppm.

HRMS: (APCI) Calculated for $C_{57}H_{42}Si_3$ [M+H$^+$] =811.2667, Found 811.2667.

FTIR: (neat): 2362, 1738, 1365, 1216, 840 cm^{-1}.

MP: >250 °C.

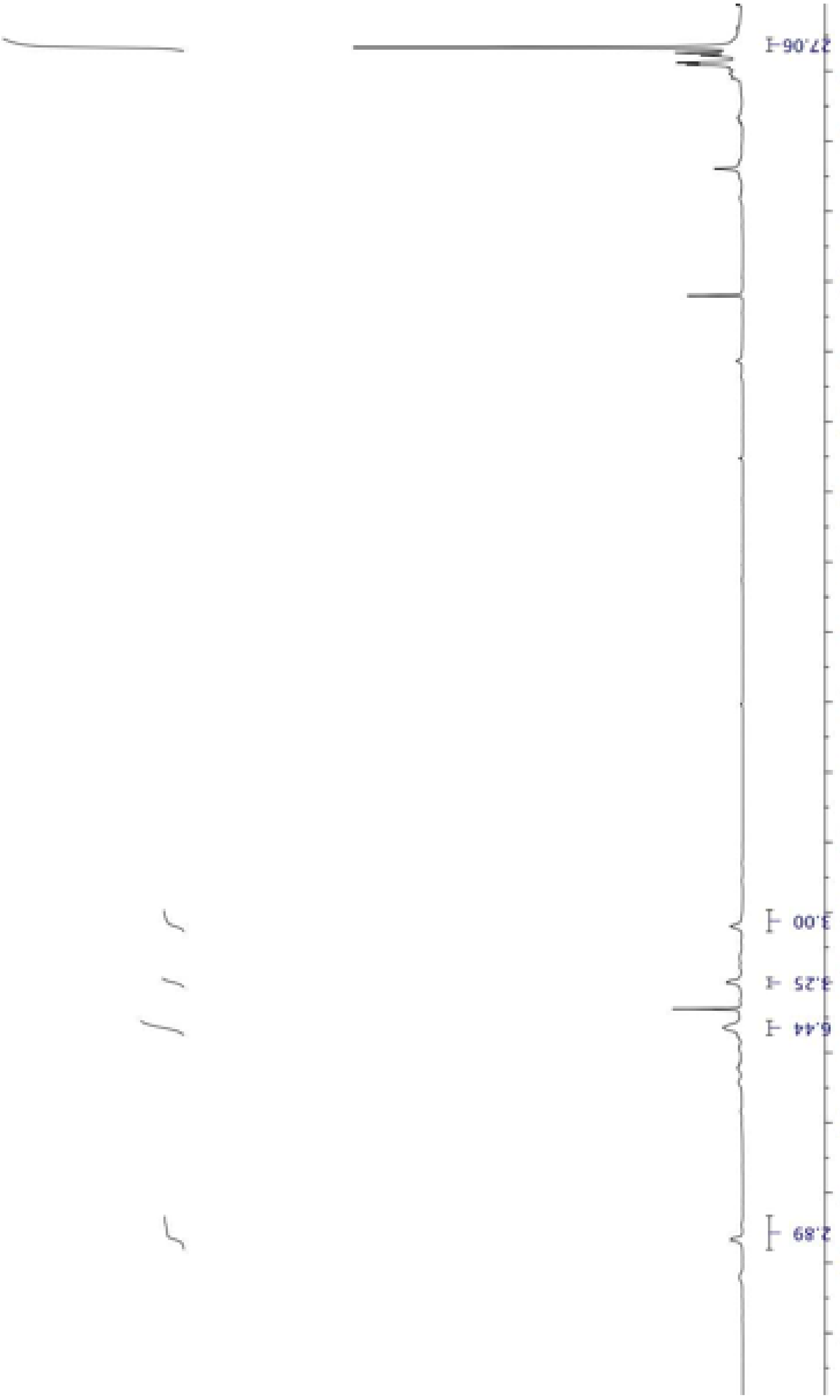

27.06
3.00
3.25
6.44
2.89
0.5
1.0
1.5
2.0
2.5
3.0
3.5
4.0
4.5
5.0
5.5
6.0
6.5
7.0
7.5
8.0
8.5
9.0
9.5

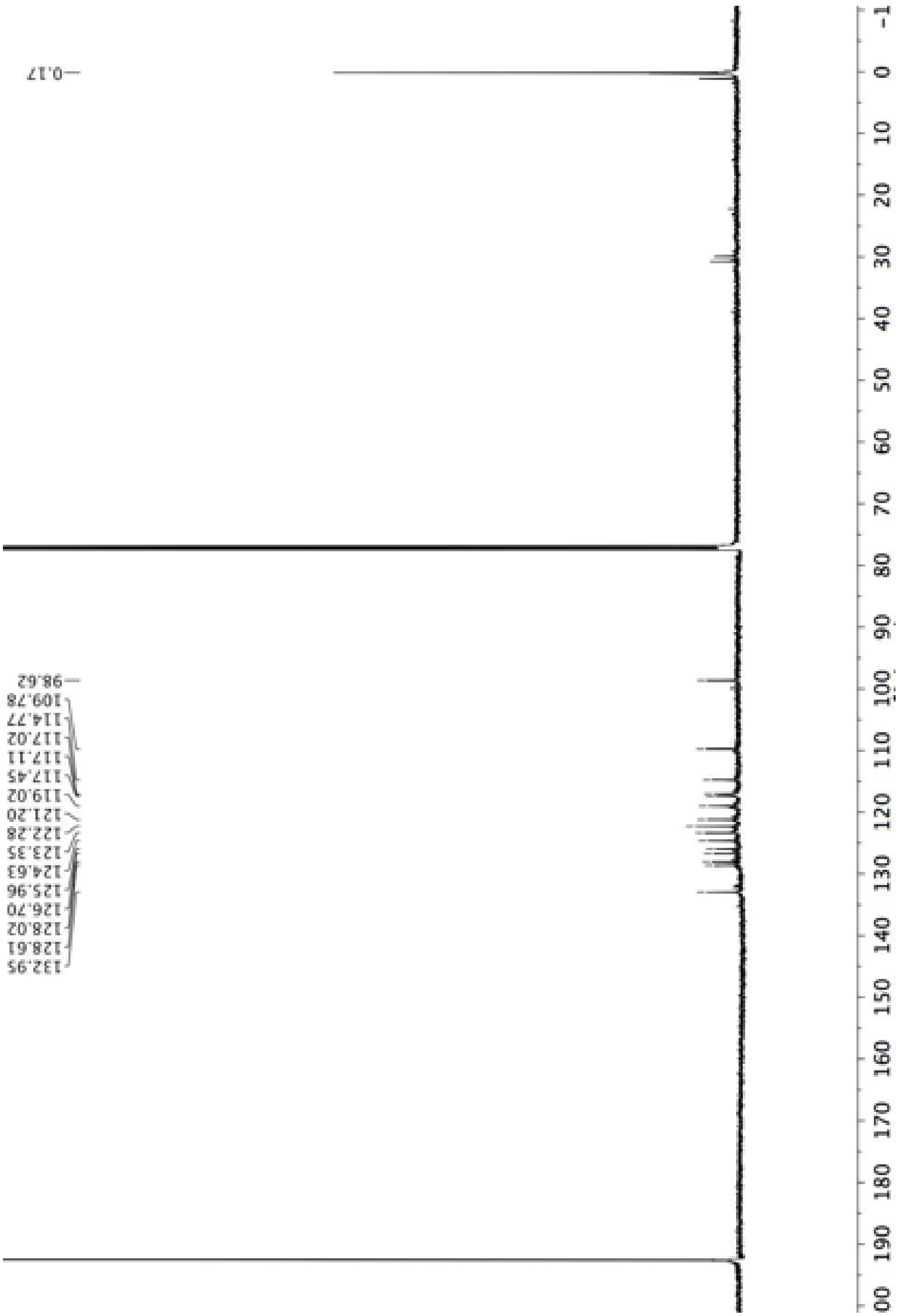

0.17
98.62
109.78
114.77
117.02
117.11
117.45
119.02
121.20
122.28
123.35
124.63
125.96
126.70
128.02
128.61
132.95
-1
0
10
20
30
40
50
60
70
80
90
100
110
120
130
140
150
160
170
180
190

Synthesis and Characterization of Biphenyl Boronic Acids and Boronates

S12 was synthesized according to literature protocol. All spectral data was identical.[vii]
Intervening bromide precursor to **S14** was synthesized according to literature procedure. All spectral data was identical.[viii]
S10 was synthesized according to literature protocol. All spectral data was identical.[ix]
S23 was synthesized according to literature protocol. All spectral data was identical.[x]

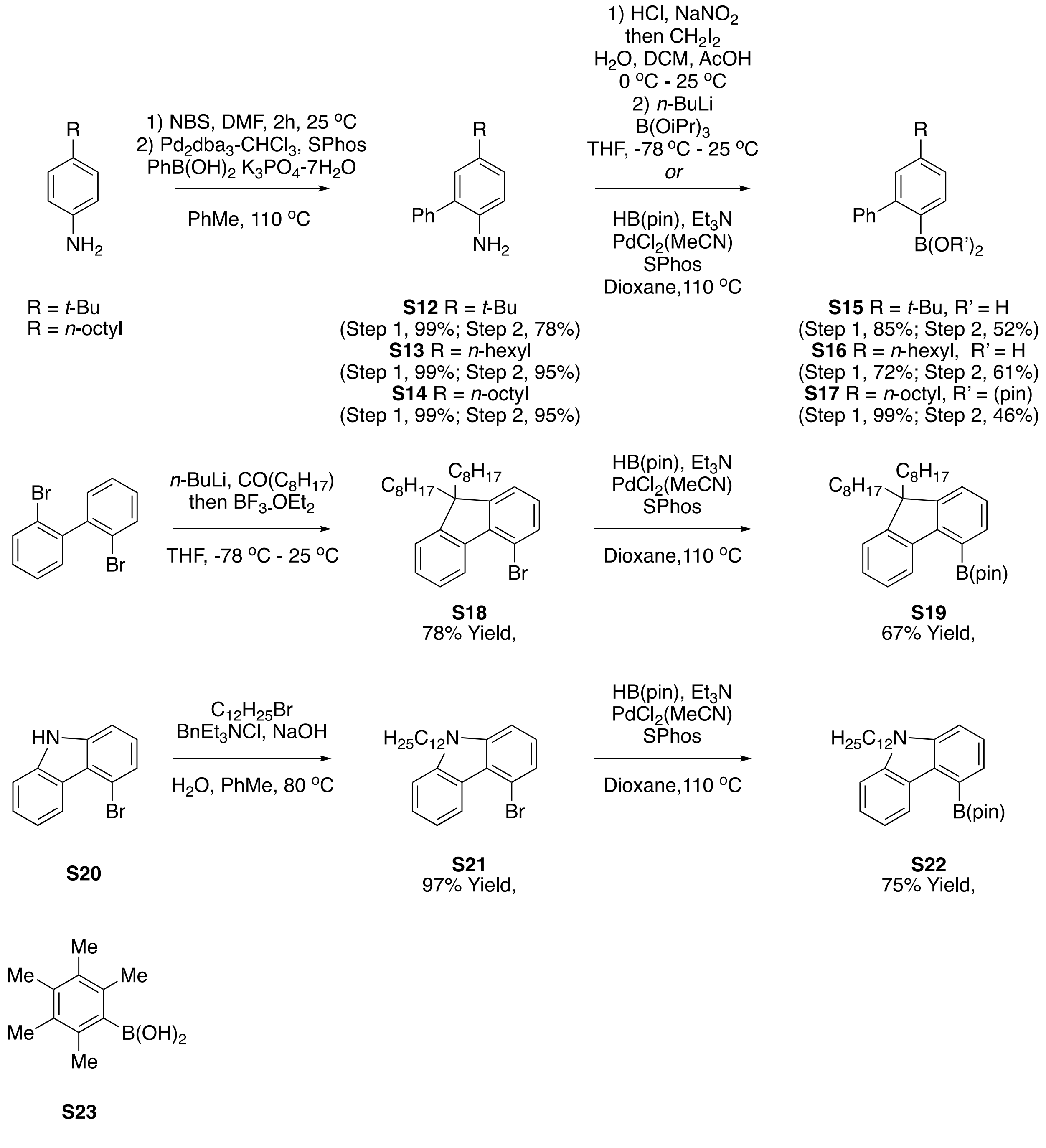

General procedures for boronic acid or boronate synthesis:

To a sealed tube equipped with stir bar was added 2-bromo-4-octylaniline (2.80g, 9.80 mmol, 100 mol%), phenylboronic acid (1.68g, 13.8 mmol, 140 mol%), Pd_2dba_3 (102.4 mg, 0.098 mmol, 1 mol%), SPhos (161 mg, 0.392 mmol, 4 mol%), and K_3PO_4-$7H_2O$ (6.77g, 29.4 mmol, 300 mol%). The seal tube was capped with a septum and then purged with argon for 10 min, after which PhMe (35ml, 0.3M) was added. The reaction mixture was sparged for additional 5 min with argon. The septum was replaced with a screw cap and the reaction mixture was heated to 110 ^oC for 18 hr. The reaction mixture was then cooled to room temperature and concentrated onto SiO_2, which was then subjected to silica gel chromotography to yield **S13** (2.61g, 9.31 mmol, 95% yield) as a viscous, colorless oil. Ca. 10% impurities could not be removed by chromatography.

Aniline **S14** (563 mg, 2.0 mmol, 100 mol%) was suspended in a mixture H_2O:DCM (1:1, 12 ml, 0.17 M) and AcOH (2.3 mL, glacial, 40 mmol, 2000 mol%). The reaction mixture was cooled to 0 ^oC and was allowed to stir for 15 minutes. At 0 ^oC $NaNO_2$ (0.690 g, 10 mmol, 1000 mol%) was then added and the mixture was stirred for a further 45 minutes, keeping the temperature at 0 ^oC. CH_2I_2 (1.071 g, 4.0 mmol, 200 mol%) was then added and the reaction was allowed to warm to room temperature over 18hrs. The mixture was then extracted with DCM (10 mL × 3), dried over Na_2SO_4, filtered, and the volatiles were removed by rotary evaporation. The residue was the purified by silica gel column chromatography (hexane: ether, 95:5) to give 2-iodo-5-octyl-1,1'-biphenyl (0.780 g, 2.0 mmol, 99% yield) as a colorless oil.

To a sealed tube equipped with stir bar was added 2-iodo-5-octyl-1,1'-biphenyl (0.78 g, 2.0 mmol, 100 mol%), $PdCl_2(MeCN)_2$ (10.4 mg, 0.04 mmol, 2 mol%), and SPhos (65.7 mg, 0.16 mmol, 8 mol%). The reaction vessel was purged with argon. Dioxane (3.5 mL, 0.6 M) and Et_3N (0.83 mL, 6.0 mmol, 300 mol%) were added, followed by carful addition of HB(pin) (0.44 mL, 3.0 mmol, 150 mol%). The reaction tube was then sealed, heated to 110 ^oC, and allowed to stir for 18 hr. The reaction mixture was then cooled, and ~5 drops of MeOH was added carefully (CAUTION: rapid H_2 gas evolution). The mixture was then concentrated onto SiO_2, which was then subjected to silica gel chromatography to yield **S17** (0.358 g, 0.91 mmol, 46% yield) as a colorless oil.

Alternatively, to a flask equipped with stir bar was added 2-iodo-5-*tert*-butyl-1,1'-biphenyl (1.43 g, 4.25 mmol, 100 mol%) and THF (30 mL, 0.15 M). The reaction mixture was cooled to -78 ^oC, and *n*-BuLi (1.95 mL, 2.5 M in hexane, 4.67 mmol, 110 mol%) was added dropwise. The reaction was stirred at -78 ^oC for 30 min before $B(OiPr)_3$ (2.92 mL, 12.75 mmol, 300 mol%) was added dropwise. The reaction was allowed to warm to room temperature over 18 hrs. HCl (25 ml, 2M, aq.) was then added and the mixture was extracted with DCM (25 ml × 3). The combined extracts were dried over Na_2SO_4, filtered, and the volatiles were removed by rotary evaporation. The residue was passed through a plug of SiO_2 with DCM and then concentrated. The resulting solid was recrystallized from 1:1 hexane: MeOH to afford **S15** (560 mg, 2.20 mmol, 52 % yield) as a colorless solid.

(5-(*tert*-butyl)-[1,1'-biphenyl]-2-yl)boronic acid (**S15**)

TLC (SiO_2): R_f = 0.65 (hexanes : Et_2O = 95:5).

^{1}H NMR: (500 MHz, $CDCl_3$): δ 7.47-7.43 (m, 3H), 7.39-7.36 (m, 2H), 7.28 (s, 1H), 7.13 (dd, *J* = 7.9, 1.7 Hz, 1H), 6.67 (d, *J* = 7.9 Hz, 1H), 1.34 (s, 9H) ppm.

^{13}C NMR: (125 MHz, $CDCl_3$): δ = 154.6, 149.8, 144.8, 137.5, 129.2, 127.8, 127.1, 126.5, 122.9, 34.9, 31.2 ppm.

HRMS: (ESI+) Calculated for $C_{18}H_{23}BO_2$ [$M+C_2H_5^+$] =282.1900, Found 282.1907.

FTIR: (neat): 3283, 2957, 1600, 1339, 1131, 829, 753, 699 cm^{-1}.

MP: 90-93 ^oC

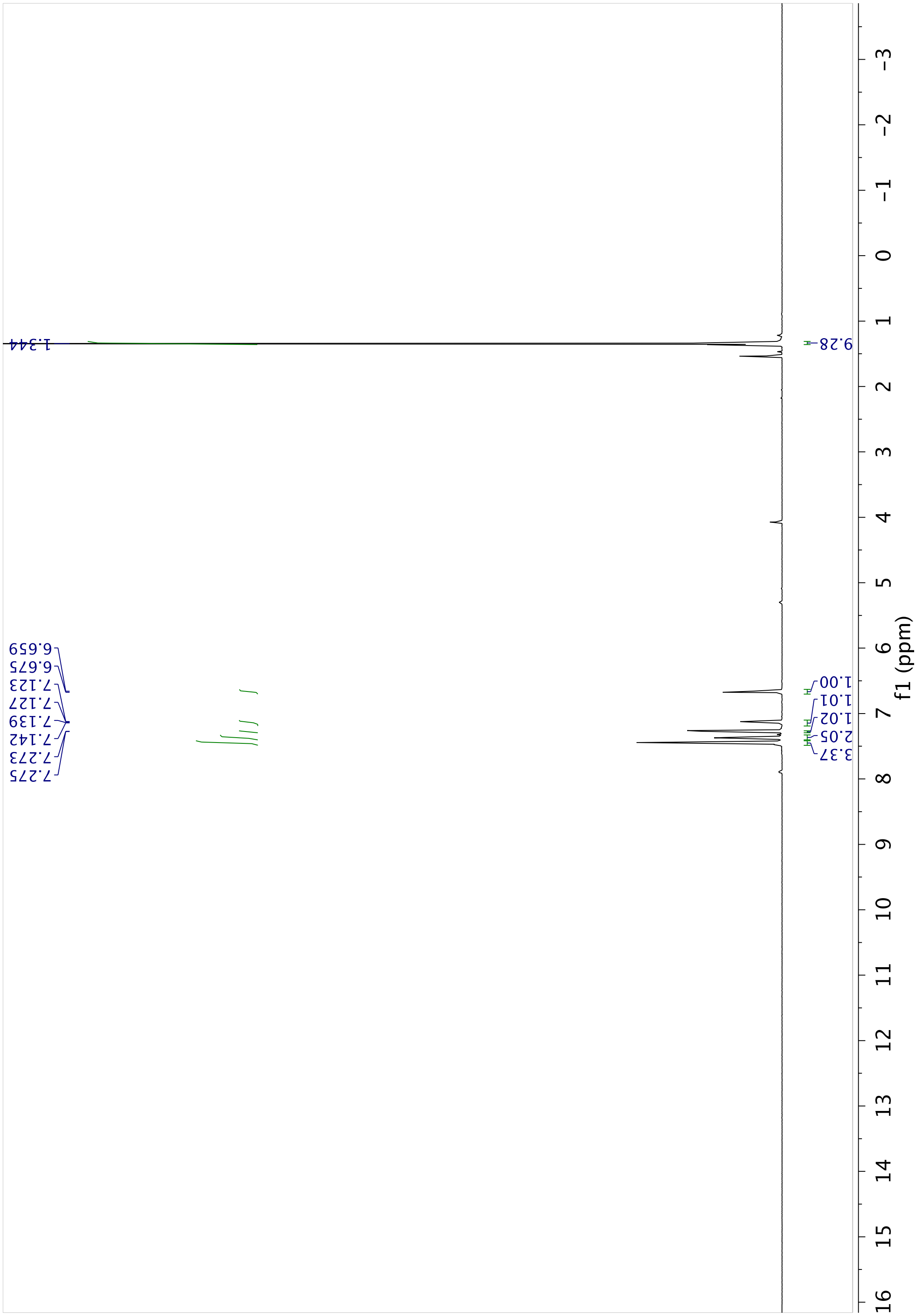

1.344
6.659
6.675
7.123
7.127
7.139
7.142
7.273
7.275
9.28
1.00
1.01
1.02
2.05
3.37
f1 (ppm)

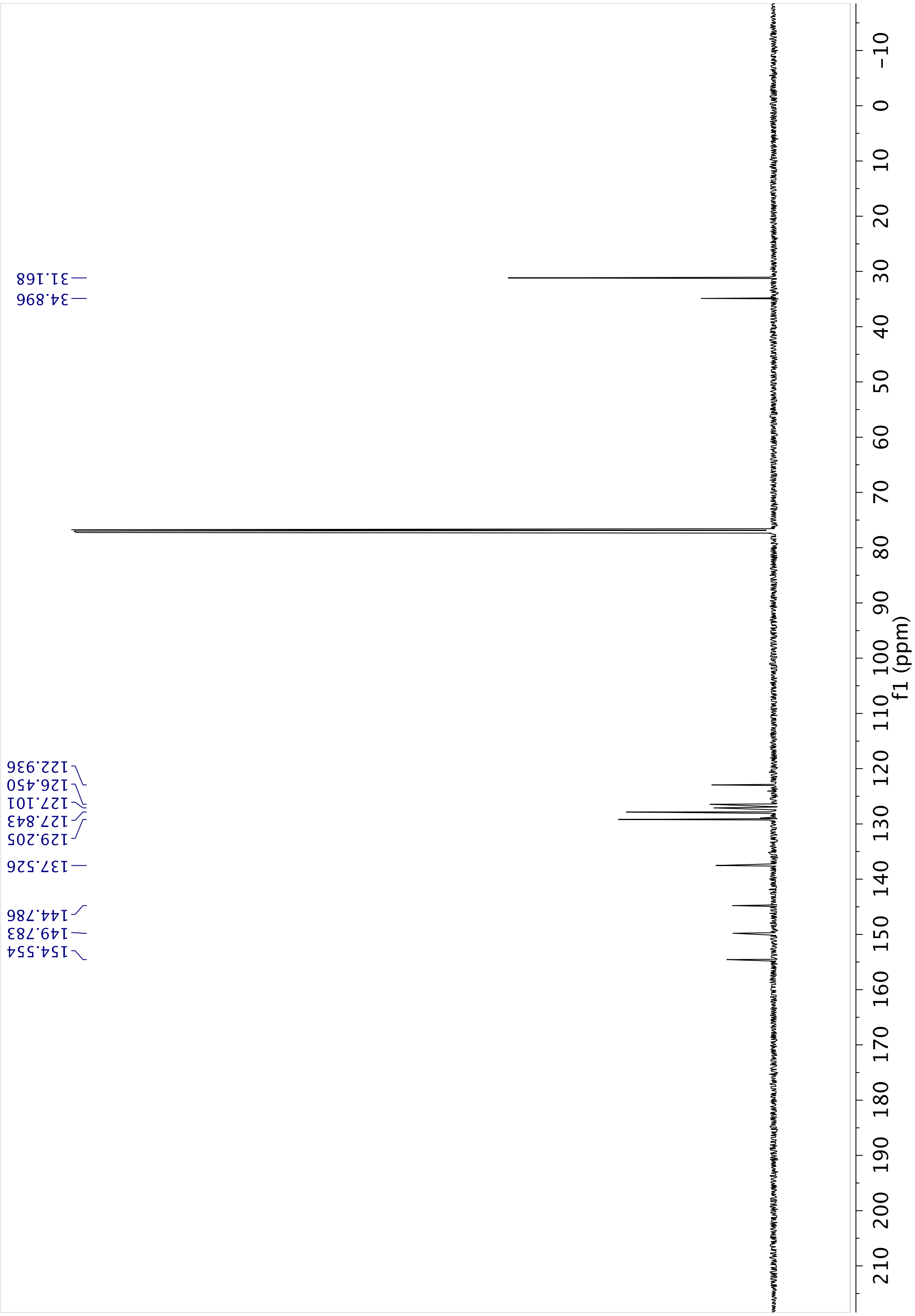
31.168
34.896
122.936
126.450
127.101
127.843
129.205
137.526
144.786
149.783
154.554
f1 (ppm)

(5-hexyl-[1,1'-biphenyl]-2-yl)boronic acid (**S16**)

TLC (SiO_2): R_f = 0.80 (hexanes : Et_2O = 95:5).

^{1}H NMR: (400 MHz, $CDCl_3$): δ = 7.80 (d, *J* = 7.2 Hz, 1H), 7.67–7.57 (m, 5H), 7.53 (t, *J* = 7.8 Hz, 1H), 7.35 (d, *J* = 18.0 Hz, 1H), 2.88 (t, *J* = 8 Hz, 2H), 1.87 (quin., *J* = 7.6 Hz, 2H) 1.64-1.59 (m, 6H), 1.16-1.10 (m, 3H) ppm.

^{13}C NMR: (100 MHz, $CDCl_3$) δ 150.0, 146.6, 144.5, 137.8, 130.2, 129.2, 127.8, 126.5, 126.2, 36.1, 31.7, 31.2, 29.1, 22.6, 14.1 ppm.

HRMS: (ESI+) Calculated for $C_{18}H_{24}BO_2$ [M+H$^+$] =282.1900, Found 282.1898.

FTIR: (neat): 3290, 2899, 1591, 1345, 900, 810, 740, 680 cm^{-1}.

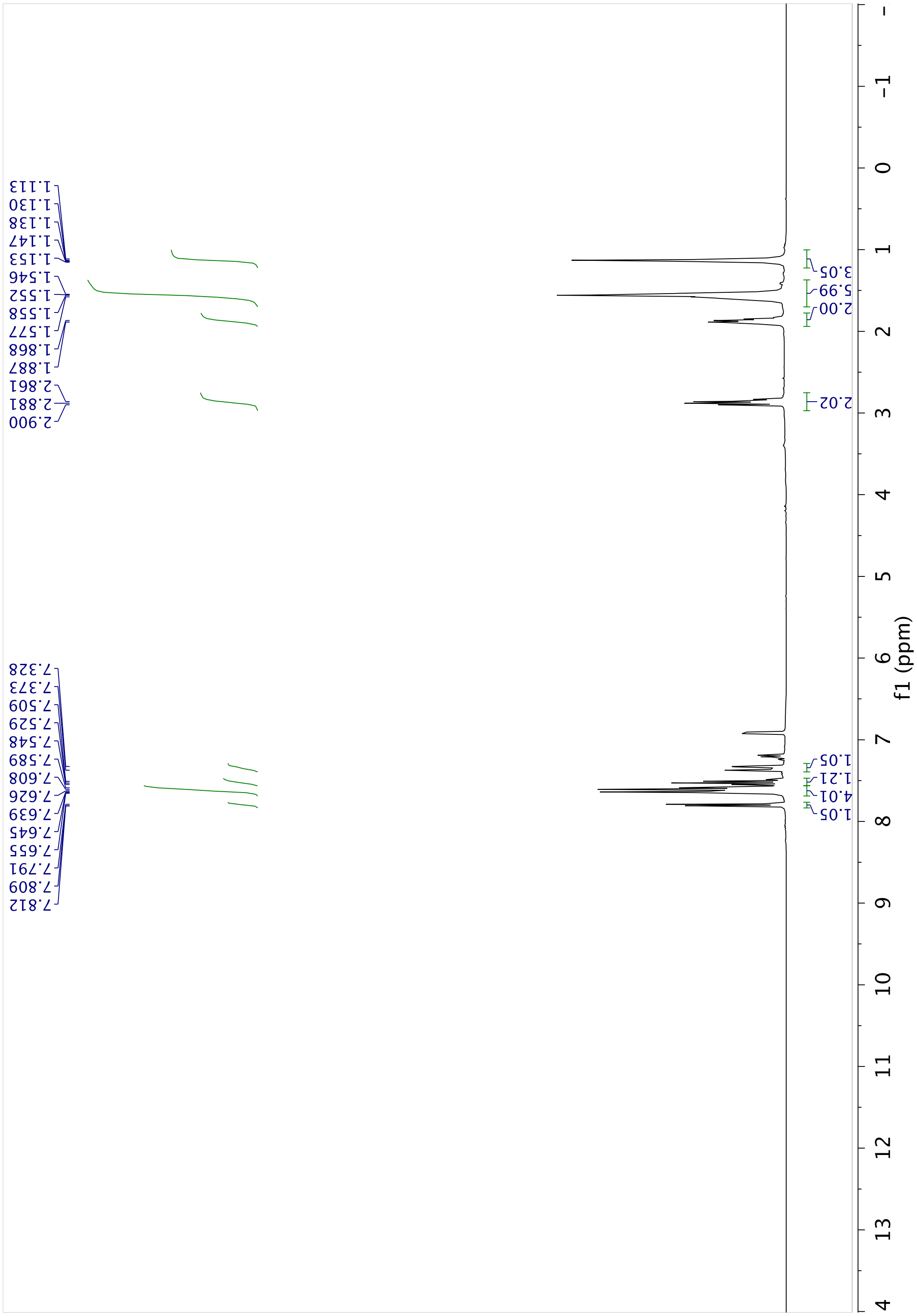

7.812
7.809
7.791
7.655
7.645
7.639
7.626
7.608
7.589
7.548
7.529
7.509
7.373
7.328
2.900
2.881
2.861
1.887
1.868
1.577
1.558
1.552
1.546
1.153
1.147
1.138
1.130
1.113
1.05
4.01
1.21
1.05
2.02
2.00
5.99
3.05
14
13
12
11
10
9
8
7
6
5
4
3
2
1
0
-1
f1 (ppm)

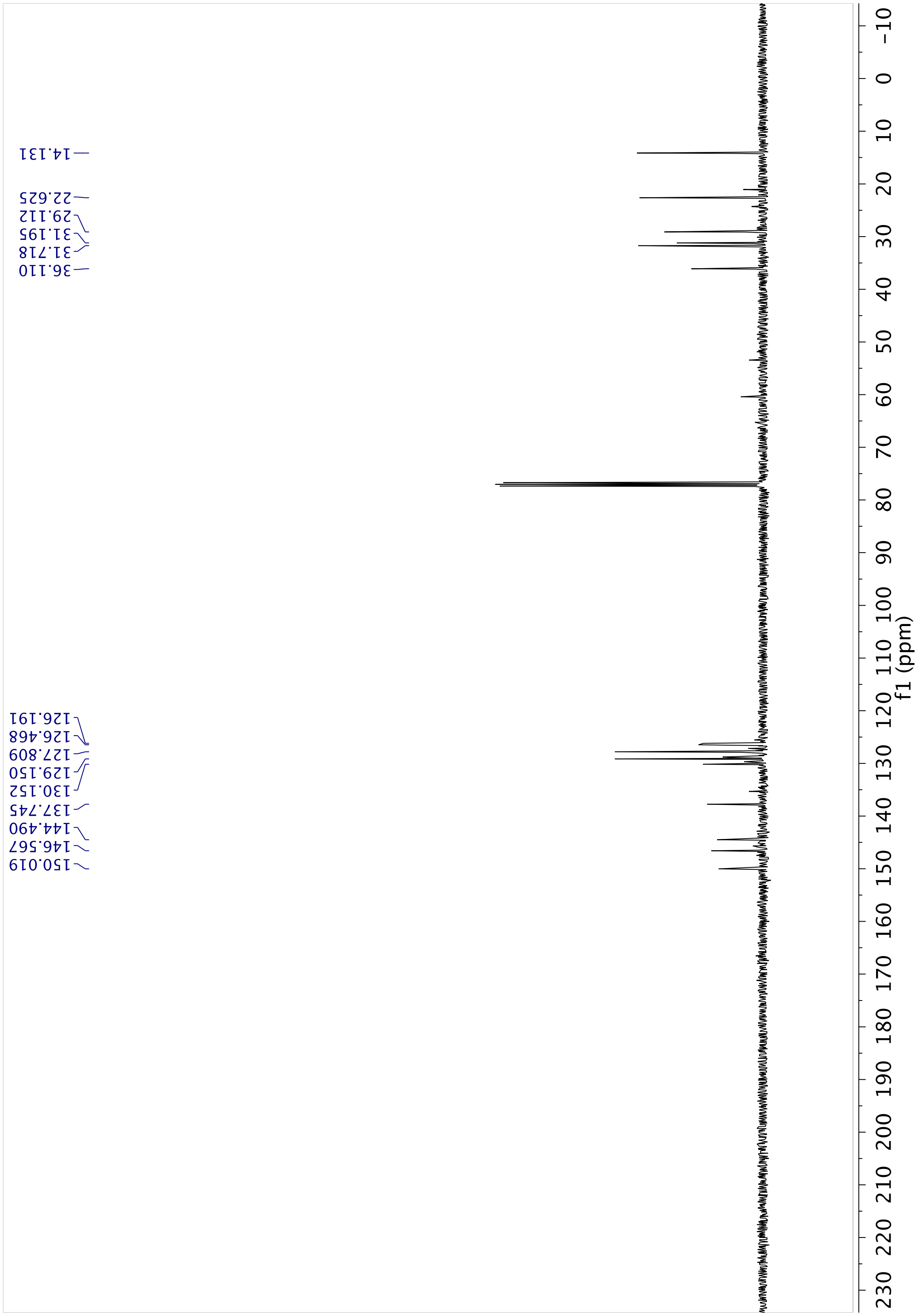
14.131
22.625
29.112
31.195
31.718
36.110
126.191
126.468
127.809
129.150
130.152
137.745
144.490
146.567
150.019
f1 (ppm)

4,4,5,5-tetramethyl-2-(5-octyl-[1,1'-biphenyl]-2-yl)-1,3,2-dioxaborolane (**S17**)

TLC (SiO_2): R_f = 0.90 (hexanes : Et_2O = 95:5).

^{1}H NMR: (400 MHz, $CDCl_3$): δ = 7.64 (d, *J* = 7.5 Hz, 1H), 7.42 – 7.28 (m, 5H), 7.18 (s, 1H), 7.15 (d, *J* = 7.6 Hz, 1H), 2.62 (t, *J* = 7.7 Hz, 2H), 1.61 (quin, *J* = 6.8 Hz, 2H), 1.37-1.21 (m, 10H), 1.89 (s, 12H), 0.87 (t, *J* = 6.0 Hz, 3H).

^{13}C NMR: (100 MHz, $CDCl_3$): δ = 146.3, 144.3, 143.2, 139.2, 130.4, 129.5, 129.3, 129.2, 128.7, 128.0, 127.9, 127.6, 127.5, 94.7, 35.4, 31.9, 31.3, 29.5, 29.3, 29.3, 22.7, 14.2 ppm.

HRMS: (ESI+) Calculated for $C_{26}H_{37}BO_2$ [M+H$^+$] =392.2996, Found 392.3003.

FTIR: (neat): 3330, 2915, 1581, 1300, 80, 854, 800, 710 cm^{-1}

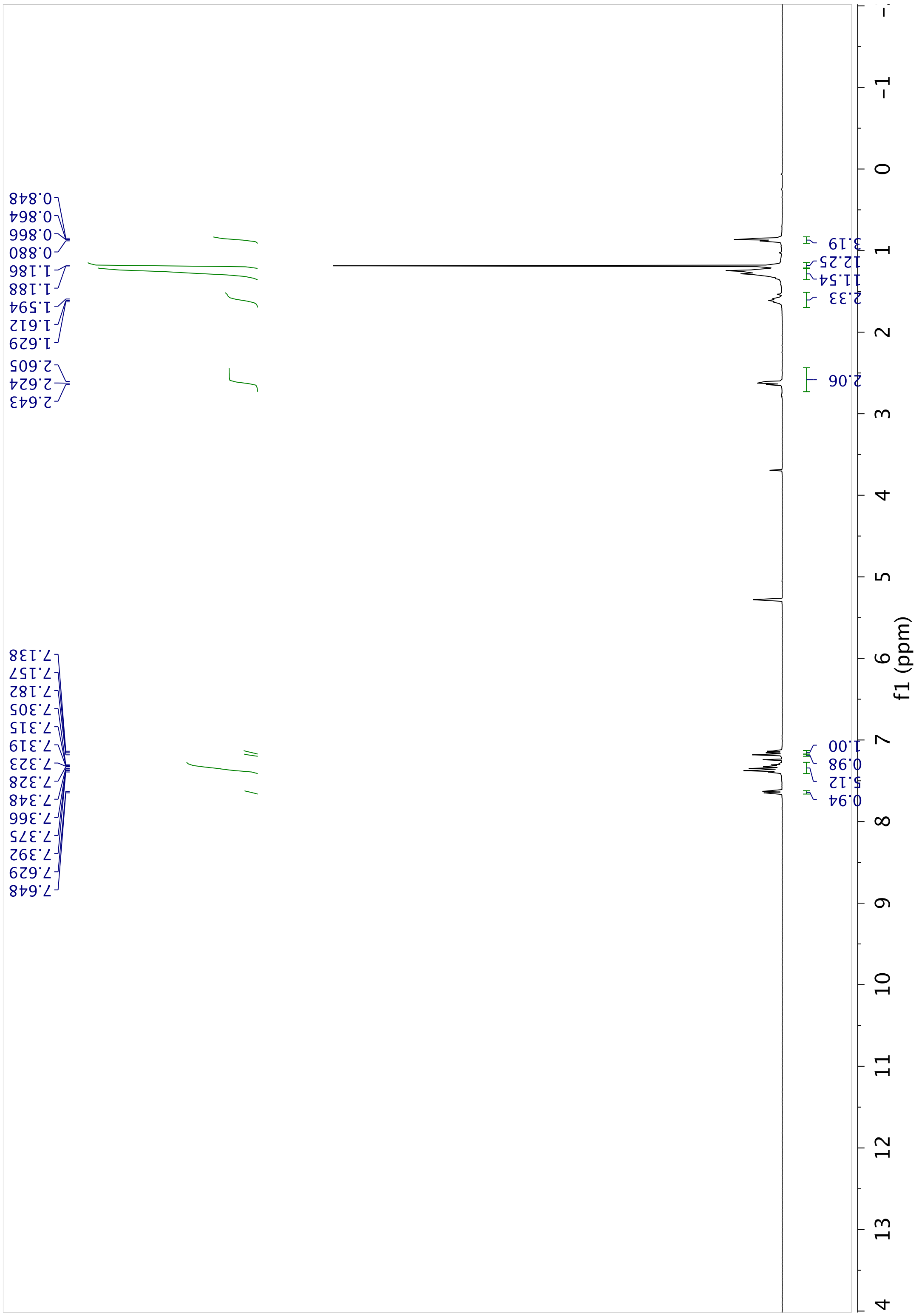
0.848
0.864
0.866
0.880
1.186
1.188
1.594
1.612
1.629
2.605
2.624
2.643
7.138
7.157
7.182
7.305
7.315
7.319
7.323
7.328
7.348
7.366
7.375
7.392
7.629
7.648
3.19
12.25
11.54
2.33
2.06
1.00
0.98
5.12
0.94
f1 (ppm)

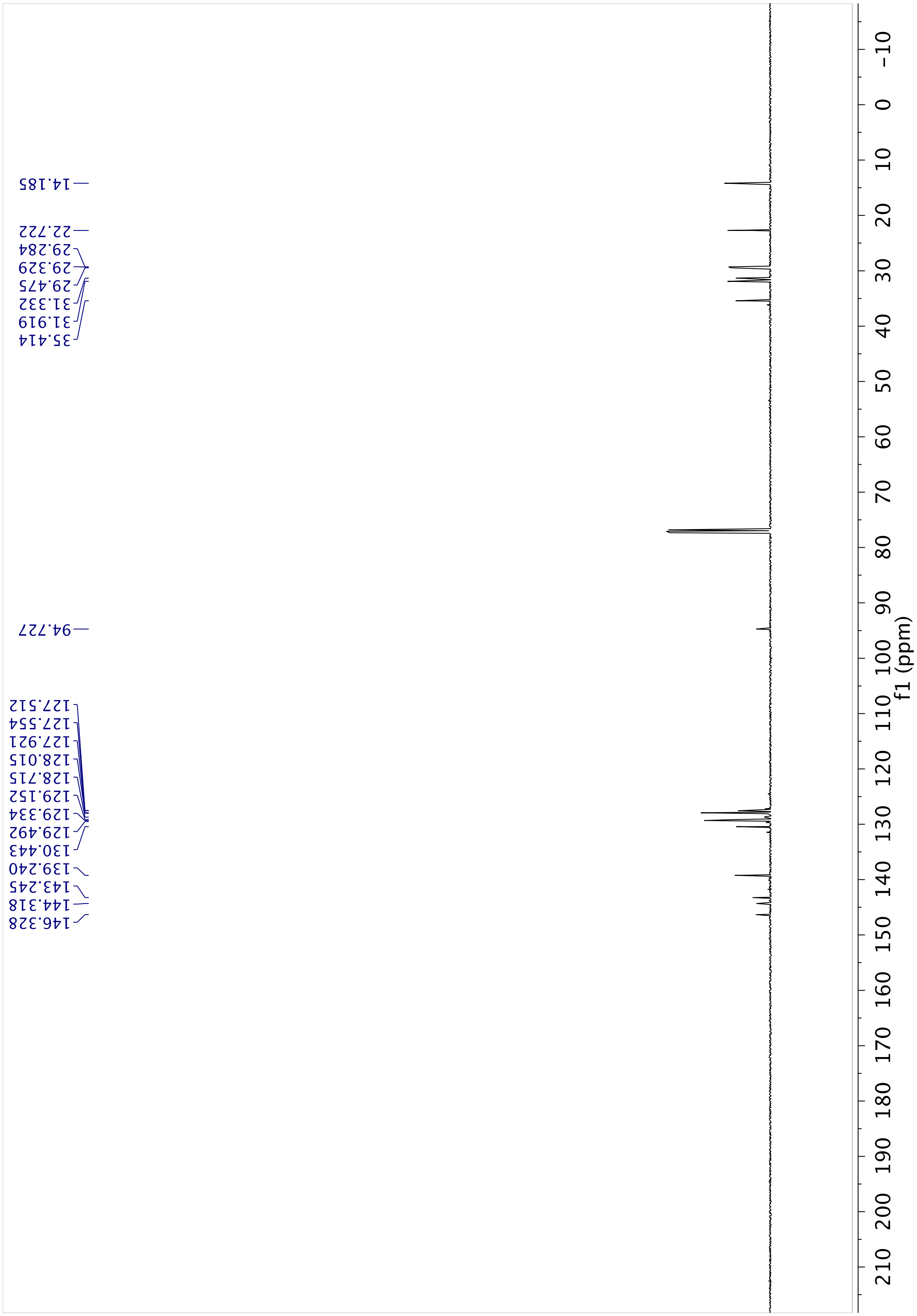

14.185
22.722
29.284
29.329
29.475
31.332
31.919
35.414
94.727
127.512
127.554
127.921
128.015
128.715
129.152
129.334
129.492
130.443
139.240
143.245
144.318
146.328
f1 (ppm)

2-(9,9-dioctyl-9*H*-fluoren-4-yl)-4,4,5,5-tetramethyl-1,3,2-dioxaborolane (**S19**)

To a flask equipped with stir bar was charged 2,2'-dibromobiphenyl (0.936 g, 3.0 mmol, 100 mol%) and THF (6 mL, 0.5 M). The reaction mixture was cooled to -78 ºC, and *n*-BuLi (1.25 mL, 2.5 M in hexane, 3.15 mmol, 105 mol%) was added dropwise. The reaction was stirred at -78 ºC for 30 min before 9-heptadecanone (839 mg, 3.3 mmol, 110 mol%) in 6 mL THF was added dropwise. The mixture was allowed to warm to room temperature over 18 hr. H_2O was then added, and the mixture was extracted with DCM (15 mL × 3). The combined extracts were dried over Na_2SO_4, filtered, and the volatiles were removed by rotary evaporation. The residue was passed through a plug of SiO_2 with DCM and then concentrated. The resulting crude solid was then dissolved in DCM (5 mL, 0.6 M), cooled to 0 ºC, and BF_3-OEt_2 (0.75, 6.0 mmol, 200 mol%) was added. The mixture was allowed to warm to room temperature over 5 hrs. H_2O was then added, and the mixture was extracted with DCM (15 mL × 3). The combined extracts were dried over Na_2SO_4, filtered, and the volatiles were removed by rotary evaporation. The residue was the purified by silica gel column chromatography (hexane: ether, 95:5) to give **S18** (1.104 g, 2.35 mmol, 78 % yield) as a colorless oil.

S18 was then subject to the general borylation procedure to give **S19**.

TLC (SiO_2): R_f = 0.86 (hexanes : Et_2O = 98:2).

^{1}H NMR: (400 MHz, $CDCl_3$): δ = 8.68-8.65 (m, 1H), 7.79 (dd, *J* = 7.4, 1.2 Hz, 1H), 7.44 (dd, *J* = 7.5, 1.2 Hz, 1H), 7.37 – 7.29 (m, 4H), 1.99 – 1.92 (m, 4H), 1.49 (s, 12H), 1.30 – 0.98 (m, 20H), 0.85 (t, *J* = 7.1 Hz, 6H), 0.71 – 0.44 (m, 4H) ppm.

^{13}C NMR: (100 MHz, $CDCl_3$): δ = 151.0, 150.7, 145.8, 142.4, 134.7, 126.8, 126.4, 125.8, 125.1, 123.4, 122.3, 84.0, 54.0, 40.7, 31.8, 30.1, 29.2, 29.2, 25.0, 23.6, 22.6 14.1ppm.

HRMS: (ESI+) Calculated for $C_{35}H_{53}BO_2$ [M+H$^+$] = 516.4248, Found 516.4257.

FTIR: (neat): 3350, 2905, 1622, 1310, 80, 874, 831, 740 cm^{-1}.

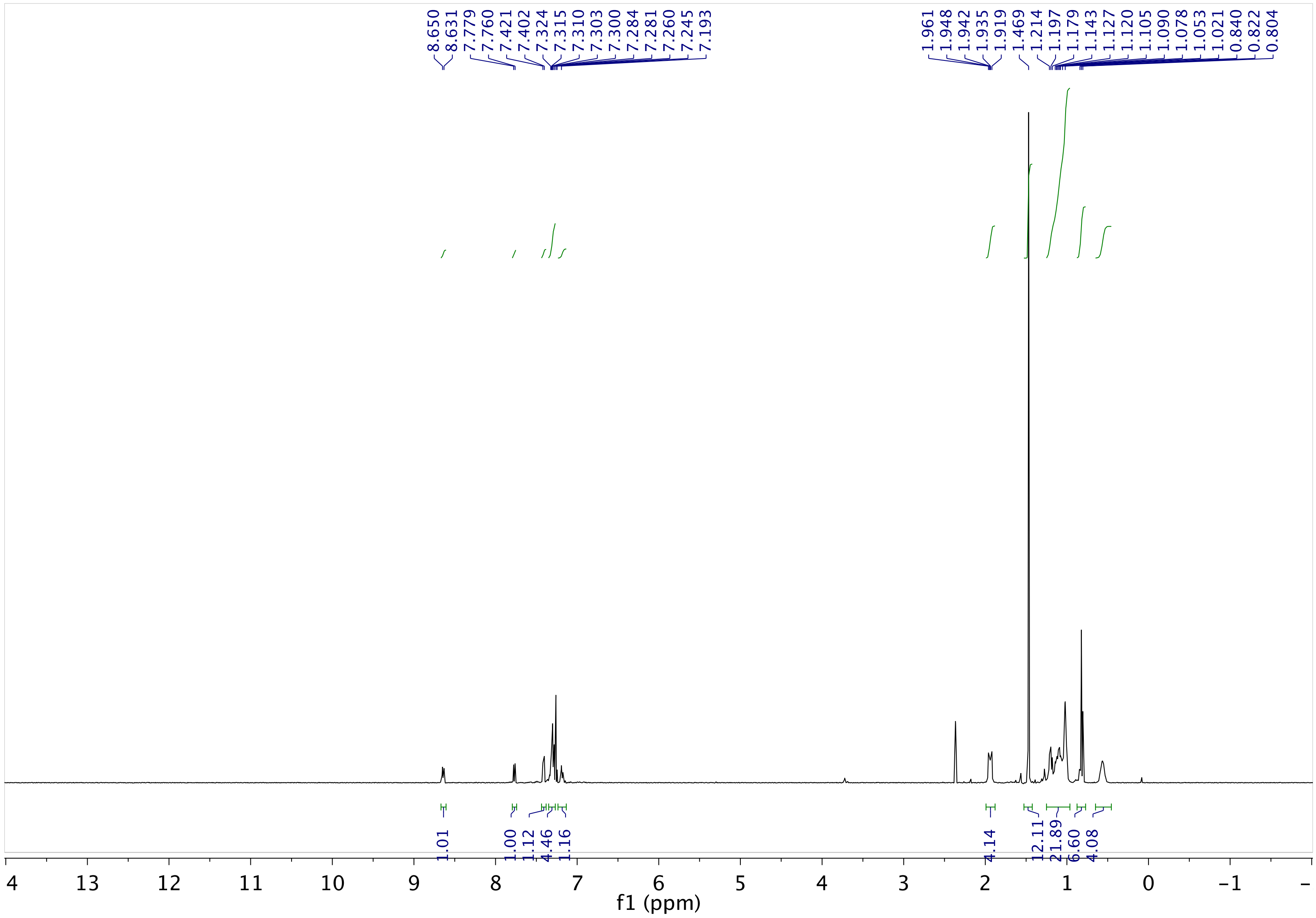
8.650
8.631
7.779
7.760
7.421
7.402
7.324
7.315
7.310
7.303
7.300
7.284
7.281
7.260
7.245
7.193
1.961
1.948
1.942
1.935
1.919
1.469
1.214
1.197
1.179
1.143
1.127
1.120
1.105
1.090
1.078
1.053
1.021
0.840
0.822
0.804
1.01
1.00
1.12
4.46
1.16
4.14
12.11
21.89
6.60
4.08
f1 (ppm)

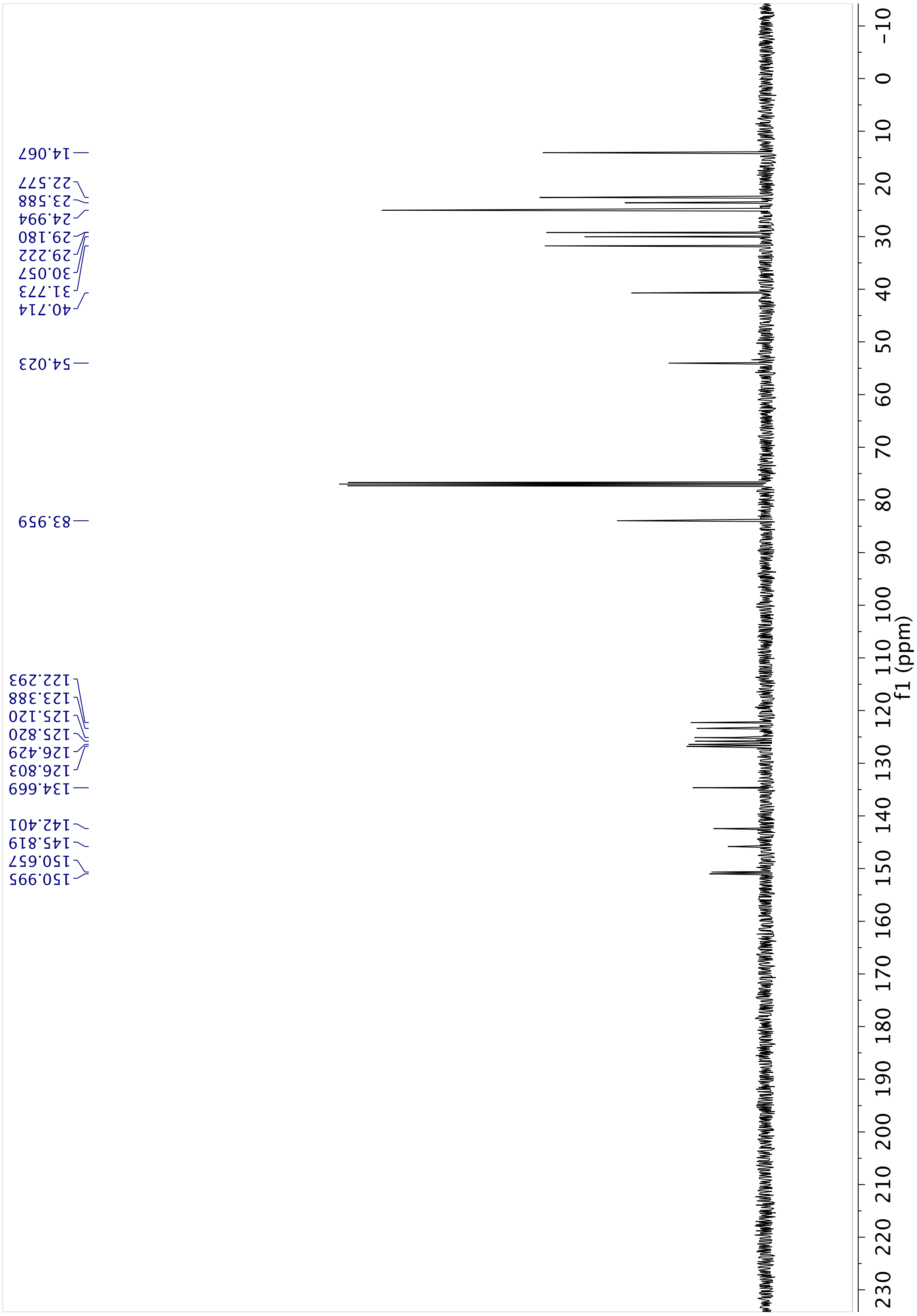
14.067
22.577
23.588
24.994
29.180
29.222
30.057
31.773
40.714
54.023
83.959
122.293
123.388
125.120
125.820
126.429
126.803
134.669
142.401
145.819
150.657
150.995
f1 (ppm)

9-dodecyl-4-(4,4,5,5-tetramethyl-1,3,2-dioxaborolan-2-yl)-9*H*-carbazole (**S22**)

To a flask equipped with stir bar was added **S20** (123 mg, 0.5 mmol, 100 mol%), $BnEt_3NCl$ (11.4 mg, 0.05 mmol, 10 mol%), NaOH (50% aq, 0.05 mL, 0.525 mmol, 105 mol%), and PhMe (1 mL, 0.5 M), followed by 1-bromododecane (131 mg, 0.525 mmol, 105 mol%). The reaction mixture was heated to 60°C for 18hrs. The reaction mixture was cooled and H_2O was added. The mixture was extracted with DCM (15 mL × 3). The combined extracts were dried over Na_2SO_4, filtered, and the volatiles were removed by rotary evaporation. The residue was the purified by silica gel column chromatography (hexane: ether, 95:5) to give **S21** (200 mg, 0.483 mmol, 97% yield) as a colorless oil.

S21 was then subject to the general borylation procedure to give **S22**.

TLC (SiO_2): R_f = 0.85 (hexanes : Et_2O = 95:5).

^{1}H NMR: (400 MHz, $CDCl_3$): δ = 9.06 (d, *J* = 8.0 Hz, 1H), 7.77 (d, *J* = 6.8 Hz, 1H), 7.54 (dd, *J* = 8.1, 1.0 Hz, 1H), 7.50 – 7.44 (m, 2H), 7.41 (t, *J* = 6.6 Hz, 1H), 7.24 (t, *J* = 7.5 Hz, 1H), 4.32 (t, *J* = 7.3 Hz, 2H), 1.85 (quin, *J* = 7.6 Hz, 2H), 1.49 (s, 12H), 1.31-1.21 (m, 18H), 0.89 (t, *J* = 6.8Hz, 3H)ppm.

^{13}C NMR: (100 MHz, $CDCl_3$): δ = δ 140.7, 140.0, 127.5, 126.5, 125.5, 124.4, 124.3, 123.6, 118.5, 111.5, 108.2, 83.9, 42.8, 31.9, 29.6, 29.6, 29.5, 29.4, 29.3, 28.9, 27.3, 25.0, 22.7, 22.7, 14.1 ppm.

HRMS: (ESI+) Calculated for $C_{30}H_{45}BNO_2$ [M+H$^+$] =462.3538, Found 462.3540.

FTIR: (neat): 3390, 1601, 1450, 1322, 753, 715 cm^{-1}.

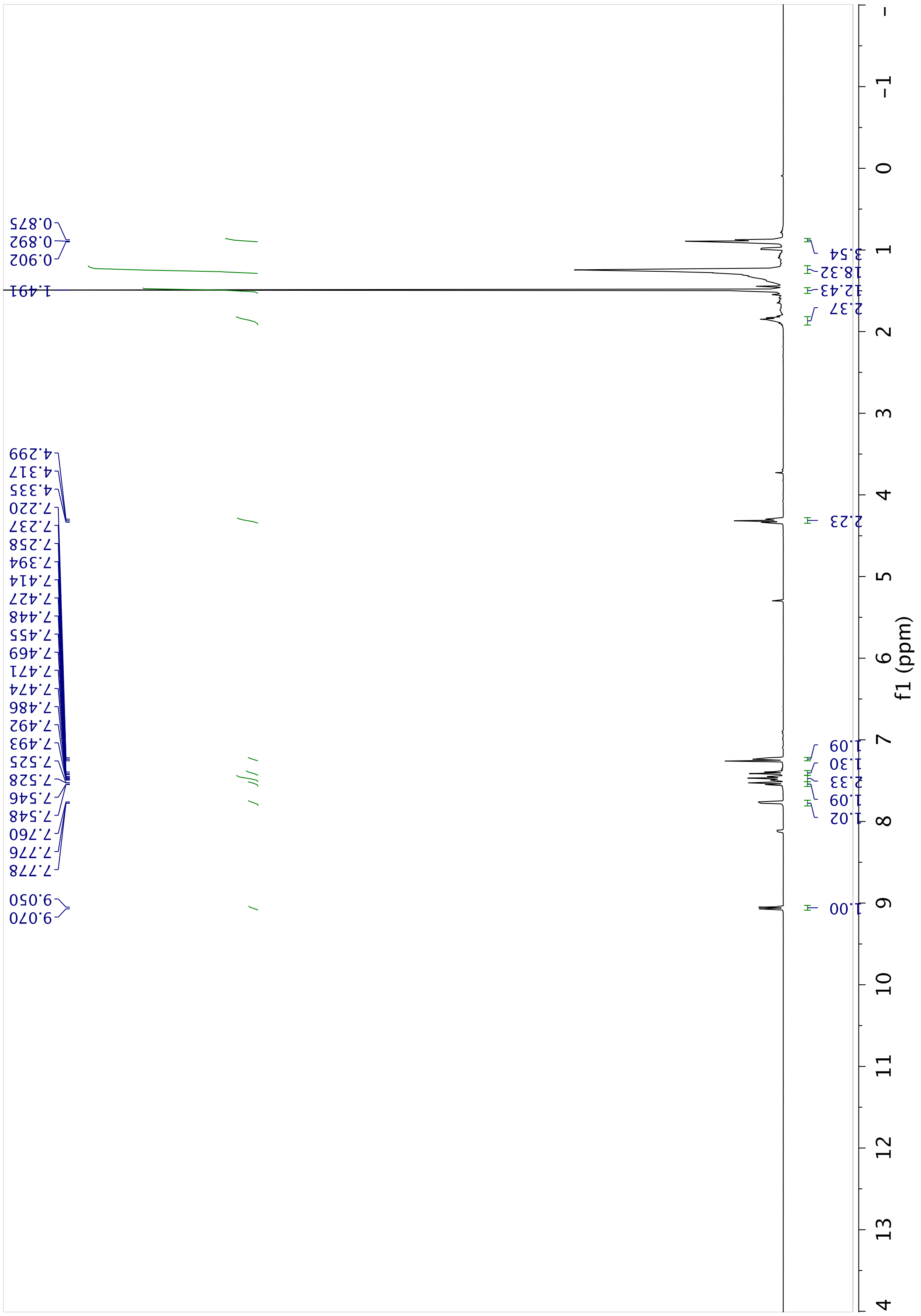
0.875
0.892
0.902
1.491
4.299
4.317
4.335
7.220
7.237
7.258
7.394
7.414
7.427
7.448
7.455
7.469
7.471
7.474
7.486
7.492
7.493
7.525
7.528
7.546
7.548
7.760
7.776
7.778
9.050
9.070
3.54
18.32
12.43
2.37
2.23
1.09
1.30
2.33
1.09
1.02
1.00
f1 (ppm)

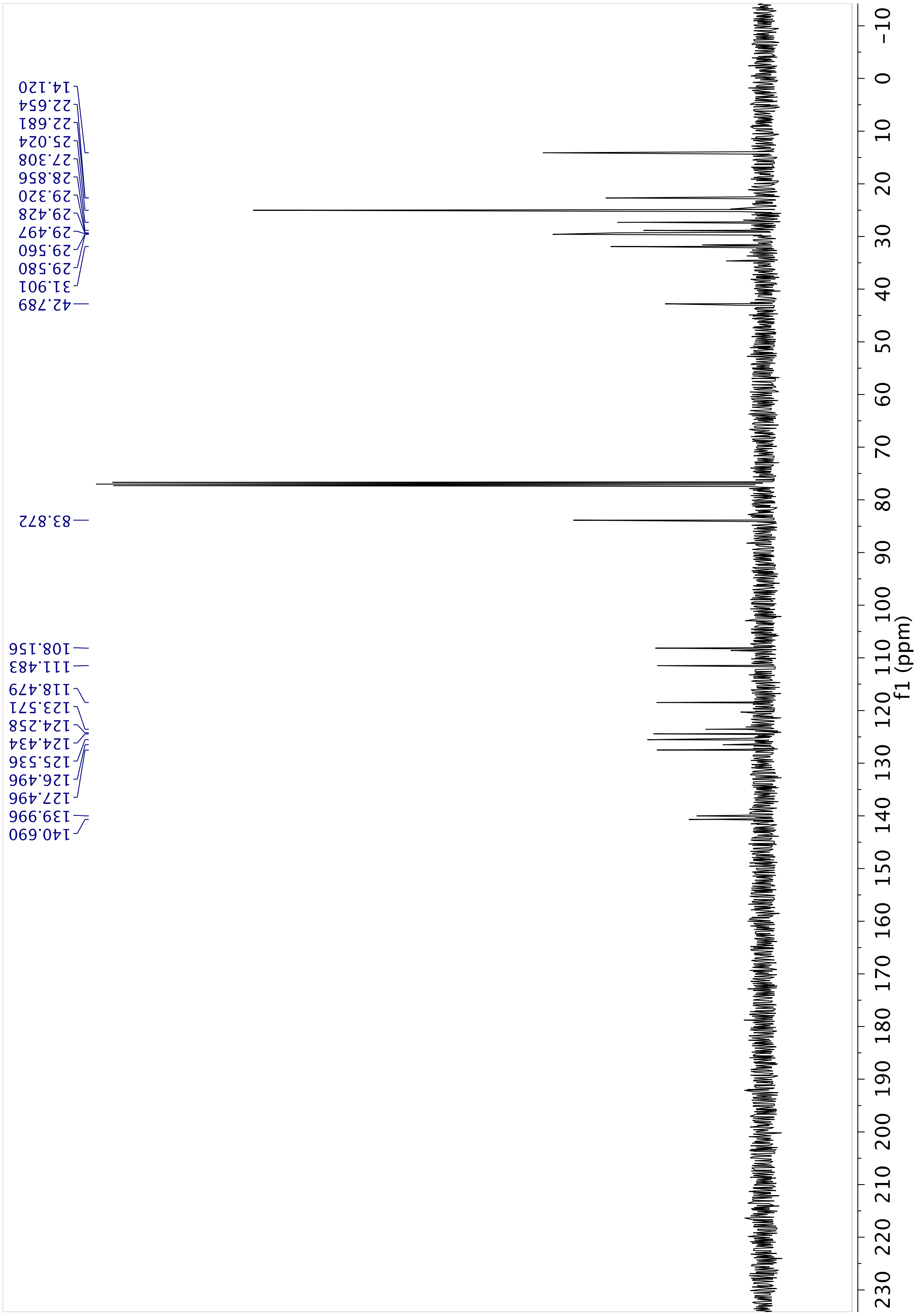
14.120
22.654
22.681
25.024
27.308
28.856
29.320
29.428
29.497
29.560
29.580
31.901
42.789
83.872
108.156
111.483
118.479
123.571
124.258
124.434
125.536
126.496
127.496
139.996
140.690
f1 (ppm)

Synthesis and Characterization of Oligophenylenes 15a-h via Cross Coupling

General procedure for cross coupling of **11** and pinacol boronates or boronic acids:

To a sealed tube equipped with stir bar was added **11** (34.6 mg, 0.05 mmol, 100 mol%), boronic acid or pinacol ester (0.15 mmol, 300 mol%), $Pd(OAc)_2$ (0.2 mg, 0.001 mmol, 2 mol%), SPhos (0.8 mg, 0.002 mmol, 4 mol%), and K_3PO_4-$7H_2O$ (46 mg, 0.2 mmol, 400 mol%). The sealed tube was capped with a septum and purged with argon for 5 min. Then, PhMe (0.1 mL, 0.5 M) was added and the septum was quickly replaced with a screw cap. The reaction mixture was then heated to 110 °C for 48 hrs. The reaction mixture was then allowed to cool to room temperature, was adsorbed onto silica under reduced pressure, and subjected to flash column chromatography (SiO_2, 90:10 hexane: DCM) to afford **15a-g** in 55-98% yield.

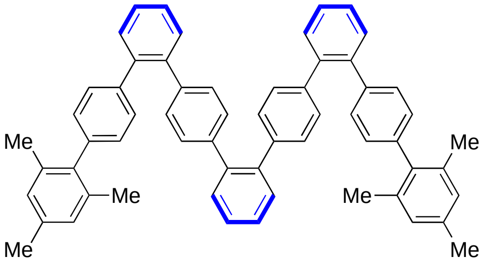

Oligophenylene **15a**

TLC (SiO_2): R_f = 0.46 (hexanes : Et_2O = 95:5).

^{1}H NMR: (500 MHz, $CDCl_3$): δ = 7.56-7.34 (m, 12H), 7.23-7.13 (m, 4H), 7.07-6.94 (m, 16H), 2.36 (s, 6H), 2.01 (s, 12H) ppm.

^{13}C NMR: (125 MHz, $CDCl_3$): δ = 140.7, 140.5, 140.4, 139.7, 139.2, 138.8, 136.5, 136.0, 130.4, 130.4, 130.3, 130.1, 129.6, 129.5, 129.4, 129.4, 128.7, 128.1, 127.9, 127.5, 127.5, 21.1, 20.8.ppm.

MS: (MALDI-TOF) Calculated for $C_{60}H_{50}$ [M^+] = 770.4, Found 770.7.

FTIR: (neat): 3022, 2976, 1471, 1348, 1143, 1004, 827, 756, 736, 698 cm^{-1}.

MP: >250 °C

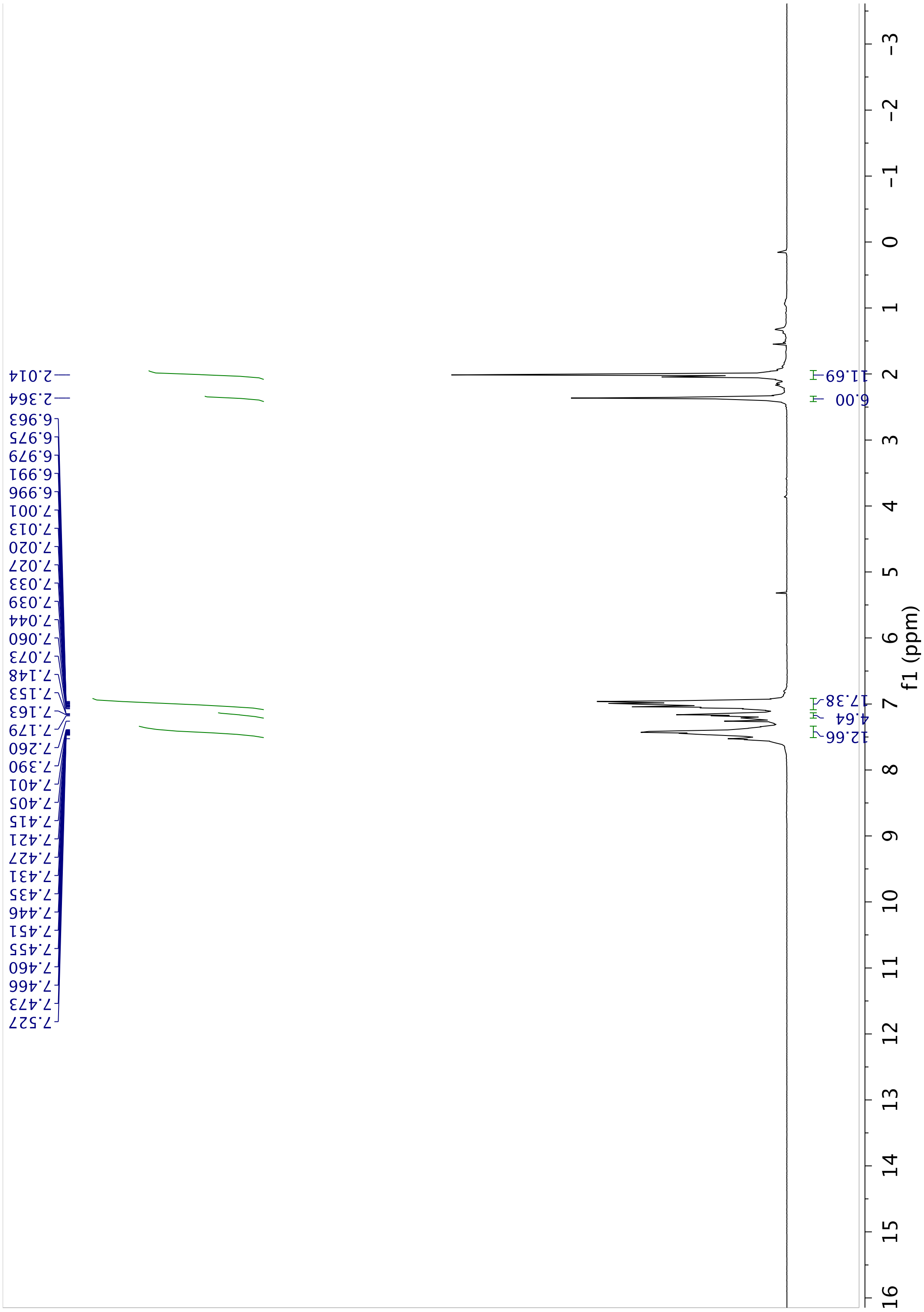
2.014
2.364
6.963
6.975
6.979
6.991
6.996
7.001
7.013
7.020
7.027
7.033
7.039
7.044
7.060
7.073
7.148
7.153
7.163
7.179
7.260
7.390
7.401
7.405
7.415
7.421
7.427
7.431
7.435
7.446
7.451
7.455
7.460
7.466
7.473
7.527
11.69
6.00
17.38
4.64
12.66
f1 (ppm)

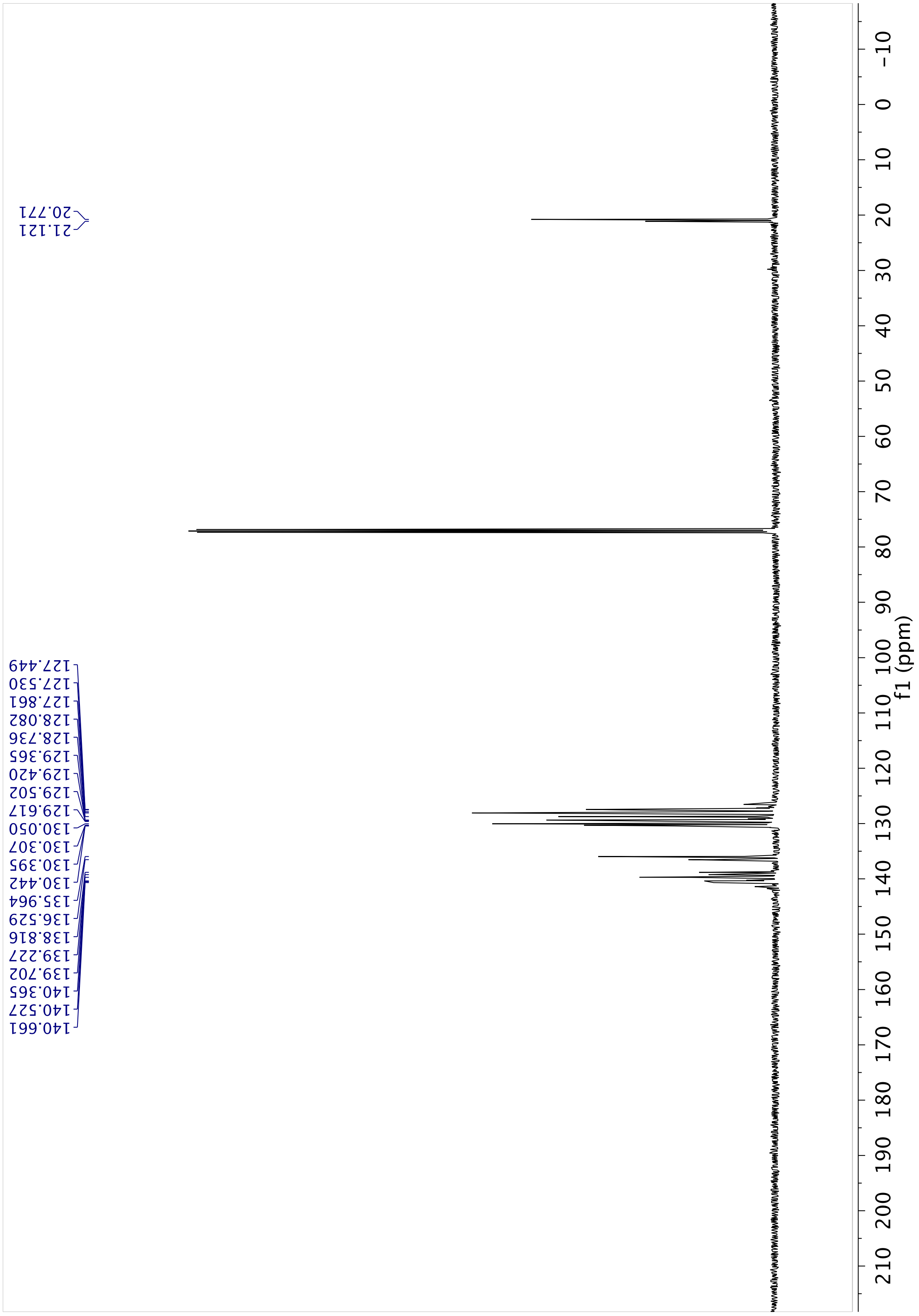

140.661
140.527
140.365
139.702
139.227
138.816
136.529
135.964
130.442
130.395
130.307
130.050
129.617
129.502
129.420
129.365
128.736
128.082
127.861
127.530
127.449
21.121
20.771
f1 (ppm)

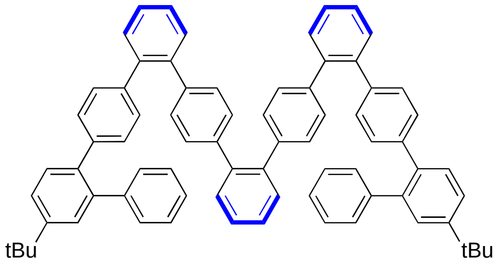

Oligophenylene **15b**

TLC (SiO_2): R_f = 0.48 (hexanes : Et_2O = 95:5).

^{1}H NMR: (500 MHz, $CDCl_3$): δ = 7.54-7.37 (m, 20H) 7.19-7.12 (m, 8H), 7.07-6.87 (m, 16H), 1.43 (s, 18H) ppm.

^{13}C NMR: (125 MHz, $CDCl_3$): δ = 150.3, 141.9, 140.3, 140.2, 140.1, 140.1, 139.6, 139.6, 139.3, 139.2, 137.3, 130.4, 130.2, 130.2, 129.8, 129.6, 129.5, 129.3, 129.2, 127.8, 127.8, 127.5, 127.4, 127.4 127.3, 126.3, 124.5, 34.6, 31.4 ppm.
MS: (MALDI-TOF) Calculated for $C_{74}H_{62}$ [M^+] = 950.5, Found 950.6.

FTIR: (neat): 3022, 2980, 1480, 1298, 1200, 1124, 834, 760, 745, 697 cm^{-1}.

MP: >250 ºC

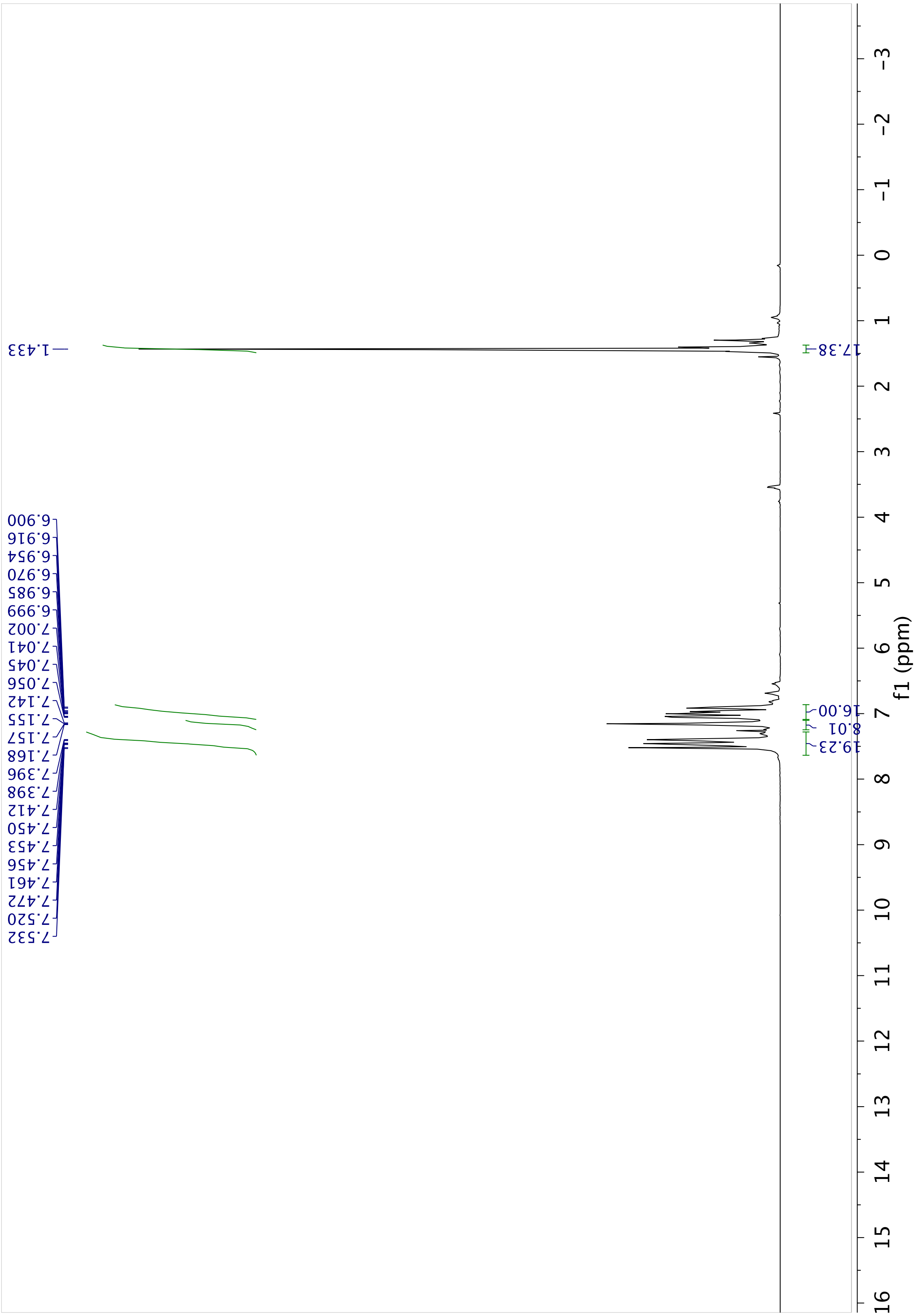
1.433
6.900
6.916
6.954
6.970
6.985
6.999
7.002
7.041
7.045
7.056
7.142
7.155
7.157
7.168
7.396
7.398
7.412
7.450
7.453
7.456
7.461
7.472
7.520
7.532
17.38
16.00
8.01
19.23
f1 (ppm)

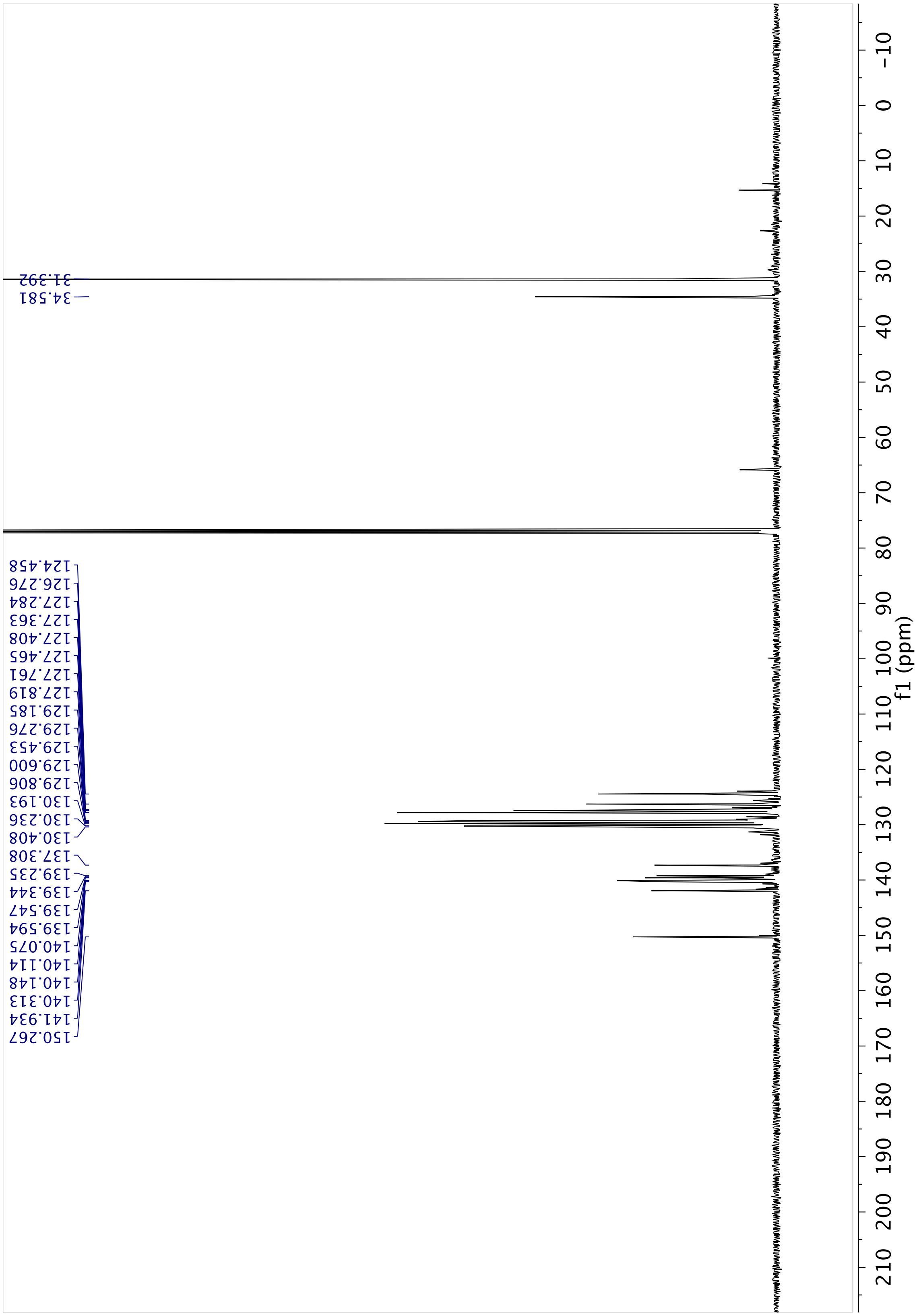
31.392
34.581
124.458
126.276
127.284
127.363
127.408
127.465
127.761
127.819
129.185
129.276
129.453
129.600
129.806
130.193
130.236
130.408
137.308
139.235
139.344
139.547
139.594
140.075
140.114
140.148
140.313
141.934
150.267
f1 (ppm)

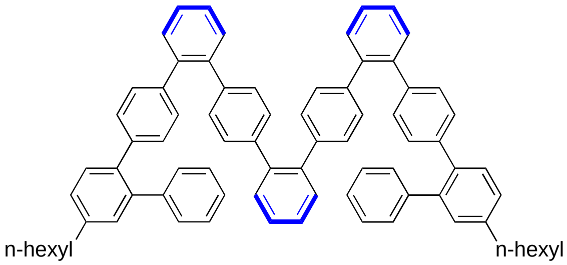

Oligophenylene **15c**

TLC (SiO_2): R_f = 0.66 (hexanes : Et_2O = 95:5).

^{1}H NMR: (400 MHz, $CDCl_3$): δ = 7.57 (s, 4H), 7.51-7.38 (m, 10H), 7.33-7.26 (m, 4H), 7.21-7.25 (m, 8H), 7.12-6.90 (m, 18H), 2.77 (t, *J* = 8.0 Hz, 4H), 1.82-1.73 (m, 4H), 1.55-1.36 (m, 12H), 1.01 (t, *J* = 2.0 Hz, 6H) ppm.

^{13}C NMR: (100 MHz, $CDCl_3$): δ =142.1, 141.6, 140.3, 140.1, 139.7, 139.6, 139.4, 139.2, 137.5, 130.7, 130.4, 130.3, 130.2, 129.7, 129.6, 129.4, 129.3, 129.2, 127.8, 127.5, 127.4, 127.4, 126.3, 35.7, 31.7, 31.5, 29.2, 22.6, 14.1 ppm.

MS: (MALDI-TOF) Calculated for $C_{78}H_{70}$ [M^+] = 1006.5, Found 1006.4.

FTIR: (neat): 3090, 2981, 1434, 1287, 1150, 1100, 850, 770, 750, 700 cm^{-1}.

MP: >250 ^oC

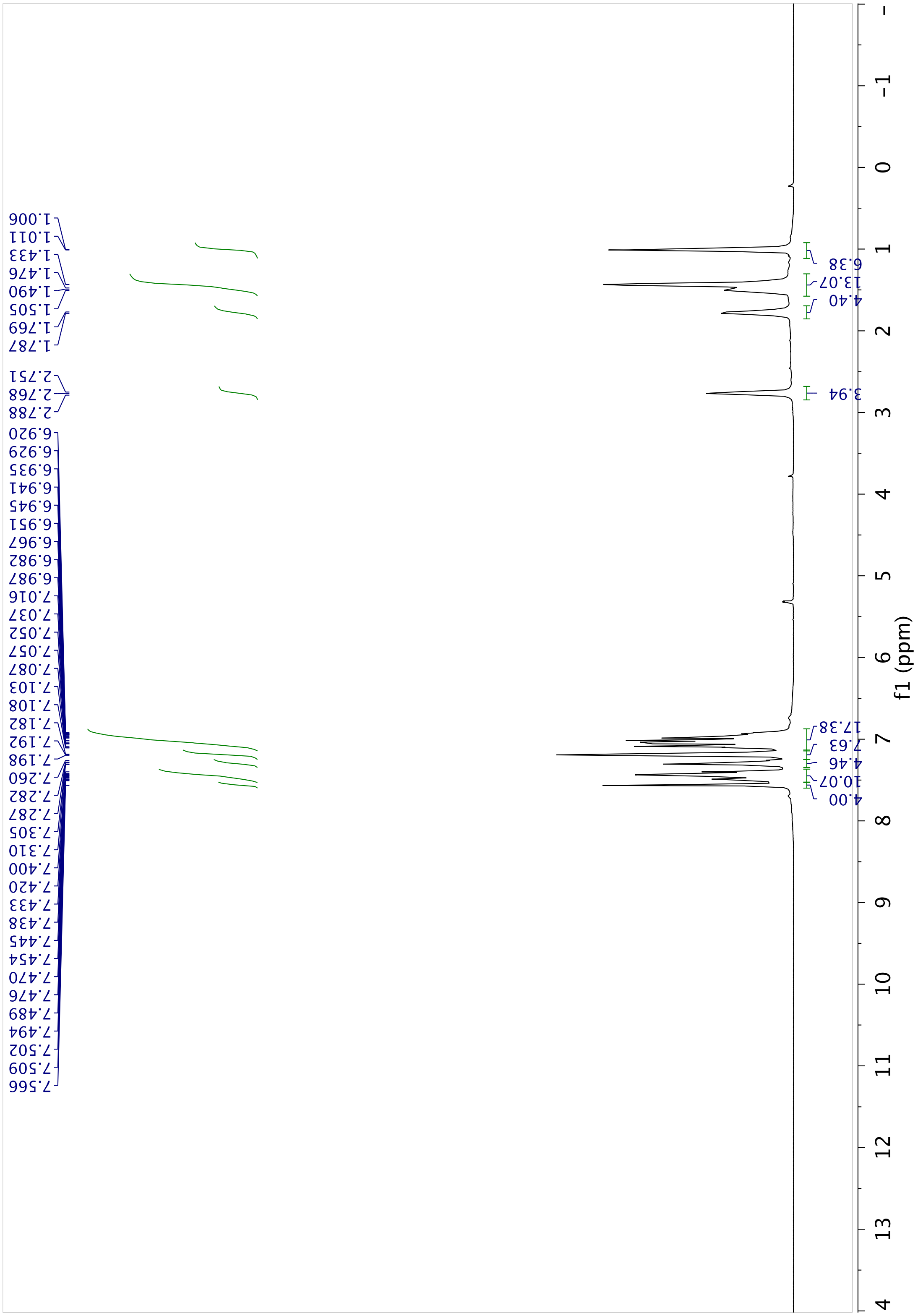
1.006
1.011
1.433
1.476
1.490
1.505
1.769
1.787
2.751
2.768
2.788
6.920
6.929
6.935
6.941
6.945
6.951
6.967
6.982
6.987
7.016
7.037
7.052
7.057
7.087
7.103
7.108
7.182
7.192
7.198
7.260
7.282
7.287
7.305
7.310
7.400
7.420
7.433
7.438
7.445
7.454
7.470
7.476
7.489
7.494
7.502
7.509
7.566
6.38
13.07
4.40
3.94
17.38
7.63
4.46
10.07
4.00
f1 (ppm)

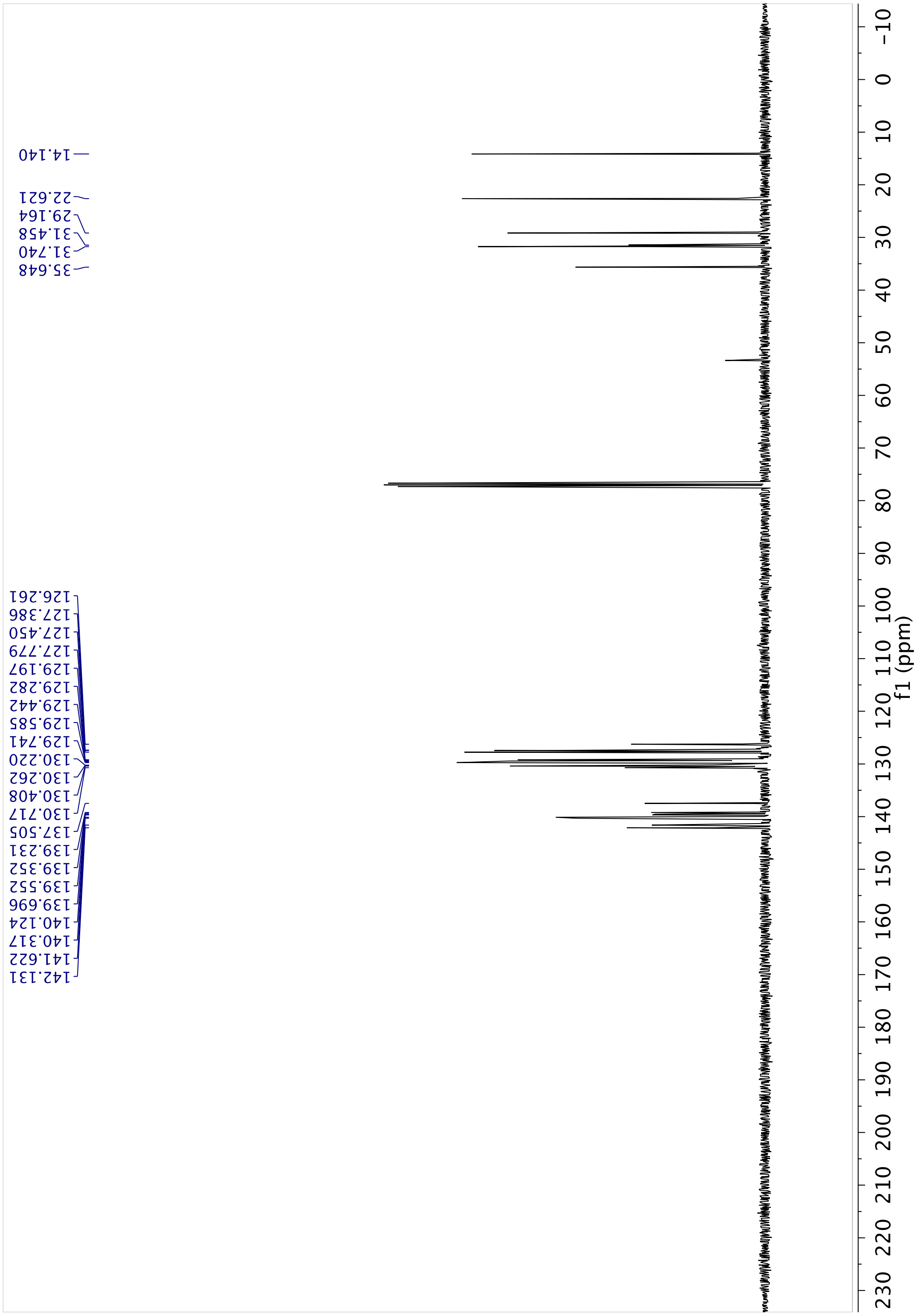
14.140
22.621
29.164
31.458
31.740
35.648
126.261
127.386
127.450
127.779
129.197
129.282
129.442
129.585
129.741
130.220
130.262
130.408
130.717
137.505
139.231
139.352
139.552
139.696
140.124
140.317
141.622
142.131
f1 (ppm)

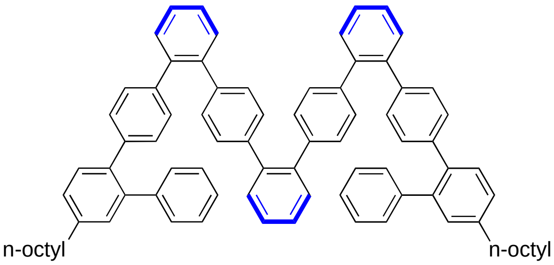

Oligophenylene **15d**

TLC (SiO_2): R_f = 0.71 (hexanes : Et_2O = 95:5).

^{1}H NMR: (400 MHz, $CDCl_3$): δ = 7.49 (s, 4H), 7.44-7.30 (m, 10H), 7.24-7.19 (m, 4H), 7.13-7.08 (m, 8H), 7.03-6.83 (m, 18H), 2.68 (t, *J* = 7.6 Hz, 4H), 1.70 (quin., *J* = 6.8 Hz , 4H), 1.47-1.324 (m, 20H), 0.91 (t, *J* = 5.5 Hz, 6H) ppm.

^{13}C NMR: (100 MHz, $CDCl_3$): δ = 142.2, 141.6, 140.3, 140.1, 139.7, 139.5, 139.3, 139.2, 137.5, 130.7, 130.4, 130.24, 130.21, 129.7, 129.6, 129.4, 129.31, 129.27, 129.18, 129.15, 127.8, 127.7, 127.5, 127.39, 127.36, 127.3, 126.3, 35.7, 31.9, 31.5, 29.5, 29.3, 24.6, 22.7, 14.1 ppm.

MS: (MALDI-TOF) Calculated for $C_{82}H_{60}$ [M^+] = 1044.5, Found 1044.7.

FTIR: (neat): 3022, 2970, 1434, 1267, 1186, 1167, 815, 793, 748, 681 cm^{-1}.

MP: >250 ºC

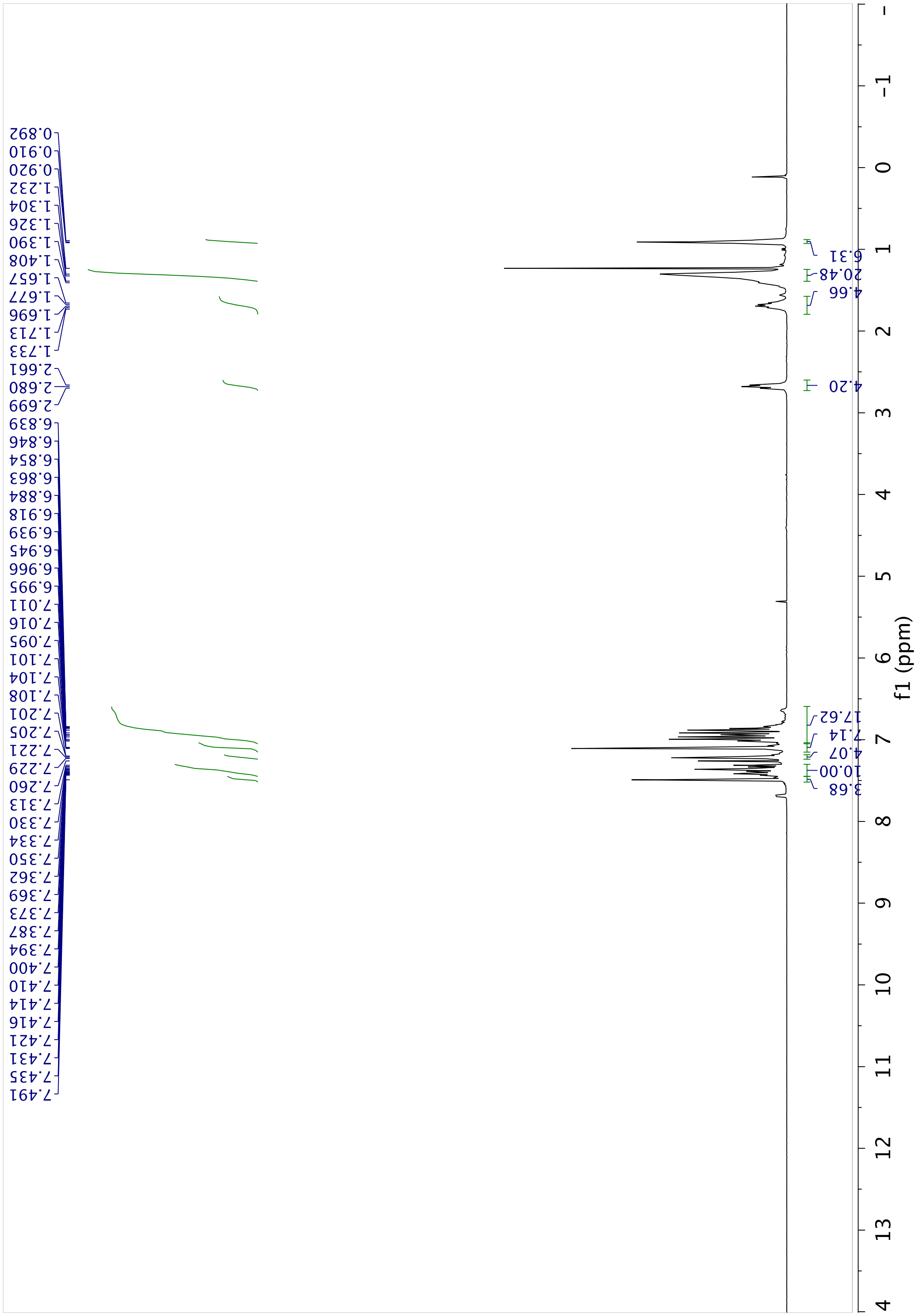
0.892
0.910
0.920
1.232
1.304
1.326
1.390
1.408
1.657
1.677
1.696
1.713
1.733
2.661
2.680
2.699
6.839
6.846
6.854
6.863
6.884
6.918
6.939
6.945
6.966
6.995
7.011
7.016
7.095
7.101
7.104
7.108
7.201
7.205
7.221
7.229
7.260
7.313
7.330
7.334
7.350
7.362
7.369
7.373
7.387
7.394
7.400
7.410
7.414
7.416
7.421
7.431
7.435
7.491
6.31
20.48
4.66
4.20
17.62
7.14
4.07
10.00
3.68
f1 (ppm)

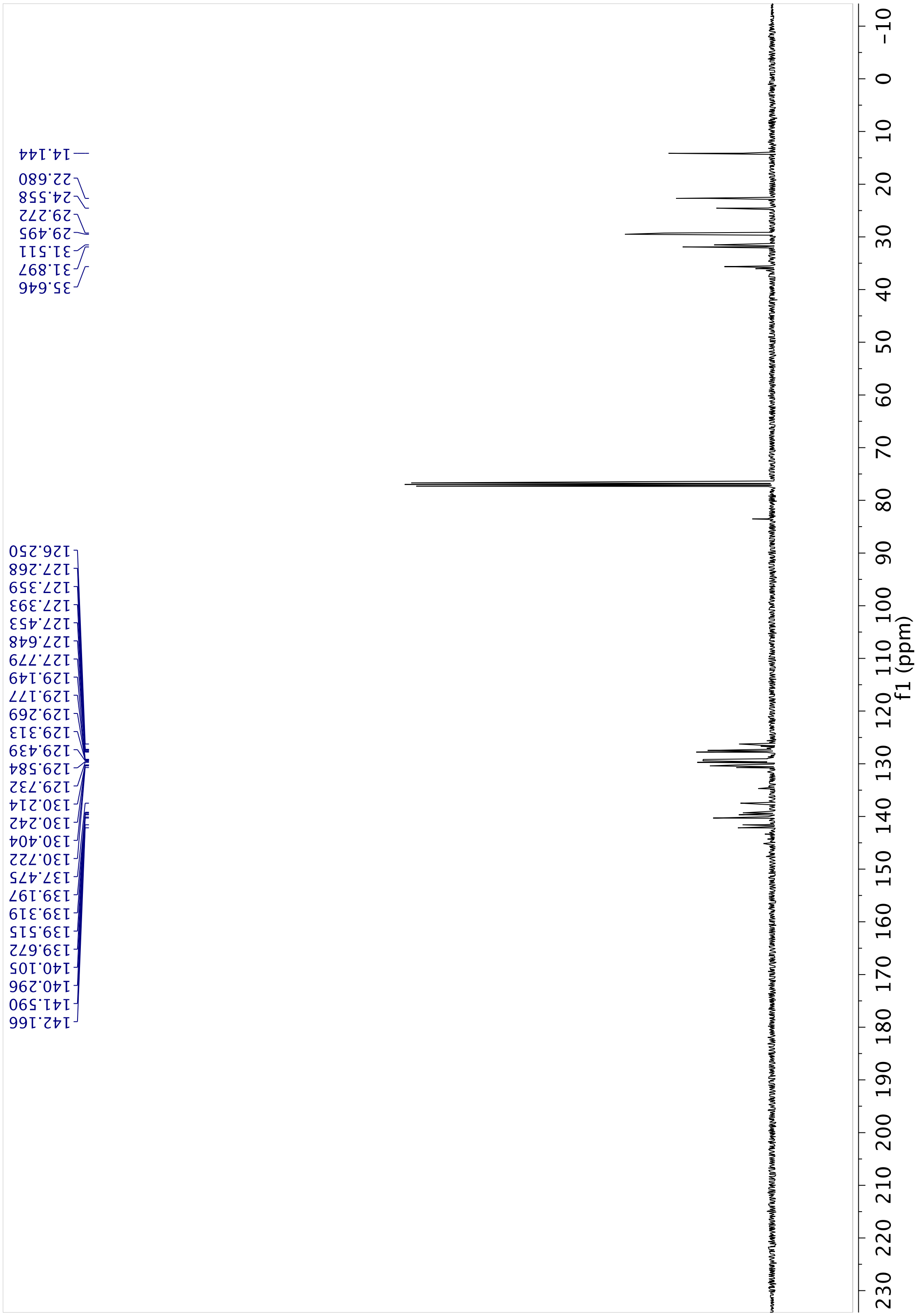
14.144
22.680
24.558
29.272
29.495
31.511
31.897
35.646
126.250
127.268
127.359
127.393
127.453
127.648
127.779
129.149
129.177
129.269
129.313
129.439
129.584
129.732
130.214
130.242
130.404
130.722
137.475
139.197
139.319
139.515
139.672
140.105
140.296
141.590
142.166
f1 (ppm)

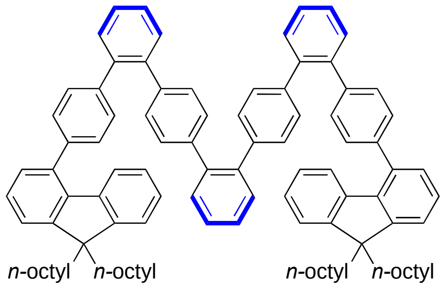

Oligophenylene **15e**

TLC (SiO_2): R_f = 0.89 (hexanes : Et_2O = 95:5).

^{1}H NMR: (400 MHz, $CDCl_3$): δ = 7.53-7.20 (m, 24H), 7.14-7.03 (m, 12H), 6.99-6.91 (m, 4H), 6.80 (t, *J* = 7.5 Hz, 1H), 1.97 (t, *J* = 8.4 Hz, 8H), 1.31-1.01 (m, 48H), 0.82 (t, *J* = 7.0 Hz, 12H) ppm.

^{13}C NMR: (100 MHz, $CDCl_3$): δ = 151.5, 151.1, 140.9, 140.6, 140.3, 140.2, 139.7, 139.6, 139.5, 137.9, 137.2, 130.54, 130.47, 130.45, 129.9, 129.53, 129.46, 128.76, 128.74, 127.6, 127.4, 126.6, 126.5, 126.4, 126.3, 126.1, 122.6, 122.5, 121.6, 54.3, 40.6, 31.8, 30.1, 29.3, 29.2, 23.7, 22.6, 14.1 ppm.

HRMS: **MS**: (MALDI-TOF) Calculated for $C_{100}H_{92}$ [M^+] = 1292.7, Found 1293.0.

FTIR: (neat): 3001, 2862, 1492, 1271, 1200, 1118, 984, 815, 793, 760, 748, 681 cm^{-1}.

MP: 210 °C (decomposed)

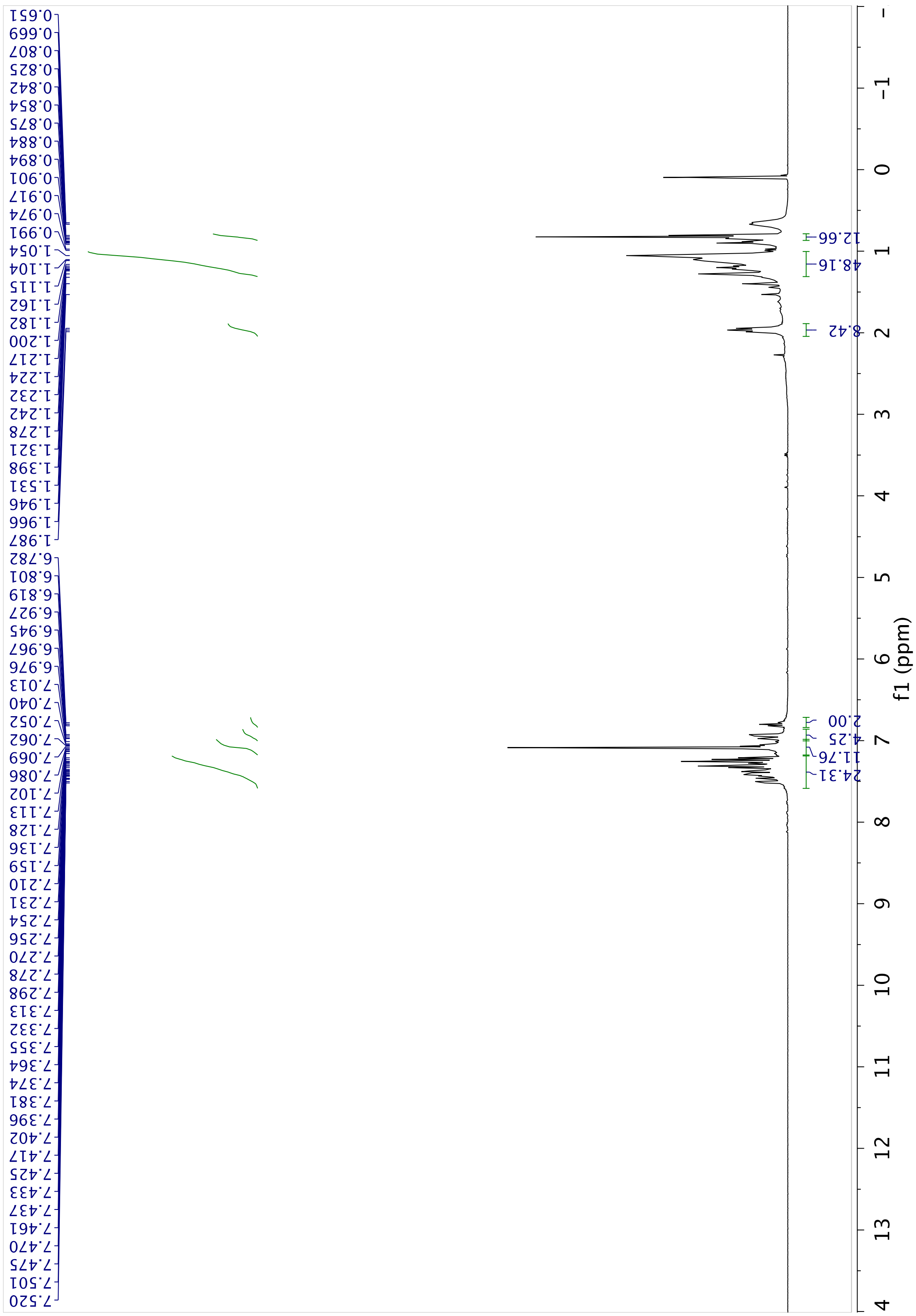

0.651
0.669
0.807
0.825
0.842
0.854
0.875
0.884
0.894
0.901
0.917
0.974
0.991
1.054
1.104
1.115
1.162
1.182
1.200
1.217
1.224
1.232
1.242
1.278
1.321
1.398
1.531
1.946
1.966
1.987
6.782
6.801
6.819
6.927
6.945
6.967
6.976
7.013
7.040
7.052
7.062
7.069
7.086
7.102
7.113
7.128
7.136
7.159
7.210
7.231
7.254
7.256
7.270
7.278
7.298
7.313
7.332
7.355
7.364
7.374
7.381
7.396
7.402
7.417
7.425
7.433
7.437
7.461
7.470
7.475
7.501
7.520
12.66
48.16
8.42
2.00
4.25
11.76
24.31
f1 (ppm)

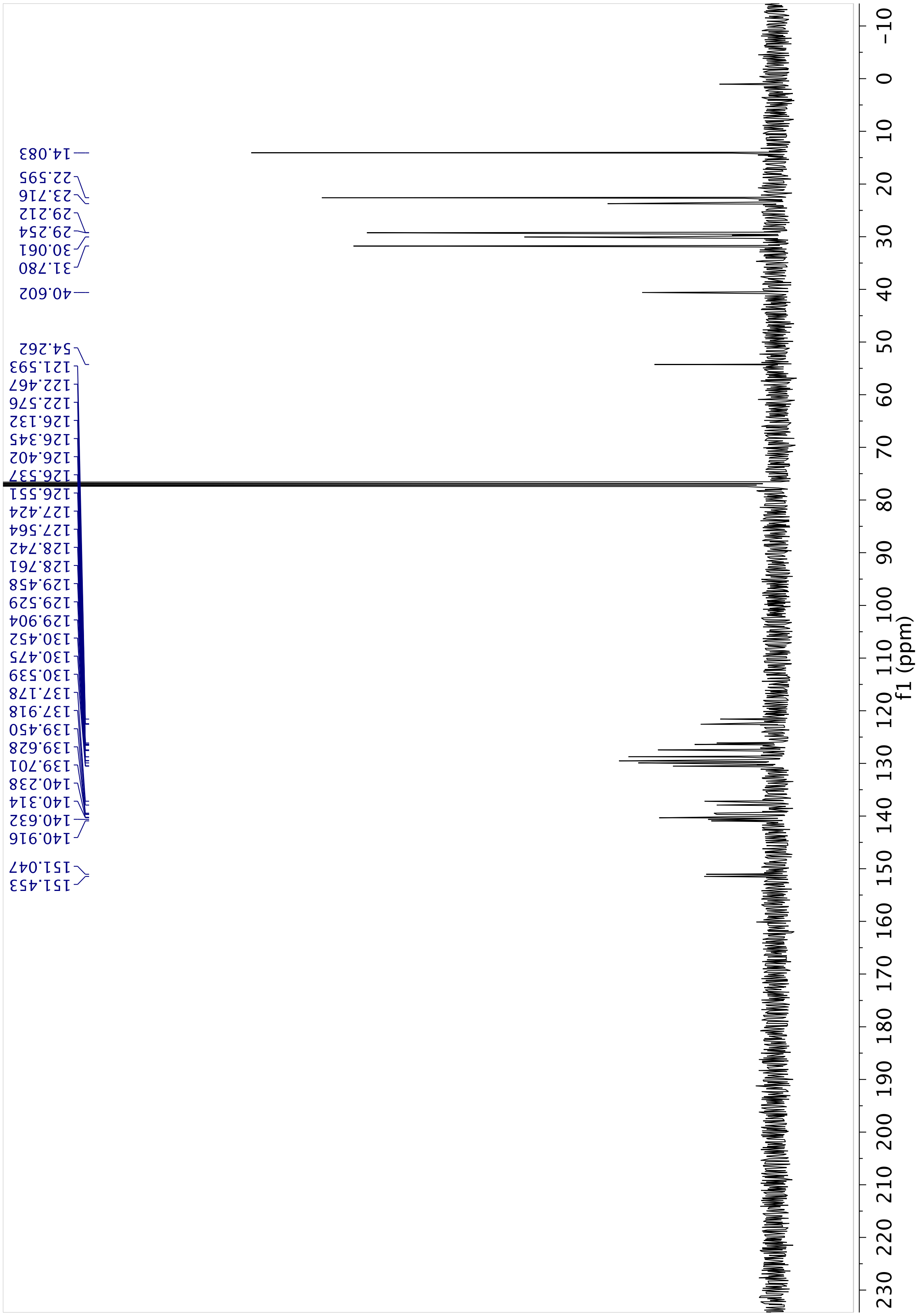
14.083
22.595
23.716
29.212
29.254
30.061
31.780
40.602
54.262
121.593
122.467
122.576
126.132
126.345
126.402
126.537
126.551
127.424
127.564
128.742
128.761
129.458
129.529
129.904
130.452
130.475
130.539
137.178
137.918
139.450
139.628
139.701
140.238
140.314
140.632
140.916
151.047
151.453
f1 (ppm)

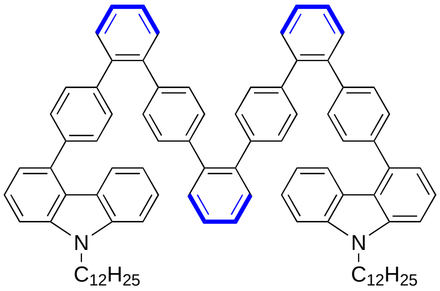

Oligophenylene **15f**

TLC (SiO_2): R_f = 0.72 (hexanes : Et_2O = 95:5).

^{1}H NMR: (400 MHz, $CDCl_3$): δ = 8.77-8.31 (m, 6H), 8.19 (s, 2H), 7.83-6.67 (m, 34H), 4.35 (t, *J* = 3.2 Hz, 4H), 1.99-1.88 (m, 4H), 1.56-1.25 (m, 36H), 0.98 (t, *J* = 6.8 Hz, 6H) ppm.

^{13}C NMR: (100 MHz, $CDCl_3$): δ = 140.7, 140.5, 140.3, 139.7, 139.63, 139.60, 137.5, 130.5, 130.4, 130.0, 129.9, 129.7, 129.6, 128.8, 128.7, 127.5, 125.6, 125.4, 125.3, 122.9, 122.4, 120.5, 120.4, 120.2, 118.7, 118.4, 108.7, 108.4, 107.5, 43.1, 32.0, 29.7, 29.6, 29.5, 29.4, 29.4, 29.0, 27.4, 24.9, 22.8, 14.3 ppm.

HRMS: (ESI+) Calculated for $C_{90}H_{92}N_2K$ [M+K$^+$] = 1239.6892, Found 1239.6894.

FTIR: (neat): 3010, 2909, 1598, 1392, 1231, 1118, 1003, 815, 738, 699 cm^{-1}.

MP: >250 ºC

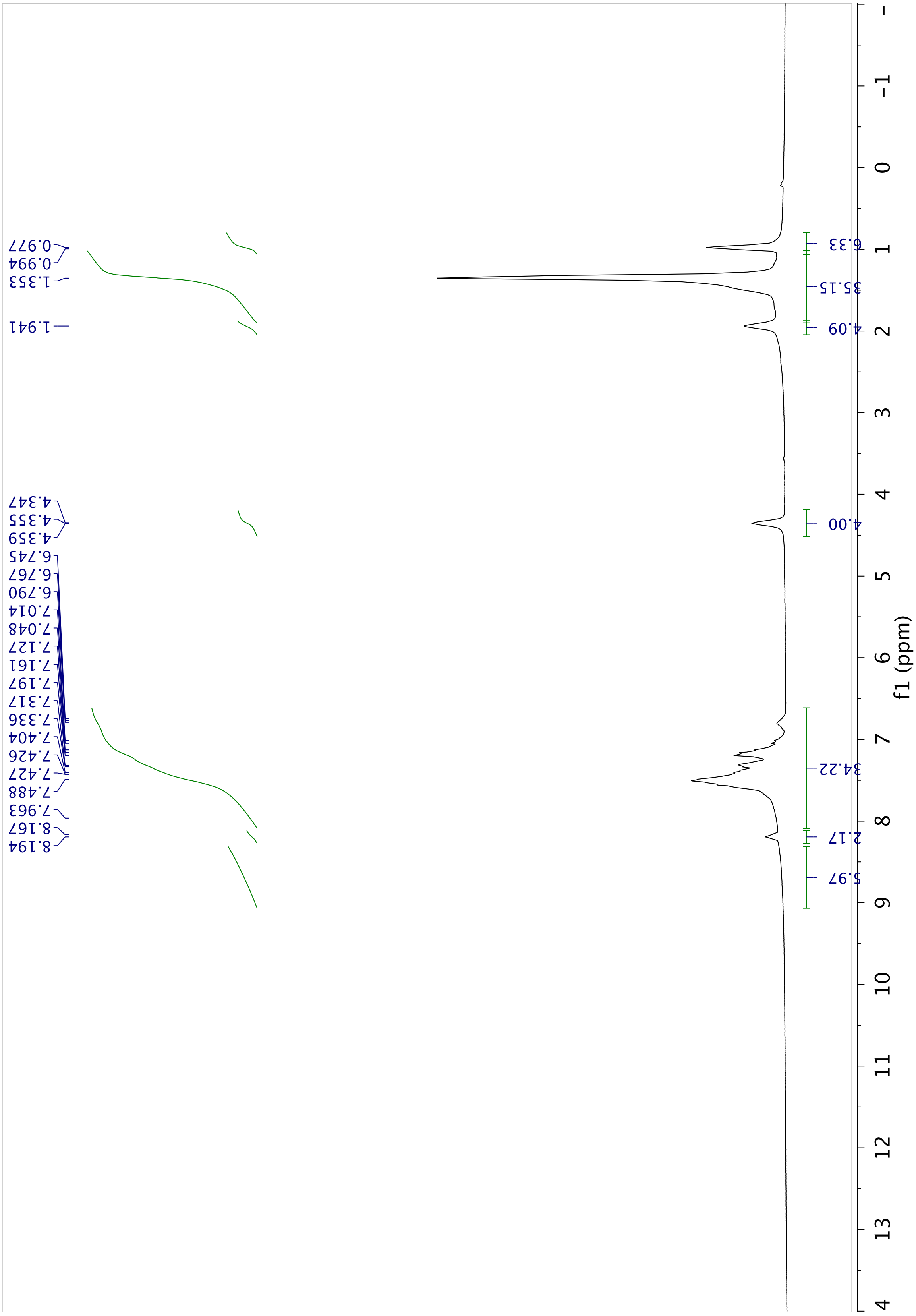
0.977
0.994
1.353
1.941
4.347
4.355
4.359
6.745
6.767
6.790
7.014
7.048
7.127
7.161
7.197
7.317
7.336
7.404
7.426
7.427
7.488
7.963
8.167
8.194
6.33
35.15
4.09
4.00
34.22
2.17
5.97
f1 (ppm)

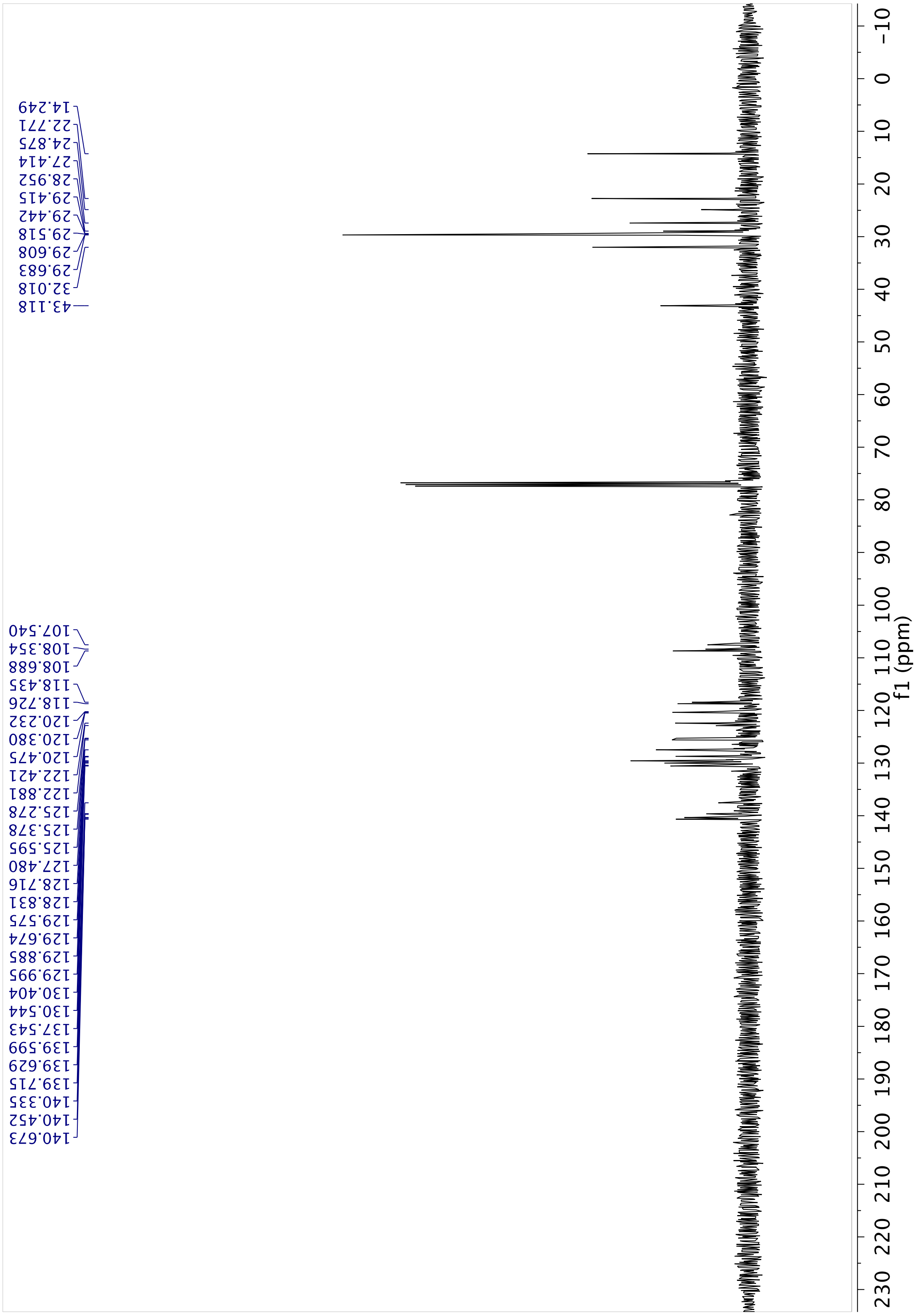
14.249
22.771
24.875
27.414
28.952
29.415
29.442
29.518
29.608
29.683
32.018
43.118
107.540
108.354
108.688
118.435
118.726
120.232
120.380
120.475
122.421
122.881
125.278
125.378
125.595
127.480
128.716
128.831
129.575
129.674
129.885
129.995
130.404
130.544
137.543
139.599
139.629
139.715
140.335
140.452
140.673
f1 (ppm)

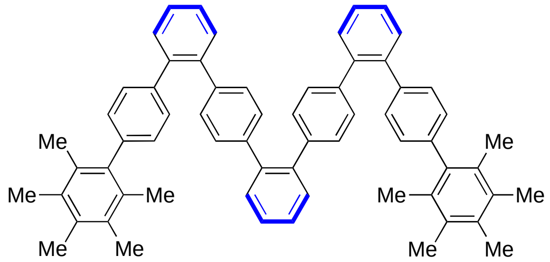

Oligophenylene **15g**

TLC (SiO_2): R_f = 0.90 (hexanes : Et_2O = 95:5).

^{1}H NMR: (400 MHz, $CDCl_3$): δ = 7.51-7.34 (m, 6H), 7.30-7.22 (m, 2H), 7.10-6.91 (m, 6H), 2.29 (s, 3H), 2.24 (s, 6H), 1.92 (t, *J* = 6.8 Hz, 6H) ppm.

^{13}C NMR: (125 MHz, $CDCl_3$): δ = 140.23, 140.16, 140.08, 139.8, 139.7, 139.5, 139.3, 139.1, 131.4, 130.9, 130.5, 130.3, 129.6, 129.5, 129.4, 129.2, 127.7, 127.5, 127.4, 126.3, 120.7, 18.3, 16.8, 16.6.
ppm.

HRMS: (MALDI-TOF): Calculated for $C_{64}H_{58}$ [M^+] = 826.45, Found 826.43.

FTIR: (neat): 2998, 1456, 1321, 1201, 1068, 1003, 819, 740 cm^{-1}.

MP: >250 °C

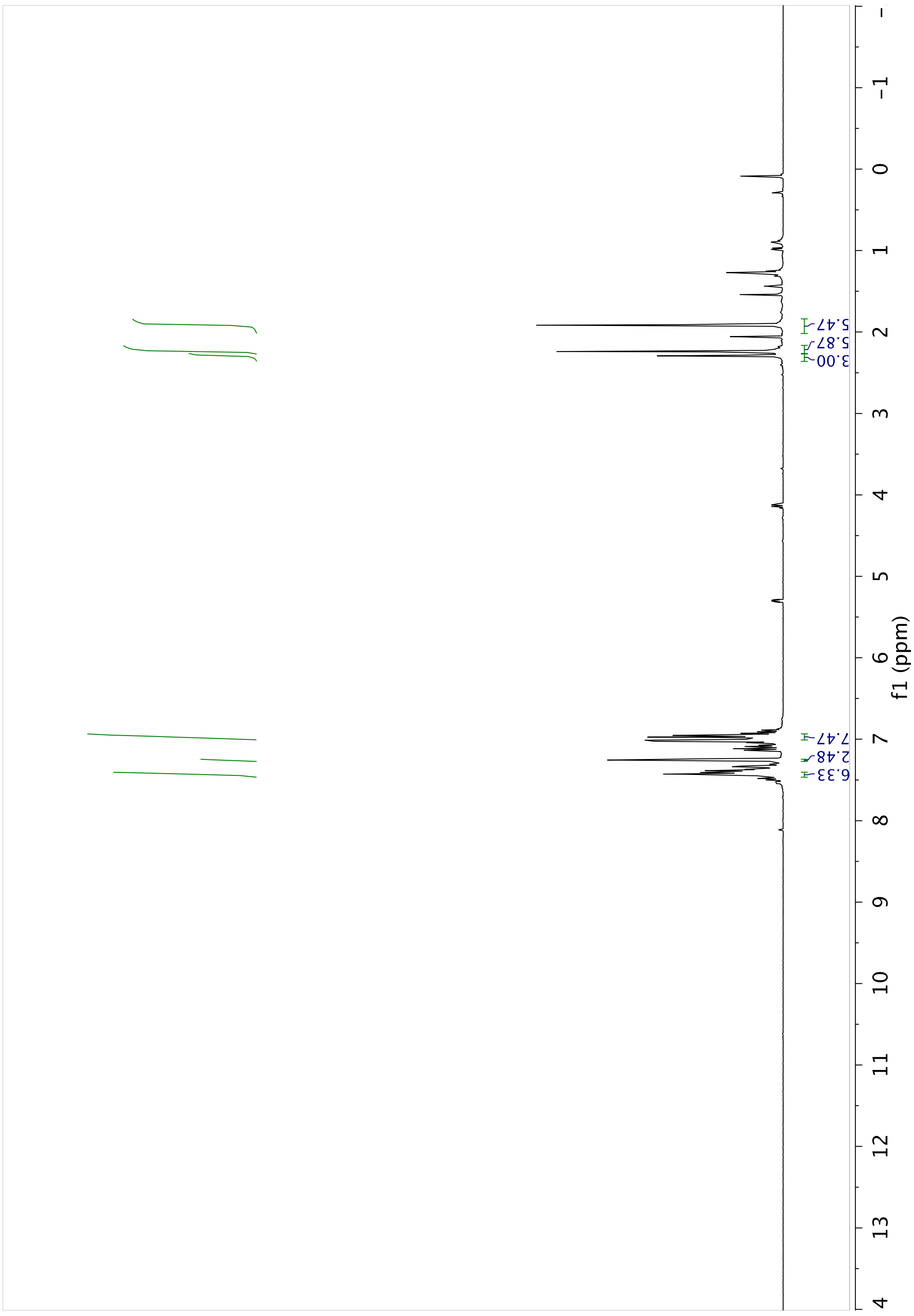
f1 (ppm)
5.47
5.87
3.00
7.47
2.48
6.33

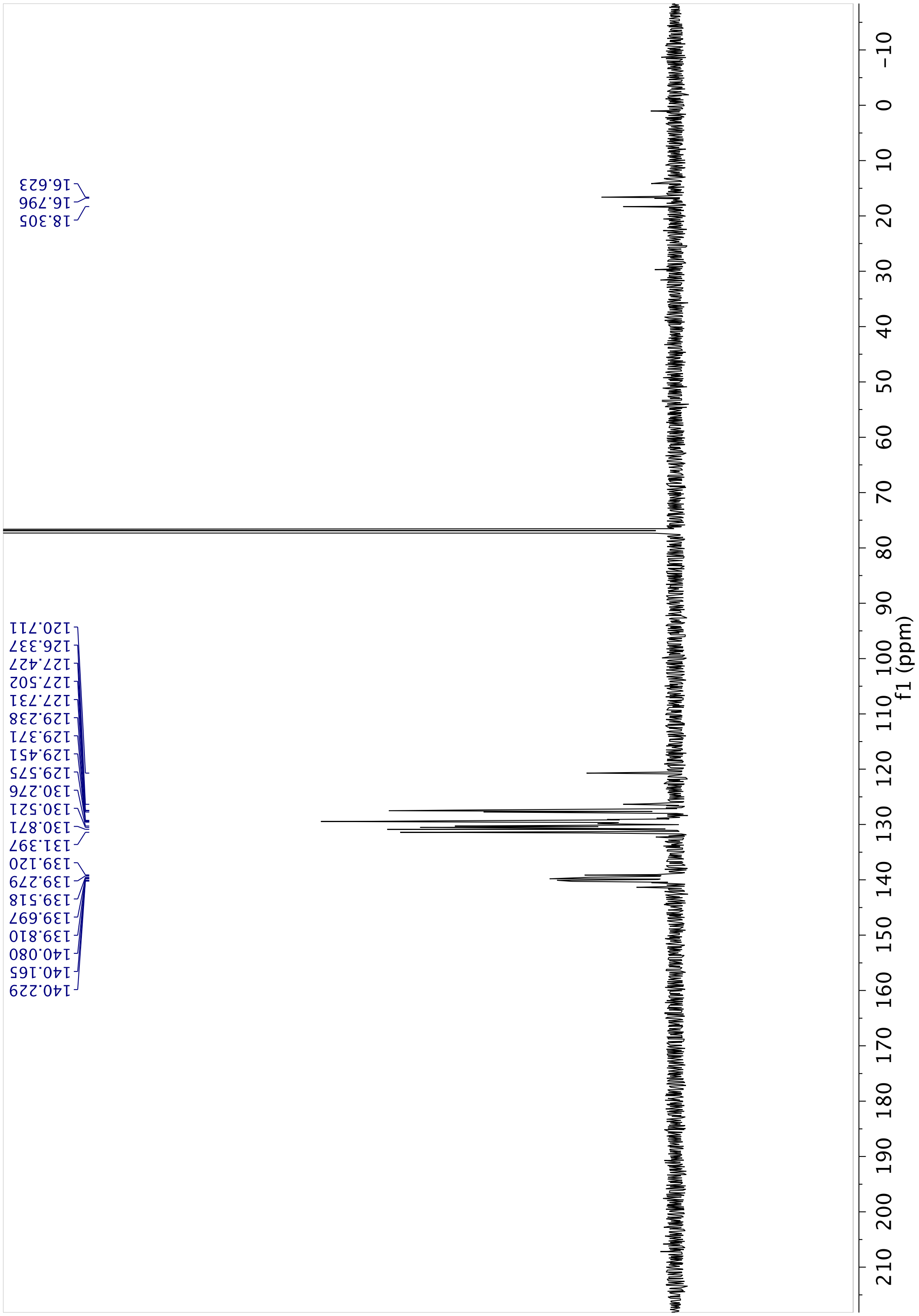
16.623
16.796
18.305
120.711
126.337
127.427
127.502
127.731
129.238
129.371
129.451
129.575
130.276
130.521
130.871
131.397
139.120
139.279
139.518
139.697
139.810
140.080
140.165
140.229
f1 (ppm)

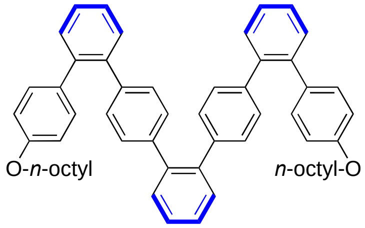

Oligophenylene **15h**

To a sealed tube with stir bar was added **11** (35 mg, 0.05 mmol, 100 mol%), CuI (2.0 mg, 0.01 mmol, 20 mol%), and LiOtBu (24 mg, 0.30 mmol, 600 mol%). 1-Octanol (0.1 mL, 0.5 M) was added. The tube was sealed with a septum and purged with argon for 5 min, before being quickly swapped with a screw cap. The reaction mixture was heated to 110 °C for 24 hr. The reaction was cooled to room temperature and concentrated onto silica, which was further subjected to flash column chromatography (hexanes: Et_2O, 90:10), to give **15h** (24 mg, 0.030 mmol, 60% yield) as a colorless, viscous oil.

TLC (SiO_2): R_f = 0.81 (hexanes : Et_2O = 95:5).

^{1}H NMR: (500 MHz, $CDCl_3$): δ = 7.45-7.38 (m, 12H), 7.07-6.99 (m, 12H), 6.78 (dd, *J* = 8.5, 1.9 Hz, 2H), 6.70 (d, *J* = 8.7 Hz, 4H), 3.78 (t, *J* = 6.6 Hz, 4H), 1.68 (quin., *J* = 6.7 Hz, 4H), 1.4-1.24 (m, 20H), 0.90 (t, *J* = 6.8 Hz, 1H) ppm.

^{13}C NMR: (125 MHz, $CDCl_3$): δ = 157.9, 140.2, 140.1, 139.4, 133.5, 130.9, 130.5, 130.4, 130.3, 129.5, 129.3, 128.7, 127.4, 127.0, 126.9, 126.5, 113.7, 67.8, 31.8, 29.39, 29.37, 29.2, 26.0, 22.7, 14.1 ppm.

HRMS: (ESI+) Calculated for $C_{58}H_{62}O_2Na$ [M+Na$^+$] = 813.4642, Found 813.4634.

FTIR: (neat): 2925, 2854, 1608, 1470, 1243, 1175, 907, 754 cm^{-1}.

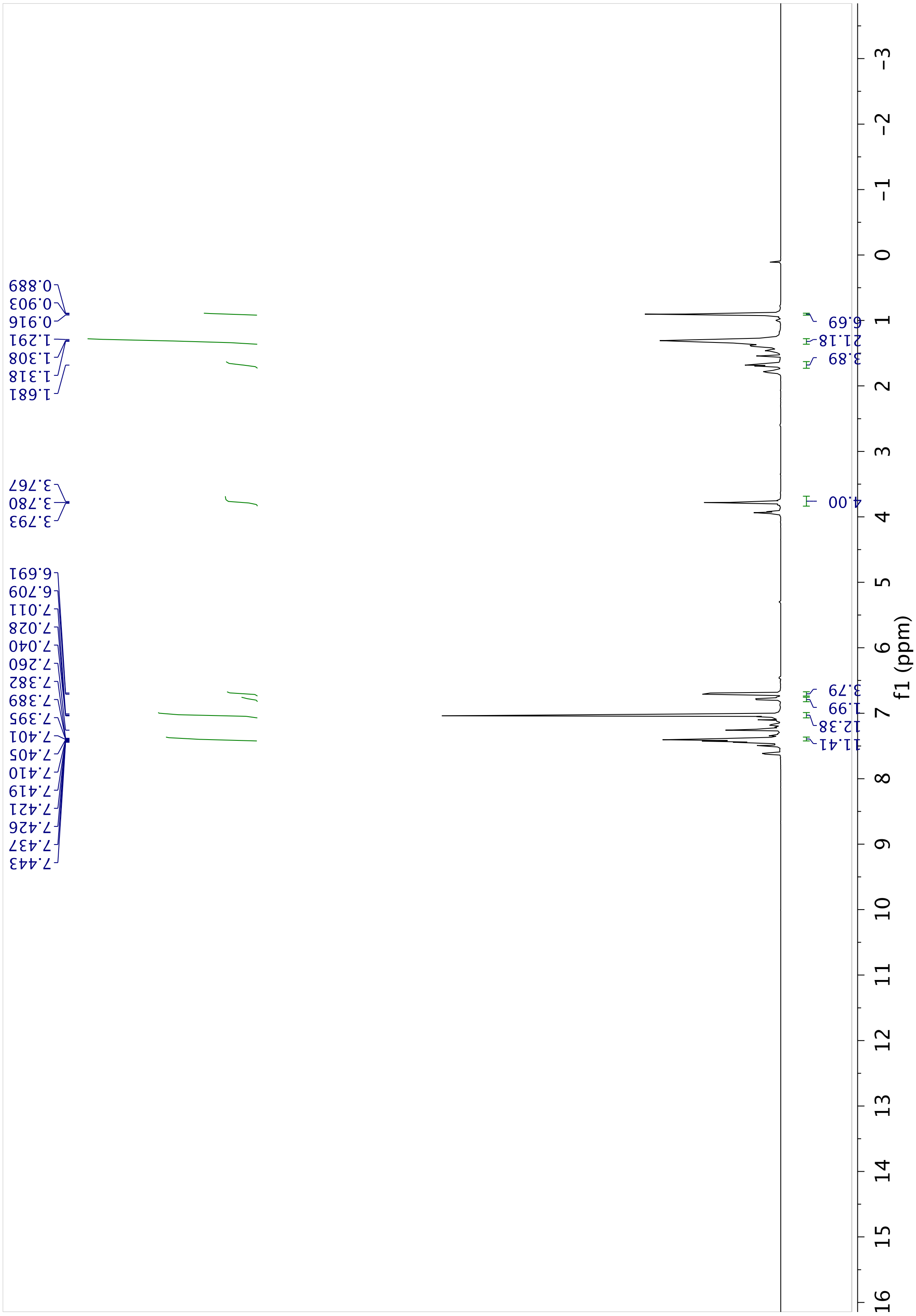

0.889
0.903
0.916
1.291
1.308
1.318
1.681
3.767
3.780
3.793
6.691
6.709
7.011
7.028
7.040
7.260
7.382
7.389
7.395
7.401
7.405
7.410
7.419
7.421
7.426
7.437
7.443
6.69
21.18
3.89
4.00
3.79
1.99
12.38
11.41
f1 (ppm)

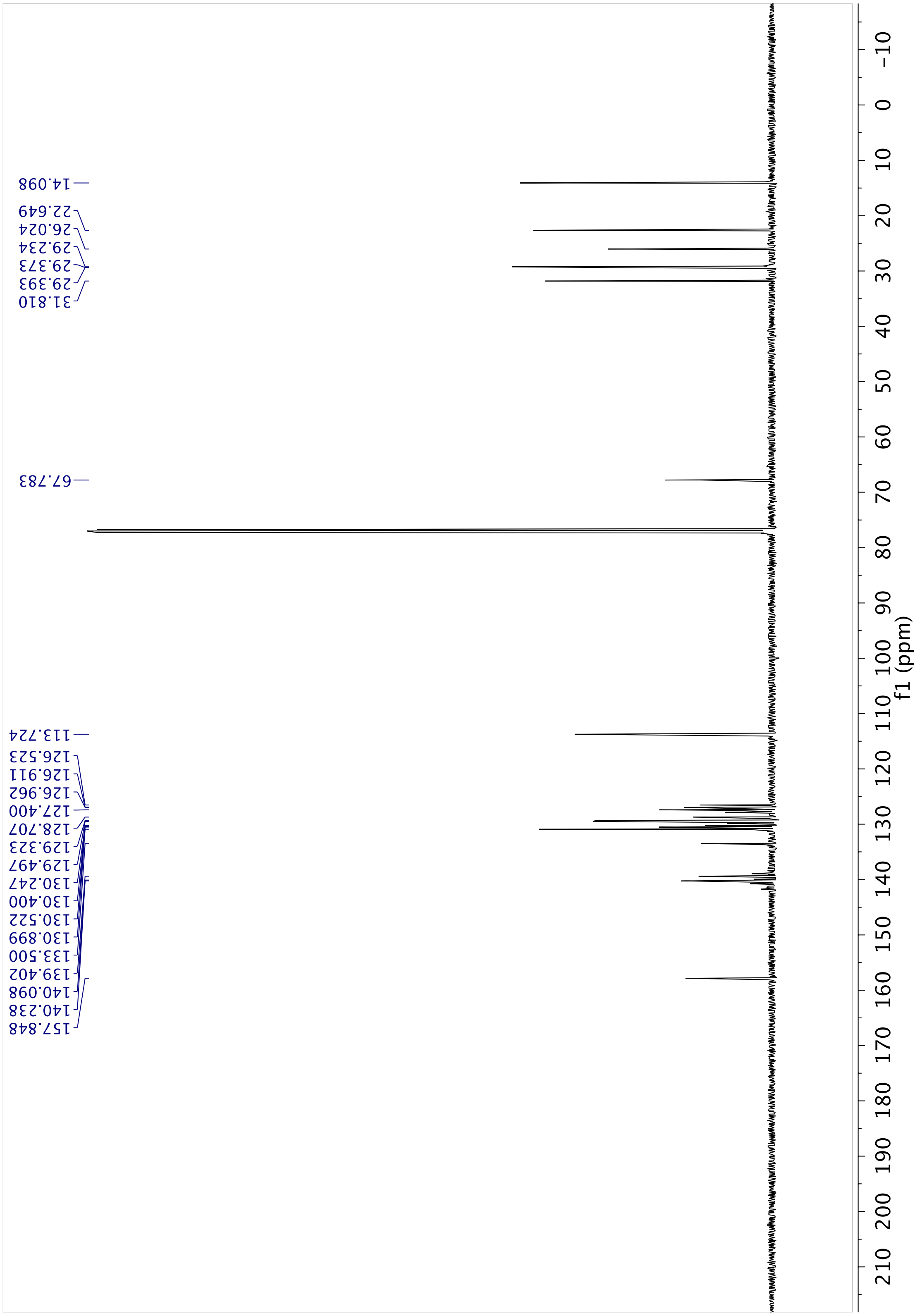
14.098
22.649
26.024
29.234
29.373
29.393
31.810
67.783
113.724
126.523
126.911
126.962
127.400
128.707
129.323
129.497
130.247
130.400
130.522
130.899
133.500
139.402
140.098
140.238
157.848
f1 (ppm)

Synthesis and Characterization of Nanographes 16a-h

General procedure for DDQ/ TfOH mediated Scholl oxidation (**16a** and **16h**):

To a vial capped with a septum under argon was added oligophenylene **15a** or **15h** (23mg (**15a**) or 24 mg (**15h**), 0.03 mmol, 100 mol%) and DCM (6 mL, 0.005 M). The vial was cooled to 0 ºC and then DDQ (20.4 mg, 0.09 mmol, 300 mol%; or 40.8 mg, 0.18 mmol, 600 mol%) was added followed by TfOH (8 μL, 0.09 mmol, 300 mol%; or 16 μL, 0.18 mmol, 600 mol%)The reaction was allowed to stir for 1-2 hr. H_2O (2 mL) was added, and the mixture was extracted with DCM (3 × 5 mL). The organic layers were dried with Na_2SO_4, filtered, and volatiles were removed to afford a brown residue, which was subjected to flash column chromatography (SiO_2, hexane:DCM, 9:1—1:1) to afford compound **16a** (15.6 mg, 0.021 mmol, 69% yield) or **16h** (10.3 mg, 0.020 mmol, 65% yield).

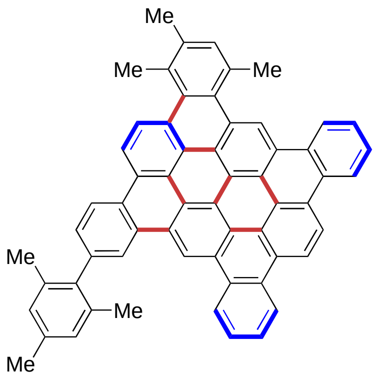

Nanographene **16a:**

TLC (SiO_2): R_f = 0.34 (hexanes : DCM = 50:50).

^{1}H NMR: (600 MHz, CD_2Cl_2): δ = 10.12 (s, 1H), 9.81 (s, 1H), 9.24 (t, *J* = 1.5 Hz, 1H), 9.12 (d, *J* = 8.2 Hz, 1H), 8.98 (dd, *J* = 24.0, 7.8 Hz, 1H), 8.89 (d, *J* = 7.7 Hz, 1H), 8.86-8.82 (m, 1H), 8.78 (t, *J* = 7.0 Hz, 2H), 8.75 (s, 1H), 8.46-8.23 (m, 1H), 7.91-7.85 (m, 2H), 7.82-7.75 (m, 2H), 7.68-7.62 (m, 2H), 7.55 (dt, *J* = 8.2, 1.7 Hz, 1H), 7.08 (s, 2H) ppm.

^{13}C NMR: (150 MHz, CD_2Cl_2): δ = 141.2, 139.5, 137.5, 136.8, 130.7, 130.6, 130.4, 130.2, 130.2, 130.1, 129.8, 129.7, 129.6, 129.3, 129.1, 128.9, 128.7, 128.4, 128.3, 128.2, 128.13, 128.09, 124.8, 124.5, 124.4, 124.1, 122.4, 122.1, 118.6, 118.4, 30.3, 21.36, 21.32 ppm.

MS: (MALDI-TOF) Calculated for $C_{60}H_{38}$ [M^+] = 758.30, Found 758.32.

FTIR: (neat): 2922, 2853, 1614, 1458, 1377, 1263, 1006, 752 cm^{-1}.

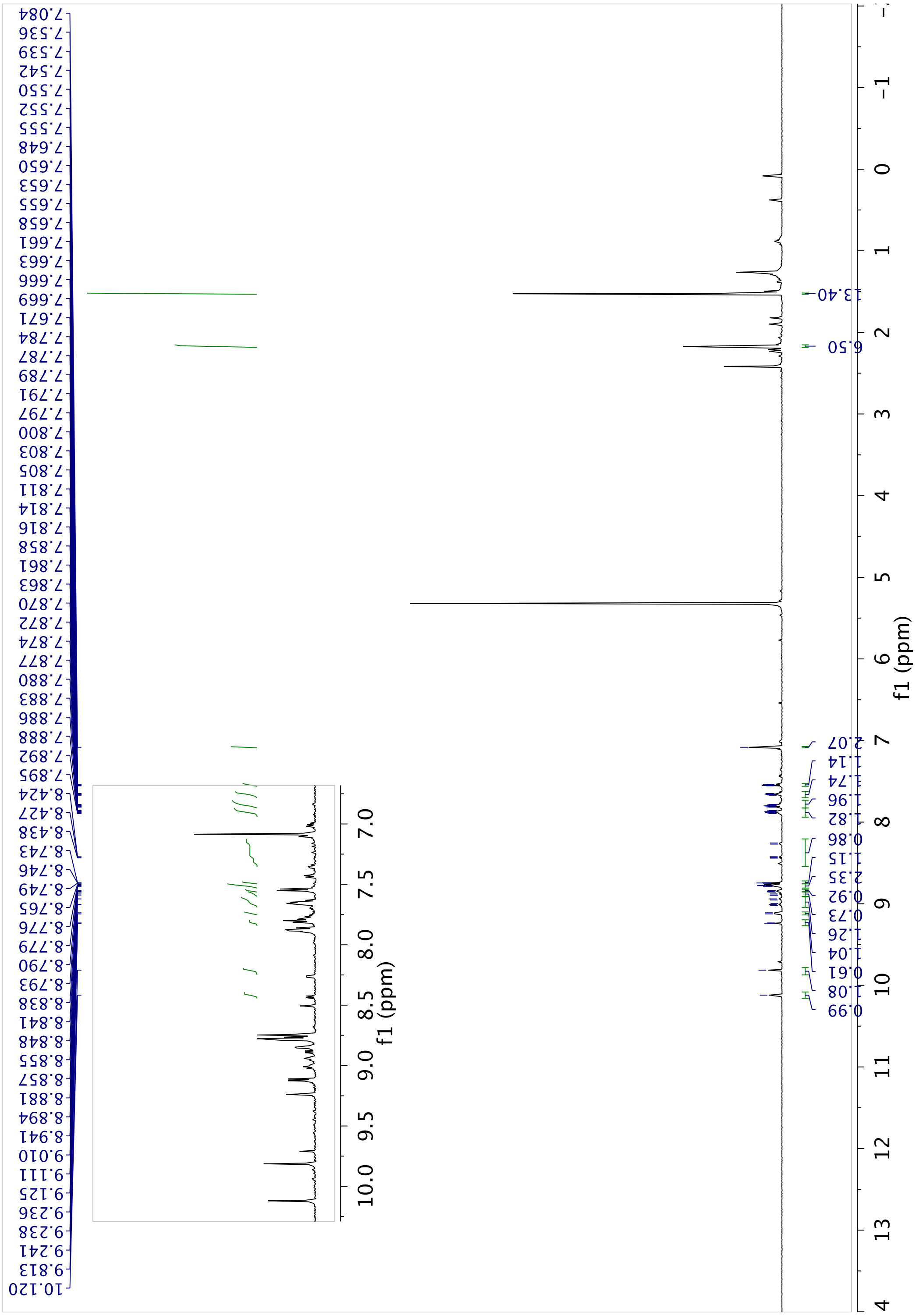
7.084
7.536
7.539
7.542
7.550
7.552
7.555
7.648
7.650
7.653
7.655
7.658
7.661
7.663
7.666
7.669
7.671
7.784
7.787
7.789
7.791
7.797
7.800
7.803
7.805
7.811
7.814
7.816
7.858
7.861
7.863
7.870
7.872
7.874
7.877
7.880
7.883
7.886
7.888
7.892
7.895
8.424
8.427
8.438
8.743
8.746
8.749
8.765
8.776
8.779
8.790
8.793
8.838
8.841
8.848
8.855
8.857
8.881
8.894
8.941
9.010
9.111
9.125
9.236
9.238
9.241
9.813
10.120
13.40
6.50
2.07
1.14
1.74
1.96
1.82
0.86
1.15
2.35
0.92
0.73
1.26
1.04
0.61
1.08
0.99
f1 (ppm)

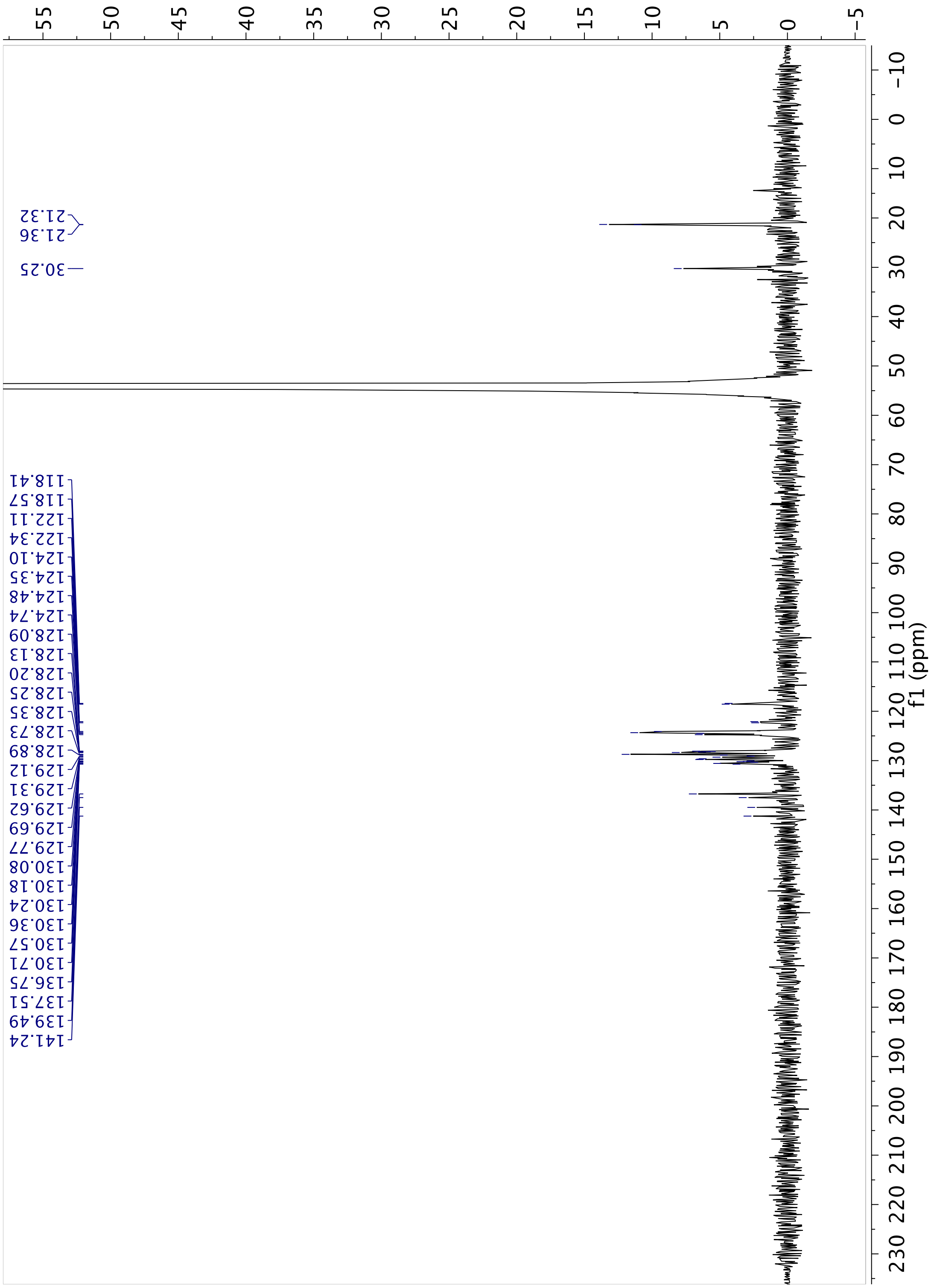
21.32
21.36
30.25
118.41
118.57
122.11
122.34
124.10
124.35
124.48
124.74
128.09
128.13
128.20
128.25
128.35
128.73
128.89
129.12
129.31
129.62
129.69
129.77
130.08
130.18
130.24
130.36
130.57
130.71
136.75
137.51
139.49
141.24
f1 (ppm)

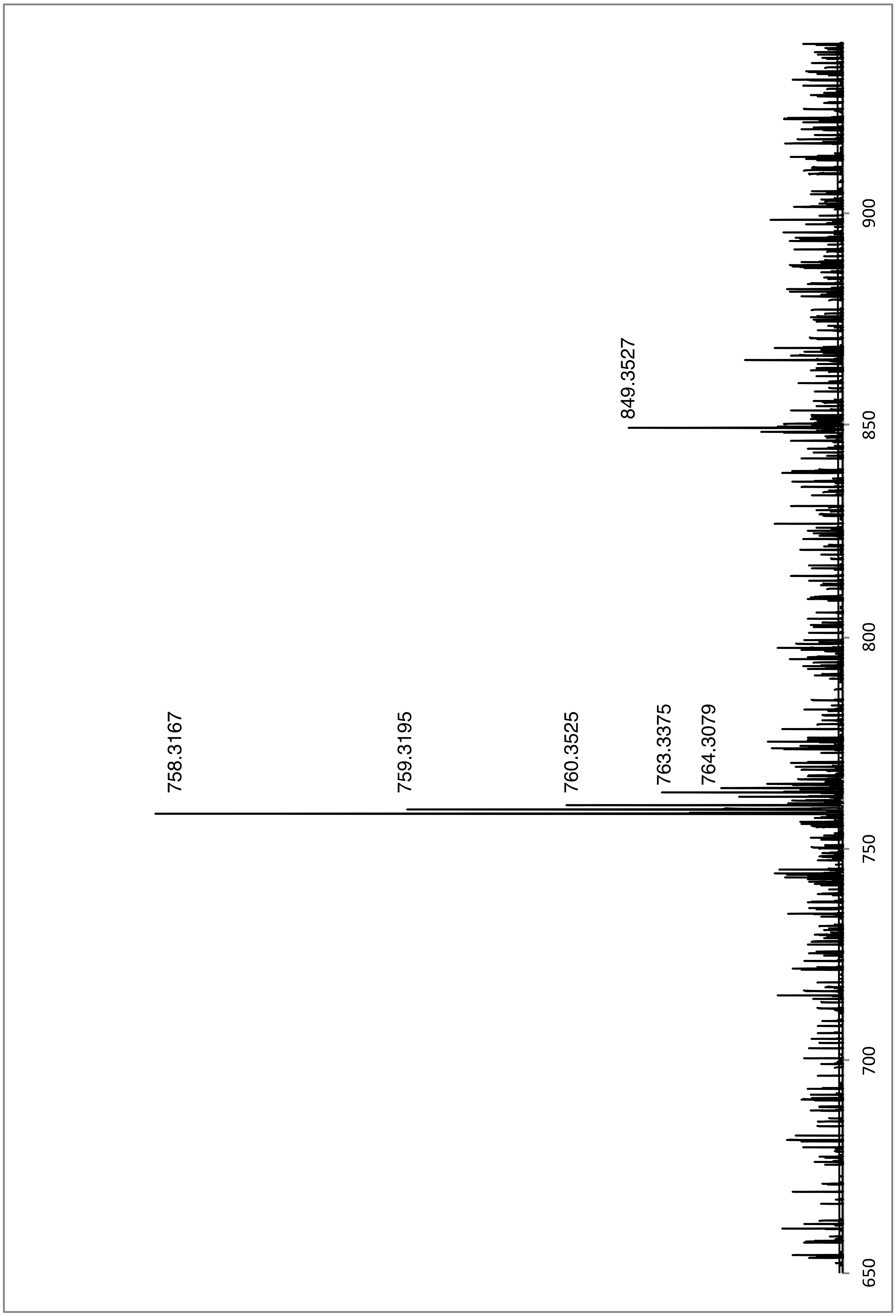
758.3167
759.3195
760.3525
763.3375
764.3079
849.3527
650
700
750
800
850
900

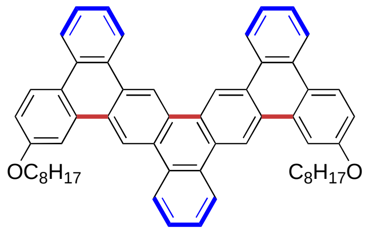

Nanographene **16h:**

TLC (SiO_2): R_f = 0.34 (hexanes : DCM = 40:60).

^{1}H NMR: (500 MHz, $CDCl_3$): δ = 8.63 (s, 4H), 7.59 (dd, *J* = 6.2, 3.2 Hz, 2H), 7.28 – 7.16 (m, 10H), 7.12 (t, *J* = 7.9 Hz, 1H), 6.83 (dd, *J* = 14.0, 7.5 Hz, 4H), 3.70 (t, *J* = 6.6 Hz, 4H), 1.61 (quin., *J* = 6.9 Hz, 4H), 1.31-1.14 (m, 20H), 0.82-0.76 (m, 6H) ppm.

^{13}C NMR: (150 MHz, CD_2Cl_2): δ = 158.78, 142.8, 141.6, 139.9, 139.8, 130.0, 129.4, 129.2, 129.0, 128.8, 128.1, 127.5, 126.8, 125.5, 125.4, 123.5, 122.4, 120.4, 116.1, 114.5, 113.6, 68.0, 31.9, 29.8, 29.4, 29.3, 26.1, 22.7, 14.2 ppm.

MS: (MALDI-TOF) Calculated for $C_{58}H_{56}O_2$ [M^+] = 784.4, Found 784.1.

FTIR: (neat): 2925, 2855, 1599, 1467, 1245, 1198, 1044, 700 cm^{-1}.

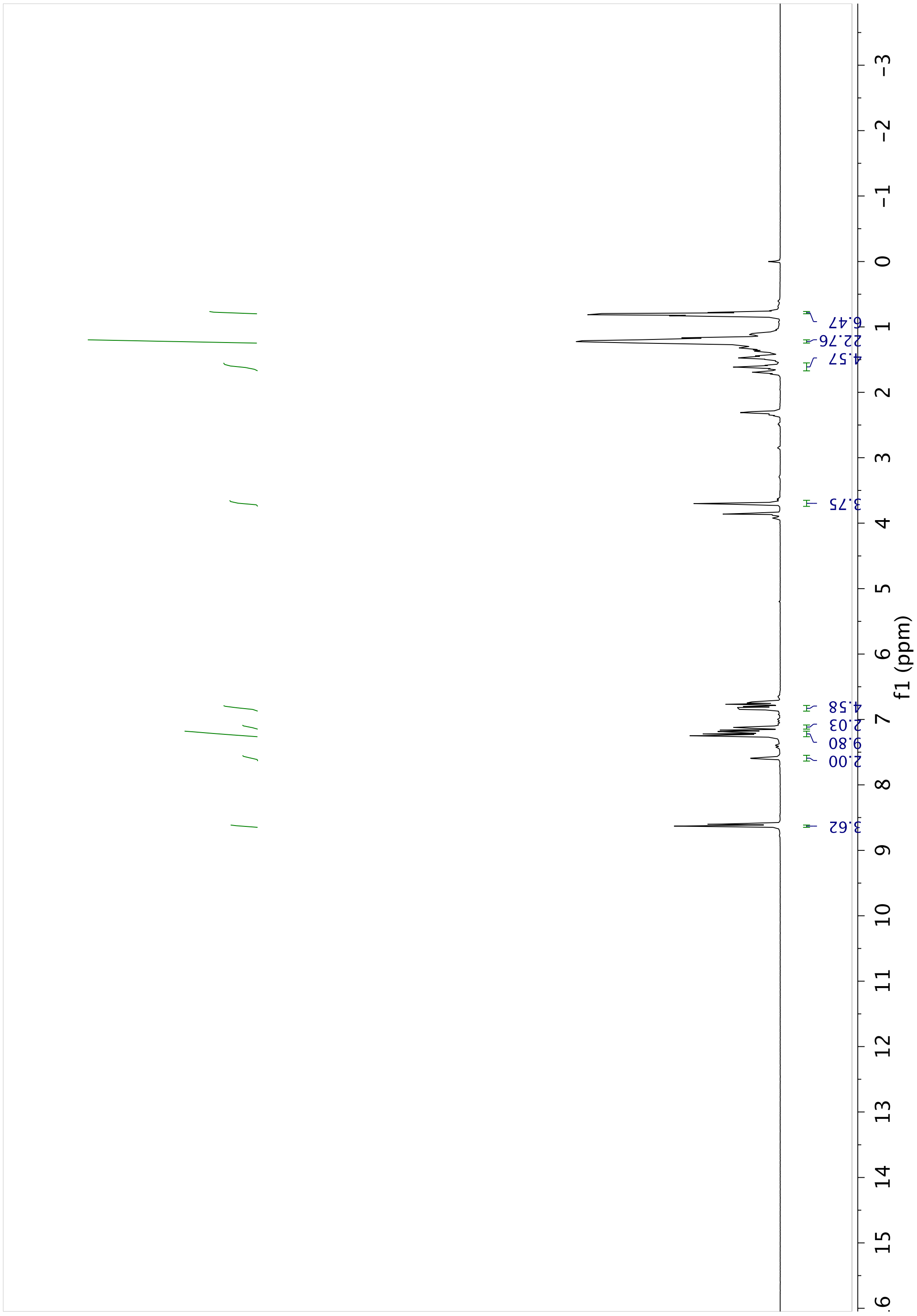
6.47
22.76
4.57
3.75
4.58
2.03
9.80
2.00
3.62
f1 (ppm)

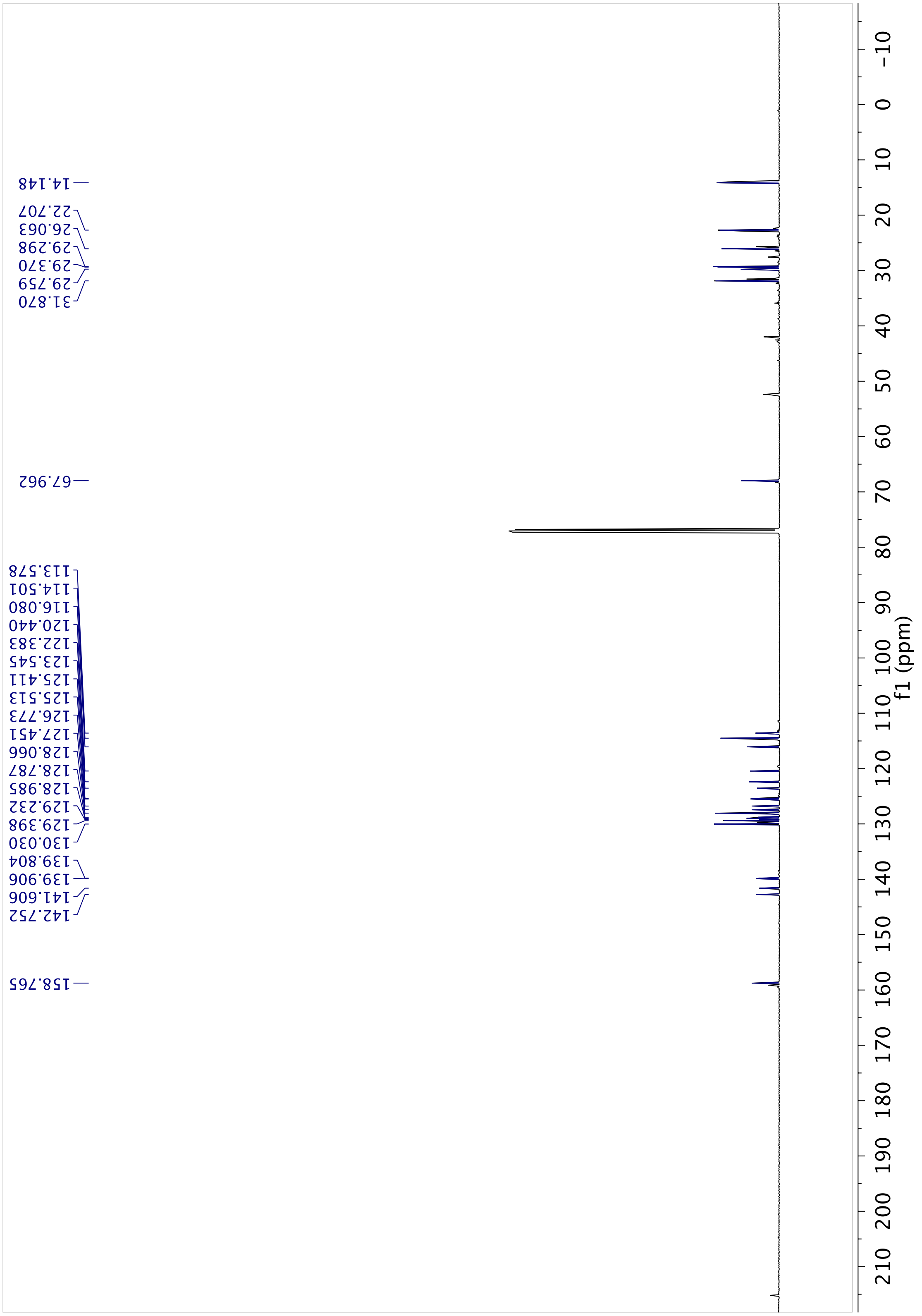
14.148
22.707
26.063
29.298
29.370
29.759
31.870
67.962
113.578
114.501
116.080
120.440
122.383
123.545
125.411
125.513
126.773
127.451
128.066
128.787
128.985
129.232
129.398
130.030
139.804
139.906
141.606
142.752
158.765
f1 (ppm)

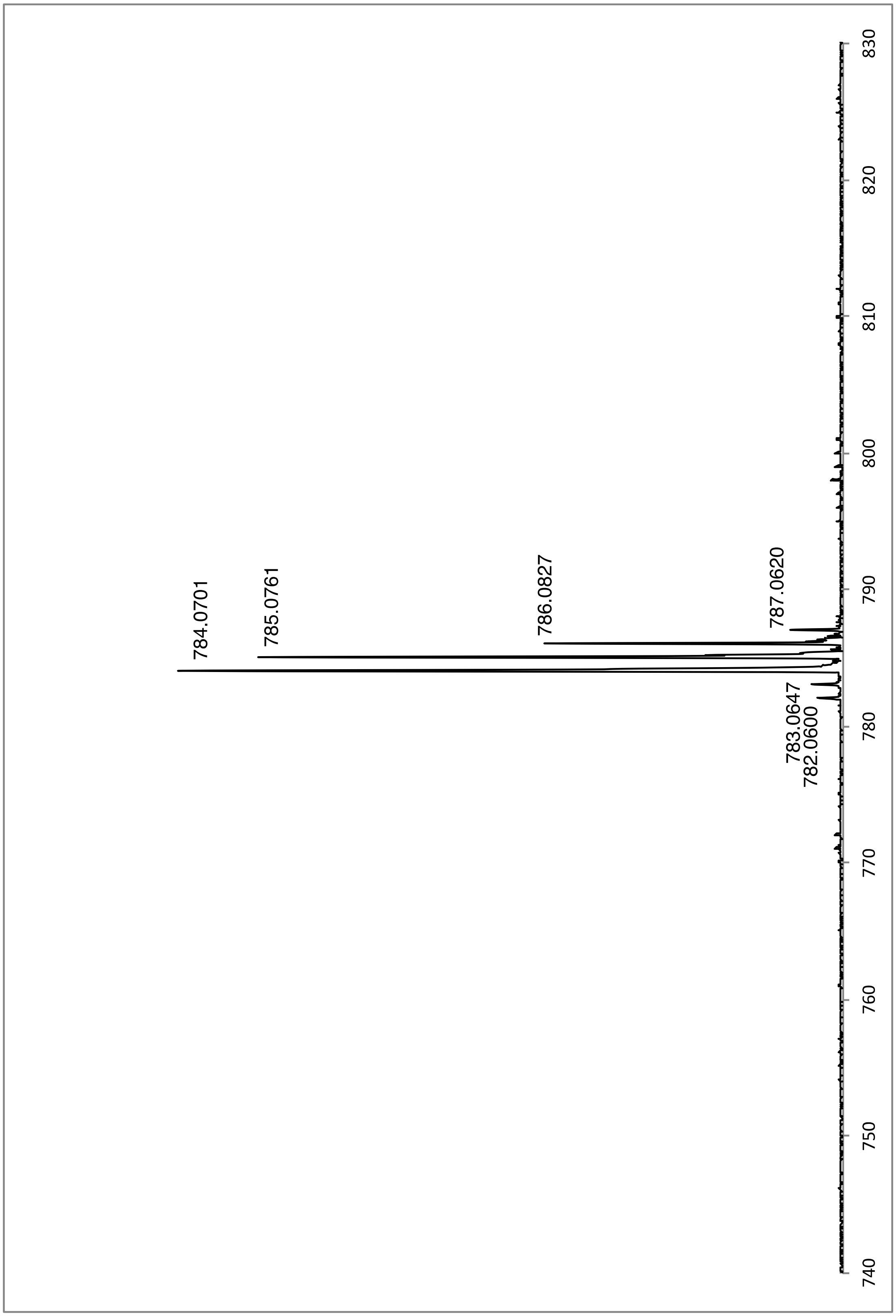
784.0701
785.0761
786.0827
787.0620
783.0647
782.0600
740
750
760
770
780
790
800
810
820
830

General procedure for $FeCl_3$ mediated Scholl oxidation:

To a vial capped with a septum under argon was added oligophenylene **15b-g** (0.03 mmol, 100 mol%), 4 Å molecular sieves (20 mass equivalents), and DCM (6 mL, 0.005 M). The vial was cooled to 0 °C and then a solution of $FeCl_3$ (2M in $MeNO_2$, 0.6mL, 4000 mol%) was added dropwise. The reaction was allowed to stir for 1-2 hr. If the product was soluble, the reaction mixture was concentrated onto silica and purified by flash column chromatography (SiO_2, 5 cm, $CHCl_3$). If insoluble, 1 mL MeOH was added and the reaction was allowed to stand without stirring. The mixture was then decanted to remove molecular sieves. The residual sieves were washed twice more with DCM and decanted. The combined organic layers were subject to vacuum filtration and the filtrate was washed with DCM (5 mL) and MeOH (3 × 15ml), to provide the desired product.

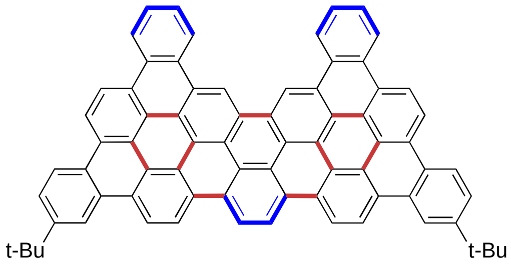

Nanographene **16b:**

TLC (SiO_2): R_f = 0.13 ($CHCl_3$).

^{1}H NMR: (500 MHz, $CDCl_3$): δ = 9.00 (s, 2H), 8.94-8.82 (m, 2H), 8.77 (s, 2H), 8.73 (d, *J* = 8.7 Hz, 1H), 8.69-8.55 (m, 8H), 8.17 (d, *J* = 31.6 Hz, 2H), 7.97-7.85 (m, 2H), 7.80 (dd, *J* = 8.5, 1.5 Hz, 4H), 7.58 (dd, *J* = 8.8, 1.8 Hz, 2H), 1.55 (s, 18H) ppm.

MS: (MALDI-TOF) Calculated for $C_{74}H_{44}$ [M^+] = 932.3, Found 932.7.

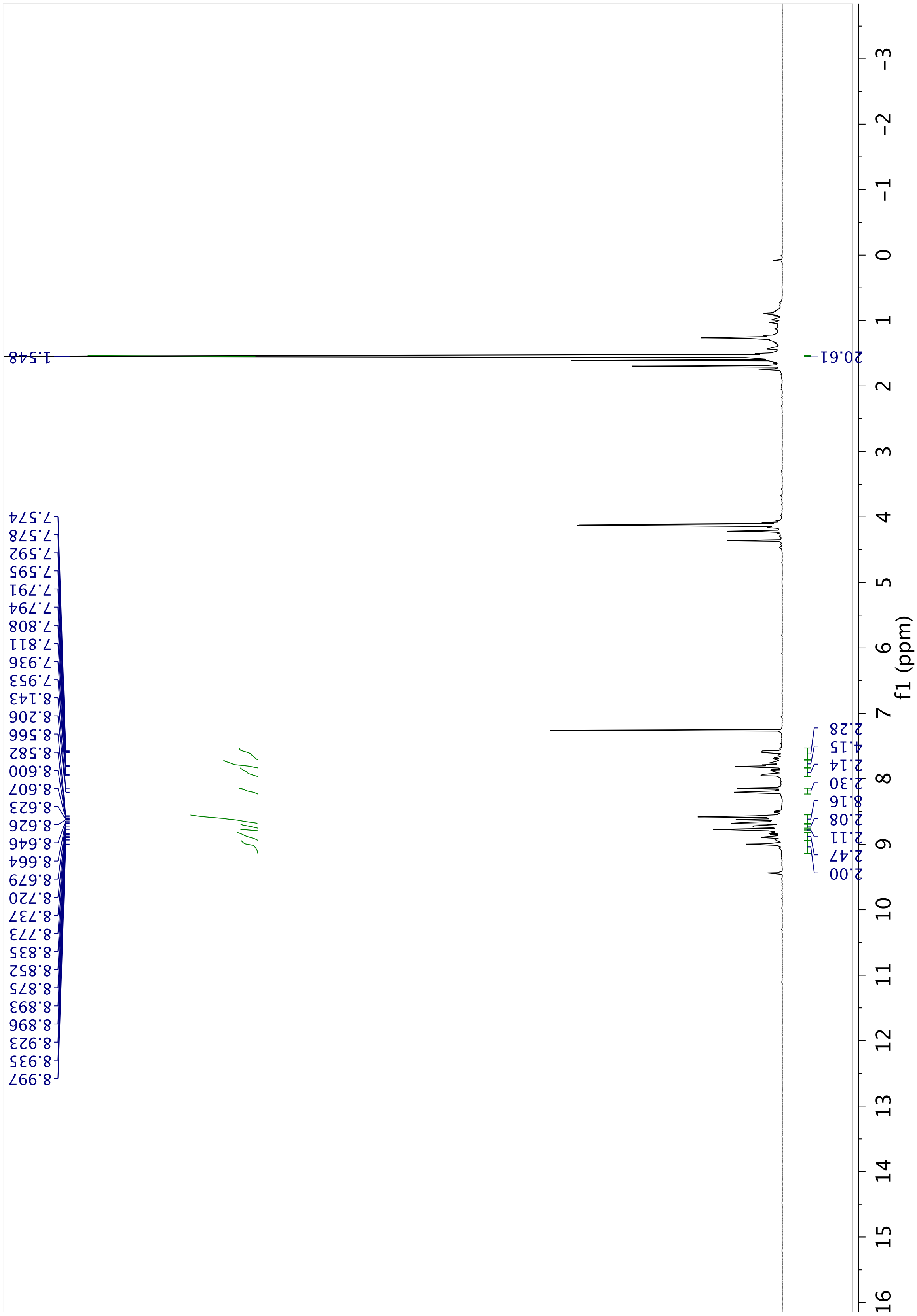

1.548
7.574
7.578
7.592
7.595
7.791
7.794
7.808
7.811
7.936
7.953
8.143
8.206
8.566
8.582
8.600
8.607
8.623
8.626
8.646
8.664
8.679
8.720
8.737
8.773
8.835
8.852
8.875
8.893
8.896
8.923
8.935
8.997
20.61
2.28
4.15
2.14
2.30
8.16
2.08
2.11
2.47
2.00
f1 (ppm)

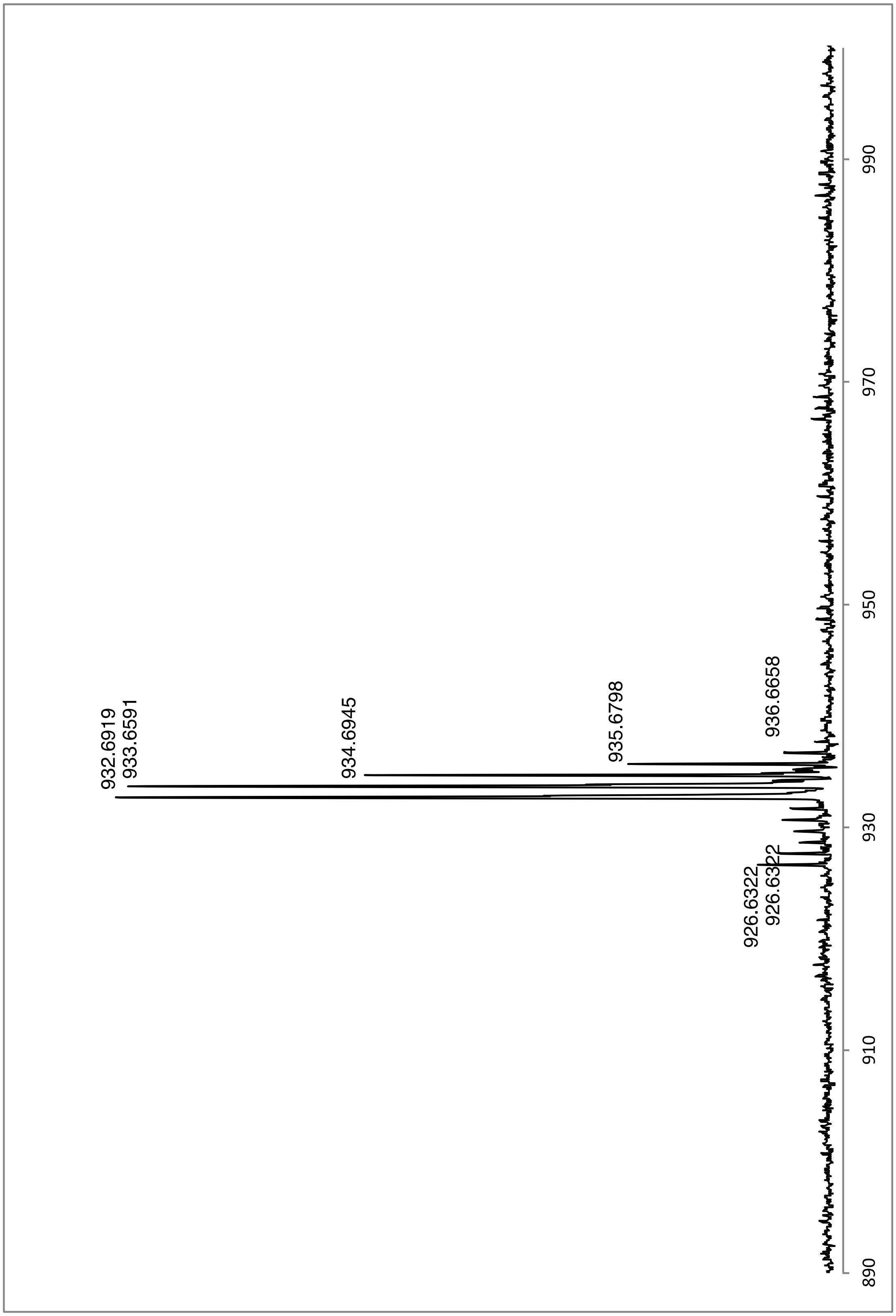
932.6919
933.6591
934.6945
935.6798
936.6658
926.6322
926.6322
890
910
930
950
970
990

Nanographene **16c:**

MS: (MALDI-TOF) Calculated for $C_{78}H_{52}$ [M^+] = 988.41, Found 988.34.

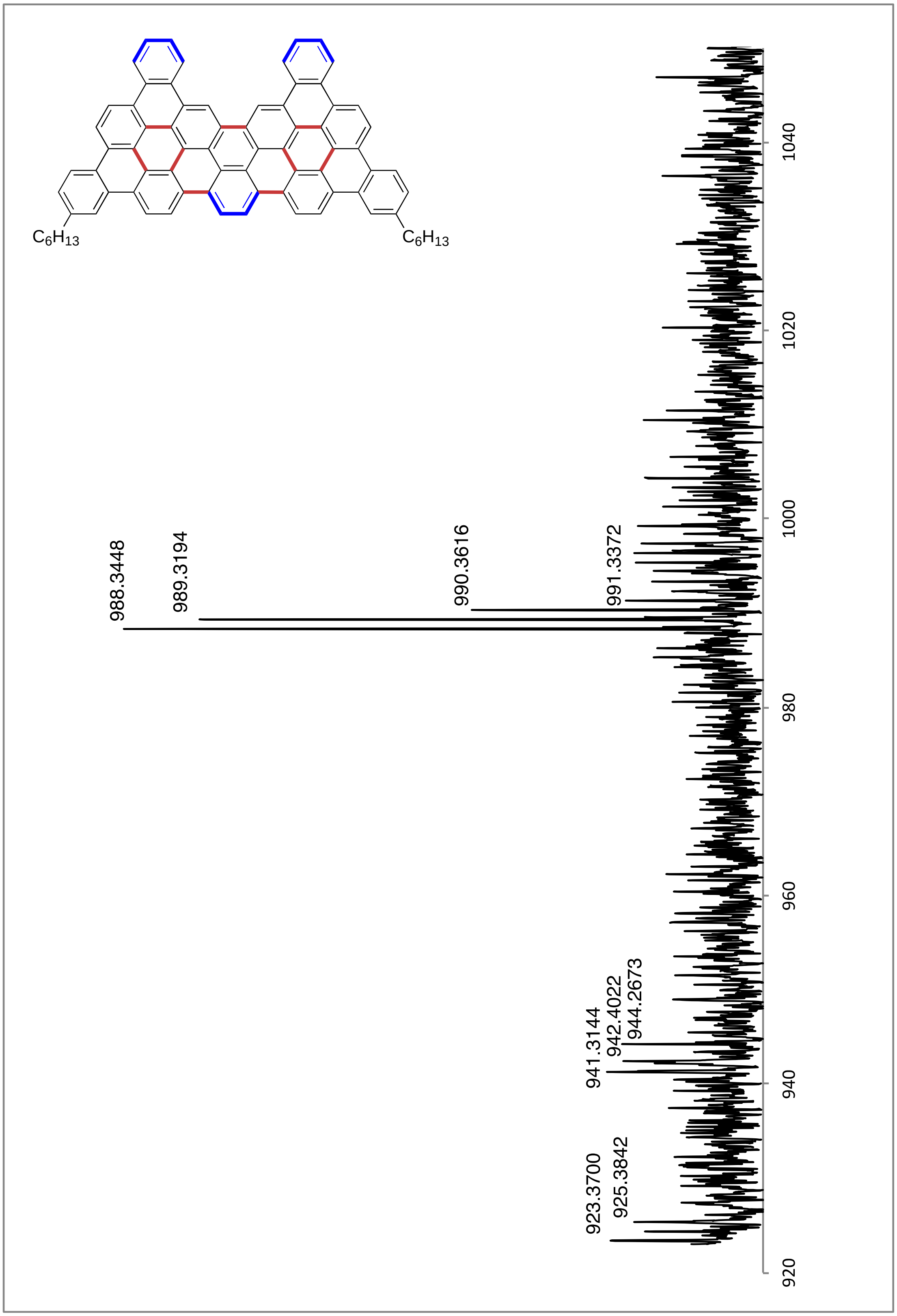

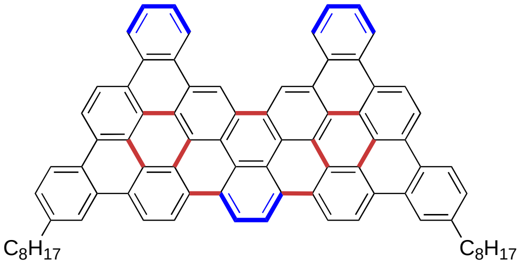

Nanographene **16d:**

TLC (SiO_2): R_f = 0.09 ($CHCl_3$).

^{1}H NMR: (500 MHz, $CDCl_3$): δ = 8.95 (s, 2H), 8.76 (d, *J* = 8.5 Hz, 2H), 8.73-8.66 (m, 4H), 8.61 (d, *J* = 8.6 Hz, 2H), 8.56 (s, 2H), 8.48 (s, 2H), 8.16 (d, *J* = 22.3 Hz, 2H), 7.97 (d, *J* = 8.4 Hz, 2H), 7.79-7.60 (m, 4H), 7.54 (t, *J* = 9.3 Hz, 2H), 2.90 (t, *J* = 9.2 Hz, 4H), 1.88-1.77 (m, 4H), 1.51-1.10 (m, 20H), 0.85 (t, *J* = 6.8 Hz, 6H) ppm.

MS: (MALDI-TOF) Calculated for $C_{82}H_{60}$ [M^+] = 1044.5, Found 1044.9.

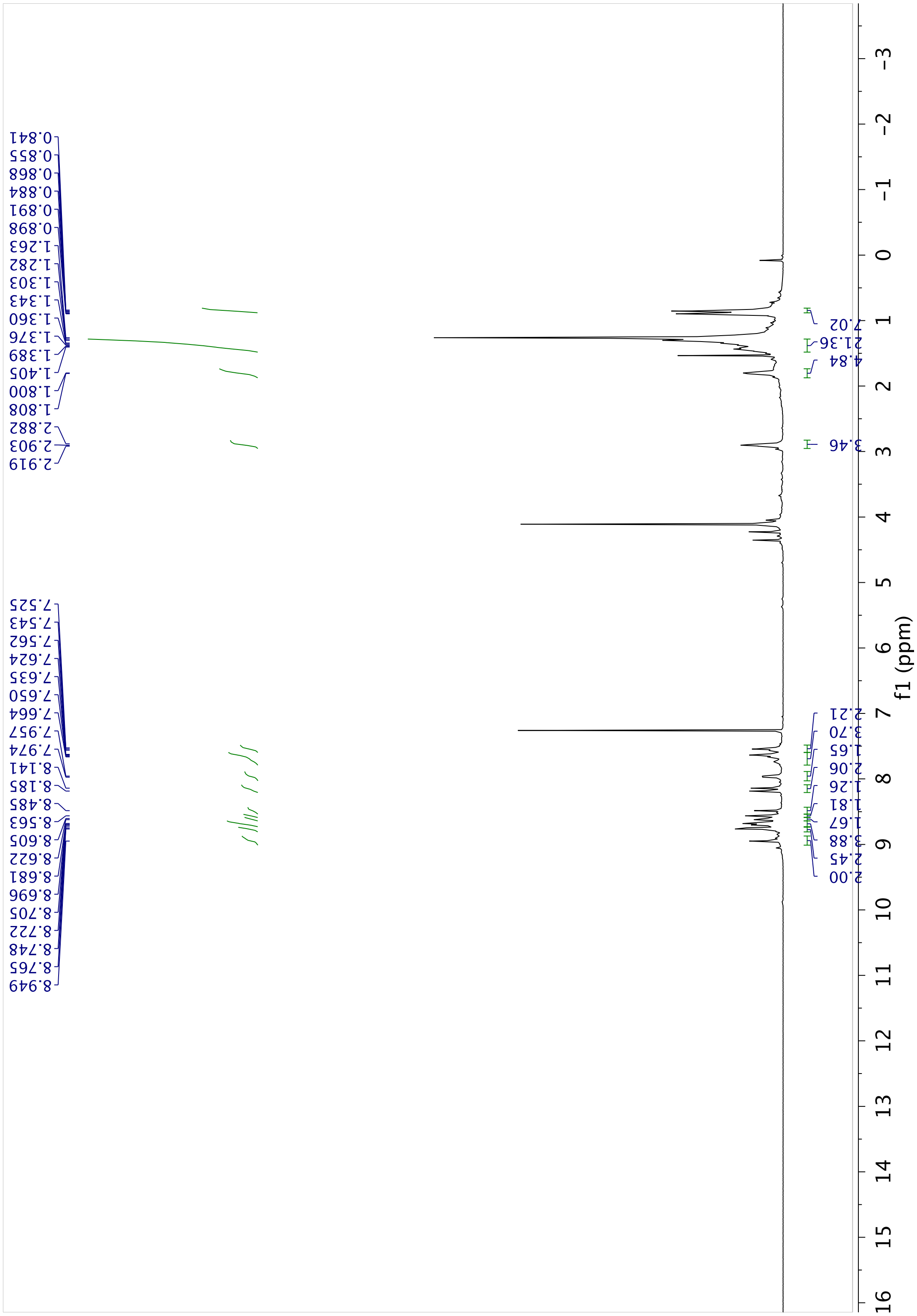
0.841
0.855
0.868
0.884
0.891
0.898
1.263
1.282
1.303
1.343
1.360
1.376
1.389
1.405
1.800
1.808
2.882
2.903
2.919
7.525
7.543
7.562
7.624
7.635
7.650
7.664
7.957
7.974
8.141
8.185
8.485
8.563
8.605
8.622
8.681
8.696
8.705
8.722
8.748
8.765
8.949
7.02
21.36
4.84
3.46
2.21
3.70
1.65
2.06
1.26
1.81
1.67
3.88
2.45
2.00
f1 (ppm)

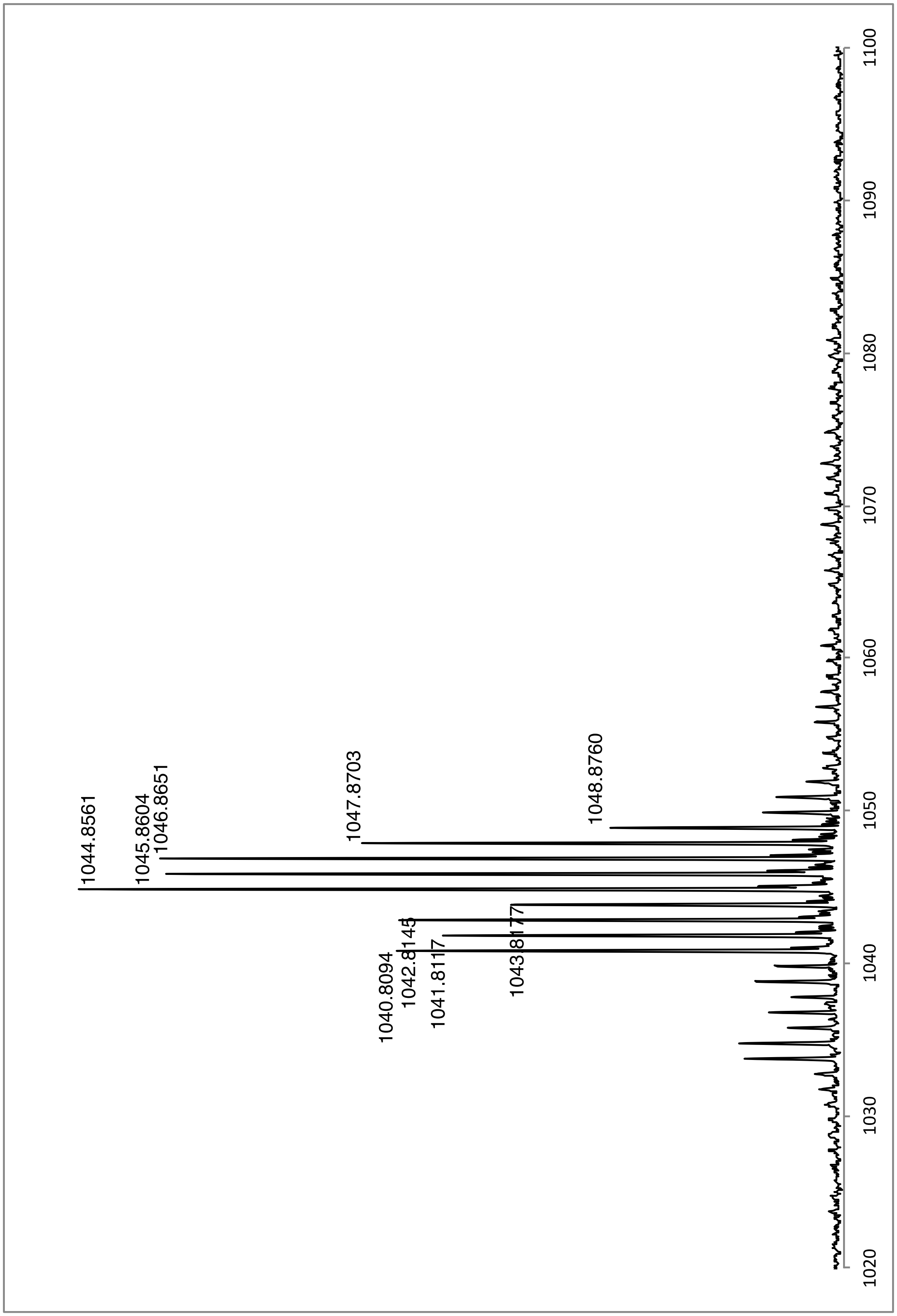
1044.8561
1045.8604
1046.8651
1047.8703
1048.8760
1040.8094
1042.8145
1041.8117
1043.8177
1020
1030
1040
1050
1060
1070
1080
1090
1100

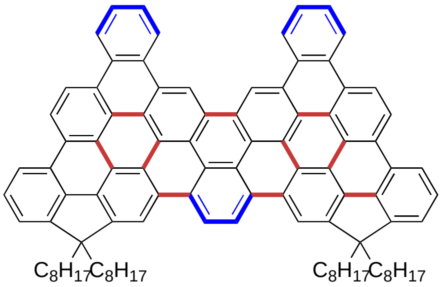

Nanographene **16e:**

TLC (SiO_2): R_f = 0.16 ($CHCl_3$).

^{1}H NMR: (500 MHz, $CDCl_3$): δ = 9.00-8.86 (m, 2H), 8.71-8.37 (m, 4H), 8.29-7.92 (m, 4H), 7.88-7.61 (m, 6H), 7.60-7.28 (m, 8H), 2.46-2.04 (m, 8H), 1.30-0.66 (m, 60H) ppm.

MS: (MALDI-TOF) Calculated for $C_{100}H_{92}$ [M^+] = 1292.7, Found 1292.9.

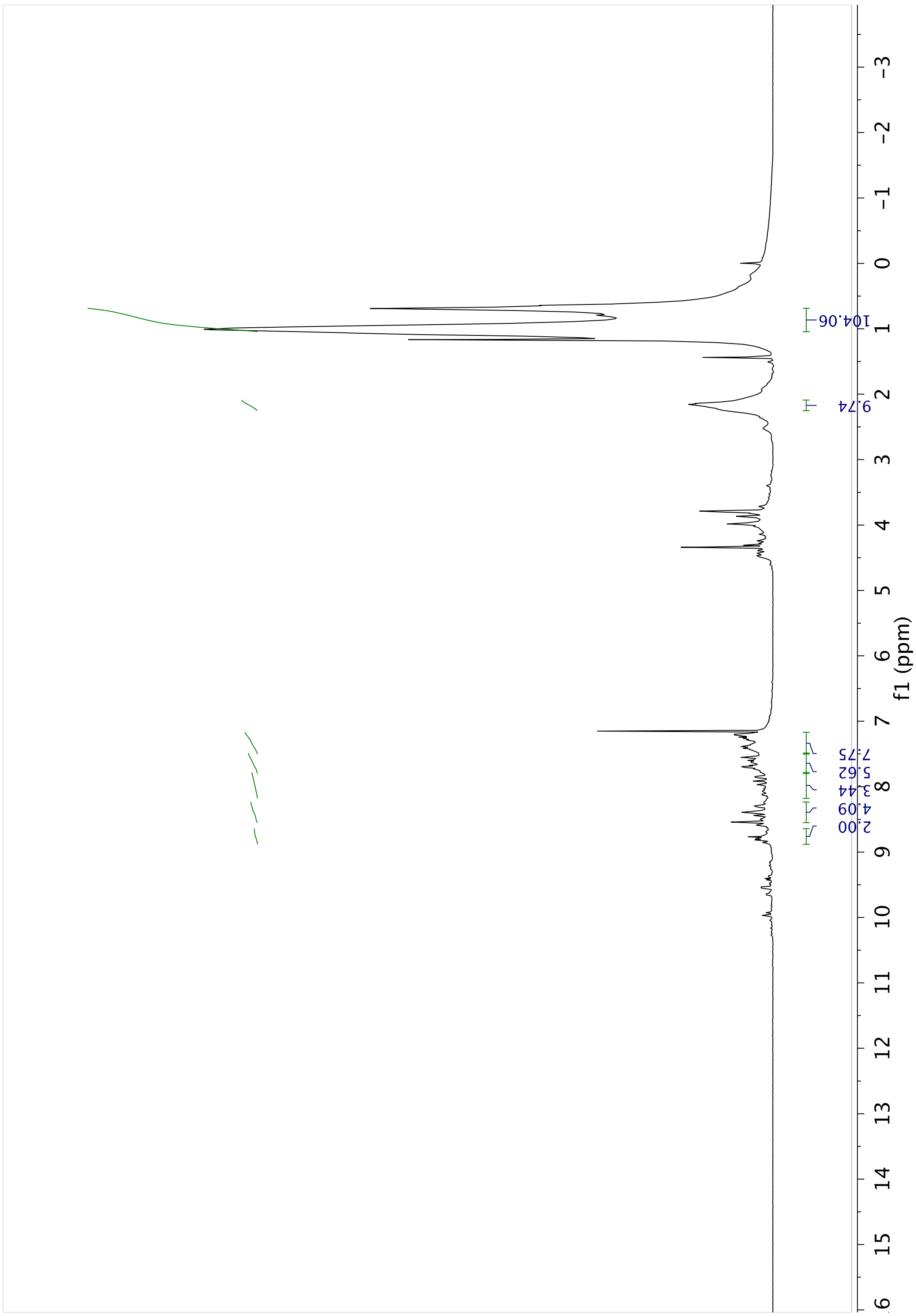
f1 (ppm)
104.06
9.74
7.75
5.62
3.44
4.09
2.00

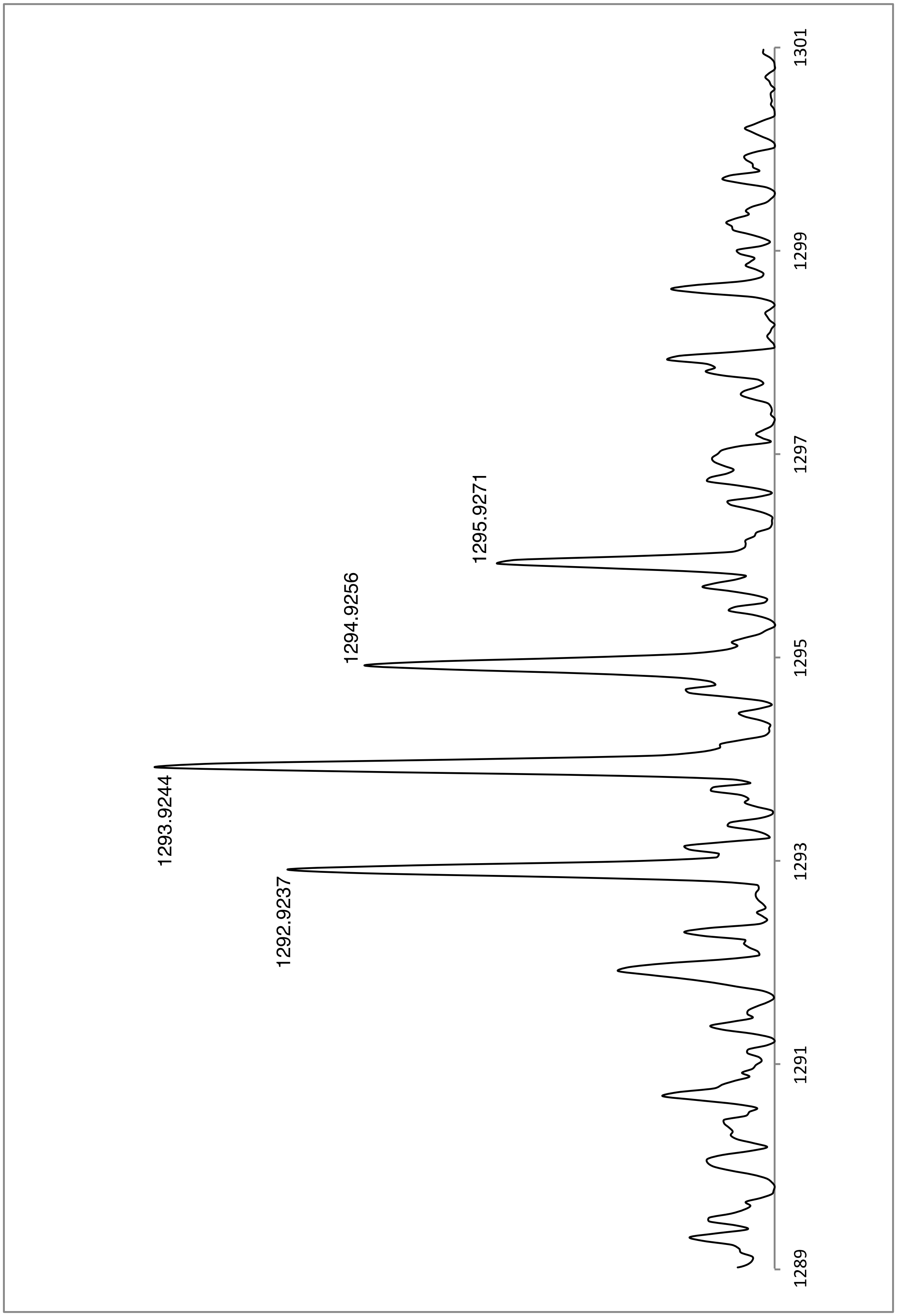
1292.9237
1293.9244
1294.9256
1295.9271
1289
1291
1293
1295
1297
1299
1301

Nanographene **16f:**

MS: (MALDI-TOF) Calculated for $C_{90}H_{74}N_2$ [M^+] = 1182.6, Found 1182.8.

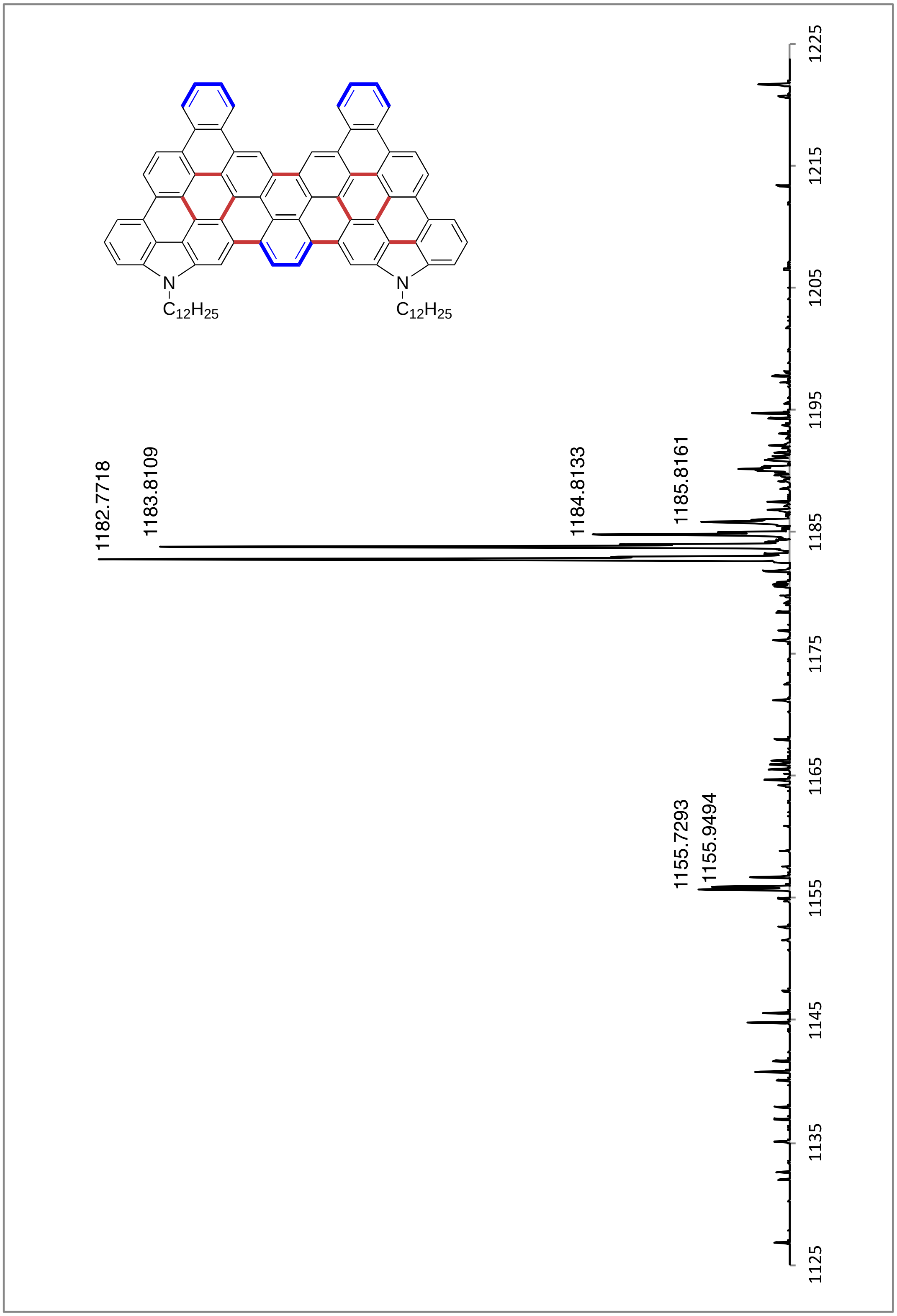

Nanographene **16g:**

MS: (MALDI-TOF) Calculated for $C_{64}H_{52}$ $[M^+]$ = 820.4, Found 820.3.

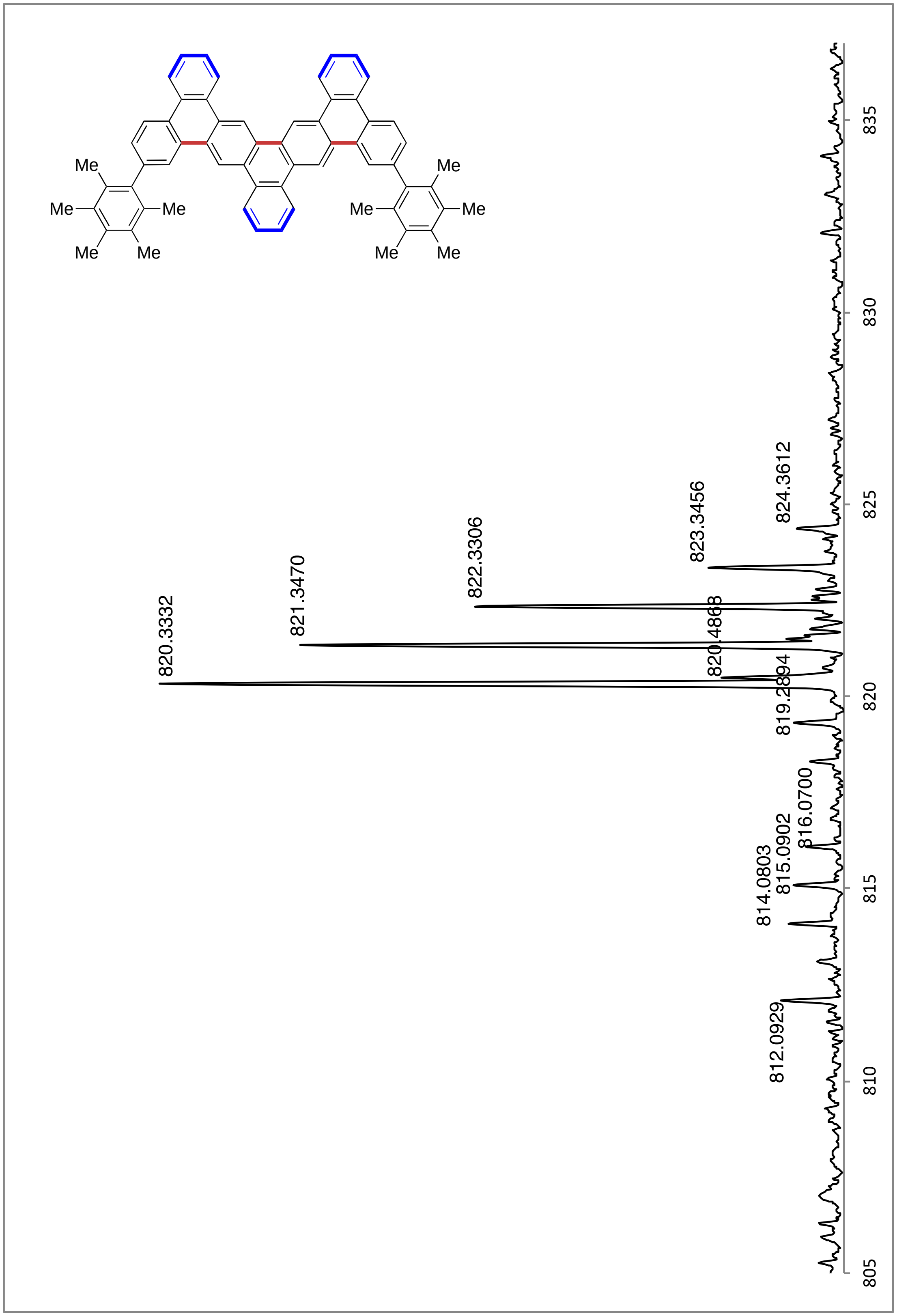

Spectroscopic Studies

Absorption and fluorescence spectra of **14**, **16a**, **16b**, **16d**, **16e**, **16h**, and **18** dissolved in dichloromethane reported herein were obtained from sufficiently dilute solutions in 1 cm pathlength cuvettes such that there were no observed changes in the absorption and emission spectra with further decreases in concentration indicating a lack of aggregation. Absorption spectra were determined from transmittance spectra measured with a Shimadzu UV-2600 UV-Vis spectrometer using an integrating sphere attachment. Corresponding emission spectra were measured with a PTI fluorimeter equipped with a 914 photomultiplier detector and a 75 W xenon lamp. The excitation slits were set to 5 nm and the emission slits were set to 1 nm. Excitation wavelengths used for emission reported for different compounds are as follows: **14** (370 nm), **16a** (425 nm), **16b** (300 nm), **16d** (300 nm), **16e** (300 nm), **16h** (300 nm), and **18** (450 nm).

The absorption and emission spectra of **16a** are present in Figure **1**. Molecule **16a** represents a novel low symmetry PAH available via the methods detailed in the main manuscript. The absorption spectra of PAH molecules are historically characterized by α-, p-, and β-bands.[xi] For **16a**, its α-band onset is at 2.09 eV, p-band at 2.92 eV, and β-band onset at 3.41 eV. Forgetting for a moment functional decoration about the PAH core, this molecule is likely to share electronic properties with those of a similar size and symmetry (D_{2h}). Most simply, it can be likened to ovalene fused with benzene rings at the K-regions or dibenzobisanthrene.[xii, xiii] The absorption and emission spectra of hexa-*peri*-hexabenzocoronene (HBC) **18** are presented in Figure **2**. HBC **18** has an α-band onset of 2.08 eV, a p-band onset of 2.79 eV, and a β-band onset of 3.30 eV. There are no other examples of HBCs functionalized solely in the bay region, so we turn to other examples of HBCs with D_{3h} symmetry for best comparison. For example, the fluorescence of HBC **18** is reminiscent of fluorescence from HBC's with three benzothiophene rings fused to the HBC periphery with a C_3 symmetry.[xiv] More generally, broadened fluorescence has been observed in other *meta*-substituted HBCs.[xv] Additionally, the redshifted absorption spectrum and anomalously large first vibronic transition in the emission spectrum for HBCs agrees with observations made for other alkyne substituted HBCs.[xvi]

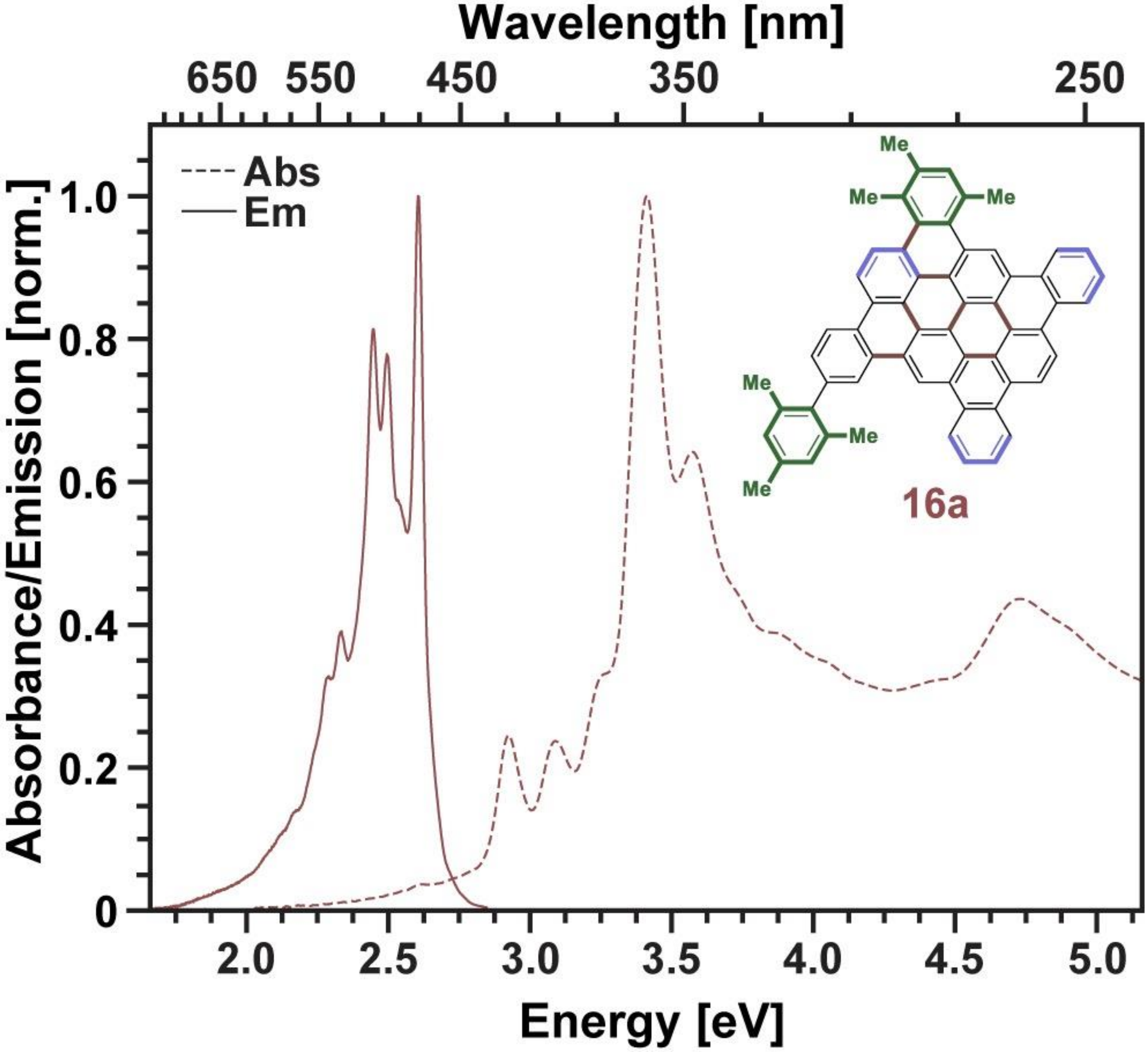

Figure 1. Absorption and emission spectra of **16a** in dichloromethane.

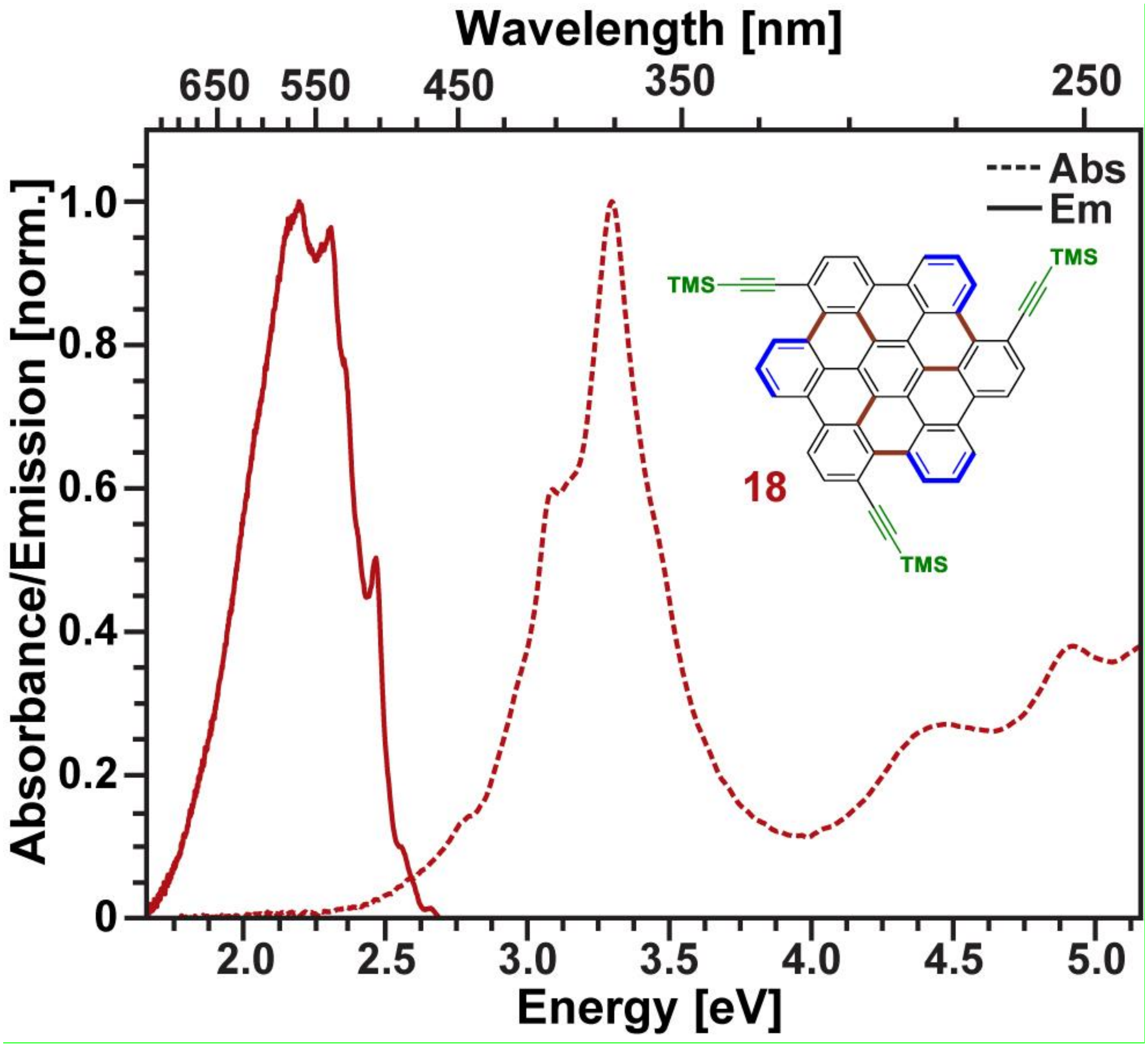

Figure 2. Absorption and emission spectra of **18** in dichloromethane.

Single Crystal Diffraction Data

X-ray Experimental for complex **11**:

X-ray Experimental for $C_{42}H_{28}Br_2$: Crystals grew as clusters of colorless prisms by slow evaporation from DCM and pentanes. The data crystal was cut from cluster of crystals and had approximate dimensions; 0.34 x 0.23 x 0.20 mm. The data were collected at room temperature on a Nonius Kappa CCD diffractometer using a Bruker AXS Apex II detector and a graphite monochromator with MoK□ radiation (□ = 0.71073Å). A total of 1323 frames of data were collected using □ and ϕ-scans with a scan range of 0.8° and a counting time of 44 seconds per frame. Details of crystal data, data collection and structure refinement are listed in Table 1. Data reduction were performed using SAINT V8.27B.[1] The structure was solved by direct methods using Superflip[2] and refined by full-matrix least-squares on F2 with anisotropic displacement parameters for the non-H atoms using SHELXL-2014/7.[3] Structure analysis was aided by use of the programs PLATON98[4] and WinGX.[5] The hydrogen atoms bound to carbon atoms were calculated in idealized positions with Uiso set to 1.2xUeq of the attached carbon atom.

The function, Σw(|Fo|2 - |Fc|2)2, was minimized, where w = 1/[(□(Fo))2 + (0.0785*P)2 + (2.1969*P)] and P = (|Fo|2 + 2|Fc|2)/3. Rw(F2) refined to 0.182, with R(F) equal to 0.0647 and a goodness of fit, S, = 1.04. Definitions used for calculating R(F),Rw(F2) and the goodness of fit, S, are given below.6 The data were checked for secondary extinction but no correction was necessary. Neutral atom scattering factors and values used to calculate the linear absorption coefficient are from the International Tables for X-ray Crystallography (1992).7 All figures were generated using SHELXTL/PC.8 Tables of positional and thermal parameters, bond lengths and angles, torsion angles and figures are found elsewhere.

Table 1. Crystal data and structure refinement for **11**.

Empirical formula	C42 H28 Br2	
Formula weight	692.46	
Temperature	293(2) K	
Wavelength	0.71073 Å	
Crystal system	triclinic	
Space group	P -1	
Unit cell dimensions	a = 9.928(2) Å	α= 91.365(8)°.
	b = 11.964(3) Å	β= 90.705(8)°.
	c = 13.330(4) Å	γ = 93.476(6)°.
Volume	1579.7(7) Å^3	
Z	2	
Density (calculated)	1.456 Mg/m^3	
Absorption coefficient	2.596 mm^{-1}	
F(000)	700	
Crystal size	0.34 x 0.23 x 0.20 mm^3	
Theta range for data collection	1.528 to 25.387°.	
Index ranges	-8<=h<=11, -14<=k<=14, -15<=l<=16	
Reflections collected	30260	
Independent reflections	5777 [R(int) = 0.0750]	
Completeness to theta = 25.242°	99.9 %	
Absorption correction	Semi-empirical from equivalents	
Max. and min. transmission	1.00 and 0.862	
Refinement method	Full-matrix least-squares on F^2	
Data / restraints / parameters	5777 / 0 / 397	
Goodness-of-fit on F^2	1.042	
Final R indices [I>2sigma(I)]	R1 = 0.0647, wR2 = 0.1562	
R indices (all data)	R1 = 0.1287, wR2 = 0.1819	
Extinction coefficient	n/a	
Largest diff. peak and hole	1.725 and -1.270 e.Å^{-3}	

Table 2. Atomic coordinates (x 10^4) and equivalent isotropic displacement parameters ($Å^2$x 10^3) for 1. U(eq) is defined as one third of the trace of the orthogonalized U^{ij} tensor.

	x	y	z	U(eq)
C1	-1314(6)	5691(5)	2952(5)	56(2)
C2	-2451(6)	6149(5)	2617(5)	57(2)
C3	-2614(5)	7278(5)	2823(4)	46(1)
C4	-1647(5)	7937(4)	3359(4)	39(1)
C5	-514(6)	7427(5)	3715(5)	52(2)
C6	-357(6)	6305(6)	3518(5)	63(2)
C7	-1847(5)	9134(4)	3574(4)	39(1)
C8	-3100(5)	9431(5)	3946(4)	45(1)
C9	-3323(6)	10532(5)	4204(4)	54(2)
C10	-2315(6)	11354(5)	4095(5)	57(2)
C11	-1094(6)	11088(5)	3713(5)	53(2)
C12	-843(5)	9987(4)	3444(4)	40(1)
C13	499(5)	9788(4)	3000(4)	34(1)
C14	629(5)	9287(4)	2060(4)	37(1)
C15	1881(5)	9187(4)	1637(4)	36(1)
C16	3061(5)	9581(4)	2132(4)	33(1)
C17	2921(5)	10061(4)	3091(4)	42(1)
C18	1681(5)	10172(4)	3507(4)	45(1)
C19	4417(5)	9551(4)	1681(4)	34(1)
C20	5250(6)	10532(5)	1738(4)	48(1)
C21	6514(6)	10605(5)	1321(5)	55(2)
C22	6978(6)	9676(6)	831(5)	55(2)
C23	6186(5)	8706(5)	767(4)	46(1)
C24	4909(5)	8611(4)	1204(4)	35(1)
C25	4163(5)	7489(4)	1129(4)	35(1)
C26	3517(5)	7005(4)	1952(4)	38(1)
C27	2876(5)	5941(4)	1881(4)	39(1)
C28	2858(5)	5324(4)	994(4)	39(1)
C29	3483(6)	5796(5)	173(4)	48(2)
C30	4116(6)	6855(5)	252(4)	48(2)
C31	2100(5)	4182(4)	871(4)	39(1)

C32	1248(6)	4009(5)	40(4)	53(2)
C33	469(6)	3019(5)	-113(5)	62(2)
C34	526(6)	2188(5)	575(5)	59(2)
C35	1381(6)	2334(5)	1401(4)	47(1)
C36	2169(5)	3334(4)	1568(4)	42(1)
C37	3029(5)	3419(4)	2495(4)	39(1)
C38	4413(6)	3651(5)	2471(4)	45(1)
C39	5195(6)	3652(5)	3331(4)	48(2)
C40	4589(6)	3455(5)	4239(4)	47(1)
C41	3226(6)	3242(5)	4291(5)	56(2)
C42	2466(6)	3213(5)	3431(5)	55(2)
Br1	-1063(1)	4160(1)	2606(1)	94(1)
Br2	5656(1)	3510(1)	5435(1)	71(1)

Table 3. Bond lengths [Å] and angles [°] for **11**.

C1-C2	1.359(9)	C19-C20	1.394(7)
C1-C6	1.372(9)	C19-C24	1.395(7)
C1-Br1	1.910(6)	C20-C21	1.378(8)
C2-C3	1.392(8)	C20-H20	0.93
C2-H2	0.93	C21-C22	1.381(9)
C3-C4	1.385(7)	C21-H21	0.93
C3-H3	0.93	C22-C23	1.362(8)
C4-C5	1.396(7)	C22-H22	0.93
C4-C7	1.479(7)	C23-C24	1.402(7)
C5-C6	1.381(8)	C23-H23	0.93
C5-H5	0.93	C24-C25	1.494(7)
C6-H6	0.93	C25-C30	1.377(7)
C7-C12	1.398(7)	C25-C26	1.397(7)
C7-C8	1.407(7)	C26-C27	1.389(7)
C8-C9	1.385(8)	C26-H26	0.93
C8-H8	0.93	C27-C28	1.377(7)
C9-C10	1.372(8)	C27-H27	0.93
C9-H9	0.93	C28-C29	1.380(7)
C10-C11	1.373(8)	C28-C31	1.524(7)
C10-H10	0.93	C29-C30	1.380(8)
C11-C12	1.395(7)	C29-H29	0.93
C11-H11	0.93	C30-H30	0.93
C12-C13	1.494(7)	C31-C32	1.389(7)
C13-C14	1.387(7)	C31-C36	1.395(7)
C13-C18	1.394(7)	C32-C33	1.385(8)
C14-C15	1.382(7)	C32-H32	0.93
C14-H14	0.93	C33-C34	1.371(9)
C15-C16	1.389(7)	C33-H33	0.93
C15-H15	0.93	C34-C35	1.383(8)
C16-C17	1.400(7)	C34-H34	0.93
C16-C19	1.484(7)	C35-C36	1.401(7)
C17-C18	1.369(7)	C35-H35	0.93
C17-H17	0.93	C36-C37	1.491(7)
C18-H18	0.93	C37-C38	1.386(7)

C37-C42	1.394(8)	C40-C41	1.364(8)
C38-C39	1.377(7)	C40-Br2	1.900(6)
C38-H38	0.93	C41-C42	1.364(8)
C39-C40	1.377(8)	C41-H41	0.93
C39-H39	0.93	C42-H42	0.93

C2-C1-C6	121.5(6)	C10-C11-C12	121.3(6)
C2-C1-Br1	118.3(5)	C10-C11-H11	119.4
C6-C1-Br1	120.2(5)	C12-C11-H11	119.4
C1-C2-C3	118.8(6)	C11-C12-C7	119.5(5)
C1-C2-H2	120.6	C11-C12-C13	117.3(5)
C3-C2-H2	120.6	C7-C12-C13	123.2(5)
C4-C3-C2	121.5(5)	C14-C13-C18	117.4(5)
C4-C3-H3	119.3	C14-C13-C12	122.5(4)
C2-C3-H3	119.3	C18-C13-C12	120.0(5)
C3-C4-C5	117.9(5)	C15-C14-C13	121.2(5)
C3-C4-C7	120.5(5)	C15-C14-H14	119.4
C5-C4-C7	121.6(5)	C13-C14-H14	119.4
C6-C5-C4	120.6(6)	C14-C15-C16	121.6(5)
C6-C5-H5	119.7	C14-C15-H15	119.2
C4-C5-H5	119.7	C16-C15-H15	119.2
C1-C6-C5	119.6(6)	C15-C16-C17	116.7(5)
C1-C6-H6	120.2	C15-C16-C19	123.3(5)
C5-C6-H6	120.2	C17-C16-C19	119.9(4)
C12-C7-C8	118.1(5)	C18-C17-C16	121.7(5)
C12-C7-C4	123.3(5)	C18-C17-H17	119.1
C8-C7-C4	118.6(5)	C16-C17-H17	119.1
C9-C8-C7	121.1(5)	C17-C18-C13	121.3(5)
C9-C8-H8	119.5	C17-C18-H18	119.4
C7-C8-H8	119.5	C13-C18-H18	119.4
C10-C9-C8	120.0(5)	C20-C19-C24	118.2(5)
C10-C9-H9	120.0	C20-C19-C16	117.4(5)
C8-C9-H9	120.0	C24-C19-C16	124.4(4)
C11-C10-C9	120.0(6)	C21-C20-C19	122.2(6)
C11-C10-H10	120.0	C21-C20-H20	118.9
C9-C10-H10	120.0	C19-C20-H20	118.9

C22-C21-C20	119.1(5)	C33-C32-C31	121.6(6)
C22-C21-H21	120.4	C33-C32-H32	119.2
C20-C21-H21	120.4	C31-C32-H32	119.2
C23-C22-C21	119.9(6)	C34-C33-C32	119.5(6)
C23-C22-H22	120.1	C34-C33-H33	120.3
C21-C22-H22	120.1	C32-C33-H33	120.3
C22-C23-C24	121.8(6)	C33-C34-C35	119.9(6)
C22-C23-H23	119.1	C33-C34-H34	120.0
C24-C23-H23	119.1	C35-C34-H34	120.0
C19-C24-C23	118.8(5)	C34-C35-C36	121.2(5)
C19-C24-C25	124.3(4)	C34-C35-H35	119.4
C23-C24-C25	116.9(5)	C36-C35-H35	119.4
C30-C25-C26	116.2(5)	C31-C36-C35	118.7(5)
C30-C25-C24	121.8(5)	C31-C36-C37	124.3(5)
C26-C25-C24	122.0(5)	C35-C36-C37	117.0(5)
C27-C26-C25	121.3(5)	C38-C37-C42	117.0(5)
C27-C26-H26	119.4	C38-C37-C36	122.5(5)
C25-C26-H26	119.4	C42-C37-C36	120.5(5)
C28-C27-C26	121.0(5)	C39-C38-C37	121.3(5)
C28-C27-H27	119.5	C39-C38-H38	119.4
C26-C27-H27	119.5	C37-C38-H38	119.4
C27-C28-C29	118.3(5)	C40-C39-C38	119.5(5)
C27-C28-C31	122.5(5)	C40-C39-H39	120.2
C29-C28-C31	119.1(5)	C38-C39-H39	120.2
C28-C29-C30	120.2(5)	C41-C40-C39	120.7(5)
C28-C29-H29	119.9	C41-C40-Br2	119.6(4)
C30-C29-H29	119.9	C39-C40-Br2	119.7(4)
C25-C30-C29	123.0(5)	C40-C41-C42	119.3(6)
C25-C30-H30	118.5	C40-C41-H41	120.4
C29-C30-H30	118.5	C42-C41-H41	120.4
C32-C31-C36	119.0(5)	C41-C42-C37	122.2(5)
C32-C31-C28	117.3(5)	C41-C42-H42	118.9
C36-C31-C28	123.6(5)	C37-C42-H42	118.9

Table 4. Anisotropic displacement parameters ($Å^2$x 10^3) for **11**. The anisotropic displacement factor exponent takes the form: $-2\pi^2[h^2 a^{*2}U^{11} + ... + 2 h k a^* b^* U^{12}]$

	U^{11}	U^{22}	U^{33}	U^{23}	U^{13}	U^{12}
C1	53(4)	40(3)	77(5)	6(3)	27(4)	6(3)
C2	53(4)	50(4)	68(4)	-2(3)	6(3)	-2(3)
C3	41(3)	52(4)	46(3)	9(3)	1(3)	7(3)
C4	38(3)	42(3)	37(3)	8(3)	5(2)	2(3)
C5	42(3)	48(4)	67(4)	15(3)	1(3)	-2(3)
C6	40(3)	61(5)	90(5)	24(4)	13(3)	8(3)
C7	40(3)	43(3)	35(3)	8(2)	3(2)	6(3)
C8	38(3)	51(4)	46(3)	6(3)	2(3)	0(3)
C9	41(3)	69(5)	52(4)	-5(3)	8(3)	17(3)
C10	66(4)	45(4)	62(4)	-2(3)	7(3)	11(3)
C11	49(4)	46(4)	64(4)	-4(3)	11(3)	2(3)
C12	38(3)	41(3)	40(3)	2(3)	1(2)	3(3)
C13	38(3)	27(3)	37(3)	2(2)	4(2)	2(2)
C14	36(3)	34(3)	41(3)	3(2)	-4(2)	0(2)
C15	42(3)	32(3)	33(3)	-2(2)	-1(2)	0(2)
C16	36(3)	24(3)	38(3)	5(2)	-1(2)	1(2)
C17	35(3)	45(3)	44(3)	-5(3)	-6(3)	-1(2)
C18	50(4)	47(4)	37(3)	-6(3)	-7(3)	0(3)
C19	35(3)	35(3)	33(3)	9(2)	-1(2)	-1(2)
C20	47(4)	45(4)	49(4)	5(3)	0(3)	0(3)
C21	49(4)	50(4)	65(4)	13(3)	-3(3)	-11(3)
C22	39(3)	67(5)	60(4)	26(3)	6(3)	0(3)
C23	44(3)	45(4)	50(4)	11(3)	6(3)	8(3)
C24	36(3)	38(3)	33(3)	11(2)	2(2)	3(2)
C25	38(3)	32(3)	36(3)	3(2)	3(2)	8(2)
C26	50(3)	34(3)	31(3)	2(2)	3(2)	6(3)
C27	51(3)	36(3)	30(3)	8(2)	11(2)	4(3)
C28	49(3)	35(3)	36(3)	5(3)	3(3)	8(2)
C29	74(4)	36(3)	35(3)	-3(3)	15(3)	4(3)
C30	66(4)	42(4)	36(3)	10(3)	20(3)	6(3)
C31	49(3)	33(3)	35(3)	-2(2)	7(3)	4(2)

C32	65(4)	47(4)	46(4)	-1(3)	-2(3)	9(3)
C33	68(4)	57(4)	59(4)	-13(4)	-19(3)	5(3)
C34	60(4)	43(4)	71(5)	-10(3)	-2(3)	-5(3)
C35	58(4)	33(3)	49(4)	4(3)	9(3)	1(3)
C36	46(3)	37(3)	44(3)	-5(3)	5(3)	5(3)
C37	47(3)	23(3)	46(3)	5(2)	3(3)	0(2)
C38	52(4)	48(3)	36(3)	1(3)	16(3)	4(3)
C39	45(3)	52(4)	48(4)	2(3)	1(3)	4(3)
C40	63(4)	37(3)	42(4)	3(3)	0(3)	12(3)
C41	58(4)	67(4)	43(4)	16(3)	9(3)	-2(3)
C42	47(4)	63(4)	54(4)	12(3)	8(3)	-5(3)
Br1	81(1)	52(1)	151(1)	2(1)	32(1)	15(1)
Br2	84(1)	76(1)	54(1)	-4(1)	-15(1)	18(1)

Table 5. Hydrogen coordinates (x 10^4) and isotropic displacement parameters ($Å^2$x 10^3) for **11**.

	x	y	z	U(eq)
H2	-3108	5715	2256	69
H3	-3389	7597	2596	55
H5	142	7847	4089	63
H6	392	5967	3768	76
H8	-3790	8879	4019	54
H9	-4156	10714	4452	64
H10	-2460	12092	4279	69
H11	-420	11653	3633	63
H14	-141	9014	1708	45
H15	1935	8847	1005	43
H17	3691	10310	3455	50
H18	1625	10510	4139	54
H20	4942	11158	2069	57
H21	7047	11271	1367	66
H22	7829	9713	547	66
H23	6501	8091	423	55
H26	3517	7403	2560	46
H27	2452	5640	2440	47
H29	3478	5399	-435	58
H30	4529	7153	-312	57
H32	1200	4573	-425	63
H33	-88	2918	-678	74
H34	-10	1528	486	70
H35	1434	1758	1853	56
H38	4822	3809	1862	54
H39	6126	3785	3300	58
H41	2820	3118	4907	67
H42	1541	3049	3469	66

Table 6. Torsion angles [°] for **11**.

C6-C1-C2-C3	2.6(9)	C15-C16-C17-C18	-2.3(8)
Br1-C1-C2-C3	-177.0(4)	C19-C16-C17-C18	175.7(5)
C1-C2-C3-C4	0.0(9)	C16-C17-C18-C13	1.5(8)
C2-C3-C4-C5	-1.8(8)	C14-C13-C18-C17	0.1(8)
C2-C3-C4-C7	-179.7(5)	C12-C13-C18-C17	-176.0(5)
C3-C4-C5-C6	1.3(8)	C15-C16-C19-C20	131.3(5)
C7-C4-C5-C6	179.0(5)	C17-C16-C19-C20	-46.4(7)
C2-C1-C6-C5	-3.2(10)	C15-C16-C19-C24	-48.5(7)
Br1-C1-C6-C5	176.4(5)	C17-C16-C19-C24	133.8(5)
C4-C5-C6-C1	1.2(9)	C24-C19-C20-C21	1.6(8)
C3-C4-C7-C12	-134.1(5)	C16-C19-C20-C21	-178.2(5)
C5-C4-C7-C12	48.2(8)	C19-C20-C21-C22	-0.2(9)
C3-C4-C7-C8	47.1(7)	C20-C21-C22-C23	0.1(9)
C5-C4-C7-C8	-130.6(6)	C21-C22-C23-C24	-1.4(9)
C12-C7-C8-C9	-1.9(8)	C20-C19-C24-C23	-2.8(7)
C4-C7-C8-C9	177.0(5)	C16-C19-C24-C23	177.0(5)
C7-C8-C9-C10	0.3(8)	C20-C19-C24-C25	176.9(5)
C8-C9-C10-C11	1.1(9)	C16-C19-C24-C25	-3.3(8)
C9-C10-C11-C12	-0.9(9)	C22-C23-C24-C19	2.8(8)
C10-C11-C12-C7	-0.8(8)	C22-C23-C24-C25	-177.0(5)
C10-C11-C12-C13	177.8(5)	C19-C24-C25-C30	137.0(5)
C8-C7-C12-C11	2.1(7)	C23-C24-C25-C30	-43.3(7)
C4-C7-C12-C11	-176.7(5)	C19-C24-C25-C26	-45.5(7)
C8-C7-C12-C13	-176.3(5)	C23-C24-C25-C26	134.2(5)
C4-C7-C12-C13	4.8(8)	C30-C25-C26-C27	0.4(7)
C11-C12-C13-C14	-122.3(6)	C24-C25-C26-C27	-177.3(5)
C7-C12-C13-C14	56.2(7)	C25-C26-C27-C28	0.2(8)
C11-C12-C13-C18	53.7(7)	C26-C27-C28-C29	-0.7(8)
C7-C12-C13-C18	-127.9(6)	C26-C27-C28-C31	-176.6(5)
C18-C13-C14-C15	-0.8(7)	C27-C28-C29-C30	0.6(8)
C12-C13-C14-C15	175.2(5)	C31-C28-C29-C30	176.6(5)
C13-C14-C15-C16	0.0(8)	C26-C25-C30-C29	-0.5(8)
C14-C15-C16-C17	1.5(7)	C24-C25-C30-C29	177.2(5)
C14-C15-C16-C19	-176.3(4)	C28-C29-C30-C25	0.0(9)

C27-C28-C31-C32	130.4(6)
C29-C28-C31-C32	-45.5(7)
C27-C28-C31-C36	-46.5(8)
C29-C28-C31-C36	137.7(6)
C36-C31-C32-C33	0.0(9)
C28-C31-C32-C33	-177.0(5)
C31-C32-C33-C34	0.6(10)
C32-C33-C34-C35	-1.5(10)
C33-C34-C35-C36	1.9(9)
C32-C31-C36-C35	0.3(8)
C28-C31-C36-C35	177.1(5)
C32-C31-C36-C37	-179.3(5)
C28-C31-C36-C37	-2.5(8)
C34-C35-C36-C31	-1.3(8)
C34-C35-C36-C37	178.3(5)
C31-C36-C37-C38	-56.7(8)
C35-C36-C37-C38	123.7(6)
C31-C36-C37-C42	126.4(6)
C35-C36-C37-C42	-53.2(7)
C42-C37-C38-C39	1.4(8)
C36-C37-C38-C39	-175.7(5)
C37-C38-C39-C40	-2.1(8)
C38-C39-C40-C41	1.0(9)
C38-C39-C40-Br2	-177.7(4)
C39-C40-C41-C42	0.8(9)
Br2-C40-C41-C42	179.5(5)
C40-C41-C42-C37	-1.5(9)
C38-C37-C42-C41	0.5(9)
C36-C37-C42-C41	177.6(5)

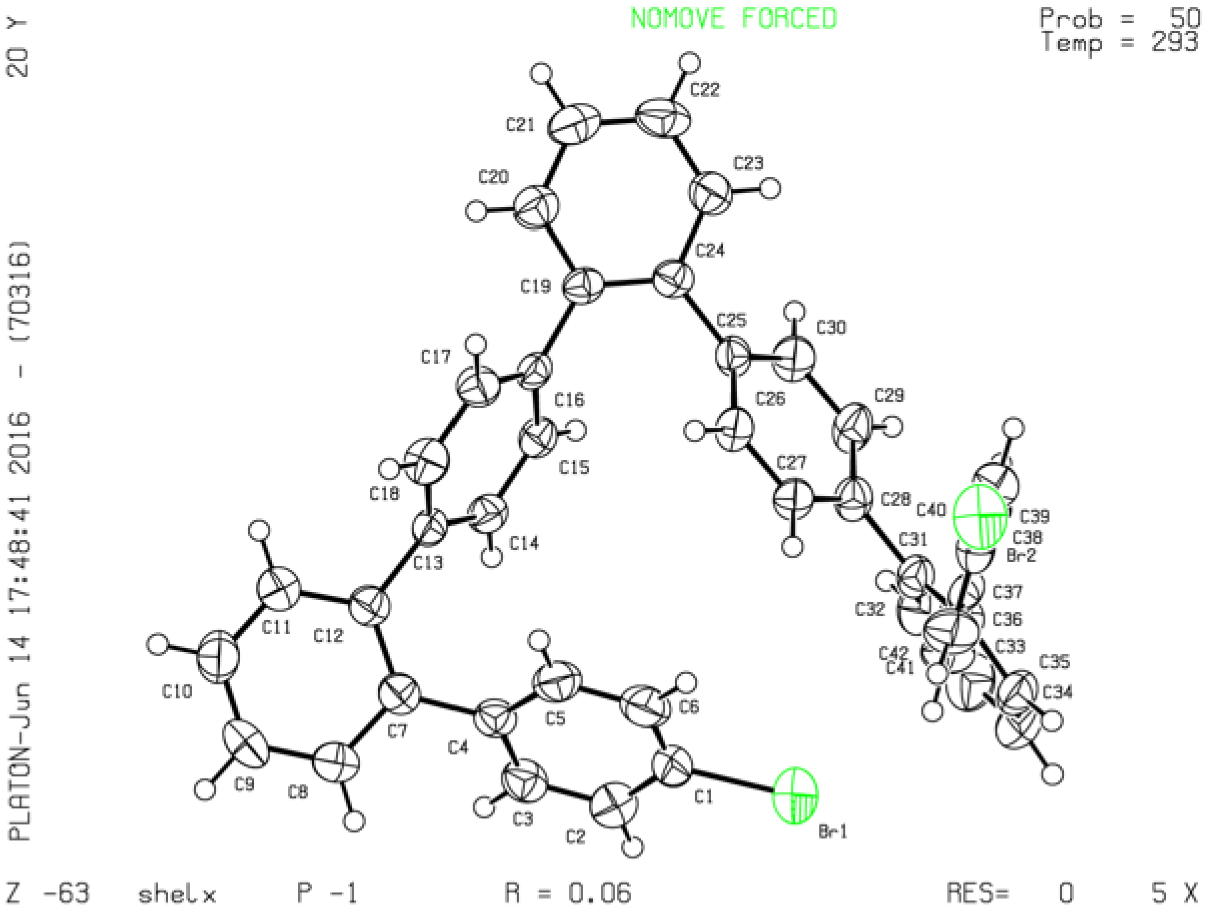
NOMOVE FORCED
Prob = 50
Temp = 293
20 Y
PLATON-Jun 14 17:48:41 2016 - (70316)
Z -63 shelx P -1 R = 0.06 RES= 0 5 X
C1
C2
C3
C4
C5
C6
C7
C8
C9
C10
C11
C12
C13
C14
C15
C16
C17
C18
C19
C20
C21
C22
C23
C24
C25
C26
C27
C28
C29
C30
C31
C32
C33
C34
C35
C36
C37
C38
C39
C40
C41
C42
Br1
Br2

X-ray Experimental for complex **14**:

X-ray Experimental for 3 $C_{34}H_{18}S_2$ – CH_2Cl_2: Crystals grew as long colorless needles by slow evaporation from dichloromethane and pentanes. The data crystal was cut from a larger crystal and had approximate dimensions; 0.52 x 0.14 x 0.11 mm. The data were collected at -173 °C on a Nonius Kappa CCD diffractometer using a Bruker AXS Apex II detector and a graphite monochromator with MoK□ radiation (□ = 0.71073Å). Reduced temperatures were maintained by use of an Oxford Cryosystems 600 low-temperature device. A total of 1155 frames of data were collected using ω-scans with a scan range of 0.5° and a counting time of 66 seconds per frame. Details of crystal data, data collection and structure refinement are listed in Table 7. Data reduction were performed using SAINT V8.27B.[1] The structure was solved by direct methods using SIR97[2] and refined by full-matrix least-squares on F2 with anisotropic displacement parameters for the non-H atoms using SHELXL-2014/7.[3] Structure analysis was aided by use of the programs PLATON98[4] and WinGX.[5]

A molecule of dichloromethane badly disordered around a crystallographic inversion center. Attempts to model the disorder were unsatisfactory. The contributions to the scattering factors due to the solvent molecule were removed by use of the utility SQUEEZE[6] in PLATON98.

The function, Σw(|Fo|2 - |Fc|2)2, was minimized, where w = 1/[(□(Fo))2 + (0.0712*P)2] and P = (|Fo|2 + 2|Fc|2)/3. Rw(F2) refined to 0.183, with R(F) equal to 0.0724 and a goodness of fit, S, = 0.963. Definitions used for calculating R(F),Rw(F2) and the goodness of fit, S, are given below.7 The data were checked for secondary extinction but no correction was necessary. Neutral atom scattering factors and values used to calculate the linear absorption coefficient are from the International Tables for X-ray Crystallography (1992).8 All figures were generated using SHELXTL/PC.9 Tables of positional and thermal parameters, bond lengths and angles, torsion angles and figures are found elsewhere.

Table 7. Crystal data and structure refinement for **14**.

Empirical formula	C103 H56 Cl2 S6	
Formula weight	1556.73	
Temperature	100(2) K	
Wavelength	0.71073 Å	
Crystal system	monoclinic	
Space group	P 21/n	
Unit cell dimensions	a = 9.2129(16) Å	α= 90°.
	b = 21.272(4) Å	β= 91.730(11)°.
	c = 36.423(6) Å	γ = 90°.
Volume	7135(2) $Å^3$	
Z	4	
Density (calculated)	1.449 Mg/m^3	
Absorption coefficient	0.323 mm^{-1}	
F(000)	3216	
Crystal size	0.52 x 0.14 x 0.12 mm^3	
Theta range for data collection	1.915 to 22.873°.	
Index ranges	-10<=h<=9, -23<=k<=23, -39<=l<=39	
Reflections collected	44139	
Independent reflections	9603 [R(int) = 0.1843]	
Completeness to theta = 22.873°	98.3 %	
Absorption correction	Semi-empirical from equivalents	
Max. and min. transmission	1.00 and 0.749	
Refinement method	Full-matrix least-squares on F^2	
Data / restraints / parameters	9603 / 0 / 973	
Goodness-of-fit on F^2	0.963	
Final R indices [I>2sigma(I)]	R1 = 0.0724, wR2 = 0.1467	
R indices (all data)	R1 = 0.1662, wR2 = 0.1829	
Extinction coefficient	n/a	
Largest diff. peak and hole	0.283 and -0.345 e.$Å^{-3}$	

Table 8. Atomic coordinates (x 10^4) and equivalent isotropic displacement parameters ($Å^2$x 10^3) for **14**. U(eq) is defined as one third of the trace of the orthogonalized U^{ij} tensor.

	x	y	z	U(eq)
C1	4616(7)	1218(3)	5532(2)	28(2)
C2	5003(7)	667(3)	5741(2)	37(2)
C3	5893(7)	701(3)	6061(2)	37(2)
C4	6415(7)	163(3)	6225(2)	41(2)
C5	6035(8)	-427(3)	6073(2)	49(2)
C6	5136(7)	-467(3)	5765(2)	39(2)
C7	4576(7)	82(3)	5592(2)	31(2)
C8	3567(7)	61(3)	5271(2)	32(2)
C9	3026(7)	617(3)	5114(2)	28(2)
C10	3755(7)	1201(3)	5216(2)	31(2)
C11	3840(7)	1797(3)	5010(2)	28(2)
C12	3352(6)	1951(3)	4656(2)	29(2)
C13	3586(7)	2549(3)	4520(2)	42(2)
C14	4323(8)	2992(3)	4736(2)	45(2)
C15	4860(7)	2847(3)	5076(2)	38(2)
C16	4653(7)	2249(3)	5214(2)	35(2)
C17	3175(7)	-517(3)	5112(2)	34(2)
C18	2266(7)	-547(3)	4813(2)	35(2)
C19	1522(7)	1(3)	4680(2)	29(2)
C20	1790(7)	578(3)	4860(2)	27(2)
C21	508(7)	-41(3)	4370(2)	34(2)
C22	256(7)	-594(3)	4166(2)	38(2)
C23	-750(7)	-616(3)	3881(2)	40(2)
C24	-1548(8)	-88(3)	3781(2)	43(2)
C25	-1317(7)	470(3)	3962(2)	36(2)
C26	-317(7)	501(3)	4259(2)	33(2)
C27	-126(7)	1051(3)	4483(2)	32(2)
C28	778(7)	1087(3)	4788(2)	30(2)
C29	412(7)	1640(3)	5010(2)	30(2)
C30	897(7)	1805(3)	5366(2)	35(2)
C31	338(8)	2338(3)	5527(2)	44(2)

C32	-654(8)	2713(3)	5338(2)	46(2)
C33	-1171(8)	2554(3)	4994(2)	47(2)
C34	-640(8)	2011(3)	4836(2)	40(2)
C35	6333(7)	3267(3)	6407(2)	34(2)
C36	6669(7)	2663(3)	6573(2)	35(2)
C37	7647(7)	2239(3)	6426(2)	36(2)
C38	8042(7)	1711(3)	6613(2)	42(2)
C39	7470(7)	1589(3)	6955(2)	41(2)
C40	6502(7)	1999(3)	7110(2)	39(2)
C41	6087(7)	2562(3)	6921(2)	32(2)
C42	5066(7)	2994(3)	7068(2)	31(2)
C43	4598(7)	3532(3)	6862(2)	33(2)
C44	5440(8)	3704(3)	6553(2)	34(2)
C45	5585(7)	4318(3)	6375(2)	36(2)
C46	5062(7)	4912(3)	6469(2)	39(2)
C47	5382(9)	5427(4)	6261(2)	53(2)
C48	6196(9)	5376(4)	5955(2)	57(2)
C49	6788(9)	4803(4)	5858(2)	55(2)
C50	6494(8)	4278(3)	6073(2)	44(2)
C51	4502(7)	2918(3)	7428(2)	35(2)
C52	3603(7)	3349(3)	7570(2)	37(2)
C53	2988(7)	3839(3)	7353(2)	35(2)
C54	3380(7)	3872(3)	6981(2)	33(2)
C55	1968(8)	4291(3)	7498(2)	41(2)
C56	1655(8)	4298(4)	7874(2)	53(2)
C57	707(9)	4736(4)	8010(2)	61(2)
C58	75(9)	5193(4)	7774(3)	65(3)
C59	374(8)	5194(3)	7417(3)	59(2)
C60	1311(8)	4742(3)	7266(3)	50(2)
C61	1524(8)	4682(3)	6881(2)	48(2)
C62	2429(7)	4235(3)	6733(2)	40(2)
C63	2164(8)	4165(3)	6335(2)	43(2)
C64	2673(8)	3727(3)	6085(2)	48(2)
C65	2219(9)	3739(4)	5726(2)	62(2)
C66	1238(11)	4190(5)	5597(3)	75(3)
C67	670(9)	4608(5)	5836(3)	77(3)

C68	1130(8)	4605(3)	6207(2)	50(2)
C69	6402(7)	2993(3)	2594(2)	29(2)
C70	6657(7)	3030(3)	2212(2)	32(2)
C71	7603(7)	3488(3)	2063(2)	32(2)
C72	8017(7)	3443(3)	1706(2)	37(2)
C73	7495(7)	2957(3)	1485(2)	36(2)
C74	6539(7)	2525(3)	1622(2)	35(2)
C75	6092(7)	2539(3)	1987(2)	28(2)
C76	5086(7)	2097(3)	2138(2)	29(2)
C77	4678(7)	2133(3)	2505(2)	28(2)
C78	5533(7)	2534(3)	2745(2)	29(2)
C79	5781(6)	2513(3)	3146(2)	29(2)
C80	5374(7)	2065(3)	3408(2)	34(2)
C81	5762(7)	2154(3)	3770(2)	34(2)
C82	6536(7)	2680(3)	3886(2)	36(2)
C83	7034(7)	3100(3)	3637(2)	36(2)
C84	6657(7)	3011(3)	3273(2)	30(2)
C85	4494(7)	1612(3)	1916(2)	31(2)
C86	3590(7)	1172(3)	2049(2)	31(2)
C87	3042(7)	1222(3)	2405(2)	26(2)
C88	3490(7)	1751(3)	2620(2)	30(2)
C89	2071(7)	748(3)	2548(2)	28(2)
C90	1649(7)	210(3)	2346(2)	33(2)
C91	769(7)	-235(3)	2490(2)	34(2)
C92	271(7)	-175(3)	2844(2)	37(2)
C93	656(7)	353(3)	3053(2)	37(2)
C94	1547(7)	817(3)	2899(2)	31(2)
C95	1791(7)	1412(3)	3087(2)	31(2)
C96	2627(7)	1881(3)	2939(2)	28(2)
C97	2364(7)	2481(3)	3129(2)	29(2)
C98	2753(7)	3096(3)	3037(2)	36(2)
C99	2309(7)	3583(3)	3255(2)	35(2)
C100	1504(7)	3485(3)	3567(2)	40(2)
C101	1055(7)	2880(3)	3653(2)	39(2)
C102	1473(7)	2396(3)	3430(2)	31(2)
S1	5373(2)	1952(1)	5623(1)	40(1)

S2	-1250(2)	1700(1)	4421(1)	39(1)
S3	7253(2)	3540(1)	6026(1)	49(1)
S4	473(2)	5072(1)	6552(1)	65(1)
S5	7304(2)	3470(1)	2915(1)	37(1)
S6	870(2)	1621(1)	3475(1)	36(1)

Table 9. Bond lengths [Å] and angles [°] for **14**.

C1-C10	1.376(8)	C19-C20	1.410(8)
C1-C2	1.436(8)	C19-C21	1.444(8)
C1-S1	1.738(6)	C20-C28	1.448(8)
C2-C3	1.408(8)	C21-C22	1.407(8)
C2-C7	1.409(9)	C21-C26	1.432(9)
C3-C4	1.370(8)	C22-C23	1.373(8)
C3-H3	0.95	C22-H22	0.95
C4-C5	1.412(9)	C23-C24	1.384(9)
C4-H4	0.95	C23-H23	0.95
C5-C6	1.377(8)	C24-C25	1.373(8)
C5-H5	0.95	C24-H24	0.95
C6-C7	1.416(8)	C25-C26	1.401(8)
C6-H6	0.95	C25-H25	0.95
C7-C8	1.471(8)	C26-C27	1.433(8)
C8-C9	1.400(8)	C27-C28	1.372(8)
C8-C17	1.401(8)	C27-S2	1.737(6)
C9-C20	1.450(8)	C28-C29	1.469(8)
C9-C10	1.455(8)	C29-C34	1.388(8)
C10-C11	1.476(8)	C29-C30	1.405(8)
C11-C12	1.390(8)	C30-C31	1.383(9)
C11-C16	1.417(8)	C30-H30	0.95
C12-C13	1.386(8)	C31-C32	1.382(9)
C12-H12	0.95	C31-H31	0.95
C13-C14	1.391(9)	C32-C33	1.369(9)
C13-H13	0.95	C32-H32	0.95
C14-C15	1.356(8)	C33-C34	1.386(9)
C14-H14	0.95	C33-H33	0.95
C15-C16	1.384(8)	C34-S2	1.729(7)
C15-H15	0.95	C35-C44	1.362(9)
C16-S1	1.732(6)	C35-C36	1.449(9)
C17-C18	1.356(8)	C35-S3	1.747(7)
C17-H17	0.95	C36-C37	1.393(9)
C18-C19	1.429(8)	C36-C41	1.408(9)
C18-H18	0.95	C37-C38	1.357(9)

C37-H37	0.95	C57-H57	0.95
C38-C39	1.390(9)	C58-C59	1.337(10)
C38-H38	0.95	C58-H58	0.95
C39-C40	1.381(9)	C59-C60	1.415(10)
C39-H39	0.95	C59-H59	0.95
C40-C41	1.429(8)	C60-C61	1.428(10)
C40-H40	0.95	C61-C62	1.384(10)
C41-C42	1.431(9)	C61-S4	1.729(7)
C42-C43	1.429(8)	C62-C63	1.470(9)
C42-C51	1.432(8)	C63-C64	1.394(9)
C43-C54	1.412(9)	C63-C68	1.406(9)
C43-C44	1.435(9)	C64-C65	1.359(9)
C44-C45	1.465(9)	C64-H64	0.95
C45-C46	1.399(9)	C65-C66	1.390(11)
C45-C50	1.407(9)	C65-H65	0.95
C46-C47	1.369(9)	C66-C67	1.359(11)
C46-H46	0.95	C66-H66	0.95
C47-C48	1.368(10)	C67-C68	1.404(10)
C47-H47	0.95	C67-H67	0.95
C48-C49	1.386(10)	C68-S4	1.725(8)
C48-H48	0.95	C69-C78	1.386(8)
C49-C50	1.392(9)	C69-C70	1.419(8)
C49-H49	0.95	C69-S5	1.742(6)
C50-S3	1.730(7)	C70-C75	1.417(8)
C51-C52	1.348(8)	C70-C71	1.426(8)
C51-H51	0.95	C71-C72	1.367(8)
C52-C53	1.415(9)	C71-H71	0.95
C52-H52	0.95	C72-C73	1.389(8)
C53-C54	1.416(9)	C72-H72	0.95
C53-C55	1.455(9)	C73-C74	1.376(8)
C54-C62	1.459(9)	C73-H73	0.95
C55-C60	1.404(9)	C74-C75	1.404(8)
C55-C56	1.407(9)	C74-H74	0.95
C56-C57	1.379(10)	C75-C76	1.442(8)
C56-H56	0.95	C76-C77	1.403(8)
C57-C58	1.411(11)	C76-C85	1.409(8)

C77-C88	1.437(8)	C90-C91	1.362(8)
C77-C78	1.439(8)	C90-H90	0.95
C78-C79	1.474(8)	C91-C92	1.386(8)
C79-C84	1.400(8)	C91-H91	0.95
C79-C80	1.407(8)	C92-C93	1.398(8)
C80-C81	1.369(8)	C92-H92	0.95
C80-H80	0.95	C93-C94	1.410(9)
C81-C82	1.385(8)	C93-H93	0.95
C81-H81	0.95	C94-C95	1.452(8)
C82-C83	1.364(8)	C95-C96	1.380(8)
C82-H82	0.95	C95-S6	1.728(7)
C83-C84	1.375(8)	C96-C97	1.475(8)
C83-H83	0.95	C97-C102	1.400(8)
C84-S5	1.746(6)	C97-C98	1.401(8)
C85-C86	1.353(8)	C98-C99	1.375(8)
C85-H85	0.95	C98-H98	0.95
C86-C87	1.409(8)	C99-C100	1.392(9)
C86-H86	0.95	C99-H99	0.95
C87-C88	1.425(8)	C100-C101	1.390(9)
C87-C89	1.455(8)	C100-H100	0.95
C88-C96	1.452(9)	C101-C102	1.374(8)
C89-C94	1.387(8)	C101-H101	0.95
C89-C90	1.410(8)	C102-S6	1.749(6)

C10-C1-C2	123.3(6)	C6-C5-C4	120.7(6)
C10-C1-S1	113.7(5)	C6-C5-H5	119.6
C2-C1-S1	122.6(5)	C4-C5-H5	119.6
C3-C2-C7	120.8(6)	C5-C6-C7	120.9(7)
C3-C2-C1	121.8(6)	C5-C6-H6	119.5
C7-C2-C1	117.1(6)	C7-C6-H6	119.5
C4-C3-C2	120.5(7)	C2-C7-C6	117.6(6)
C4-C3-H3	119.7	C2-C7-C8	119.7(6)
C2-C3-H3	119.7	C6-C7-C8	122.7(6)
C3-C4-C5	119.3(6)	C9-C8-C17	119.3(6)
C3-C4-H4	120.3	C9-C8-C7	120.4(6)
C5-C4-H4	120.3	C17-C8-C7	120.2(6)

C8-C9-C20	118.4(6)	C22-C21-C26	116.5(6)
C8-C9-C10	117.5(6)	C22-C21-C19	123.8(6)
C20-C9-C10	124.1(6)	C26-C21-C19	119.7(6)
C1-C10-C9	119.0(6)	C23-C22-C21	121.7(7)
C1-C10-C11	111.4(5)	C23-C22-H22	119.1
C9-C10-C11	129.3(5)	C21-C22-H22	119.1
C12-C11-C16	118.5(6)	C22-C23-C24	120.8(7)
C12-C11-C10	130.8(6)	C22-C23-H23	119.6
C16-C11-C10	110.5(5)	C24-C23-H23	119.6
C13-C12-C11	119.9(6)	C25-C24-C23	120.2(6)
C13-C12-H12	120.0	C25-C24-H24	119.9
C11-C12-H12	120.0	C23-C24-H24	119.9
C12-C13-C14	119.9(6)	C24-C25-C26	120.0(6)
C12-C13-H13	120.1	C24-C25-H25	120.0
C14-C13-H13	120.1	C26-C25-H25	120.0
C15-C14-C13	121.4(6)	C25-C26-C21	120.8(6)
C15-C14-H14	119.3	C25-C26-C27	122.8(6)
C13-C14-H14	119.3	C21-C26-C27	116.2(6)
C14-C15-C16	119.4(6)	C28-C27-C26	124.6(6)
C14-C15-H15	120.3	C28-C27-S2	113.9(5)
C16-C15-H15	120.3	C26-C27-S2	120.9(5)
C15-C16-C11	120.7(6)	C27-C28-C20	118.3(6)
C15-C16-S1	126.5(5)	C27-C28-C29	110.2(6)
C11-C16-S1	112.8(5)	C20-C28-C29	131.1(6)
C18-C17-C8	121.2(6)	C34-C29-C30	118.5(6)
C18-C17-H17	119.4	C34-C29-C28	112.0(6)
C8-C17-H17	119.4	C30-C29-C28	129.4(6)
C17-C18-C19	121.0(6)	C31-C30-C29	118.9(6)
C17-C18-H18	119.5	C31-C30-H30	120.5
C19-C18-H18	119.5	C29-C30-H30	120.5
C20-C19-C18	118.4(6)	C32-C31-C30	120.9(7)
C20-C19-C21	121.1(6)	C32-C31-H31	119.6
C18-C19-C21	120.5(6)	C30-C31-H31	119.6
C19-C20-C28	117.8(6)	C33-C32-C31	121.2(7)
C19-C20-C9	118.1(6)	C33-C32-H32	119.4
C28-C20-C9	124.1(6)	C31-C32-H32	119.4

C32-C33-C34	118.0(6)	C46-C45-C50	117.6(6)
C32-C33-H33	121.0	C46-C45-C44	131.3(7)
C34-C33-H33	121.0	C50-C45-C44	111.0(6)
C33-C34-C29	122.4(6)	C47-C46-C45	120.5(7)
C33-C34-S2	124.9(5)	C47-C46-H46	119.8
C29-C34-S2	112.6(5)	C45-C46-H46	119.8
C44-C35-C36	124.5(6)	C48-C47-C46	121.3(8)
C44-C35-S3	113.4(5)	C48-C47-H47	119.4
C36-C35-S3	121.6(6)	C46-C47-H47	119.4
C37-C36-C41	120.9(6)	C47-C48-C49	120.5(8)
C37-C36-C35	123.2(7)	C47-C48-H48	119.8
C41-C36-C35	115.4(6)	C49-C48-H48	119.8
C38-C37-C36	120.7(7)	C48-C49-C50	118.5(8)
C38-C37-H37	119.7	C48-C49-H49	120.7
C36-C37-H37	119.7	C50-C49-H49	120.7
C37-C38-C39	120.1(7)	C49-C50-C45	121.5(7)
C37-C38-H38	119.9	C49-C50-S3	125.9(7)
C39-C38-H38	119.9	C45-C50-S3	112.5(5)
C40-C39-C38	121.0(7)	C52-C51-C42	121.2(7)
C40-C39-H39	119.5	C52-C51-H51	119.4
C38-C39-H39	119.5	C42-C51-H51	119.4
C39-C40-C41	119.9(7)	C51-C52-C53	121.8(7)
C39-C40-H40	120.0	C51-C52-H52	119.1
C41-C40-H40	120.0	C53-C52-H52	119.1
C36-C41-C40	117.3(6)	C52-C53-C54	117.4(6)
C36-C41-C42	120.9(6)	C52-C53-C55	122.4(7)
C40-C41-C42	121.7(7)	C54-C53-C55	120.2(6)
C43-C42-C41	120.6(6)	C43-C54-C53	119.7(6)
C43-C42-C51	117.2(6)	C43-C54-C62	123.4(7)
C41-C42-C51	122.2(6)	C53-C54-C62	116.9(7)
C54-C43-C42	118.7(7)	C60-C55-C56	118.8(7)
C54-C43-C44	124.2(6)	C60-C55-C53	120.2(7)
C42-C43-C44	117.1(6)	C56-C55-C53	121.0(7)
C35-C44-C43	118.8(6)	C57-C56-C55	120.4(8)
C35-C44-C45	111.8(7)	C57-C56-H56	119.8
C43-C44-C45	129.3(6)	C55-C56-H56	119.8

C56-C57-C58	120.1(8)	C78-C69-C70	123.0(6)
C56-C57-H57	120.0	C78-C69-S5	114.3(5)
C58-C57-H57	120.0	C70-C69-S5	122.4(5)
C59-C58-C57	120.0(9)	C75-C70-C69	117.2(6)
C59-C58-H58	120.0	C75-C70-C71	120.1(6)
C57-C58-H58	120.0	C69-C70-C71	122.2(6)
C58-C59-C60	121.5(9)	C72-C71-C70	120.3(6)
C58-C59-H59	119.3	C72-C71-H71	119.8
C60-C59-H59	119.3	C70-C71-H71	119.8
C55-C60-C59	119.2(8)	C71-C72-C73	120.1(6)
C55-C60-C61	117.5(7)	C71-C72-H72	119.9
C59-C60-C61	123.0(8)	C73-C72-H72	119.9
C62-C61-C60	123.0(7)	C74-C73-C72	120.1(7)
C62-C61-S4	113.0(6)	C74-C73-H73	120.0
C60-C61-S4	123.2(6)	C72-C73-H73	120.0
C61-C62-C54	118.8(7)	C73-C74-C75	122.5(6)
C61-C62-C63	111.6(6)	C73-C74-H74	118.7
C54-C62-C63	129.3(7)	C75-C74-H74	118.7
C64-C63-C68	117.7(7)	C74-C75-C70	116.8(6)
C64-C63-C62	131.6(6)	C74-C75-C76	123.9(6)
C68-C63-C62	110.5(7)	C70-C75-C76	119.3(6)
C65-C64-C63	121.1(7)	C77-C76-C85	118.4(6)
C65-C64-H64	119.5	C77-C76-C75	121.4(6)
C63-C64-H64	119.5	C85-C76-C75	120.2(6)
C64-C65-C66	121.1(9)	C76-C77-C88	118.4(6)
C64-C65-H65	119.4	C76-C77-C78	117.1(6)
C66-C65-H65	119.4	C88-C77-C78	124.4(6)
C67-C66-C65	119.5(8)	C69-C78-C77	119.2(6)
C67-C66-H66	120.2	C69-C78-C79	110.0(6)
C65-C66-H66	120.2	C77-C78-C79	130.6(6)
C66-C67-C68	120.2(8)	C84-C79-C80	117.0(5)
C66-C67-H67	119.9	C84-C79-C78	111.9(6)
C68-C67-H67	119.9	C80-C79-C78	130.9(6)
C67-C68-C63	120.3(8)	C81-C80-C79	119.3(6)
C67-C68-S4	126.6(7)	C81-C80-H80	120.3
C63-C68-S4	113.0(6)	C79-C80-H80	120.3

C80-C81-C82	121.6(6)	C92-C93-C94	119.1(7)
C80-C81-H81	119.2	C92-C93-H93	120.5
C82-C81-H81	119.2	C94-C93-H93	120.5
C83-C82-C81	120.4(6)	C89-C94-C93	121.0(6)
C83-C82-H82	119.8	C89-C94-C95	118.2(6)
C81-C82-H82	119.8	C93-C94-C95	120.4(6)
C82-C83-C84	118.1(6)	C96-C95-C94	121.7(6)
C82-C83-H83	120.9	C96-C95-S6	115.3(5)
C84-C83-H83	120.9	C94-C95-S6	122.4(5)
C83-C84-C79	123.2(6)	C95-C96-C88	120.0(6)
C83-C84-S5	124.1(5)	C95-C96-C97	109.9(6)
C79-C84-S5	112.6(5)	C88-C96-C97	129.9(6)
C86-C85-C76	122.3(6)	C102-C97-C98	117.9(6)
C86-C85-H85	118.9	C102-C97-C96	111.4(6)
C76-C85-H85	118.9	C98-C97-C96	130.5(6)
C85-C86-C87	121.2(6)	C99-C98-C97	118.9(7)
C85-C86-H86	119.4	C99-C98-H98	120.5
C87-C86-H86	119.4	C97-C98-H98	120.5
C86-C87-C88	117.4(6)	C98-C99-C100	122.2(6)
C86-C87-C89	121.2(6)	C98-C99-H99	118.9
C88-C87-C89	121.3(6)	C100-C99-H99	118.9
C87-C88-C77	119.7(6)	C101-C100-C99	119.5(7)
C87-C88-C96	115.7(6)	C101-C100-H100	120.2
C77-C88-C96	124.5(6)	C99-C100-H100	120.2
C94-C89-C90	117.9(6)	C102-C101-C100	118.0(7)
C94-C89-C87	119.5(6)	C102-C101-H101	121.0
C90-C89-C87	122.6(6)	C100-C101-H101	121.0
C91-C90-C89	121.5(7)	C101-C102-C97	123.3(6)
C91-C90-H90	119.3	C101-C102-S6	123.7(6)
C89-C90-H90	119.3	C97-C102-S6	112.9(5)
C90-C91-C92	120.7(6)	C16-S1-C1	91.2(3)
C90-C91-H91	119.6	C34-S2-C27	90.8(3)
C92-C91-H91	119.6	C50-S3-C35	90.9(4)
C91-C92-C93	119.7(6)	C68-S4-C61	91.6(4)
C91-C92-H92	120.1	C69-S5-C84	90.5(3)
C93-C92-H92	120.1	C95-S6-C102	90.0(3)

Table **10**. Anisotropic displacement parameters ($Å^2$x 10^3) for **14**. The anisotropic displacement factor exponent takes the form: $-2\pi^2[h^2 a^{*2}U^{11} + ... + 2 h k a^* b^* U^{12}]$

	U^{11}	U^{22}	U^{33}	U^{23}	U^{13}	U^{12}
C1	27(4)	25(4)	32(4)	-1(3)	-3(3)	-5(3)
C2	33(5)	38(5)	40(5)	6(4)	4(4)	-4(4)
C3	33(4)	39(4)	38(4)	12(4)	-2(4)	-5(4)
C4	42(5)	48(5)	33(4)	9(4)	-5(4)	-3(4)
C5	44(5)	47(5)	54(5)	25(4)	-15(4)	1(4)
C6	35(5)	27(4)	55(5)	14(4)	-1(4)	6(3)
C7	19(4)	34(4)	41(4)	4(4)	10(3)	-8(3)
C8	29(4)	30(4)	37(4)	0(4)	-2(3)	1(3)
C9	31(4)	30(4)	23(4)	2(3)	1(3)	-1(3)
C10	33(4)	31(4)	30(4)	-3(3)	-1(3)	0(3)
C11	32(4)	23(4)	30(4)	-5(3)	-3(3)	-2(3)
C12	25(4)	29(4)	33(4)	0(3)	-4(3)	-3(3)
C13	40(5)	44(5)	42(5)	10(4)	-6(4)	0(4)
C14	47(5)	34(5)	53(5)	12(4)	-12(4)	-10(4)
C15	44(5)	26(4)	45(5)	-3(4)	-7(4)	0(3)
C16	38(5)	31(4)	36(4)	3(4)	-2(3)	-6(4)
C17	34(4)	24(4)	44(5)	2(4)	-2(4)	-2(3)
C18	37(5)	23(4)	45(5)	-7(3)	7(4)	-6(3)
C19	31(4)	24(4)	32(4)	0(3)	6(3)	-3(3)
C20	27(4)	25(4)	30(4)	-1(3)	4(3)	-1(3)
C21	29(4)	36(4)	39(4)	2(4)	3(4)	-13(4)
C22	35(5)	31(4)	49(5)	-4(4)	7(4)	-11(3)
C23	33(5)	38(5)	49(5)	-11(4)	0(4)	-14(4)
C24	35(5)	46(5)	47(5)	-7(4)	-4(4)	-3(4)
C25	29(4)	34(4)	45(5)	6(4)	-9(4)	-7(3)
C26	27(4)	39(5)	32(4)	-4(4)	7(3)	-8(4)
C27	29(4)	30(4)	38(4)	3(3)	-4(4)	-1(3)
C28	29(4)	22(4)	39(4)	6(3)	-1(4)	-3(3)
C29	29(4)	31(4)	30(4)	-2(3)	3(3)	-6(3)
C30	26(4)	38(5)	41(5)	3(4)	5(3)	7(3)

C31	43(5)	42(5)	45(5)	-8(4)	-2(4)	0(4)
C32	50(5)	33(4)	54(5)	-9(4)	-2(4)	16(4)
C33	46(5)	41(5)	54(5)	4(4)	-15(4)	13(4)
C34	49(5)	27(4)	43(5)	5(4)	-7(4)	0(4)
C35	31(4)	36(4)	36(4)	-6(4)	-7(3)	-7(4)
C36	26(4)	25(4)	52(5)	-12(4)	-11(4)	5(3)
C37	39(5)	28(4)	41(5)	-10(4)	-12(4)	5(4)
C38	32(5)	32(5)	60(5)	-23(4)	-16(4)	6(4)
C39	32(5)	27(4)	63(6)	-3(4)	-10(4)	-1(4)
C40	37(5)	29(4)	49(5)	-7(4)	-12(4)	-2(4)
C41	28(4)	20(4)	47(5)	-8(4)	-12(4)	-4(3)
C42	26(4)	27(4)	38(5)	-13(4)	-9(4)	1(3)
C43	29(4)	20(4)	50(5)	-11(4)	-17(4)	4(3)
C44	42(5)	31(4)	29(4)	-3(4)	-3(4)	2(4)
C45	41(5)	22(4)	43(5)	2(4)	-17(4)	-8(3)
C46	37(5)	30(5)	50(5)	-1(4)	-19(4)	-2(4)
C47	55(6)	44(5)	58(6)	1(5)	-17(5)	6(4)
C48	65(6)	44(6)	60(6)	11(5)	-20(5)	-5(5)
C49	61(6)	69(6)	34(5)	13(5)	-9(4)	-7(5)
C50	53(5)	35(5)	44(5)	-3(4)	-12(4)	3(4)
C51	20(4)	31(4)	54(5)	-7(4)	-8(4)	-7(3)
C52	25(4)	38(5)	47(5)	-3(4)	-9(4)	-14(4)
C53	22(4)	27(4)	55(5)	-7(4)	-7(4)	-1(3)
C54	31(5)	18(4)	49(5)	-1(3)	-6(4)	-13(3)
C55	39(5)	30(4)	54(5)	-11(4)	2(4)	-13(4)
C56	32(5)	43(5)	84(7)	-22(5)	2(5)	-11(4)
C57	37(5)	68(6)	79(7)	-33(6)	9(5)	-19(5)
C58	35(5)	45(6)	117(9)	-37(6)	13(6)	-13(4)
C59	33(5)	26(5)	117(8)	-22(5)	2(5)	-6(4)
C60	41(5)	25(4)	84(7)	-18(5)	-7(5)	-5(4)
C61	33(5)	22(4)	88(7)	5(4)	-13(5)	-8(4)
C62	30(5)	18(4)	70(6)	0(4)	-12(4)	-4(3)
C63	37(5)	29(4)	61(6)	14(4)	-23(4)	-12(4)
C64	53(5)	33(5)	57(5)	3(4)	-25(4)	1(4)
C65	70(6)	57(6)	58(6)	20(5)	-30(5)	-11(5)
C66	84(8)	79(7)	61(7)	19(6)	-34(6)	-23(6)

C67	45(6)	86(8)	97(8)	42(6)	-29(6)	3(5)
C68	43(5)	38(5)	69(6)	8(4)	-8(5)	0(4)
C69	26(4)	20(4)	41(5)	0(3)	-11(3)	-3(3)
C70	27(4)	30(4)	40(5)	9(4)	-5(3)	6(3)
C71	25(4)	20(4)	51(5)	4(4)	-11(4)	-5(3)
C72	37(5)	33(4)	42(5)	15(4)	3(4)	-3(4)
C73	31(4)	34(4)	41(5)	5(4)	-8(4)	-3(4)
C74	33(4)	24(4)	46(5)	4(4)	-13(4)	-3(3)
C75	27(4)	18(4)	39(5)	8(3)	-9(4)	0(3)
C76	30(4)	23(4)	35(5)	1(3)	-11(4)	3(3)
C77	26(4)	17(4)	41(5)	-1(3)	-8(4)	-2(3)
C78	31(4)	21(4)	35(4)	5(3)	-2(3)	3(3)
C79	21(4)	31(4)	34(4)	-1(3)	-8(3)	-1(3)
C80	40(5)	21(4)	40(5)	2(3)	-9(4)	9(3)
C81	41(5)	27(4)	33(4)	4(3)	-9(4)	2(3)
C82	36(5)	43(5)	28(4)	2(4)	-6(3)	4(4)
C83	40(5)	31(4)	38(5)	1(4)	-13(4)	-1(4)
C84	36(4)	24(4)	31(4)	10(3)	-5(3)	-5(3)
C85	39(5)	23(4)	31(4)	7(3)	-11(3)	6(3)
C86	24(4)	34(4)	36(5)	-2(3)	-7(3)	7(3)
C87	22(4)	21(4)	35(4)	-6(3)	-4(3)	4(3)
C88	28(4)	23(4)	37(4)	10(3)	-8(4)	9(3)
C89	24(4)	17(4)	43(5)	5(3)	-6(4)	0(3)
C90	30(4)	25(4)	44(4)	1(4)	-8(3)	-2(3)
C91	36(5)	24(4)	42(5)	-3(3)	-4(4)	0(4)
C92	34(5)	23(4)	52(5)	4(4)	-10(4)	-1(3)
C93	34(5)	34(4)	43(5)	13(4)	-13(4)	-4(4)
C94	28(4)	25(4)	41(5)	3(4)	-2(4)	-1(3)
C95	28(4)	27(4)	36(4)	-2(3)	-10(3)	-2(3)
C96	26(4)	17(4)	41(4)	6(3)	-8(3)	-3(3)
C97	28(4)	24(4)	33(4)	-3(3)	-8(3)	5(3)
C98	44(5)	20(4)	42(4)	0(4)	-11(4)	0(3)
C99	37(5)	20(4)	47(5)	5(4)	-7(4)	1(3)
C100	44(5)	28(4)	48(5)	2(4)	-5(4)	1(4)
C101	38(5)	39(5)	39(4)	5(4)	-4(4)	9(4)
C102	26(4)	17(4)	49(5)	0(3)	-3(4)	4(3)

S1	44(1)	31(1)	43(1)	2(1)	-10(1)	-9(1)
S2	40(1)	33(1)	44(1)	2(1)	-9(1)	6(1)
S3	55(1)	46(1)	45(1)	-4(1)	-2(1)	2(1)
S4	44(1)	38(1)	113(2)	20(1)	-10(1)	10(1)
S5	39(1)	29(1)	43(1)	2(1)	-8(1)	-7(1)
S6	38(1)	27(1)	44(1)	1(1)	1(1)	-1(1)

Table 11. Hydrogen coordinates (x 10^4) and isotropic displacement parameters ($Å^2$x 10^3) for **14**.

	x	y	z	U(eq)
H3	6134	1099	6164	44
H4	7026	187	6440	49
H5	6405	-800	6184	58
H6	4887	-868	5667	47
H12	2858	1646	4509	35
H13	3244	2657	4280	51
H14	4452	3405	4643	54
H15	5374	3154	5219	46
H17	3553	-895	5216	41
H18	2122	-937	4691	42
H22	796	-962	4228	46
H23	-900	-998	3750	48
H24	-2257	-113	3587	52
H25	-1835	836	3887	44
H30	1597	1554	5495	42
H31	641	2448	5770	52
H32	-985	3089	5449	55
H33	-1871	2808	4868	57
H37	8041	2319	6193	44
H38	8711	1426	6511	50
H39	7748	1217	7083	49
H40	6112	1906	7343	46
H46	4480	4958	6678	47
H47	5032	5829	6332	64
H48	6356	5736	5807	68
H49	7381	4768	5650	66
H51	4765	2558	7569	42
H52	3377	3323	7822	45
H56	2099	3999	8036	64
H57	479	4731	8262	74

H58	-564	5499	7868	78
H59	-52	5506	7261	70
H64	3349	3416	6166	58
H65	2577	3434	5562	75
H66	967	4206	5343	90
H67	-39	4903	5752	92
H71	7948	3827	2212	39
H72	8663	3745	1610	45
H73	7799	2922	1239	43
H74	6167	2206	1463	42
H80	4835	1703	3334	40
H81	5495	1848	3945	41
H82	6721	2747	4141	43
H83	7625	3445	3713	44
H85	4737	1594	1665	37
H86	3320	822	1900	38
H90	1985	156	2104	40
H91	495	-592	2347	41
H92	-331	-491	2943	44
H93	322	399	3296	45
H98	3314	3176	2827	43
H99	2561	4001	3191	42
H100	1262	3828	3720	48
H101	477	2803	3860	46

Table 12. Torsion angles [°] for **14**.

C10-C1-C2-C3	179.6(6)	C9-C10-C11-C16	179.4(6)
S1-C1-C2-C3	7.7(9)	C16-C11-C12-C13	-4.0(9)
C10-C1-C2-C7	5.9(10)	C10-C11-C12-C13	-178.9(6)
S1-C1-C2-C7	-166.1(5)	C11-C12-C13-C14	0.7(10)
C7-C2-C3-C4	2.9(10)	C12-C13-C14-C15	1.9(11)
C1-C2-C3-C4	-170.6(6)	C13-C14-C15-C16	-1.0(11)
C2-C3-C4-C5	-0.8(10)	C14-C15-C16-C11	-2.5(10)
C3-C4-C5-C6	-0.8(11)	C14-C15-C16-S1	174.4(6)
C4-C5-C6-C7	0.4(11)	C12-C11-C16-C15	5.0(10)
C3-C2-C7-C6	-3.3(10)	C10-C11-C16-C15	-179.1(6)
C1-C2-C7-C6	170.6(6)	C12-C11-C16-S1	-172.3(5)
C3-C2-C7-C8	175.9(6)	C10-C11-C16-S1	3.5(7)
C1-C2-C7-C8	-10.2(9)	C9-C8-C17-C18	-0.9(10)
C5-C6-C7-C2	1.6(10)	C7-C8-C17-C18	-178.9(6)
C5-C6-C7-C8	-177.6(6)	C8-C17-C18-C19	-8.6(10)
C2-C7-C8-C9	-0.2(9)	C17-C18-C19-C20	2.0(10)
C6-C7-C8-C9	178.9(6)	C17-C18-C19-C21	-177.3(6)
C2-C7-C8-C17	177.7(6)	C18-C19-C20-C28	-164.2(6)
C6-C7-C8-C17	-3.2(10)	C21-C19-C20-C28	15.1(9)
C17-C8-C9-C20	16.5(9)	C18-C19-C20-C9	13.5(9)
C7-C8-C9-C20	-165.5(6)	C21-C19-C20-C9	-167.2(6)
C17-C8-C9-C10	-163.0(6)	C8-C9-C20-C19	-22.7(9)
C7-C8-C9-C10	15.0(9)	C10-C9-C20-C19	156.7(6)
C2-C1-C10-C9	9.0(10)	C8-C9-C20-C28	154.8(6)
S1-C1-C10-C9	-178.3(5)	C10-C9-C20-C28	-25.8(10)
C2-C1-C10-C11	-165.5(6)	C20-C19-C21-C22	176.2(6)
S1-C1-C10-C11	7.1(7)	C18-C19-C21-C22	-4.5(10)
C8-C9-C10-C1	-19.3(9)	C20-C19-C21-C26	-4.5(9)
C20-C9-C10-C1	161.2(6)	C18-C19-C21-C26	174.8(6)
C8-C9-C10-C11	154.1(6)	C26-C21-C22-C23	-1.3(10)
C20-C9-C10-C11	-25.4(11)	C19-C21-C22-C23	178.1(6)
C1-C10-C11-C12	168.5(6)	C21-C22-C23-C24	0.6(10)
C9-C10-C11-C12	-5.4(12)	C22-C23-C24-C25	1.5(11)
C1-C10-C11-C16	-6.7(8)	C23-C24-C25-C26	-2.8(10)

C24-C25-C26-C21	2.0(10)	S3-C35-C36-C41	166.5(4)
C24-C25-C26-C27	-173.7(6)	C41-C36-C37-C38	1.1(9)
C22-C21-C26-C25	0.0(9)	C35-C36-C37-C38	172.7(6)
C19-C21-C26-C25	-179.4(6)	C36-C37-C38-C39	0.1(10)
C22-C21-C26-C27	176.0(6)	C37-C38-C39-C40	-0.4(10)
C19-C21-C26-C27	-3.4(9)	C38-C39-C40-C41	-0.6(10)
C25-C26-C27-C28	176.0(6)	C37-C36-C41-C40	-2.1(9)
C21-C26-C27-C28	0.1(10)	C35-C36-C41-C40	-174.3(5)
C25-C26-C27-S2	5.5(9)	C37-C36-C41-C42	-179.0(6)
C21-C26-C27-S2	-170.4(5)	C35-C36-C41-C42	8.8(9)
C26-C27-C28-C20	10.7(10)	C39-C40-C41-C36	1.8(9)
S2-C27-C28-C20	-178.2(5)	C39-C40-C41-C42	178.7(6)
C26-C27-C28-C29	-163.7(6)	C36-C41-C42-C43	1.0(9)
S2-C27-C28-C29	7.4(7)	C40-C41-C42-C43	-175.8(5)
C19-C20-C28-C27	-17.9(9)	C36-C41-C42-C51	-177.3(6)
C9-C20-C28-C27	164.5(6)	C40-C41-C42-C51	5.9(9)
C19-C20-C28-C29	155.1(6)	C41-C42-C43-C54	166.9(6)
C9-C20-C28-C29	-22.4(11)	C51-C42-C43-C54	-14.7(8)
C27-C28-C29-C34	-6.1(8)	C41-C42-C43-C44	-15.1(8)
C20-C28-C29-C34	-179.5(6)	C51-C42-C43-C44	163.2(5)
C27-C28-C29-C30	169.3(6)	C36-C35-C44-C43	-9.7(10)
C20-C28-C29-C30	-4.2(11)	S3-C35-C44-C43	178.6(4)
C34-C29-C30-C31	-1.6(9)	C36-C35-C44-C45	166.1(6)
C28-C29-C30-C31	-176.7(6)	S3-C35-C44-C45	-5.7(7)
C29-C30-C31-C32	-1.6(10)	C54-C43-C44-C35	-163.0(6)
C30-C31-C32-C33	3.3(11)	C42-C43-C44-C35	19.2(9)
C31-C32-C33-C34	-1.6(11)	C54-C43-C44-C45	22.1(10)
C32-C33-C34-C29	-1.7(11)	C42-C43-C44-C45	-155.7(6)
C32-C33-C34-S2	175.0(6)	C35-C44-C45-C46	-169.1(6)
C30-C29-C34-C33	3.3(10)	C43-C44-C45-C46	6.0(11)
C28-C29-C34-C33	179.2(6)	C35-C44-C45-C50	6.4(8)
C30-C29-C34-S2	-173.7(5)	C43-C44-C45-C50	-178.4(6)
C28-C29-C34-S2	2.2(7)	C50-C45-C46-C47	2.7(9)
C44-C35-C36-C37	-176.6(6)	C44-C45-C46-C47	178.1(6)
S3-C35-C36-C37	-5.4(9)	C45-C46-C47-C48	1.1(10)
C44-C35-C36-C41	-4.6(9)	C46-C47-C48-C49	-3.7(11)

C47-C48-C49-C50	2.2(11)	C59-C60-C61-C62	-179.3(7)
C48-C49-C50-C45	1.8(11)	C55-C60-C61-S4	164.2(5)
C48-C49-C50-S3	-173.7(6)	C59-C60-C61-S4	-9.8(10)
C46-C45-C50-C49	-4.2(10)	C60-C61-C62-C54	-9.6(10)
C44-C45-C50-C49	179.6(6)	S4-C61-C62-C54	179.9(5)
C46-C45-C50-S3	171.9(5)	C60-C61-C62-C63	165.1(6)
C44-C45-C50-S3	-4.4(7)	S4-C61-C62-C63	-5.4(7)
C43-C42-C51-C52	-2.1(9)	C43-C54-C62-C61	-162.6(6)
C41-C42-C51-C52	176.3(6)	C53-C54-C62-C61	20.9(9)
C42-C51-C52-C53	10.4(9)	C43-C54-C62-C63	23.8(10)
C51-C52-C53-C54	-1.6(9)	C53-C54-C62-C63	-152.7(7)
C51-C52-C53-C55	177.3(6)	C61-C62-C63-C64	-170.8(7)
C42-C43-C54-C53	23.7(8)	C54-C62-C63-C64	3.2(12)
C44-C43-C54-C53	-154.1(6)	C61-C62-C63-C68	4.3(8)
C42-C43-C54-C62	-152.7(6)	C54-C62-C63-C68	178.3(7)
C44-C43-C54-C62	29.5(9)	C68-C63-C64-C65	2.1(11)
C52-C53-C54-C43	-15.5(9)	C62-C63-C64-C65	176.9(7)
C55-C53-C54-C43	165.6(6)	C63-C64-C65-C66	0.4(12)
C52-C53-C54-C62	161.2(5)	C64-C65-C66-C67	-3.2(13)
C55-C53-C54-C62	-17.8(8)	C65-C66-C67-C68	3.4(14)
C52-C53-C55-C60	-175.7(6)	C66-C67-C68-C63	-0.9(13)
C54-C53-C55-C60	3.2(9)	C66-C67-C68-S4	-176.7(7)
C52-C53-C55-C56	6.4(10)	C64-C63-C68-C67	-1.8(11)
C54-C53-C55-C56	-174.7(6)	C62-C63-C68-C67	-177.7(7)
C60-C55-C56-C57	0.8(10)	C64-C63-C68-S4	174.5(5)
C53-C55-C56-C57	178.8(6)	C62-C63-C68-S4	-1.3(7)
C55-C56-C57-C58	-1.8(11)	C78-C69-C70-C75	-5.7(9)
C56-C57-C58-C59	1.2(11)	S5-C69-C70-C75	167.5(5)
C57-C58-C59-C60	0.5(12)	C78-C69-C70-C71	-177.2(6)
C56-C55-C60-C59	0.9(10)	S5-C69-C70-C71	-4.0(9)
C53-C55-C60-C59	-177.2(6)	C75-C70-C71-C72	-2.6(9)
C56-C55-C60-C61	-173.4(6)	C69-C70-C71-C72	168.7(6)
C53-C55-C60-C61	8.5(10)	C70-C71-C72-C73	1.2(9)
C58-C59-C60-C55	-1.5(11)	C71-C72-C73-C74	1.2(9)
C58-C59-C60-C61	172.4(7)	C72-C73-C74-C75	-2.4(10)
C55-C60-C61-C62	-5.3(10)	C73-C74-C75-C70	1.0(9)

C73-C74-C75-C76	179.6(6)	C77-C76-C85-C86	-2.3(9)
C69-C70-C75-C74	-170.2(5)	C75-C76-C85-C86	177.4(6)
C71-C70-C75-C74	1.5(9)	C76-C85-C86-C87	8.4(9)
C69-C70-C75-C76	11.0(8)	C85-C86-C87-C88	-0.7(9)
C71-C70-C75-C76	-177.3(5)	C85-C86-C87-C89	-180.0(6)
C74-C75-C76-C77	180.0(6)	C86-C87-C88-C77	-12.7(8)
C70-C75-C76-C77	-1.4(9)	C89-C87-C88-C77	166.6(5)
C74-C75-C76-C85	0.3(9)	C86-C87-C88-C96	164.1(5)
C70-C75-C76-C85	178.9(5)	C89-C87-C88-C96	-16.6(8)
C85-C76-C77-C88	-11.0(8)	C76-C77-C88-C87	18.6(8)
C75-C76-C77-C88	169.3(5)	C78-C77-C88-C87	-158.4(6)
C85-C76-C77-C78	166.2(5)	C76-C77-C88-C96	-157.9(6)
C75-C76-C77-C78	-13.5(8)	C78-C77-C88-C96	25.1(9)
C70-C69-C78-C77	-9.4(9)	C86-C87-C89-C94	-179.6(6)
S5-C69-C78-C77	176.9(4)	C88-C87-C89-C94	1.2(9)
C70-C69-C78-C79	165.2(6)	C86-C87-C89-C90	1.4(9)
S5-C69-C78-C79	-8.5(7)	C88-C87-C89-C90	-177.8(6)
C76-C77-C78-C69	18.7(8)	C94-C89-C90-C91	-0.8(9)
C88-C77-C78-C69	-164.2(6)	C87-C89-C90-C91	178.2(6)
C76-C77-C78-C79	-154.6(6)	C89-C90-C91-C92	-0.6(10)
C88-C77-C78-C79	22.5(10)	C90-C91-C92-C93	0.9(9)
C69-C78-C79-C84	8.3(7)	C91-C92-C93-C94	0.2(9)
C77-C78-C79-C84	-178.0(6)	C90-C89-C94-C93	1.9(9)
C69-C78-C79-C80	-166.8(6)	C87-C89-C94-C93	-177.1(6)
C77-C78-C79-C80	7.0(11)	C90-C89-C94-C95	-170.7(5)
C84-C79-C80-C81	4.2(9)	C87-C89-C94-C95	10.2(9)
C78-C79-C80-C81	179.1(6)	C92-C93-C94-C89	-1.6(9)
C79-C80-C81-C82	0.6(10)	C92-C93-C94-C95	170.9(6)
C80-C81-C82-C83	-5.3(10)	C89-C94-C95-C96	-5.6(9)
C81-C82-C83-C84	4.6(10)	C93-C94-C95-C96	-178.3(6)
C82-C83-C84-C79	0.5(10)	C89-C94-C95-S6	165.4(5)
C82-C83-C84-S5	-175.2(5)	C93-C94-C95-S6	-7.3(8)
C80-C79-C84-C83	-4.9(9)	C94-C95-C96-C88	-10.6(9)
C78-C79-C84-C83	179.3(6)	S6-C95-C96-C88	177.7(4)
C80-C79-C84-S5	171.3(5)	C94-C95-C96-C97	164.4(5)
C78-C79-C84-S5	-4.6(7)	S6-C95-C96-C97	-7.3(7)

C87-C88-C96-C95	21.2(8)	C2-C1-S1-C16	168.3(6)
C77-C88-C96-C95	-162.2(6)	C33-C34-S2-C27	-175.4(7)
C87-C88-C96-C97	-152.6(6)	C29-C34-S2-C27	1.6(5)
C77-C88-C96-C97	24.0(10)	C28-C27-S2-C34	-5.3(5)
C95-C96-C97-C102	6.5(7)	C26-C27-S2-C34	166.1(6)
C88-C96-C97-C102	-179.2(6)	C49-C50-S3-C35	176.9(7)
C95-C96-C97-C98	-167.5(6)	C45-C50-S3-C35	1.1(5)
C88-C96-C97-C98	6.8(11)	C44-C35-S3-C50	2.7(5)
C102-C97-C98-C99	3.2(9)	C36-C35-S3-C50	-169.3(5)
C96-C97-C98-C99	176.9(6)	C67-C68-S4-C61	174.7(7)
C97-C98-C99-C100	0.9(9)	C63-C68-S4-C61	-1.4(6)
C98-C99-C100-C101	-3.7(10)	C62-C61-S4-C68	4.0(6)
C99-C100-C101-C102	2.1(9)	C60-C61-S4-C68	-166.5(6)
C100-C101-C102-C97	2.2(9)	C78-C69-S5-C84	5.2(5)
C100-C101-C102-S6	-174.1(5)	C70-C69-S5-C84	-168.5(5)
C98-C97-C102-C101	-4.9(9)	C83-C84-S5-C69	176.0(6)
C96-C97-C102-C101	-179.7(6)	C79-C84-S5-C69	-0.1(5)
C98-C97-C102-S6	171.7(4)	C96-C95-S6-C102	4.8(5)
C96-C97-C102-S6	-3.1(7)	C94-C95-S6-C102	-166.8(5)
C15-C16-S1-C1	-176.9(7)	C101-C102-S6-C95	175.9(6)
C11-C16-S1-C1	0.3(5)	C97-C102-S6-C95	-0.8(5)
C10-C1-S1-C16	-4.4(5)		

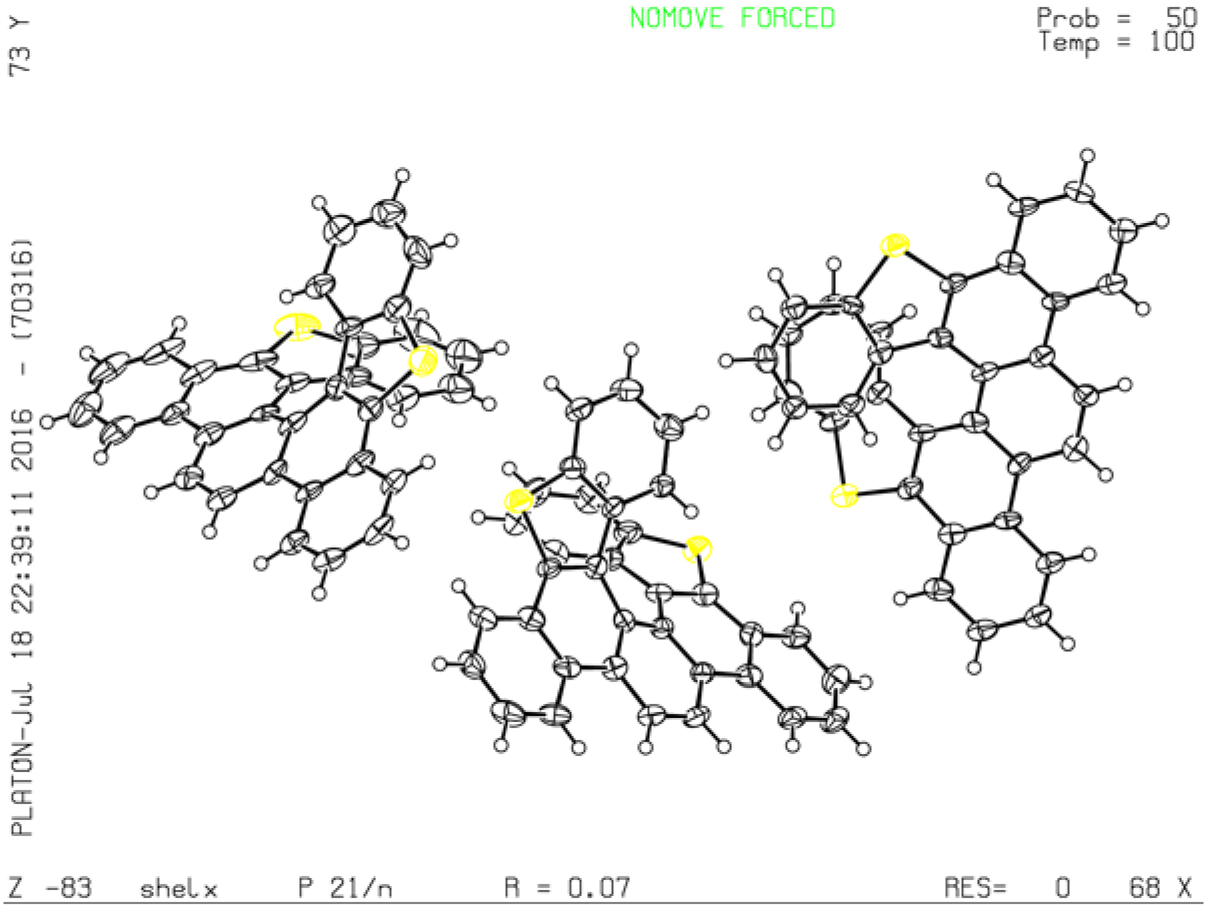

73 Y
NOMOVE FORCED
Prob = 50
Temp = 100
PLATON-Jul 18 22:39:11 2016 - (70316)
Z -83 shelx P 21/n R = 0.07 RES= 0 68 X

X-ray Experimental for complex **16a**:

X-ray Experimental for complex $C_{60}H_{38}$: Crystals grew as thin, yellow plates by slow evaporation from DCM/ Hexanes. The data crystal had approximate dimensions; 0.18 x 0.07 x 0.03 mm. The data were collected on an Agilent Technologies SuperNova Dual Source diffractometer using a μ-focus Cu Kα radiation source (λ = 1.5418Å) with collimating mirror monochromators. A total of 1160 frames of data were collected using □-scans with a scan range of 1° and a counting time of 25 seconds per frame with a detector offset of +/- 38.4° and 80 seconds per frame with a detector offset of +/- 110.1°. The data were collected at 100 K using an Oxford 700 Cryostream low temperature device. Details of crystal data, data collection and structure refinement are listed in Table 13. Data collection, unit cell refinement and data reduction were performed using Agilent Technologies CrysAlisPro V 1.171.38.43f. The structure was solved by direct methods using SHELXT and refined by full-matrix least-squares on F^2 with anisotropic displacement parameters for the non-H atoms using SHELXL-2016/6. Structure analysis was aided by use of the programs PLATON98 and WinGX. The hydrogen atoms were calculated in ideal positions with isotropic displacement parameters set to 1.2xUeq of the attached atom (1.5xUeq for methyl hydrogen atoms). The function, $\Sigma w(|F_o|^2 - |F_c|^2)^2$, was minimized, where w = 1/[(□(F_o))^2 + (0.0229*P)^2] and P = ($|F_o|^2 + 2|F_c|^2$)/3. $R_w(F^2)$ refined to 0.272, with R(F) equal to 0.0900 and a goodness of fit, S, = 0.938. Definitions used for calculating R(F), $R_w(F^2)$ and the goodness of fit, S, are given below. The data were checked for secondary extinction effects but no correction was necessary. Neutral atom scattering factors and values used to calculate the linear absorption coefficient are from the International Tables for X-ray Crystallography (1992). All figures were generated using SHELXTL/PC. Tables of positional and thermal parameters, bond lengths and angles, torsion angles and figures are found elsewhere.

Table 13. Crystal data and structure refinement for **16a**.

Empirical formula	C60 H38	
Formula weight	758.90	
Temperature	100(2) K	
Wavelength	1.54184 Å	
Crystal system	triclinic	
Space group	P -1	
Unit cell dimensions	a = 9.859(4) Å	α= 91.78(3)°.
	b = 13.666(4) Å	β= 96.21(3)°.
	c = 14.502(6) Å	γ = 110.33(3)°.
Volume	1816.5(12) $Å^3$	
Z	2	
Density (calculated)	1.387 Mg/m^3	
Absorption coefficient	0.597 mm^{-1}	
F(000)	796	
Crystal size	0.18 x 0.069 x 0.025 mm^3	
Theta range for data collection	4.456 to 58.934°.	
Index ranges	-10<=h<=10, -15<=k<=10, -15<=l<=16	
Reflections collected	8555	
Independent reflections	5170 [R(int) = 0.0982]	
Completeness to theta = 58.934°	99.3 %	
Absorption correction	Semi-empirical from equivalents	
Max. and min. transmission	1.00 and 0.521	
Refinement method	Full-matrix least-squares on F^2	
Data / restraints / parameters	5170 / 360 / 547	
Goodness-of-fit on F^2	0.926	
Final R indices [I>2sigma(I)]	R1 = 0.0900, wR2 = 0.1676	
R indices (all data)	R1 = 0.2932, wR2 = 0.2722	
Extinction coefficient	n/a	
Largest diff. peak and hole	0.304 and -0.260 e.$Å^{-3}$	

Table 14. Atomic coordinates (x 10^4) and equivalent isotropic displacement parameters ($Å^2$x 10^3) for **16a**. U(eq) is defined as one third of the trace of the orthogonalized U^{ij} tensor.

	x	y	z	U(eq)
C1	7978(13)	9660(7)	2766(7)	46(3)
C2	7155(14)	10016(8)	3309(7)	58(3)
C3	6448(13)	9456(8)	4015(7)	55(3)
C4	6639(12)	8482(7)	4213(7)	38(3)
C5	6057(13)	7930(8)	4994(7)	43(3)
C6	5187(13)	8237(7)	5538(7)	50(3)
C7	4795(13)	7783(8)	6346(7)	57(3)
C8	5224(12)	6966(7)	6659(7)	44(3)
C9	4913(13)	6522(8)	7549(8)	53(3)
C10	4471(15)	7088(9)	8277(9)	65(4)
C11	3870(15)	6586(9)	9020(8)	62(3)
C12	3757(14)	5533(10)	9070(8)	69(4)
C13	4269(14)	4991(9)	8455(7)	55(3)
C14	4999(13)	5497(8)	7685(8)	53(3)
C15	5706(12)	5077(8)	7069(7)	45(3)
C16	6053(12)	4198(7)	7282(7)	48(3)
C17	6746(12)	3739(8)	6691(7)	41(3)
C18	7034(13)	2782(8)	6902(7)	48(3)
C19	6648(13)	2346(8)	7746(8)	57(3)
C20	6951(14)	1435(8)	7927(8)	62(3)
C21	7645(14)	1003(8)	7354(8)	66(4)
C22	7986(13)	1450(8)	6543(8)	57(3)
C23	7720(13)	2371(8)	6322(7)	48(3)
C24	8159(12)	2866(7)	5464(7)	43(3)
C25	8813(13)	2436(8)	4823(7)	53(3)
C26	9210(13)	2892(8)	4009(7)	52(3)
C27	8994(12)	3835(8)	3811(7)	47(3)
C28	9437(12)	4327(7)	2952(7)	42(3)
C29	10026(13)	3867(8)	2304(7)	54(3)
C30	10485(12)	4363(8)	1499(7)	53(3)
C31	10285(13)	5301(8)	1328(7)	55(3)

C32	9709(12)	5746(8)	1946(7)	51(3)
C33	9230(13)	5260(8)	2771(7)	47(3)
C34	8588(12)	5736(8)	3423(7)	45(3)
C35	8381(12)	6686(7)	3277(7)	43(3)
C36	7699(12)	7119(7)	3845(7)	45(3)
C37	7482(13)	8117(8)	3674(7)	45(3)
C38	8149(12)	8733(8)	2958(7)	48(3)
C39	6373(12)	7029(8)	5224(7)	43(3)
C40	5928(12)	6522(8)	6056(7)	43(3)
C41	6174(12)	5581(8)	6263(7)	45(3)
C42	6942(12)	5162(7)	5676(7)	42(3)
C43	7394(12)	5679(7)	4847(7)	42(3)
C44	7144(12)	6612(7)	4643(7)	39(3)
C45	7221(12)	4253(7)	5857(6)	38(3)
C46	7878(11)	3782(7)	5251(7)	40(3)
C47	8339(13)	4286(8)	4421(7)	48(3)
C48	8079(11)	5239(7)	4240(7)	39(3)
C49	8809(14)	10337(8)	2090(8)	59(3)
C50	10286(14)	10966(8)	2351(8)	58(3)
C51	11012(14)	11545(8)	1641(8)	60(3)
C52	10430(16)	11472(10)	749(9)	70(4)
C53	8977(16)	10877(9)	523(9)	78(4)
C54	8177(15)	10291(9)	1188(8)	64(3)
C55	10982(14)	11035(8)	3331(8)	70(4)
C56	11201(17)	12127(9)	20(9)	101(5)
C57	6622(14)	9586(9)	878(8)	78(4)
C58	4779(14)	8230(8)	8312(8)	70(4)
C59	3272(15)	7120(8)	9728(8)	78(4)
C60	4036(15)	3833(8)	8635(8)	82(5)

Table 15. Bond lengths [Å] and angles [°] for **16a**.

C1-C38	1.369(14)	C18-C23	1.356(16)
C1-C2	1.379(15)	C18-C19	1.410(13)
C1-C49	1.480(14)	C19-C20	1.403(16)
C2-C3	1.397(13)	C19-H19	0.95
C2-H2	0.95	C20-C21	1.371(17)
C3-C4	1.441(14)	C20-H20	0.95
C3-H3	0.95	C21-C22	1.363(14)
C4-C37	1.398(15)	C21-H21	0.95
C4-C5	1.439(13)	C22-C23	1.410(15)
C5-C6	1.382(15)	C22-H22	0.95
C5-C39	1.413(14)	C23-C24	1.469(13)
C6-C7	1.373(13)	C24-C25	1.408(15)
C6-H6	0.95	C24-C46	1.409(14)
C7-C8	1.395(15)	C25-C26	1.382(13)
C7-H7	0.95	C25-H25	0.95
C8-C40	1.416(15)	C26-C27	1.411(15)
C8-C9	1.461(13)	C26-H26	0.95
C9-C14	1.450(15)	C27-C47	1.394(15)
C9-C10	1.479(16)	C27-C28	1.470(12)
C10-C11	1.372(15)	C28-C33	1.388(14)
C10-C58	1.480(14)	C28-C29	1.397(14)
C11-C12	1.410(16)	C29-C30	1.407(13)
C11-C59	1.522(16)	C29-H29	0.95
C12-C13	1.385(16)	C30-C31	1.388(15)
C12-H12	0.95	C30-H30	0.95
C13-C14	1.456(14)	C31-C32	1.346(15)
C13-C60	1.552(14)	C31-H31	0.95
C14-C15	1.413(15)	C32-C33	1.432(13)
C15-C16	1.393(15)	C32-H32	0.95
C15-C41	1.414(13)	C33-C34	1.448(15)
C16-C17	1.409(15)	C34-C35	1.402(14)
C16-H16	0.95	C34-C48	1.435(12)
C17-C45	1.456(12)	C35-C36	1.359(15)
C17-C18	1.466(14)	C35-H35	0.95

C36-C44	1.428(13)	C52-C56	1.502(14)
C36-C37	1.476(13)	C53-C54	1.402(15)
C37-C38	1.427(12)	C53-H53	0.95
C38-H38	0.95	C54-C57	1.509(16)
C39-C44	1.422(15)	C55-H55A	0.98
C39-C40	1.444(12)	C55-H55B	0.98
C40-C41	1.425(15)	C55-H55C	0.98
C41-C42	1.427(15)	C56-H56A	0.98
C42-C45	1.390(14)	C56-H56B	0.98
C42-C43	1.446(12)	C56-H56C	0.98
C43-C48	1.403(14)	C57-H57A	0.98
C43-C44	1.415(14)	C57-H57B	0.98
C45-C46	1.409(14)	C57-H57C	0.98
C46-C47	1.442(13)	C58-H58A	0.98
C47-C48	1.439(14)	C58-H58B	0.98
C49-C54	1.378(16)	C58-H58C	0.98
C49-C50	1.412(16)	C59-H59A	0.98
C50-C51	1.420(15)	C59-H59B	0.98
C50-C55	1.495(15)	C59-H59C	0.98
C51-C52	1.343(16)	C60-H60A	0.98
C51-H51	0.95	C60-H60B	0.98
C52-C53	1.376(18)	C60-H60C	0.98

C38-C1-C2	117.9(9)	C6-C5-C4	123.1(10)
C38-C1-C49	121.3(11)	C39-C5-C4	119.4(10)
C2-C1-C49	120.2(10)	C7-C6-C5	122.2(11)
C1-C2-C3	123.1(10)	C7-C6-H6	118.9
C1-C2-H2	118.5	C5-C6-H6	118.9
C3-C2-H2	118.4	C6-C7-C8	122.0(12)
C2-C3-C4	118.5(11)	C6-C7-H7	119.0
C2-C3-H3	120.7	C8-C7-H7	119.0
C4-C3-H3	120.7	C7-C8-C40	117.7(9)
C37-C4-C3	118.9(9)	C7-C8-C9	123.1(11)
C37-C4-C5	120.4(9)	C40-C8-C9	119.1(10)
C3-C4-C5	120.5(11)	C14-C9-C8	118.3(11)
C6-C5-C39	117.5(9)	C14-C9-C10	121.0(10)

C8-C9-C10	120.7(11)	C20-C21-H21	121.0
C11-C10-C9	120.7(11)	C21-C22-C23	121.3(12)
C11-C10-C58	115.8(12)	C21-C22-H22	119.3
C9-C10-C58	123.2(10)	C23-C22-H22	119.3
C10-C11-C12	117.0(13)	C18-C23-C22	119.2(10)
C10-C11-C59	121.7(12)	C18-C23-C24	120.5(10)
C12-C11-C59	121.2(10)	C22-C23-C24	120.3(11)
C13-C12-C11	124.9(11)	C25-C24-C46	118.2(10)
C13-C12-H12	117.6	C25-C24-C23	122.2(10)
C11-C12-H12	117.6	C46-C24-C23	119.6(11)
C12-C13-C14	120.9(11)	C26-C25-C24	122.4(11)
C12-C13-C60	116.7(11)	C26-C25-H25	118.8
C14-C13-C60	122.4(11)	C24-C25-H25	118.8
C15-C14-C9	119.3(10)	C25-C26-C27	119.7(11)
C15-C14-C13	126.7(10)	C25-C26-H26	120.1
C9-C14-C13	114.0(12)	C27-C26-H26	120.1
C16-C15-C41	118.6(11)	C47-C27-C26	120.0(10)
C16-C15-C14	120.4(9)	C47-C27-C28	120.4(10)
C41-C15-C14	120.9(10)	C26-C27-C28	119.6(11)
C15-C16-C17	122.8(9)	C33-C28-C29	119.3(9)
C15-C16-H16	118.6	C33-C28-C27	119.0(10)
C17-C16-H16	118.6	C29-C28-C27	121.8(10)
C16-C17-C45	118.5(10)	C28-C29-C30	121.0(10)
C16-C17-C18	122.2(9)	C28-C29-H29	119.5
C45-C17-C18	119.3(10)	C30-C29-H29	119.5
C23-C18-C19	121.8(11)	C31-C30-C29	119.2(12)
C23-C18-C17	120.4(9)	C31-C30-H30	120.4
C19-C18-C17	117.7(11)	C29-C30-H30	120.4
C20-C19-C18	116.0(12)	C32-C31-C30	120.1(10)
C20-C19-H19	122.0	C32-C31-H31	120.0
C18-C19-H19	122.0	C30-C31-H31	120.0
C21-C20-C19	123.4(10)	C31-C32-C33	122.0(11)
C21-C20-H20	118.3	C31-C32-H32	119.0
C19-C20-H20	118.3	C33-C32-H32	119.0
C22-C21-C20	118.1(12)	C28-C33-C32	118.3(11)
C22-C21-H21	121.0	C28-C33-C34	120.1(9)

C32-C33-C34	121.6(10)	C46-C45-C17	118.7(10)
C35-C34-C48	116.2(11)	C24-C46-C45	121.3(9)
C35-C34-C33	122.1(9)	C24-C46-C47	119.8(11)
C48-C34-C33	121.6(10)	C45-C46-C47	118.8(10)
C36-C35-C34	123.4(9)	C27-C47-C48	121.8(10)
C36-C35-H35	118.3	C27-C47-C46	119.8(11)
C34-C35-H35	118.3	C48-C47-C46	118.3(11)
C35-C36-C44	120.7(10)	C43-C48-C34	121.4(10)
C35-C36-C37	122.5(9)	C43-C48-C47	121.6(9)
C44-C36-C37	116.8(10)	C34-C48-C47	116.9(11)
C4-C37-C38	118.9(10)	C54-C49-C50	119.9(11)
C4-C37-C36	120.9(9)	C54-C49-C1	120.0(12)
C38-C37-C36	120.2(11)	C50-C49-C1	119.9(11)
C1-C38-C37	122.6(11)	C49-C50-C51	115.7(11)
C1-C38-H38	118.7	C49-C50-C55	120.6(11)
C37-C38-H38	118.7	C51-C50-C55	123.6(12)
C5-C39-C44	120.5(9)	C52-C51-C50	124.8(13)
C5-C39-C40	120.5(11)	C52-C51-H51	117.6
C44-C39-C40	119.0(10)	C50-C51-H51	117.6
C8-C40-C41	120.4(9)	C51-C52-C53	117.8(12)
C8-C40-C39	119.0(10)	C51-C52-C56	123.7(14)
C41-C40-C39	120.6(11)	C53-C52-C56	117.9(13)
C15-C41-C40	120.2(11)	C52-C53-C54	120.6(13)
C15-C41-C42	119.6(10)	C52-C53-H53	119.7
C40-C41-C42	120.1(9)	C54-C53-H53	119.7
C45-C42-C41	122.0(9)	C49-C54-C53	120.8(13)
C45-C42-C43	119.1(10)	C49-C54-C57	121.1(11)
C41-C42-C43	118.9(9)	C53-C54-C57	118.1(12)
C48-C43-C44	120.1(9)	C50-C55-H55A	109.5
C48-C43-C42	119.1(10)	C50-C55-H55B	109.5
C44-C43-C42	120.8(10)	H55A-C55-H55B	109.5
C43-C44-C39	120.5(9)	C50-C55-H55C	109.5
C43-C44-C36	118.0(11)	H55A-C55-H55C	109.5
C39-C44-C36	121.6(9)	H55B-C55-H55C	109.5
C42-C45-C46	123.0(8)	C52-C56-H56A	109.5
C42-C45-C17	118.2(10)	C52-C56-H56B	109.5

H56A-C56-H56B	109.5	H58A-C58-H58C	109.5
C52-C56-H56C	109.5	H58B-C58-H58C	109.5
H56A-C56-H56C	109.5	C11-C59-H59A	109.5
H56B-C56-H56C	109.5	C11-C59-H59B	109.5
C54-C57-H57A	109.5	H59A-C59-H59B	109.5
C54-C57-H57B	109.5	C11-C59-H59C	109.5
H57A-C57-H57B	109.5	H59A-C59-H59C	109.5
C54-C57-H57C	109.5	H59B-C59-H59C	109.5
H57A-C57-H57C	109.5	C13-C60-H60A	109.5
H57B-C57-H57C	109.5	C13-C60-H60B	109.5
C10-C58-H58A	109.5	H60A-C60-H60B	109.5
C10-C58-H58B	109.5	C13-C60-H60C	109.5
H58A-C58-H58B	109.5	H60A-C60-H60C	109.5
C10-C58-H58C	109.5	H60B-C60-H60C	109.5

Table 16. Anisotropic displacement parameters ($Å^2$x 10^3) for **16a**. The anisotropic displacement factor exponent takes the form: $-2\pi^2[h^2 a^{*2}U^{11} + ... + 2 h k a^* b^* U^{12}]$

	U^{11}	U^{22}	U^{33}	U^{23}	U^{13}	U^{12}
C1	73(7)	25(5)	40(5)	15(4)	17(5)	13(5)
C2	91(8)	41(6)	53(6)	31(5)	23(6)	31(5)
C3	73(7)	48(6)	52(6)	20(5)	22(5)	24(5)
C4	51(6)	18(4)	41(5)	19(4)	4(4)	8(4)
C5	62(6)	34(5)	36(5)	4(4)	10(5)	20(5)
C6	73(7)	26(5)	54(6)	23(4)	22(5)	17(5)
C7	69(7)	52(6)	53(6)	20(5)	22(5)	18(5)
C8	58(6)	31(5)	49(6)	26(4)	21(5)	18(5)
C9	63(7)	44(6)	53(6)	12(5)	16(5)	16(5)
C10	85(8)	53(6)	69(7)	28(6)	27(6)	31(6)
C11	76(7)	57(6)	53(6)	18(5)	15(5)	21(6)
C12	83(8)	76(7)	55(6)	20(6)	25(6)	30(6)
C13	73(7)	46(6)	45(6)	19(5)	5(5)	18(5)
C14	71(7)	39(5)	55(6)	31(5)	12(5)	22(5)
C15	60(6)	43(5)	37(5)	24(4)	17(5)	21(5)
C16	59(6)	38(5)	45(6)	21(5)	19(5)	8(5)
C17	52(6)	38(5)	34(5)	15(4)	10(4)	14(5)
C18	59(6)	43(6)	45(6)	23(5)	16(5)	19(5)
C19	71(7)	40(6)	60(6)	29(5)	12(5)	15(5)
C20	79(7)	56(6)	62(6)	44(5)	28(6)	27(5)
C21	85(7)	45(6)	71(7)	36(5)	17(6)	20(5)
C22	74(7)	45(6)	62(6)	28(5)	32(5)	25(5)
C23	60(6)	35(5)	47(6)	21(5)	11(5)	13(5)
C24	56(6)	32(5)	44(5)	16(4)	12(5)	16(5)
C25	66(6)	35(5)	58(6)	21(5)	9(5)	18(5)
C26	72(7)	43(6)	45(6)	7(5)	21(5)	20(5)
C27	52(6)	39(5)	52(6)	23(5)	14(5)	15(5)
C28	68(6)	23(5)	46(6)	23(4)	27(5)	22(5)
C29	71(7)	39(5)	55(6)	18(5)	8(5)	22(5)
C30	58(6)	49(6)	49(6)	13(5)	14(5)	14(5)
C31	67(7)	51(6)	52(6)	18(5)	21(5)	23(5)

C32	66(6)	43(6)	53(6)	20(5)	20(5)	24(5)
C33	62(6)	38(5)	36(5)	13(4)	11(5)	8(5)
C34	55(6)	36(5)	42(5)	20(5)	7(5)	11(5)
C35	53(6)	32(5)	44(5)	14(4)	16(5)	14(5)
C36	54(6)	26(5)	56(6)	27(5)	11(5)	14(4)
C37	62(6)	29(5)	44(6)	20(4)	17(5)	10(5)
C38	63(6)	38(5)	45(5)	23(4)	23(5)	14(5)
C39	58(6)	32(5)	41(5)	26(4)	19(5)	15(5)
C40	50(6)	38(5)	39(5)	20(4)	14(5)	7(5)
C41	47(6)	37(5)	50(6)	14(5)	15(5)	11(5)
C42	61(6)	31(5)	34(5)	23(4)	11(5)	15(5)
C43	46(6)	32(5)	41(5)	1(4)	16(5)	4(5)
C44	50(6)	26(5)	41(5)	21(4)	4(4)	11(4)
C45	59(6)	37(5)	24(5)	20(4)	17(4)	18(5)
C46	53(6)	28(5)	35(5)	18(4)	1(5)	9(5)
C47	59(6)	44(6)	45(6)	15(5)	18(5)	18(5)
C48	49(6)	23(5)	37(5)	20(4)	2(4)	4(4)
C49	82(7)	41(6)	63(6)	15(5)	24(6)	28(5)
C50	79(7)	40(5)	58(6)	13(5)	8(5)	24(5)
C51	71(7)	46(6)	68(7)	11(5)	12(5)	25(5)
C52	95(8)	63(7)	68(7)	27(6)	30(6)	40(6)
C53	96(8)	71(7)	67(7)	29(6)	19(6)	26(6)
C54	82(7)	50(6)	64(7)	25(5)	13(6)	26(6)
C55	72(9)	51(7)	79(9)	-6(6)	6(7)	13(7)
C56	155(13)	55(7)	116(11)	44(7)	84(10)	40(8)
C57	113(11)	68(8)	58(8)	24(6)	5(7)	38(8)
C58	86(9)	69(8)	68(8)	33(6)	23(7)	38(7)
C59	108(11)	53(7)	61(8)	22(6)	22(7)	9(8)
C60	109(11)	61(8)	71(8)	34(7)	18(8)	19(8)

Table 17. Hydrogen coordinates (x 10^4) and isotropic displacement parameters ($Å^2$x 10^3) for 1.

	x	y	z	U(eq)
H2	7064	10673	3197	69
H3	5856	9711	4356	66
H6	4848	8779	5347	60
H7	4215	8033	6704	69
H12	3294	5168	9563	83
H16	5812	3895	7851	58
H19	6209	2652	8168	68
H20	6660	1101	8474	75
H21	7882	408	7518	79
H22	8410	1136	6119	68
H25	8987	1810	4955	63
H26	9626	2571	3585	63
H29	10119	3209	2409	65
H30	10927	4060	1077	63
H31	10554	5628	773	66
H32	9617	6402	1831	61
H35	8736	7047	2753	51
H38	8737	8490	2601	58
H51	11982	12020	1809	72
H53	8512	10862	-88	93
H55A	10755	10330	3546	105
H55B	10607	11447	3730	105
H55C	12042	11374	3358	105
H56A	12203	12543	283	152
H56B	10687	12595	-187	152
H56C	11215	11670	-510	152
H57A	6580	8861	796	117
H57B	6271	9798	287	117
H57C	6007	9640	1351	117
H58A	3882	8360	8101	105

H58B	5520	8554	7907	105
H58C	5138	8531	8953	105
H59A	4080	7584	10170	117
H59B	2602	6588	10065	117
H59C	2750	7529	9407	117
H60A	3354	3601	9095	123
H60B	4972	3773	8871	123
H60C	3636	3394	8053	123

Table 18. Torsion angles [°] for **16a**.

C38-C1-C2-C3	-3(2)	C12-C13-C14-C15	172.8(13)
C49-C1-C2-C3	-174.3(12)	C60-C13-C14-C15	-7(2)
C1-C2-C3-C4	3(2)	C12-C13-C14-C9	-9.5(18)
C2-C3-C4-C37	-2.1(17)	C60-C13-C14-C9	171.0(11)
C2-C3-C4-C5	173.6(11)	C9-C14-C15-C16	167.6(11)
C37-C4-C5-C6	-178.3(11)	C13-C14-C15-C16	-15(2)
C3-C4-C5-C6	6.1(18)	C9-C14-C15-C41	-7.6(18)
C37-C4-C5-C39	1.4(17)	C13-C14-C15-C41	170.0(12)
C3-C4-C5-C39	-174.2(11)	C41-C15-C16-C17	-5.3(18)
C39-C5-C6-C7	8.7(18)	C14-C15-C16-C17	179.4(11)
C4-C5-C6-C7	-171.6(12)	C15-C16-C17-C45	4.9(17)
C5-C6-C7-C8	-1.5(19)	C15-C16-C17-C18	-176.7(11)
C6-C7-C8-C40	-7.8(19)	C16-C17-C18-C23	-179.1(11)
C6-C7-C8-C9	175.4(11)	C45-C17-C18-C23	-0.8(18)
C7-C8-C9-C14	161.6(12)	C16-C17-C18-C19	-2.6(18)
C40-C8-C9-C14	-15.2(17)	C45-C17-C18-C19	175.8(11)
C7-C8-C9-C10	-16(2)	C23-C18-C19-C20	-3.0(18)
C40-C8-C9-C10	167.0(11)	C17-C18-C19-C20	-179.5(11)
C14-C9-C10-C11	-12(2)	C18-C19-C20-C21	3(2)
C8-C9-C10-C11	165.4(13)	C19-C20-C21-C22	-4(2)
C14-C9-C10-C58	161.0(12)	C20-C21-C22-C23	4(2)
C8-C9-C10-C58	-21(2)	C19-C18-C23-C22	3.5(19)
C9-C10-C11-C12	3(2)	C17-C18-C23-C22	179.9(11)
C58-C10-C11-C12	-171.3(12)	C19-C18-C23-C24	-177.6(11)
C9-C10-C11-C59	-174.0(13)	C17-C18-C23-C24	-1.2(18)
C58-C10-C11-C59	12(2)	C21-C22-C23-C18	-4(2)
C10-C11-C12-C13	3(2)	C21-C22-C23-C24	177.1(12)
C59-C11-C12-C13	179.9(13)	C18-C23-C24-C25	-177.0(12)
C11-C12-C13-C14	0(2)	C22-C23-C24-C25	1.9(18)
C11-C12-C13-C60	179.9(13)	C18-C23-C24-C46	0.8(17)
C8-C9-C14-C15	15.3(18)	C22-C23-C24-C46	179.7(11)
C10-C9-C14-C15	-166.9(12)	C46-C24-C25-C26	1.3(18)
C8-C9-C14-C13	-162.7(11)	C23-C24-C25-C26	179.1(11)
C10-C9-C14-C13	15.2(18)	C24-C25-C26-C27	1.6(19)

C25-C26-C27-C47	-2.1(18)	C36-C37-C38-C1	179.6(11)
C25-C26-C27-C28	179.1(11)	C6-C5-C39-C44	174.0(11)
C47-C27-C28-C33	1.7(18)	C4-C5-C39-C44	-5.7(17)
C26-C27-C28-C33	-179.4(12)	C6-C5-C39-C40	-6.5(17)
C47-C27-C28-C29	-176.6(12)	C4-C5-C39-C40	173.7(11)
C26-C27-C28-C29	2.2(18)	C7-C8-C40-C41	-169.6(11)
C33-C28-C29-C30	3.5(18)	C9-C8-C40-C41	7.4(17)
C27-C28-C29-C30	-178.1(11)	C7-C8-C40-C39	9.6(17)
C28-C29-C30-C31	-3.0(18)	C9-C8-C40-C39	-173.5(11)
C29-C30-C31-C32	2.6(19)	C5-C39-C40-C8	-2.6(17)
C30-C31-C32-C33	-2.7(19)	C44-C39-C40-C8	176.9(11)
C29-C28-C33-C32	-3.5(18)	C5-C39-C40-C41	176.5(11)
C27-C28-C33-C32	178.1(11)	C44-C39-C40-C41	-4.0(17)
C29-C28-C33-C34	178.5(11)	C16-C15-C41-C40	-175.7(11)
C27-C28-C33-C34	0.1(17)	C14-C15-C41-C40	-0.4(18)
C31-C32-C33-C28	3.1(19)	C16-C15-C41-C42	2.0(17)
C31-C32-C33-C34	-178.9(12)	C14-C15-C41-C42	177.3(11)
C28-C33-C34-C35	178.9(11)	C8-C40-C41-C15	0.5(17)
C32-C33-C34-C35	1.0(18)	C39-C40-C41-C15	-178.6(11)
C28-C33-C34-C48	-3.4(18)	C8-C40-C41-C42	-177.2(11)
C32-C33-C34-C48	178.6(11)	C39-C40-C41-C42	3.7(17)
C48-C34-C35-C36	-2.1(17)	C15-C41-C42-C45	1.5(18)
C33-C34-C35-C36	175.7(12)	C40-C41-C42-C45	179.3(11)
C34-C35-C36-C44	0.0(18)	C15-C41-C42-C43	179.1(11)
C34-C35-C36-C37	-179.5(11)	C40-C41-C42-C43	-3.2(16)
C3-C4-C37-C38	1.3(17)	C45-C42-C43-C48	1.1(16)
C5-C4-C37-C38	-174.4(11)	C41-C42-C43-C48	-176.5(11)
C3-C4-C37-C36	-179.5(11)	C45-C42-C43-C44	-179.3(11)
C5-C4-C37-C36	4.8(17)	C41-C42-C43-C44	3.0(16)
C35-C36-C37-C4	173.0(11)	C48-C43-C44-C39	176.2(10)
C44-C36-C37-C4	-6.6(17)	C42-C43-C44-C39	-3.4(17)
C35-C36-C37-C38	-7.8(18)	C48-C43-C44-C36	-4.3(16)
C44-C36-C37-C38	172.6(11)	C42-C43-C44-C36	176.1(10)
C2-C1-C38-C37	1.8(19)	C5-C39-C44-C43	-176.7(11)
C49-C1-C38-C37	173.3(11)	C40-C39-C44-C43	3.8(17)
C4-C37-C38-C1	-1.2(18)	C5-C39-C44-C36	3.8(17)

C40-C39-C44-C36	-175.7(11)	C33-C34-C48-C47	4.7(16)
C35-C36-C44-C43	3.2(17)	C27-C47-C48-C43	178.8(11)
C37-C36-C44-C43	-177.2(10)	C46-C47-C48-C43	-0.1(17)
C35-C36-C44-C39	-177.3(11)	C27-C47-C48-C34	-2.9(17)
C37-C36-C44-C39	2.3(16)	C46-C47-C48-C34	178.2(10)
C41-C42-C45-C46	175.1(11)	C38-C1-C49-C54	97.5(16)
C43-C42-C45-C46	-2.5(17)	C2-C1-C49-C54	-91.2(15)
C41-C42-C45-C17	-1.9(17)	C38-C1-C49-C50	-77.5(16)
C43-C42-C45-C17	-179.5(10)	C2-C1-C49-C50	93.9(15)
C16-C17-C45-C42	-1.2(16)	C54-C49-C50-C51	1.8(18)
C18-C17-C45-C42	-179.6(11)	C1-C49-C50-C51	176.8(11)
C16-C17-C45-C46	-178.3(11)	C54-C49-C50-C55	179.1(12)
C18-C17-C45-C46	3.3(16)	C1-C49-C50-C55	-5.9(18)
C25-C24-C46-C45	179.7(11)	C49-C50-C51-C52	-5(2)
C23-C24-C46-C45	1.8(17)	C55-C50-C51-C52	177.6(12)
C25-C24-C46-C47	-3.6(17)	C50-C51-C52-C53	7(2)
C23-C24-C46-C47	178.5(10)	C50-C51-C52-C56	177.3(13)
C42-C45-C46-C24	179.3(11)	C51-C52-C53-C54	-6(2)
C17-C45-C46-C24	-3.8(16)	C56-C52-C53-C54	-176.6(13)
C42-C45-C46-C47	2.6(17)	C50-C49-C54-C53	-1(2)
C17-C45-C46-C47	179.5(10)	C1-C49-C54-C53	-175.9(12)
C26-C27-C47-C48	-179.1(11)	C50-C49-C54-C57	177.0(12)
C28-C27-C47-C48	-0.3(18)	C1-C49-C54-C57	2.1(19)
C26-C27-C47-C46	-0.3(18)	C52-C53-C54-C49	3(2)
C28-C27-C47-C46	178.6(11)	C52-C53-C54-C57	-175.1(12)
C24-C46-C47-C27	3.2(17)		
C45-C46-C47-C27	179.9(11)		
C24-C46-C47-C48	-177.9(10)		
C45-C46-C47-C48	-1.2(16)		
C44-C43-C48-C34	2.3(16)		
C42-C43-C48-C34	-178.1(11)		
C44-C43-C48-C47	-179.4(11)		
C42-C43-C48-C47	0.2(16)		
C35-C34-C48-C43	0.9(16)		
C33-C34-C48-C43	-176.9(11)		
C35-C34-C48-C47	-177.5(10)		

Figure 3. View of **16a** showing the atom labeling scheme. Displacement ellipsoids are scaled to the 50% probability level.

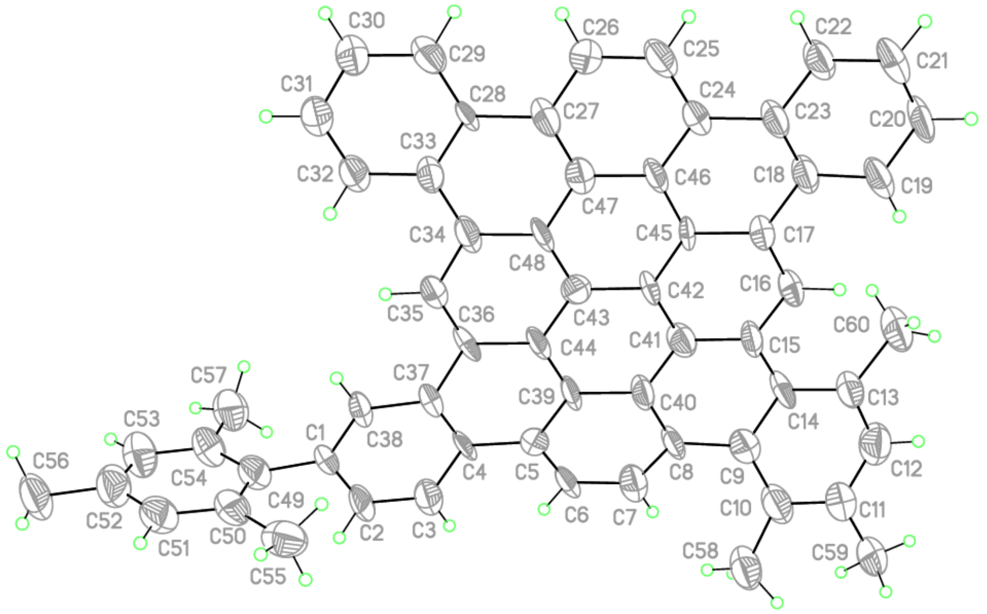

References

[i] Matthews, J. M.; Qin, N.; Colburn, R. W., Dax, S. L.; Hawkins, M.; McNally, J. J.; Reany, L.; Youngman, M. A.; Baker, J.; Hutchinson, T.; Liu, Y.; Lubin, M. L.; Neeper, M.; Brandt, M. R.; Stone, D. J.; Flores, C. M. *Bioorg. Med. Chem. Lett.* **2012**, *22*, 2922.

[ii] Barawkar, D. A.; Meru, A.; Bandyopadhyay, A.; Banerjee, A.; Deshpande, A. M.; Athare, C.; Koduru, C.; Khose, G.; Gundu, J.; Mahajan, K.; Patil, P.; Kandalkar, S. R.; Niranjan, S.; Bhosale, S.; De, S.; Mukhopadhyay, S.; Chaudhary, S.; Koul, S.; Singh, U.; Chugh, A.; Palle, V. P.; Mookhtiar, K. A.; Vacca, J.; Chakravarty, P. K.; Nargund, R. P.; Wright, S. D.; Roy, S.; Granziano, M. P.; Singh, S. B.; Cully, D.; Cai, T. –Q. *ACS Med. Chem. Lett.* **2011**, *2*, 919.

[iii] Mata, J. A.; Falomir, E.; Llusar, R.; Peris, E. *J. Organomet. Chem.* **2000**, 616, 80.

[iv] (a) Katz, H. E.; Bent, S. F.; Wilson, W. L.; Schilling, M. L.; Ungashe, S. B. *J. Am. Chem. Soc.* **1994**, *116*, 6631; (b) Tanner, D.; Wennerstròm, O. *Acta Chem. Scand.* **1980**, *B34*, 529.

[v] Bolag, A.; Lopez-Andarias, J.; Lascano, S.; Soleimanpour, S.; Atienza, C.; Sakai, N.; Martin, N.; Matile, S.

Angew. Chem. Int. Ed. **2014**, *53*, 4890.

[vi] Ozasa, S.; Hatada, N.; Fujioka, Y.; Ibuki, E. *Bull. Chem. Soc. Jpn.* **1980**, *53*, 2610.

[vii] Zuo, Z.; Liu, J.; Nan, J.; Fan, L.; Sun, W.; Wang, Y.; Luan, X. *Angew. Chem. Int. Ed.* **2015**, *54*, 15385.

[viii] Oyama, T.; Yang, Y. S.; Matsuo, K.; Tasuda, T. *Chem. Commun.* **2017**, *53*, 3814.

[ix] Birrell, J. A.; Desrosiers, J.-N.; Jacobsen, E. N. *J. Am. Chem. Soc.* **2011**, *133*, 13875.

[x] Filthaus, M.; Oppel, I. M.; Bettinger, H. F. *Org. Biomol. Chem.* **2008**, *6*, 1201.

[xi] (a) Kübel, C.; Eckhardt, K.; Enkelmann, V.; Wegner, G.; Müllen, K. *J. Mater. Chem.*, **2000**, *10*, 879-886. (b) Balaban, A. T.; Klein, D. J. *J. Phys. Chem. C* **2009**, *113*, 19123-19133. (c) Liu, Y.-M.; Hou, H.; Zhao, X.-J.; Tang, C.; Tan, Y.-Z.; Müllen, K. *Nat. Comm.* **2018**, *9*, 1901.

[xii] (a) Clar, E.; Ironside, C. T.; Zander, M. *J. Chem. Soc.* **1959**, 142-147. (b) Kübel, C.; Eckhardt, K.; Enkelmann, V.; Wegner, G.; Müllen, K. *J. Mater. Chem.*, **2000**, *10*, 879-886. (c) Dötz, F.; Brand, J. D.; Ito, S.; Gherghel, L.; Müllen, K. *J. Am. Chem. Soc.* **2000**, *122*, 7707-7717. (d) Ruiterkamp, R.; Halasinski, T.; Salama, F.; Foing, B. H.; Allamandola, L. J.; Schmidt, W.; Ehrenfreund, P. *A&A* **2002**, *390*, 1153-1170 (e) Kastler, M.; Schmidt, J.; Pisula, W.; Sebastiani, D.; Müllen, K. *J. Am. Chem. Soc.* **2006**, *128*, 9526-9534. (f) Sk, M. A.; Ananthanarayanan, A.; Huang, L.; Lim, K. H.; Chen, P. *J. Mater. Chem. C* **2014**, *2*, 6954.

[xiii] Clar, E.; Fell, G. S.; Ironside, C. T.; Balsillie, A. *Tetrahedron* **1960**, *10*, 26-36.

[xiv] Liu, Y. PhD Dissertation, Max-Planck-Institut für Polymerforschung, Mainz, Germany, **2017**.

[xv] (a) Yamaguchi, R.; Hiroto, S.; Shinokubo, H. *Org. Lett.* **2012**, *14*, 2472-2475. (b) Yamaguchi, R. PhD Dissertation, Nagoya University, Chikusa-ku, Nagoya, Japan, **2014**.

[xvi] (a) Wu, J.; Qu, J.; Tchebotareva, N.; Müllen, K. *Tetrahedron Letters* **2005**, *46*, 1565-1568. (b) Kim, K.-Y.; Liu, S.; Köse, M. E.; Schanze, K. S. *Inorg. Chem.* **2006**, *45*, 2509-2519. (c) Chen, C.-W.; Chang, H.-Y.; Lee, S.-L.; Hsu, I-J.; Lee, J.-J. Chen, C.-h.; Luh, T.-Y. *Macromolecules* **2010**, *43*, 8741-8746. (d) Murphy, F. A.; Draper, S. M. *J. Org. Chem.* **2010**, *75*, 1862-1870. (e) Yamaguchi, R. PhD Dissertation, Nagoya University, Chikusa-ku, Nagoya, Japan, **2014**. (f) Hirose, T.; Miyazaki, Y.; Watabe, M.; Akimoto, S.; Tachikawa, T.; Kodama, K.; Yasutake, M. *Tetrahedron* **2015**, *71*, 4714-4721.
